# Fifth Universal Definition of Myocardial Infarction (2026): On behalf of the Joint European Society of Cardiology (ESC)/American College of Cardiology (ACC)/American Heart Association (AHA)/World Heart Federation (WHF) Task Force for the Universal Definition of Myocardial Infarction

**DOI:** 10.5334/gh.1578

**Published:** 2026-08-28

**Authors:** Nicholas L. Mills, L. Kristin Newby, Sarah Zaman, Wael A. Almahmeed, Chiara Bucciarelli-Ducci, Monica M. Colvin, George D. Dangas, Holli A. DeVon, Rohan Dharmakumar, Thomas A. Gaziano, Derek J. Hausenloy, Mohamed Jeilan, Konstantinos C. Koskinas, Bertil Lindahl, Zuzana Motovska, Maria Rubini Gimenez, Ana María Salvati, Jacqueline E. Tamis-Holland, ESC/ACC/AHA/WHF Universal Definition Document Advisory Group

**Affiliations:** 1 ESC Chairperson, United Kingdom; 2 ACC/AHA Co-Chairperson, United States of America; 3 WHF Co-Chairperson, Australia; 4 Heart Vascular and Thoracic Institute, Cleveland Clinic, Abu Dhabi, United Arab Emirates; 5 Cardiology and Cardiac MRI Department, Royal Brompton and Harefield Hospitals, Guys and St Thomas NHS Foundation Trust, London, United Kingdom; 6 National Heart and Lung Institute, Imperial College London, London, United Kingdom; 7 School of Biomedical Engineering and Imaging Sciences, Kings College London, London, United Kingdom; 8 Cardiovascular Division, University of Michigan, Ann Arbor, MI, United States of America; 9 Cardiology, Mount Sinai Medical Center, New York, NY, United States of America; 10 School of Nursing, University of California Los Angeles, Los Angeles, United States of America; 11 Cardiovascular Imaging Research Center, Medical Imaging Research Institute, Department of Radiology and Imaging Sciences, Indiana University School of Medicine, Indianapolis, IN, United States of America; 12 Division of Cardiovascular Medicine, Department of Medicine, Indiana University School of Medicine, Indianapolis, IN, United States of America; 13 Cardiovascular Medicine, Brigham and Women’s Hospital, Boston, MA, United States of America; 14 Health Policy and Management, Harvard School of Public Health, Boston, MA, United States of America; 15 Medicine, Harvard Medical School, Boston, MA, United States of America; 16 Cardiovascular and Metabolic Disorders Program, Duke-NUS Medical School, Singapore, Singapore; 17 National Heart Research Institute, Singapore; 18 National Heart Centre Singapore, Singapore; 19 The Hatter Cardiovascular Institute, University College London, London, United Kingdom; 20 Department of Medicine, Aga Khan University Hospital, Nairobi, Kenya; 21 Department of Cardiology, Bern University Hospital, Inselspital, Bern, Switzerland; 22 Department of Medical Sciences, Uppsala University, Uppsala, Sweden; 23 Cardiocenter, Third Faculty of Medicine, Charles University and University Hospital Kralovske Vinohrady, Prague, Czech Republic; 24 Cardiology Department, Centro Nacional de Investigaciones Cardiovasculares (CNIC), Madrid, Spain; 25 Cardiology Department, Ascires Biomedical Group, Valencia, Spain; 26 Cardiology, Clínica Modelo de Morón, Buenos Aires, Argentina; 27 Argentine Society of Cardiology, Buenos Aires, Argentina; 28 Cardiovascular Medicine, Cleveland Clinic, Heart, Vascular and Thoracic Institute, Cleveland, OH, United States of America

**Keywords:** Universal Definition, Expert Consensus Document, Myocardial infarction, Primary myocardial infarction, Secondary myocardial infarction, Procedure-related myocardial infarction, Cardiac troponin, High-sensitivity cardiac troponin, Acute myocardial injury, Chronic myocardial injury, Unrecognized myocardial infarction, Unstable angina, Recurrent myocardial infarction, Coronary and cardiac imaging, Takotsubo syndrome, Myocardial injury with non-obstructive coronary arteries (MINOCA), International Classification of Diseases 11th Revision (ICD-11)

## Abbreviations and Acronyms

ACC: American College of Cardiology
ACS: Acute coronary syndrome
AHA: American Heart Association
AI: Artificial intelligence
CAD: Coronary artery disease
CABG: Coronary artery bypass graft(ing)
CCTA: Coronary computed tomography angiography
CK-MB: Creatine kinase-muscle/brain isoenzyme
CKD: Chronic kidney disease
CMR: Cardiac magnetic resonance
cMyC: Cardiac myosin-binding protein
CCT: Computed tomography
ECG: Electrocardiograme
GFR: Estimated glomerular filtration rate
ESC: European Society of Cardiology
FFR: Fractional flow reserve
ICD: International Classification of Diseasesi
FR: Instantaneous wave-free ratio
IVUS: Intravascular ultrasound
LBBB: Left bundle branch block
LGE: Late gadolinium enhancement
LMICs: Low- and middle-income countries
MINOCA: Myocardial injury with non-obstructive coronary arteries
MINS: Myocardial injury after non-cardiac surgery
NSTEMI: Non-ST-segment elevation myocardial infarction
OCT: Optical coherence tomography
PCI: Percutaneous coronary intervention
PET: Positron emission tomography
PMI: Peri-operative myocardial injury
RFR: Resting full-cycle ratio
SCAD: Spontaneous coronary artery dissection
SPECT: Single photon emission computed tomography
STEMI: ST-segment elevation myocardial infarction
TAVI: Transcatheter aortic valve implantation
UDMI: Universal definition of myocardial infarction
URL: Upper reference limit
WHF: World Heart Federation

## 1. Executive summary

The Fifth Universal Definition of Myocardial Infarction (UDMI) updates the classification to better reflect underlying pathophysiology, align with clinical evaluation of patients, and incorporate objective diagnostic criteria. This classification recognizes that myocardial infarction occurs in three clinical settings. First, myocardial infarction can arise spontaneously due to an acute coronary pathology (**primary myocardial infarction**). While most presentations are due to atherothrombosis, alternative coronary pathologies can be identified using coronary imaging and functional testing. Second, myocardial infarction may result from another acute condition causing oxygen supply–demand imbalance (**secondary myocardial infarction**). Here, objective criteria are proposed to guide the use of cardiac and invasive or non-invasive coronary imaging, promoting consistent diagnosis and informing treatment. Third, myocardial infarction may occur as a complication of percutaneous or surgical cardiac procedures (**procedure-related myocardial infarction**). The diagnosis of procedure-related myocardial infarction does not rely on cardiac biomarker thresholds and is comparable for percutaneous and surgical procedures. In each of these three settings, guidance is provided on the interpretation of symptoms and signs of myocardial ischaemia, cardiac biomarkers, and imaging. Alignment of the classification of myocardial infarction with International Disease Classification (ICD)-11 codes, its distinction from myocardial injury, and implications for patients, public health, and research are discussed. It is anticipated that adoption of the classification in practice and research will ensure the diagnosis of myocardial infarction is meaningful for patients and clinicians with clear implications for care across healthcare settings.

## 2. Context

The Fifth UDMI is a joint statement from the European Society of Cardiology (ESC), the American College of Cardiology (ACC), the American Heart Association (AHA), and the World Heart Federation (WHF). This is a consensus statement and should be applied in conjunction with national and regional clinical practice guidelines (1,2,3). The statement was written by a global task force consisting of a multidisciplinary group of experts with representation from 12 countries across 5 continents. It has undergone independent peer review by a committee comprising 18 members selected by the above societies as well as by a panel of patients and representatives selected from the European Association for Cardio-Thoracic Surgery (EACTS), the Society of Thoracic Surgeons (STS), Clinical Practice Guidelines Committee and National Cardiac Societies of the ESC. Following this writing and peer review process the document was approved for publication.

The work of this task force builds on the success of prior consensus statements, which have improved the standardization of the diagnosis of myocardial infarction worldwide. The first international task force to define the diagnosis of myocardial infarction was established by the World Health Organization (WHO) in 1979, when the diagnosis was based on symptoms, electrocardiographic criteria, and cardiac enzymes, but excluded coronary angiography (4). This was updated in 2000, when the consensus statement was led by the ESC and ACC. This document was subsequently considered to be the first universal definition (5). Revision was necessary with the introduction of cardiac troponin testing and increasing use of coronary angiography in practice, and subsequent iterations in 2007 (6), 2012 (7), and 2018 (8) addressed the opportunities and challenges introduced by the development of high-sensitivity cardiac troponin assays and further advances in cardiac imaging. With these iterations, the UDMI has encouraged clinicians to consider the underlying mechanism of myocardial infarction and myocardial injury, stimulated research, and promoted the use of sex-specific diagnostic criteria. This important international scientific effort will continue to require regular revision as new evidence emerges.

Making the diagnosis of myocardial infarction promptly and accurately is important as treatment improves survival. Myocardial infarction can have a substantial impact on future health, well-being, and livelihood. Disruption of an atherosclerotic plaque resulting in thrombosis (atherothrombosis) is the predominant cause of myocardial infarction. However, due to greater access to coronary angiography, intravascular imaging, and functional testing, other acute coronary pathologies, such as spontaneous coronary artery dissection, coronary embolism, and vasospasm are being increasingly identified. Treatment varies depending on the underlying mechanism, but the initial approach to evaluation of a patient with suspected myocardial infarction is similar. In parallel, cardiac troponin testing has become widely available in both ambulatory and hospital settings, and with this, our recognition of alternative causes of acute and chronic myocardial injury.

The classification of disease should be clinically relevant, widely applicable, and inform the approach to management and prognosis. To achieve these goals the classification of myocardial infarction needs to reflect the underlying pathophysiology with objective and reproducible diagnostic criteria that can be consistently applied in practice. This is necessary to establish trust and ensure the implications of the diagnosis are clearly understood by both clinicians and the person whom it affects. To support adoption in practice, ICD codes that align with the classification are required. It also needs to recognize that not all healthcare systems have access to the coronary or cardiac imaging modalities that are required to identify the underlying mechanism and to confirm the diagnosis of myocardial infarction (9).

## 3. Objective

The Fifth Universal Definition of Myocardial Infarction provides an updated, evidence-based definition and classification of myocardial infarction to support consistent diagnosis in both clinical practice and research. A simplified classification based on the underlying pathophysiology and clinical context is proposed, with objective criteria to guide treatment.

The Universal Definition was developed jointly by an ESC/ACC/AHA/WHF Task Force, under the auspices of the ESC Clinical Practice Guidelines Committee, in accordance with established ESC processes agreed by the participating societies, and is endorsed by the EACTS and STS, with affirmation of value by SCAI. The Task Force worked collaboratively with the World Health Organization (WHO) on the clinical taxonomy and proposed ICD-11 codes which were under review by the Classification and Statistics Advisory Committee of the WHO Family of International Classifications at the time of publication. A summary of studies referenced in support of the statement are included in the Supplementary data online *Evidence Tables*.

## 4. What is new

The classification of myocardial infarction in the Third UDMI and the expansion in the Fourth UDMI were major advances, though some aspects remained difficult to apply consistently in practice. This is particularly true when myocardial infarction results from another acute illness or a coronary process other than atherothrombosis (previously type 2) or arises following coronary intervention (previously type 4a–c) or cardiac surgery (previously type 5).

**Figure 1:**
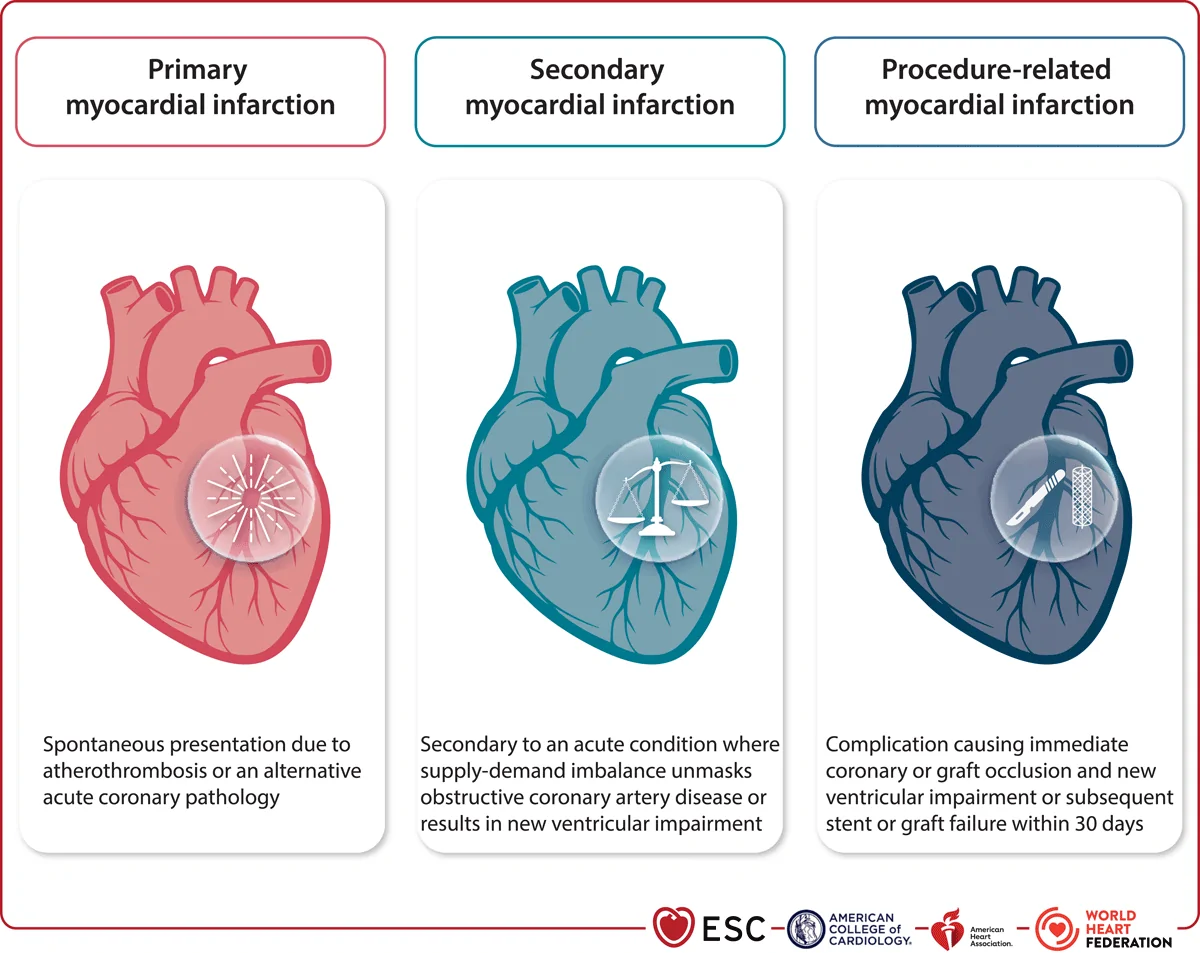
Central illustration. Fifth Universal Definition of Myocardial Infarction. Myocardial infarction can occur in three clinical settings: spontaneously due to a primary acute coronary pathology, secondary to an acute condition, or following a cardiac procedure.

A new clinical classification of myocardial infarction is proposed (Figure 1) based on pathophysiology, aligned with clinical evaluation of patients and supported by objective diagnostic criteria. Key updates from the Fourth UDMI are outlined in Table 1.

**Table 1:** Comparison of the Fourth and Fifth Universal Definitions of Myocardial Infarction.


FOURTH UDMI	FIFTH UDMI—WHAT’S NEW	RATIONALE FOR CHANGE

**Numerical classification** of myocardial infarction: types 1, 2, 3, 4a, 4b, 4c, 5.	**Clinical classification** of myocardial infarction; **primary, secondary**, or **procedure related**.	To reflect underlying pathophysiology, align with clinical evaluation, and enable consistent application.

**Type 1 myocardial infarction** Restricted to atherothrombosis.	**Primary myocardial infarction** Includes all acute coronary pathologies: atherothrombosis; spontaneous coronary artery dissection; coronary embolism; vasospasm; and restenosis, stent thrombosis, or graft failure >30 days from procedure.	Prioritizes sensitivity to avoid missing any primary acute coronary pathology. Promotes coronary angiography and adjunctive testing to confirm diagnosis and identify underlying acute coronary pathology with new diagnostic codes for each mechanism. Reclassifies late stent/graft failure as *de novo* disease, not a procedure-related complication.

**Type 2 myocardial infarction** Myocardial oxygen supply–demand imbalance due to either: Acute coronary pathology other than atherothrombosis, including spontaneous coronary artery dissection, coronary embolism, and vasospasm *or* An alternative acute condition with or without coronary artery disease.	**Secondary myocardial infarction** Myocardial oxygen supply–demand imbalance due to an alternative acute condition: *and* obstructive coronary artery disease without acute coronary pathology *and/or* new or presumed new regional wall motion abnormality or absence of viable myocardium.	Prioritize specificity to differentiate myocardial infarction from acute myocardial injury in conditions resulting in oxygen supply–demand imbalance. Objective diagnostic criteria to allow consistent application in practice and identify patients in whom the diagnosis has treatment implications.

**Type 3 myocardial infarction** Cardiac death where myocardial infarction is the likely cause, but death occurs before diagnostic testing is performed.	Term removed. Where myocardial infarction is the likely cause of death, clinical classification (primary, secondary, or procedure-related) should be applied, based on setting or post-mortem findings.	Limited use in clinical practice.

**Type 4 and 5 myocardial infarction** Type 4a myocardial infarction: cardiac troponin concentration >5 times the 99th percentile with myocardial ischaemia, imaging evidence of new loss of viable myocardium, *or* angiographic evidence of a procedural complication within 48 h. Type 4b due to stent thrombosis and type 4c due to stent restenosis, at any time after the procedure. Type 5 myocardial infarction: coronary artery bypass graft-related; cardiac troponin >10 times the 99th percentile, with new pathological Q waves, imaging evidence of new loss of viable myocardium, *or* angiographic evidence of a graft occlusion within 48 h.	**Procedure-related myocardial infarction** Coronary complication within 30 days of a cardiac procedure resulting in acute myocardial injury with one or more features. Both features are required when the complication arises during the procedure or in the setting of acute myocardial infarction. Evidence of a coronary complication of percutaneous intervention or cardiac surgery New or presumed new regional wall motion abnormality or absence of viable myocardium.	Prioritizes specificity to ensure the diagnosis is clinically meaningful, with objective cardiac imaging criteria. Diagnostic criteria the same for all cardiac procedures to enable meaningful comparisons. Excludes late stent/graft failure as a procedural complication as it typically reflects *de novo* disease.


UDMI, universal definition of myocardial infarction.

New or updated concepts:

- The criteria for acute and chronic myocardial injury are refined and the underlying mechanisms discussed (see Section 6). Sex-specific 99th percentile upper reference limits for cardiac troponin define myocardial injury to avoid systematic bias and the under-recognition of both myocardial infarction and other cardiac conditions associated with myocardial injury in female patients.
- The previous numerical classification of myocardial infarction has been replaced. Myocardial infarction is now classified into one of three clinical types:
  - **Primary myocardial infarction:** spontaneous presentation due to a primary acute coronary pathology
  - **Secondary myocardial infarction:** resulting from myocardial oxygen supply–demand imbalance due to another acute condition
  - **Procedure-related myocardial infarction:** occurring as a complication of a percutaneous or surgical cardiac procedure.
- Primary myocardial infarction is likely when symptoms or signs of acute myocardial ischaemia and injury arise spontaneously, and no alternative acute condition or cardiac procedure has triggered the presentation. The diagnosis is confirmed and aetiology defined if atherothrombosis or an alternative acute coronary pathology is identified (see Section 7.2.1).
- Secondary myocardial infarction is considered when acute myocardial ischaemia and injury is secondary to an alternative acute condition resulting in a myocardial oxygen supply–demand imbalance. The diagnosis is confirmed if ischaemia is a consequence of obstructive coronary artery disease without acute coronary pathology, or myocardial injury results in a new or presumed new regional wall motion abnormality or absence of viable myocardium (see Section 7.2.2).
- Procedure-related myocardial infarction is considered when acute myocardial ischaemia and injury occur due to a complication of any cardiac procedure (percutaneous or surgical) within 30 days. The diagnosis is confirmed if a coronary complication is identified ***or*** myocardial injury results in a new or presumed new regional wall motion abnormality or absence of viable myocardium. However, both a complication ***and*** a new or presumed new regional wall motion abnormality or absence of viable myocardium are required when the complication arises during the index procedure, or the procedure is performed in the management of acute myocardial infarction (see Section 7.2.3).
- The diagnosis of myocardial infarction following sudden death is discussed (see Section 8) and new, objective criteria are provided for silent or unrecognized myocardial infarction (see Section 9).
- The definition of MINOCA is updated to ‘myocardial injury with non-obstructive coronary arteries’ in recognition that this is a working rather than final diagnosis (see Section 11).
- Accelerated diagnostic pathways using high-sensitivity cardiac troponin assays are introduced and guidance is provided for the areas of practice in which the interpretation of cardiac troponin is challenging (see Section 14).
- New guidance is provided for the diagnosis of myocardial infarction in low-resource settings (see Section 19).
- International Classification of Disease 11th Revision (ICD-11) codes for use in hospital episode statistics and public health monitoring are proposed that align with the updated classification of myocardial infarction (see Section 18) to encourage implementation and further research (see Section 20).

## 5. Definition of myocardial infarction

Myocardial infarction is defined pathologically as myocardial cell death due to prolonged periods of ischaemia caused by insufficient or absent coronary blood flow and reperfusion injury (10,11). A histopathological diagnosis requires autopsy, but a clinical diagnosis of myocardial infarction can be confirmed through the joint evaluation of clinical features, biomarkers, electrocardiography, and coronary or cardiac imaging. A clinical diagnosis requires indirect evidence of irreversible cell death and confirmation that cell death was caused by myocardial ischaemia. No single criterion can confirm the diagnosis of acute myocardial infarction; therefore, several criteria are necessary that collectively increase the likelihood that myocardial infarction has occurred.

The clinical diagnosis of myocardial infarction requires evidence of acute myocardial injury (see Section 6) **and** one or more of the following: symptoms or other evidence of acute myocardial ischaemia that include new ischaemic changes or pathological Q waves on the electrocardiogram (ECG); imaging evidence of an acute coronary pathology; or new loss of viable myocardium or a new regional wall motion abnormality in a pattern consistent with an ischaemic aetiology (8).

These criteria are easier to apply when the clinical presentation is spontaneous acute onset of chest discomfort in the absence of other precipitating factors, and the initial evaluation should follow clinical practice guidelines (1,2). The diagnosis is often straightforward when the presentation is with central and radiating chest pain or discomfort (see Section 12) accompanied by ST-segment elevation on the ECG (see Section 13). However, many people do not have classical symptoms, and the ECG is often normal or not diagnostic of myocardial ischaemia (12). Thus, in a substantial proportion of cases, the working diagnosis of myocardial infarction is suggested by cardiac biomarker testing (see Section 14). Among those with acute myocardial injury but without ST-segment elevation, subsequent coronary or cardiac imaging identifies an alternative diagnosis in around 1 in 20 cases, excluding the diagnosis of myocardial infarction (13,14). The clinical diagnosis is even more challenging in settings in which myocardial injury is common—acute illness or following a cardiac procedure. In these settings, where the cause of myocardial injury can be ischaemic or non-ischaemic, imaging should be encouraged to confirm or refute the diagnosis of myocardial infarction (see Section 15).

## 6. Acute and chronic myocardial injury

The term *myocardial injury* was defined in the Third UDMI as an elevated cardiac troponin I or T level with at least one value above the 99th percentile upper reference limit (URL) regardless of the underlying cause (7). The Fourth UDMI added the terms *acute myocardial injury* and *chronic myocardial injury* (8). Both acute and chronic myocardial injury are common, but their prevalence varies depending on the approach to testing (15) and the assay (16), with chronic myocardial injury up to five times more frequent with cardiac troponin T than cardiac troponin I (17).

### 6.1. Acute myocardial injury

*Acute myocardial injury* is defined as a rise and/or fall in cardiac troponin with at least one value above the sex-specific 99th percentile URL (Box 1). Sex-specific thresholds are necessary to avoid a systematic bias and the under-recognition of myocardial injury in female patients (see Section 14.1.1).

**Box 1 Criteria for acute myocardial injury**

Acute myocardial injury is defined as a rise and/or fall in cardiac troponin I or T with at least one value above the sex-specific 99th percentile URL.

Acute myocardial injury can be caused by ischaemic and non-ischaemic mechanisms. When demonstrated to be due to ischaemia, acute myocardial injury is the hallmark of myocardial infarction, and it should be classified as such when the diagnostic criteria for myocardial infarction are met. However, many other cardiac and non-cardiac conditions are commonly associated with acute myocardial injury without myocardial ischaemia (18). Non-ischaemic mechanisms for acute myocardial injury include inflammation, physiological stress response, catecholamine stress, cardiotoxic agents, and trauma (Table 2).

**Table 2:** Mechanisms of acute myocardial injury.


MECHANISM	CONDITION

**Ischaemia**	Myocardial infarction

**Inflammation**	Myocarditis (autoimmune, infectious, toxic)

	Heart transplant rejection

	Cytokine mediated in sepsis

**Haemodynamic stress**	Supply–demand imbalance^a^

	Tachy- or brady-arrhythmia

	Acute heart failure

	Acute pulmonary embolism

	Malignant hypertension

**Physiological stress**	Strenuous exercise

**Catecholamine stress**	Takotsubo syndrome

	Subarachnoid haemorrhage

	Stroke

	Epileptic seizures

	Phaeochromocytoma

**Toxicity**	Anthracyclines

	ErbB (erythroblastic oncogene B)-targeted therapies

	Tyrosine kinase inhibitors

	Immune checkpoint inhibitors

	Radiation induced

**Trauma**	Electrical cardioversion

	Percutaneous coronary or structural intervention

	Catheter ablation or device implantation

	Cardiac surgery

	Cardiac contusion


^a^Any acute condition that results in a myocardial oxygen imbalance with reduced supply (hypoxia, anaemia, hypotension) and/or increased demand (tachycardia, hypertension).

In some cases, there may be multiple mechanisms responsible for acute myocardial injury, for example in sepsis, where both inflammation and the physiological stress response may contribute (19).

Whether acute myocardial injury results in transient or permanent effects on myocardial function varies depending on the severity of the injury and underlying condition. In several conditions associated with significant increases in cardiac troponin, e.g. Takotsubo syndrome, severe left ventricular dysfunction and regional wall motion abnormalities may resolve without evidence of permanent damage, suggesting the underlying mechanism is due to a functional abnormality rather than myocardial ischaemia or necrosis (20,21). In myocarditis, myocardial injury-related cardiac dysfunction may be fully reversible (22). Elevated cardiac troponin may not always reflect necrosis (23,24,25), as seen in athletes after strenuous exercise, where troponin release likely reflects reversible injury (26), though some irreversible damage cannot be excluded (27).

Acute myocardial injury requires clinical evaluation to determine its aetiology, assess functional impact, and identify ischaemia, in which case, coronary and cardiac imaging is required to confirm or exclude myocardial infarction.

### 6.2. Chronic myocardial injury

*Chronic myocardial injury* is a term used to describe persistently elevated cardiac troponin due to an underlying cardiac condition or a non-cardiac condition associated with cardiac remodelling (Box 2). Cardiac troponin can be elevated chronically due to three mechanisms: increased release from the myocardium due to a cardiac condition; reduced clearance from the circulation; or analytical interference that can mimic either (see Section 14).

**Box 2 Criteria for chronic myocardial injury**

Chronic myocardial injury is **considered** if two or more cardiac troponin I or T values are above the sex-specific 99th percentile URL when testing is performed in a stable clinical setting. Chronic myocardial injury is **confirmed** when a cardiac or non-cardiac condition associated with cardiac remodelling is identified and is excluded when the elevation in cardiac troponin is explained by analytical interference or reduced clearance.

At levels within the normal reference range cardiac troponin is cleared by the kidneys, whereas at higher concentrations elimination is thought to be predominantly by the liver (28,29). A halving of the estimated glomerular filtration rate (eGFR) will lead to a doubling of the basal level of cardiac troponin (28). Given that the majority of patients with chronic elevations in cardiac troponin only have minor increases above the 99th percentile, it is important to consider the effect of reduced renal function when interpreting chronically elevated cardiac troponin levels (see Section 16.3).

Atherosclerotic coronary artery disease (CAD) is a chronic, progressive lifelong condition (30) and chronic myocardial injury can arise as a consequence of low-grade recurrent myocardial ischaemia in patients with chronic coronary syndromes or ischaemic cardiomyopathy, or may arise due to haemodynamic stress in chronic congestive cardiac failure or severe hypertension (Table 3). It may also occur due to left ventricular hypertrophy and in other cardiomyopathies or structural conditions, such as valvular and congenital heart disease (31,32,33). In many of these conditions the presence of chronic myocardial injury is an adverse prognostic feature (34). However, persistently elevated cardiac troponin concentrations are also observed in older people without detectable heart disease. Furthermore, elevated cardiac troponin may not be due to myocardial injury at all but can arise due to *in vivo* or *ex vivo* antibody-mediated assay interference (see Section 14.1.2). The detection of a persistently elevated cardiac troponin concentration merits further investigation to determine whether this is due to chronic myocardial injury, in which case cardiac imaging may help to determine whether this is a consequence of structural or coronary heart disease.

**Table 3:** Mechanisms of chronic myocardial injury.


AETIOLOGY	CONDITION

**Ischaemia**	Chronic coronary syndrome

	Ischaemic cardiomyopathy

**Other cardiomyopathies**	Hypertrophic cardiomyopathy

	Hypertensive cardiomyopathy

	Dilated cardiomyopathy

	Chronic inflammatory cardiomyopathy

	Restrictive cardiomyopathy

	Infiltrative cardiomyopathy

	Uraemic cardiomyopathy in chronic kidney disease

**Haemodynamic stress**	Chronic heart failure

	Systemic hypertension

	Pulmonary hypertension

**Structural**	Valvular heart disease

	Congenital heart disease

	Cardiac tumour


In an acute setting, it can be difficult to determine whether elevated cardiac troponin is due to acute or chronic myocardial injury, especially if serial measurements are taken at short intervals (35). The priority in the acute setting is to exclude acute myocardial injury or infarction using a validated accelerated diagnostic pathway (see Section 14.1.5). In those patients where acute myocardial injury or infarction is excluded, troponin testing can be repeated in an outpatient setting or values compared with those from a previous assessment (17,36). Injury is more likely to be chronic and stable than progressive if the change is less than 20% between troponin measurements, but clinicians should be aware that this criterion is simply based on the analytical imprecision of cardiac troponin assays. If cardiac troponin levels remain elevated, then further investigation could be considered to confirm whether this is a result of chronic myocardial injury due to an underlying cardiac condition.

## 7. Classification of myocardial infarction

### 7.1. Approaches to the classification of myocardial infarction

Several pathophysiological mechanisms can lead to myocardial infarction. A logical classification of myocardial infarction should reflect the underlying pathophysiology, use objective diagnostic criteria, and be intuitive for consistent application and clear understanding by clinicians and patients.

#### 7.1.1. Classification based on aetiology

The Third UDMI introduced a classification that was based on the underlying cause, identifying five subtypes of myocardial infarction (7). This was expanded to seven subtypes in the fourth iteration (8). In recognition that cardiac troponin levels are elevated in many cardiac and non-cardiac conditions, two further diagnostic classifications were introduced: acute non-ischaemic myocardial injury and chronic myocardial injury, defined by whether a rise and/or fall in cardiac troponin levels was observed on serial testing (see Section 6).

The introduction of different types of myocardial infarction represented a major conceptual step forward (37), but has been challenging to apply consistently in practice (38,39). This is particularly true when myocardial infarction results from another acute illness or an acute coronary pathology other than atherothrombosis (previously type 2) or arises following coronary intervention (previously type 4a–c) or cardiac surgery (previously type 5).

Grouping non-atherosclerotic coronary causes (e.g. coronary dissection, embolism, or vasospasm) with myocardial oxygen supply–demand imbalance as type 2 myocardial infarction has limited its adoption in practice (40). These patients follow very different diagnostic pathways and require different treatments. Under the Fourth UDMI, type 2 myocardial infarction was diagnosed two ways: retrospectively, when patients initially presumed to have type 1 myocardial infarction underwent coronary angiography that identified no acute coronary pathology; and prospectively, when supply–demand imbalance from another acute condition was evident at presentation. As many of these patients were not managed by cardiologists, the diagnosis was frequently made based on symptoms or signs of myocardial ischaemia alone (41). Without coronary or cardiac imaging to confirm the cause or consequences of myocardial injury, a diagnosis of type 2 myocardial infarction had no direct treatment implications for many patients.

The previous classifications of type 4a and type 5 myocardial infarction following coronary intervention and cardiac surgery have been equally challenging to apply in practice as myocardial injury is ubiquitous in these settings and the classifications did not address complications following procedures other than revascularization. Thresholds that were 5 and 10 times greater than the 99th percentile URL of the cardiac troponin assay were arbitrary and applied along with additional criteria for the diagnosis of type 4a and type 5 myocardial infarction, respectively (8). However, for type 5 myocardial infarction, a large prospective international study showed that 97.5% of patients undergoing cardiac surgery had cardiac troponin levels more than 10 times the URL of the assay, and that thresholds more than 218 times the URL (95% confidence interval 40 to 318) were required to identify those at increased risk of peri-procedural mortality (42). Following coronary artery bypass graft (CABG) surgery several alternative criteria have been proposed (43) using cardiac troponin thresholds up to 170 times the URL or using less sensitive and specific biomarkers, such as creatine kinase-muscle/brain isoenzyme (CK-MB), which may no longer be widely available (44). Likewise, definitions using alternative thresholds led to markedly different frequencies of the diagnosis of procedural myocardial infarction (type 4a) following percutaneous coronary intervention (PCI) and also had different prognostic significance (45). Recent consensus statements have focused on the identification of prognostically relevant myocardial injury after coronary surgery and percutaneous coronary procedures (46,47). However, prognostic thresholds are not a substitute for clinical diagnosis and as the URLs between assays are not biologically equivalent the use of multiples of the URL to confirm the diagnosis is problematic. This variation in the diagnostic criteria has made it challenging to interpret myocardial infarction as an endpoint in clinical trials (48), and to advise patients on the risks and benefits of revascularization using either approach.

Finally, while a classification that used alpha-numerical terms has encouraged research, these terms did not directly convey information about the underlying mechanism of myocardial infarction. Therefore, they were of less value when communicating with patients and with many clinicians.

#### 7.1.2. Classification based on the electrocardiogram

The most widely adopted classification in practice stratifies myocardial infarction into two groups based on the presenting ECG, according to the presence or absence of ST-segment elevation (see Section 13). The recently released ICD-11 reflects the widespread use of this classification by introducing distinct codes for ST-segment elevation myocardial infarction (STEMI) (BA41.0) and non-ST-segment elevation myocardial infarction (NSTEMI) (BA41.1) (1,2). Regional ST-segment elevation generally reflects an acute coronary occlusion requiring immediate restoration of blood flow to reduce infarct size (49). However, not all patients with acute coronary occlusion demonstrate ST-segment elevation on the 12-lead ECG and other confounders (such as bundle branch block) can obscure interpretation of the ST segment (see Section 13) (50,51,52,53). While imperfect, this classification is simple and useful to identify those patients likely to benefit from immediate coronary intervention or fibrinolysis (49,54). The terms in isolation offer limited insight into the underlying pathophysiological mechanism of myocardial infarction, however, and are therefore less useful at guiding management beyond the acute presentation.

### 7.2. Clinical classification of myocardial infarction

Building on the existing clinical definition and classifications of myocardial infarction, while addressing some of the limitations that have prevented consistent application in practice, a simplified new clinical classification of myocardial infarction is proposed. It is aligned with the usual pathway for evaluation of patients with suspected myocardial infarction, considers the underlying pathophysiology, and incorporates more objective diagnostic criteria to enable more consistent application in practice, research, and comparative clinical trials.

This simplified clinical classification recognizes that myocardial infarction can arise in three settings: (i) spontaneously due to a primary acute coronary pathology (**primary myocardial infarction**); (ii) secondary to another acute condition resulting in myocardial oxygen supply–demand imbalance (**secondary myocardial infarction**); or (iii) as a complication following a cardiac procedure (**procedure-related myocardial infarction**). This revised classification aims to increase adoption and consistent application in practice (see Central illustration Figure 1).

#### 7.2.1. Primary myocardial infarction

Primary myocardial infarction is considered in a patient with spontaneous onset of symptoms or signs of acute myocardial ischaemia and acute myocardial injury where no alternative acute condition or cardiac procedure has triggered the presentation. While the majority are due to atherothrombosis, it includes all primary acute coronary pathologies. The diagnostic criteria for primary myocardial infarction prioritize sensitivity to minimize missing an acute coronary pathology and encourages coronary angiography to confirm the diagnosis and define the underlying mechanism (Box 3).

**Box 3 Diagnostic criteria for primary myocardial infarction**

The diagnosis is **likely** in patients with acute *myocardial injury* identified through the measurement of cardiac troponin when one or more clinical or electrocardiographic features are present:

- Symptoms consistent with acute myocardial ischaemia
- New or presumed new ischaemic changes on the electrocardiogram
- Development of pathological Q waves.

The diagnosis is **confirmed** with one or more additional features on imaging:

- Identification of an acute coronary pathology comprising atherothrombosis, spontaneous coronary artery dissection, vasospasm, or embolism; or restenosis, stent thrombosis, or bypass graft failure more than 30 days from revascularization
- Development of a new or presumed new regional wall motion abnormality and/or absence of viable myocardium in a pattern consistent with an ischaemic aetiology.

**Figure 2:**
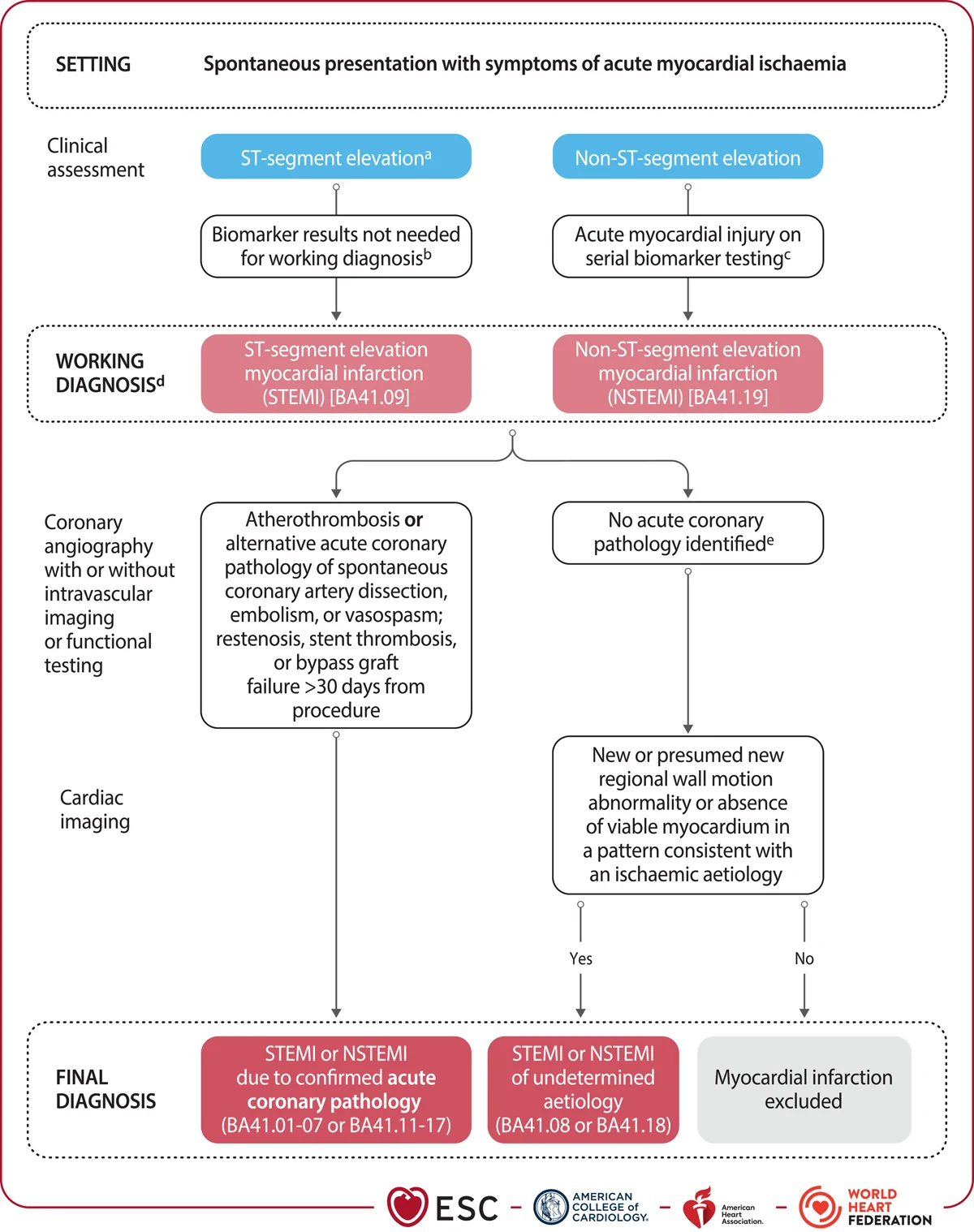
Diagnostic pathway for possible primary myocardial infarction. NSTEMI, non-ST-segment elevation myocardial infarction; STEMI, ST-segment elevation myocardial infarction. ^a^Or other electrocardiographic changes indicative of possible acute coronary occlusion (Figure 9). ^b^For patients with a working diagnosis of STEMI, acute myocardial injury is necessary to confirm the final diagnosis. ^c^Rise and/or fall of cardiac troponin I or T values and with at least one value above the sex-specific 99th percentile upper reference limit on serial testing. ^d^Where coronary angiography and cardiac imaging are not available or are not considered appropriate, a final diagnosis of primary myocardial infarction can be assigned based on an evaluation that considers biomarkers, electrocardiography, and clinical criteria alone without additional confirmatory features and the codes BA41.09 or BA41.19 applied as the aetiology is unknown. ^e^This includes patients with a working diagnosis of myocardial injury with non-obstructive coronary arteries (MINOCA).

**Figure 3:**
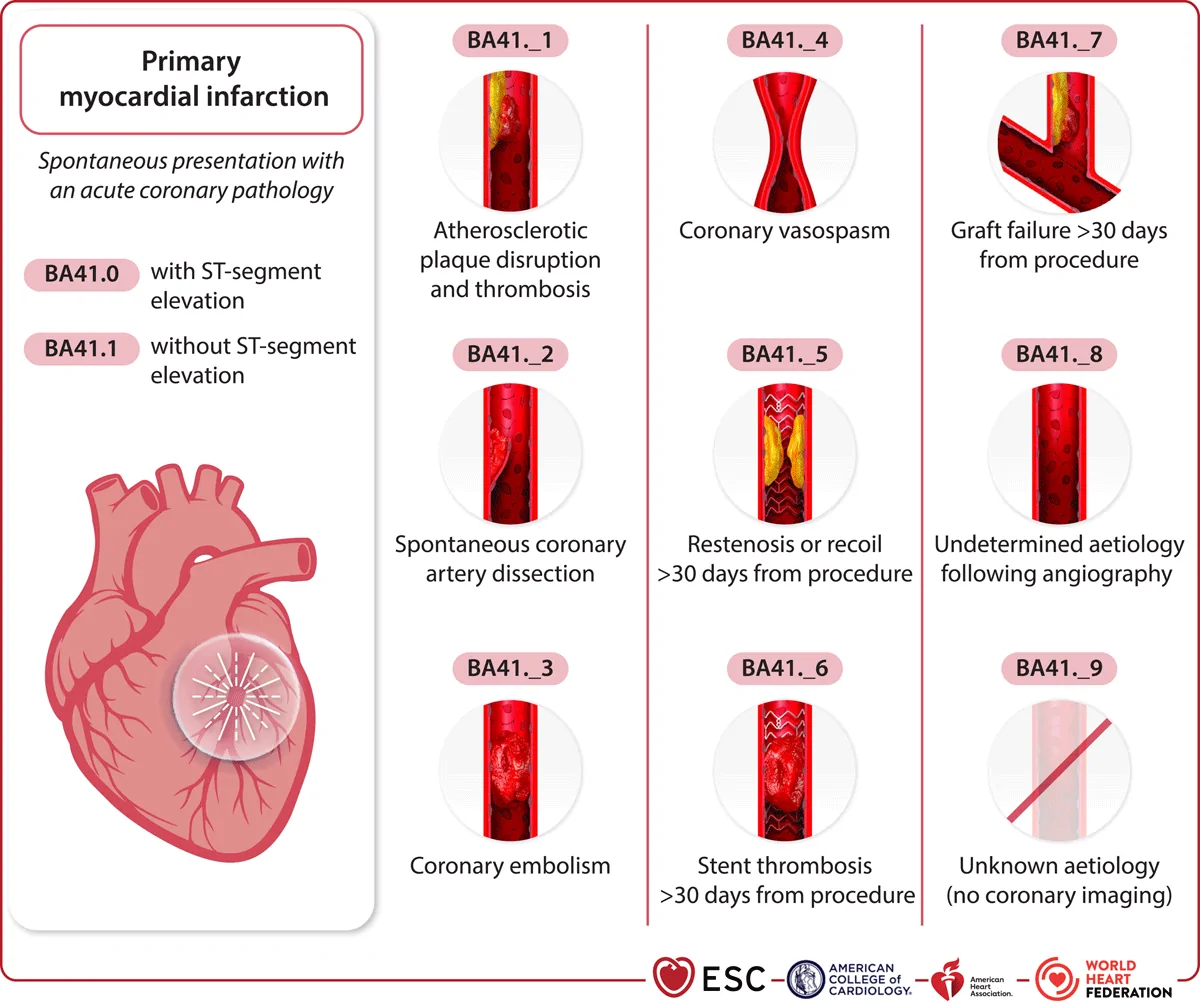
Primary myocardial infarction due to acute coronary pathology.

The diagnostic pathway for patients with possible primary myocardial infarction is illustrated in Figure 2. The diagnosis is likely in those with acute myocardial injury presenting with the spontaneous onset of symptoms suggestive of myocardial ischaemia (see Section 12) or changes on the ECG consistent with acute myocardial ischaemia (see Section 13). When more clinical features are present, the likelihood of myocardial infarction is greater. Coronary angiography and/or cardiac imaging are required to confirm the diagnosis, and invasive assessment with angiography or intracoronary imaging is essential to identify the underlying acute coronary pathology for further classification (Figure 3). However, where coronary angiography and cardiac imaging are not available or are not considered appropriate, a final diagnosis of primary myocardial infarction can be made based on an evaluation that considers biomarkers, electrocardiography, and clinical criteria alone.

Although the term primary myocardial infarction is used in this classification to differentiate from secondary and procedure-related myocardial infarction, it is synonymous with spontaneous myocardial infarction. In practice, when this diagnosis is made, clinicians may simply refer to the diagnosis as myocardial infarction.

In primary myocardial infarction an acute coronary pathology can cause a total or sub-total coronary artery occlusion, presenting with or without ST-segment elevation. Atherothrombosis (thrombus associated with plaque rupture or erosion) is the most common acute coronary pathology in primary myocardial infarction (55,56,57). In addition to causing acute coronary occlusion, thrombus formation can also cause distal embolization (58). The degree of atherosclerosis and thrombus in the culprit lesion varies greatly and non-flow-limiting stenoses are also common causes of myocardial infarction (59). Other less common but equally important acute coronary pathologies in primary myocardial infarction, with or without ST-segment elevation, include spontaneous coronary artery dissection (SCAD), epicardial or microcirculatory vasospasm (hence forth ‘vasospasm’), or embolism from a non-coronary source; as well as restenosis, stent thrombosis, or graft failure arising more than 30 days from a revascularization procedure (see Section 7.2.3 for events within 30 days). SCAD should be considered particularly in female patients under 50 years of age and in pregnancy or the post-partum period (60,61).

Coronary angiography does not always identify the underlying acute coronary pathology that is the cause of the primary myocardial infarction. Here, adjunctive intravascular imaging, such as optical coherence tomography (OCT) and intravascular ultrasound (IVUS) may be helpful (see Section 15). In some, both coronary and cardiac imaging are necessary to confirm or exclude the diagnosis of primary myocardial infarction. This is particularly relevant in patients with non-obstructive coronary arteries (see Section 11). In these patients, cardiac magnetic resonance (CMR) imaging is the modality of choice to confirm the diagnosis of myocardial infarction (1,62), but more often identifies an alternative cause of acute myocardial injury such as myocarditis or Takotsubo syndrome (63).

When coronary angiography is not performed, the diagnosis of STEMI or NSTEMI can be applied with primary myocardial infarction of unknown aetiology (Table 4). However, when coronary angiography is performed, and the underlying acute coronary pathology identified, the final diagnosis should reflect this. This information will provide important additional insight into future risk of coronary events and treatment.

**Table 4:** ICD-11 codes for the classification of myocardial infarction.


FINAL DIAGNOSIS	ICD-11	SIXTH DIGIT STEM CODE/EXTENSION CODE

Acute ST-segment elevation myocardial infarction (STEMI)^a^	BA41.0	**Primary myocardial infarction** BA41._1 atherothrombosis BA41._2 spontaneous coronary artery dissection BA41._3 coronary embolism BA41._4 coronary vasospasm BA41._5 restenosis >30 days from procedure BA41._6 stent thrombosis >30 days from procedure BA41._7 graft failure >30 days from procedure BA41._8 undetermined aetiology BA41._9 unknown aetiology (no coronary imaging) **Secondary myocardial infarction** BA41._A/ICD-11 code for alternative acute condition **Procedure-related myocardial infarction** BA41._B due to a complication of any percutaneous cardiac procedure within 30 days BA41._C due to a complication of any open cardiac surgical procedure within 30 days

Acute non-ST-segment elevation myocardial infarction (NSTEMI)	BA41.1	

**Other diagnoses**		

Unstable angina	BA40	

Unspecified myocardial infarction	BA41.Z	Sudden death without evaluation^b^

Unrecognized myocardial infarction	BA50	


ICD-11, 11th edition of the International Classification of Diseases. ^a^Including electrocardiographic findings of acute coronary occlusion (Figure 9). ^b^See Figure 8.

#### 7.2.2. Secondary myocardial infarction

The diagnosis of secondary myocardial infarction is considered in patients with acute myocardial injury secondary to an alternative acute condition, causing a mismatch between myocardial oxygen supply and demand when this results in symptoms or signs of myocardial ischaemia (64,65,66). The diagnosis of secondary myocardial infarction is confirmed if the myocardial ischaemia resulting from supply–demand mismatch is a *consequence* of obstructive CAD without evidence of an acute coronary pathology, or the resulting myocardial injury is sufficient to cause a new regional wall motion abnormality or loss of myocardial viability. The diagnostic criteria prioritize specificity and encourage coronary angiography and cardiac imaging to reduce uncertainty, increasing the likelihood that the diagnosis has treatment implications for the patient (Box 4). If imaging provided evidence of an acute coronary pathology (Figure 3) then a diagnosis of primary myocardial infarction would be confirmed.

**Box 4 Diagnostic criteria for secondary myocardial infarction**

The diagnosis is **considered** in patients with acute myocardial injury identified through the measurement of cardiac troponin who present with an *alternative acute condition resulting in myocardial oxygen supply–demand mismatch*, when one or more clinical or electrocardiographic features are present:

- Symptoms consistent with acute myocardial ischaemia
- New or presumed new ischaemic changes on the electrocardiogram
- Development of pathological Q waves.

The diagnosis is **likely** with one or more additional features:

- Known coronary artery disease
- A strong clinical suspicion due to the extent of myocardial ischaemia on the electrocardiogram or cardiac troponin elevation.

For patients in whom coronary and/or cardiac imaging is feasible and appropriate, the diagnosis is **confirmed** if one or more of the following features are present:

- Obstructive coronary artery disease, defined as ≥70% stenosis in an epicardial vessel by angiography (or ≥50% stenosis in an epicardial vessel that is flow-limiting on physiological assessment) **without** an acute coronary pathology
- Development of a new or presumed new regional wall motion abnormality or absence of viable myocardium in a pattern consistent with an ischaemic aetiology.

**Figure 4:**
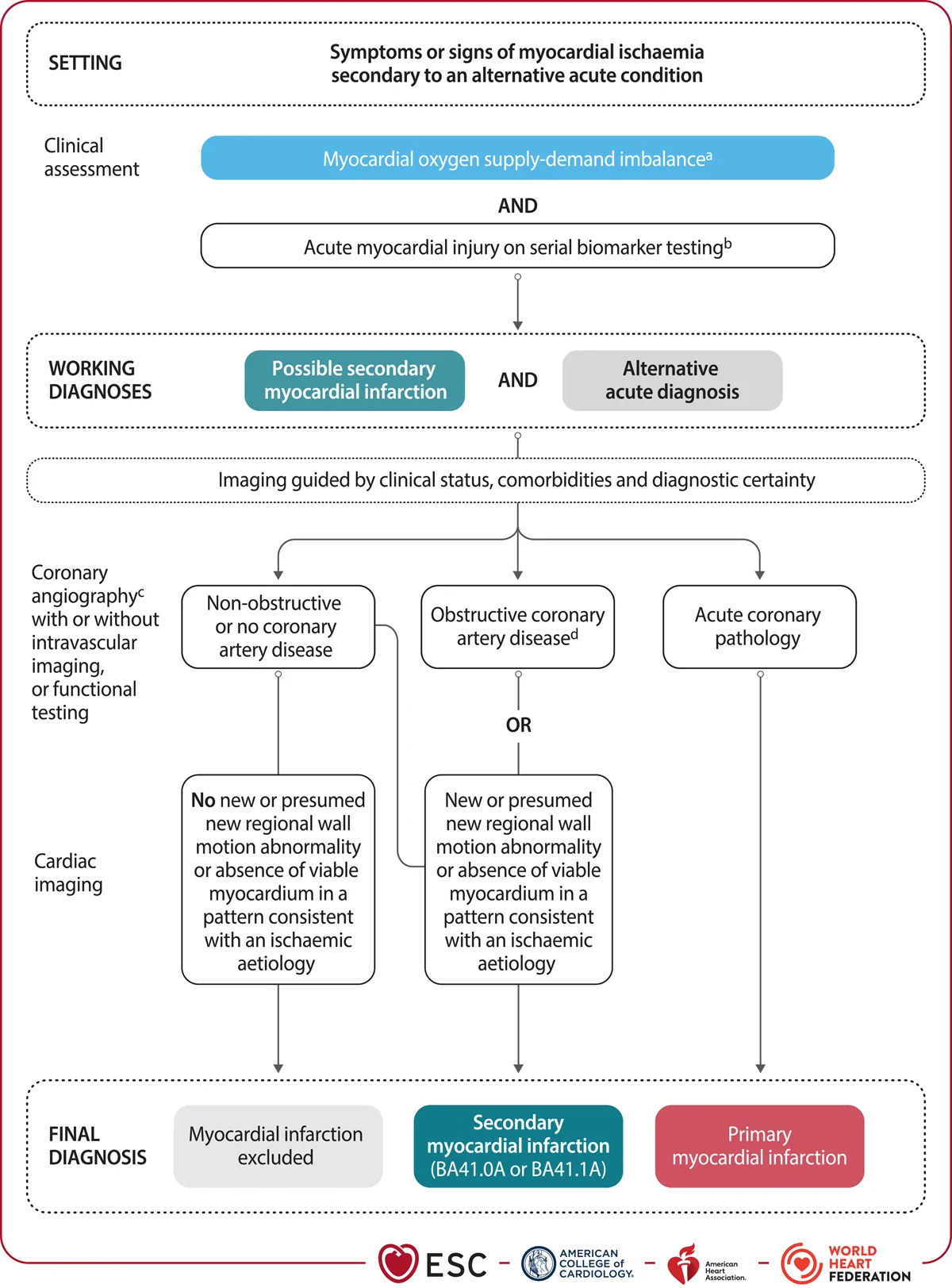
Diagnostic pathway for possible secondary myocardial infarction. ^a^Myocardial oxygen supply–demand imbalance can occur as a result of increased demand (e.g. tachycardia or severe hypertension) and/or reduced supply (e.g. hypotension, hypoxia, or anaemia). ^b^Rise and/or fall of cardiac troponin values and with at least one value above the sex-specific 99th percentile upper reference limit on serial testing. ^c^If primary myocardial infarction is an unlikely differential diagnosis, then non-invasive computed tomography (CT) coronary angiography following recovery from acute illness may be considered. ^d^Obstructive coronary artery disease is defined as ≥70% stenosis in an epicardial vessel by angiography or ≥50% stenosis that is flow-limiting on physiological assessment.

**Figure 5:**
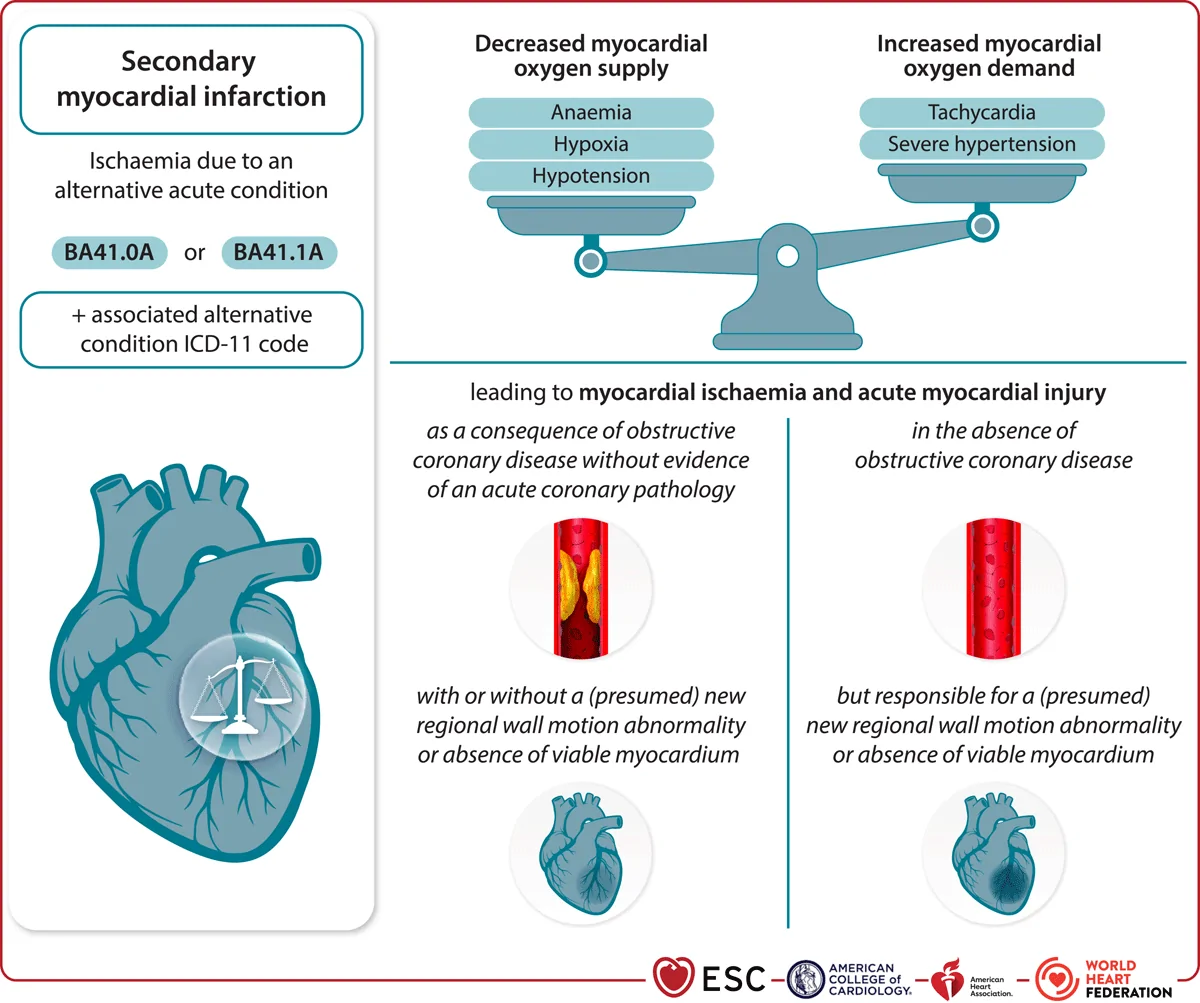
Secondary myocardial infarction due to supply–demand imbalance.

The pathway for patients in whom the diagnosis of secondary myocardial infarction is being considered is illustrated in Figure 4. Myocardial oxygen supply–demand imbalance can occur as a result of increased demand (e.g. tachycardia or severe hypertension) or reduced supply (e.g. hypotension, hypoxia, or anaemia) (Figure 5). Therefore, acute myocardial ischaemia can arise secondary to oxygen supply–demand imbalance in many acute conditions, the most common being tachyarrhythmia (41,67). The duration and severity of the trigger for supply–demand imbalance and whether the individual has underlying obstructive CAD or structural cardiac disease will influence the extent and severity of ischaemia (64). Furthermore, two or more triggers often coexist (68). Therefore, it is not possible to define thresholds for any of the triggers of supply–demand imbalance that could be reliably applied to all.

In this setting, symptoms of myocardial ischaemia are challenging to interpret as patients are less likely to report chest discomfort and are more likely to have other symptoms due to the underlying acute condition (69,70). While myocardial ischaemia secondary to supply–demand imbalance can manifest as regional ST-segment depression or T-wave inversion, these changes are often global. Acute myocardial injury is common in many conditions that result in supply–demand imbalance, but current cardiac troponin assays cannot differentiate ischaemic from non-ischaemic causes (71). Given that the diagnosis of myocardial infarction in this setting cannot be reliably established using symptoms or signs of myocardial ischaemia and cardiac troponin levels alone (72,73), additional clinical assessment and cardiac imaging are needed, when it is feasible and appropriate based on the clinical status, to confirm or exclude the diagnosis of secondary myocardial infarction. Most patients in this setting will not have obstructive CAD or imaging evidence of new loss of viable myocardium, and the diagnosis of secondary myocardial infarction can be excluded (74,75). It is important to note that acute illness, particularly sepsis or bleeding, can also trigger atherothrombosis; therefore, primary myocardial infarction is an important differential diagnosis. If myocardial infarction is excluded, the underlying aetiology of acute myocardial injury should be determined when possible (Table 2).

If there is persistent or recurrent myocardial ischaemia and primary myocardial infarction is considered as a differential diagnosis, invasive coronary angiography is indicated (1,2). Otherwise, imaging is often deferred until after the acute condition has been treated and non-invasive approaches can be applied, such as the use of coronary computed tomography angiography (CCTA) (75). For patients with comorbidities, advanced frailty, or limited life expectancy due to the underlying acute condition, additional cardiac imaging is sometimes not appropriate. Hence, it may not be possible to definitively confirm the diagnosis of secondary myocardial infarction and clinical judgment is required. Secondary myocardial infarction is more likely when patients have known CAD or significant myocardial injury that affects cardiac function (74). Those with obstructive coronary disease are also more likely to have myocardial ischaemia on the ECG (72). These features can support a clinical diagnosis when imaging is not feasible or appropriate.

One study suggests that application of these diagnostic criteria for secondary myocardial infarction would reduce the frequency of the diagnosis of myocardial infarction in acute illness and identify those patients at highest risk (74). A diagnosis of secondary myocardial infarction confirmed by coronary and/or cardiac imaging would have treatment implications for those with CAD (3) and/or left ventricular systolic dysfunction (1,2). The application of objective criteria to confirm the diagnosis of secondary myocardial infarction should encourage cardiac imaging in this setting. Imaging may also have additional benefits through the identification of non-obstructive but clinically relevant CAD or other previously unrecognized cardiac conditions, such as non-ischaemic cardiomyopathy or valvular heart disease (72).

#### 7.2.3. Procedure-related myocardial infarction

The diagnosis of procedure-related myocardial infarction is made when myocardial infarction arises as a complication of a cardiac procedure within 30 days of the procedure (Box 5). While 30 days is an arbitrary period, this is aligned with most studies of post-procedure complications (76). A cardiac procedure is defined as coronary angiography with or without intervention, structural cardiac intervention, or catheter ablation, and any open or minimally invasive cardiac surgical procedure, including CABG, valve replacement, or other procedures for structural heart conditions. The risk of procedure-related myocardial infarction is higher following PCI or CABG but is recognized following many other cardiac procedures. The new diagnostic criteria for procedure-related myocardial infarction are the same whether the complication arises from a percutaneous interventional or open surgical approach.

**Box 5 Diagnostic criteria for procedure-related myocardial infarction**

The diagnosis is **considered** in patients with a suspected coronary complication arising within 30 days of a cardiac procedure and acute *myocardial injury* identified through the measurement of cardiac troponin when one or more clinical or electrocardiographic features are present:

- Symptoms consistent with acute myocardial ischaemia
- New or presumed new ischaemic changes on the electrocardiogram
- Development of pathological Q waves
- Unexplained sudden clinical deterioration if under sedation.

Immediately following a procedure, the diagnosis is **likely** with higher cardiac biomarker levels that have increased from pre-procedural levels and continue to rise at 6 h (e.g. >5 times URL for intervention) and 24 h (e.g. >35 times URL for surgery).

The diagnosis is **confirmed** with one or more additional features, except when the complication arises during the procedure or the procedure is performed in the setting of an acute myocardial infarction, when both are required:

- Angiographic evidence of a coronary complication of percutaneous intervention (e.g. side branch occlusion, iatrogenic dissection, no or slow flow, recoil or early restenosis, stent thrombosis), or surgery (e.g. bypass graft failure, impingement of a coronary artery), or either (e.g. embolism, occlusion of coronary ostium during valve intervention)
- Cardiac imaging showing a new or presumed new regional wall motion abnormality or absence of viable myocardium in a pattern consistent with an ischaemic aetiology within the territory subtended by the coronary artery in which the complication arose.

**Figure 6:**
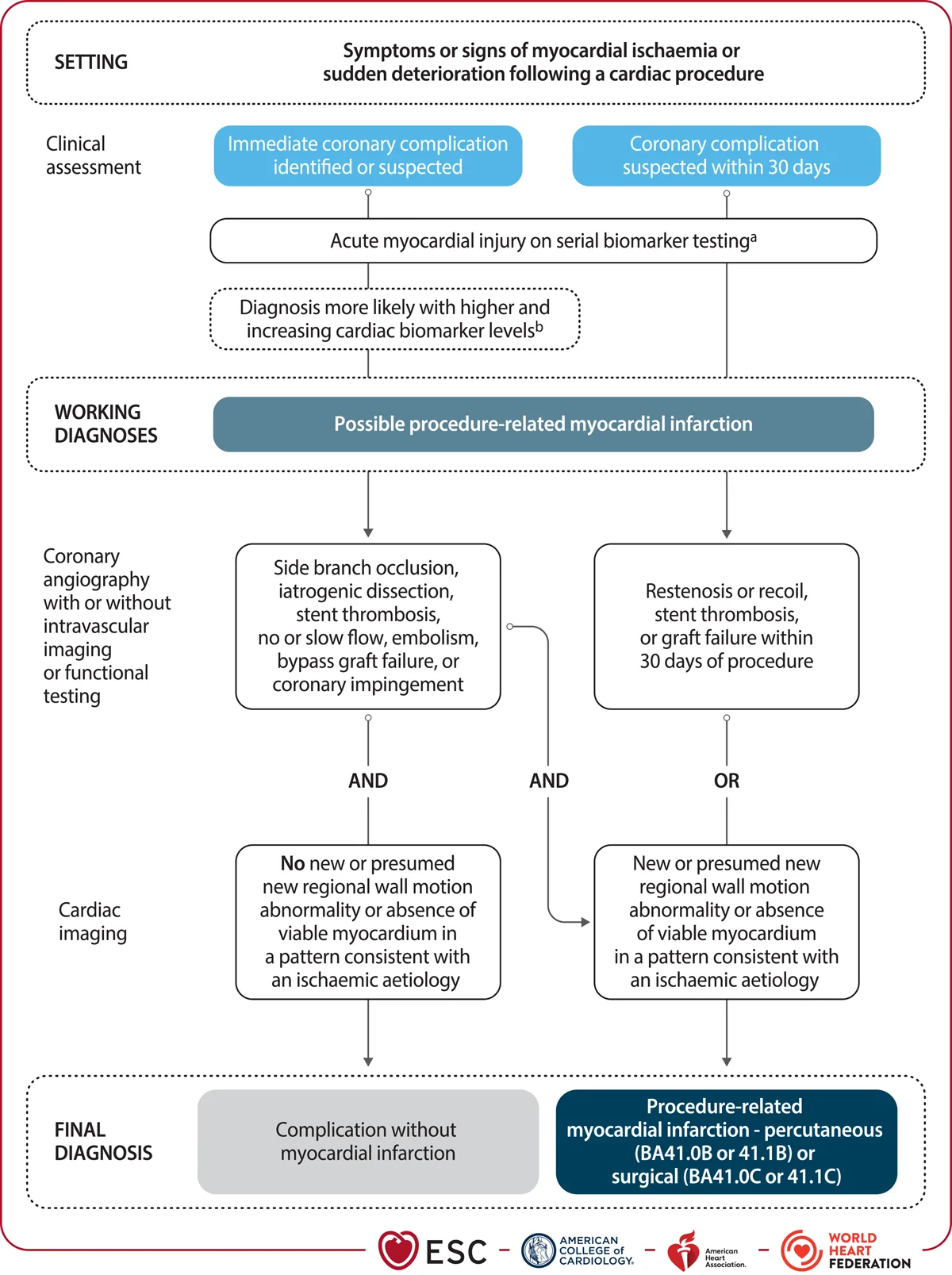
Diagnostic pathway for possible procedure-related myocardial infarction. ^a^Rising cardiac troponin values with at least one value above the sex-specific 99th percentile AND pre-procedural value (if known) on serial testing. ^b^Immediately following a procedure, the diagnosis is more likely with higher cardiac biomarker levels that have increased from pre-procedural levels and continue to rise at 6 h (e.g. >5 times upper reference limit [URL] for intervention) and 24 h (e.g. >35 times URL for surgery).

The pathway for patients in whom the diagnosis of procedure-related myocardial infarction is being considered is illustrated in Figure 6. Symptoms can be difficult to interpret immediately after cardiac procedures. Many patients experience some chest discomfort (77), but if the symptoms are persistent, increase in severity, or develop suddenly after the patient being symptom-free at the end of the procedure, they are more likely due to a complication. Adding to the challenge, patients often receive sedation for cardiac procedures and receive general anaesthesia for cardiac surgery. Although it may be impossible to determine whether chest discomfort is present, a sudden deterioration in clinical condition (e.g. pulmonary oedema, unexpected hypotension, tachycardia, or ventricular arrhythmia) may be a sign of acute myocardial ischaemia.

Electrocardiographic changes following coronary intervention are helpful, but ST-segment deviation and T-wave inversion are common after cardiac surgery due to other conditions (e.g. pericarditis, pneumopericardium, and electrolyte disturbance) (78,79). Furthermore, what may appear as pathological Q waves can be transient early after CABG (80). Thus, the presence of electrocardiographic changes requires further evaluation, but is not sufficient to diagnose procedure-related myocardial infarction.

Routine cardiac troponin measurement before and after coronary intervention or cardiac surgery is not supported by some clinical practice guidelines (3,81) or the European Association for Cardio-Thoracic Surgery (47), but is encouraged by the European Association of Percutaneous Cardiovascular Interventions (46); practice varies across the world. When cardiac troponin is measured, some degree of acute myocardial injury is ubiquitous (82,83,84). The extent of injury differs markedly depending on the duration and complexity of the procedure (42,85). For these reasons, it is not possible to use a single diagnostic threshold for cardiac troponin in this setting and cardiac imaging is required to confirm or exclude the diagnosis of procedure-related myocardial infarction.

Cardiac troponin testing is, however, essential in patients with clinical features of a suspected or evident coronary complication immediately following a procedure to guide the use of cardiac imaging. In this setting cardiac troponin thresholds may be helpful (and are recommended in some consensus statements) (8,86) to identify those in whom cardiac imaging is needed to confirm or exclude the diagnosis. Whilst thresholds of >5 times the URL following coronary intervention and >35 times the URL following cardiac surgery are arbitrary, patients with cardiac troponin concentrations below these thresholds may be less likely to have a new or presumed new regional abnormality due to a procedure-related complication. Further research is required to validate this assumption (see Section 20). The sensitivity and negative predictive value of these thresholds to identify patients in whom cardiac imaging is not required to exclude procedure-related myocardial infarction is unknown. Different thresholds may be required for cardiac troponin I and T, for males and females, and for complex procedures, such as percutaneous intervention for a chronic total occlusion or combined valve and coronary surgery. If pre-procedural cardiac troponin levels are available, comparison with post-procedural levels is informative (87). The timing of measurement is also important and can help differentiate transient myocardial injury due to instrumentation from injury due to myocardial ischaemia and a procedural complication (88,89,90). For example, after cardiac surgery, increasing cardiac troponin concentrations between 6 and 24 h are more likely in those with new or presumed new absence of viable myocardium on CMR imaging (88).

Immediately following a procedure, or when the procedure is performed in the setting of acute myocardial infarction, both cardiac and coronary imaging are required to confirm the diagnosis of procedure-related myocardial infarction. The diagnosis is confirmed if angiographic evidence of a coronary complication is identified **AND** there is a new regional wall motion abnormality or loss of viable myocardium in a pattern consistent with an ischaemic aetiology on cardiac imaging. When previous imaging is available, comparison with post-procedural imaging can help to differentiate new abnormalities. Because some complications arising during a procedure are transient and can be addressed promptly, the requirement for both features increases the specificity of the diagnosis of procedure-related myocardial infarction and ensures it is clinically meaningful. Examples of coronary complications that may arise during percutaneous intervention and cardiac surgery are given in Box 5.

**Figure 7:**
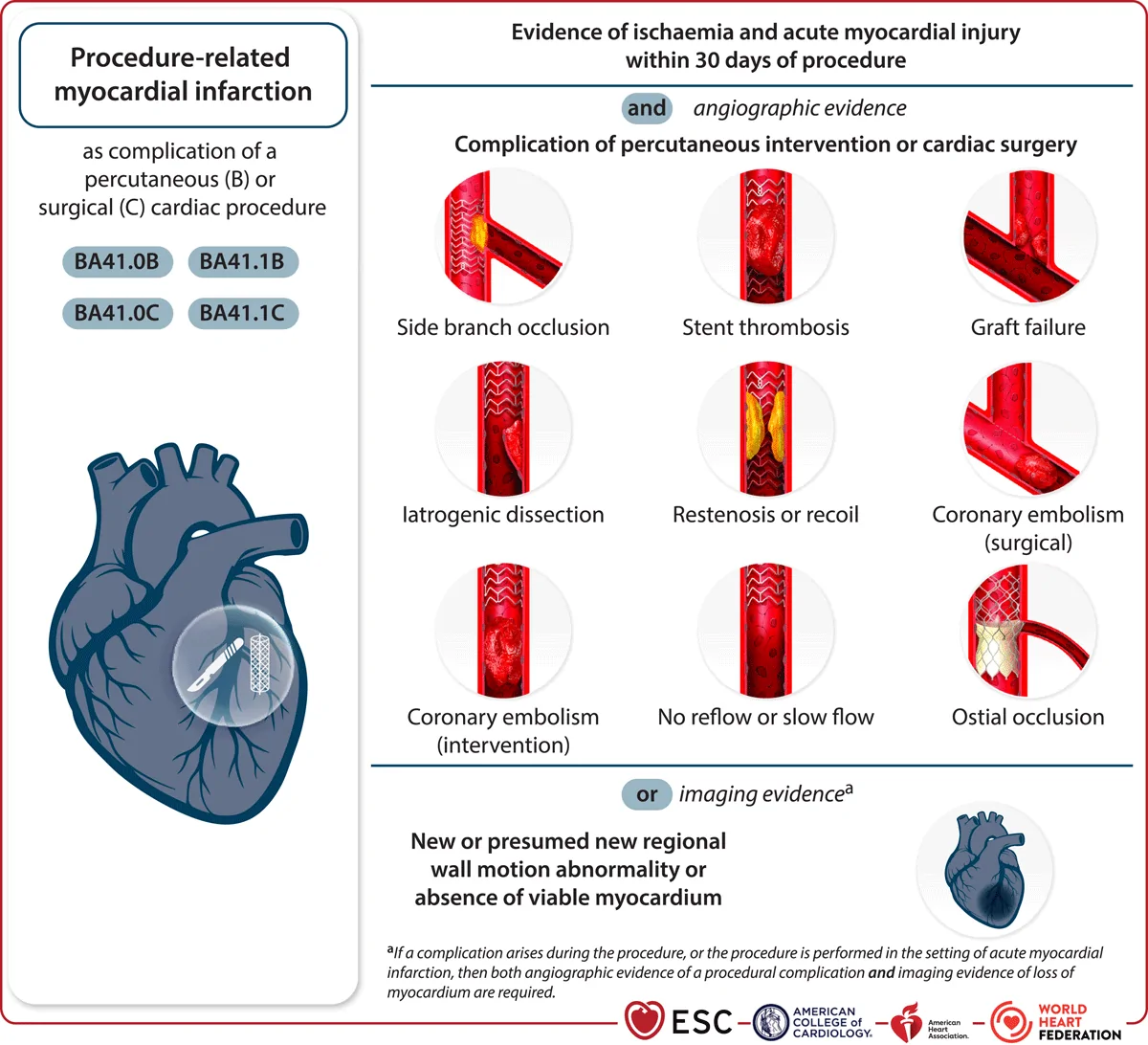
Procedure-related myocardial infarction due to a complication of cardiac surgery or intervention.

When new symptoms or signs of myocardial ischaemia and acute myocardial injury occur subsequent to an initially uncomplicated cardiac procedure and within 30 days, the diagnosis of procedure-related myocardial infarction is confirmed if either early restenosis or recoil, stent thrombosis, or graft failure **OR** a new regional wall motion abnormality or loss of viable myocardium is identified. To be considered a procedure-related myocardial infarction after an initially uncomplicated cardiac procedure, the acute coronary pathology needs to have arisen in the same vessel or territory treated in the index procedure. The criteria for, and causes of, procedure-related myocardial infarction are shown in Figure 7.

Patients may also have a myocardial infarction unrelated to the index procedure during this 30-day period, in which case the diagnosis of primary or secondary myocardial infarction would be considered depending on the setting (see Section 7.2.1 and Section 7.2.2). When an acute coronary event related to a prior cardiac procedure occurs beyond 30 days, the diagnosis of primary myocardial infarction should be considered.

## 8. Diagnosis of myocardial infarction following sudden death

Patients with symptoms or signs of myocardial ischaemia may die suddenly before clinical evaluation or investigations can be performed to confirm a diagnosis of myocardial infarction. An accurate diagnosis in this setting is important as it can carry implications for family members and insurance, death certification, and as an endpoint for clinical trials.

**Figure 8:**
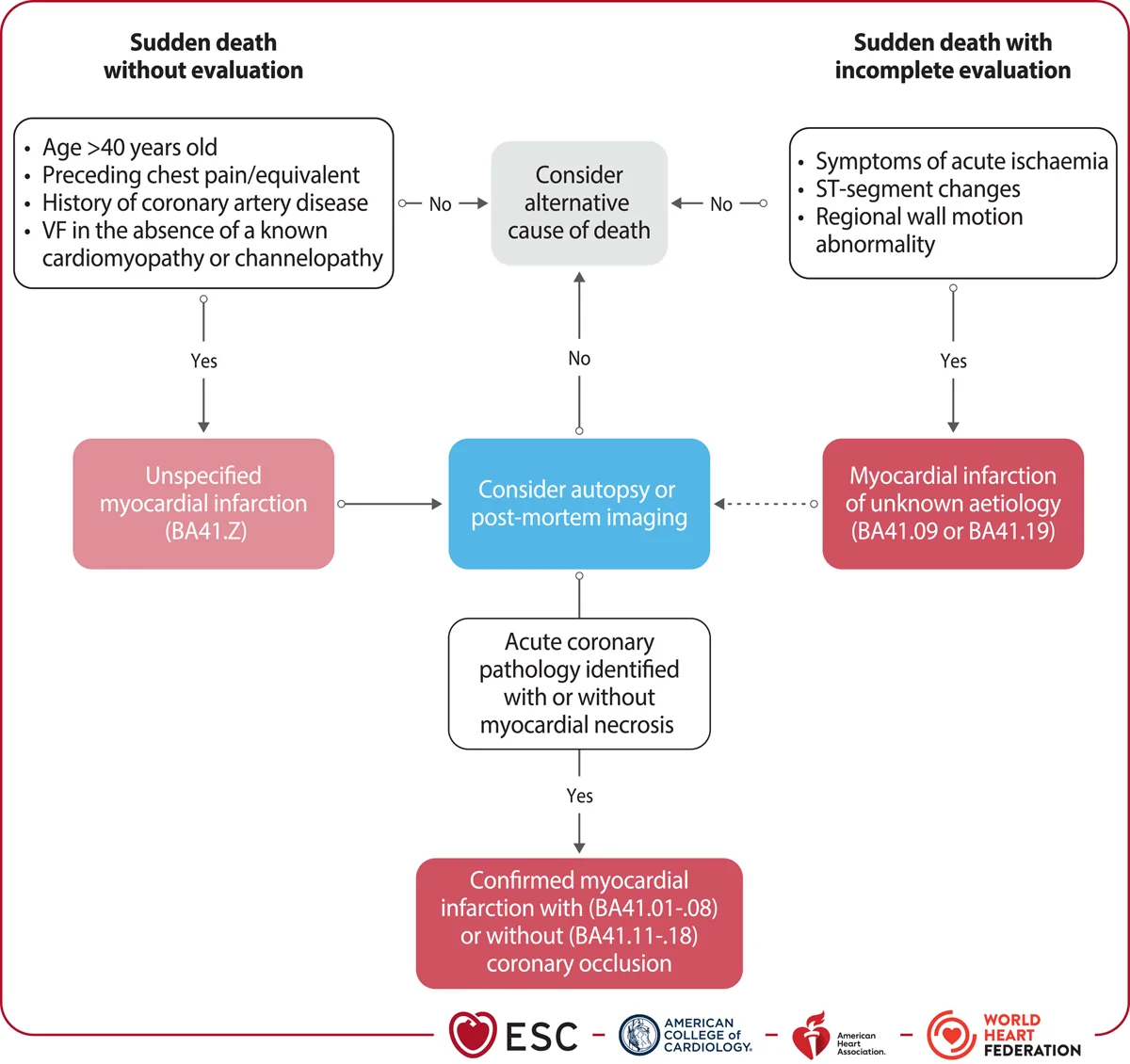
Diagnostic pathway for possible primary myocardial infarction following sudden death. VF, ventricular fibrillation. Following a sudden death in the community without clinical evaluation, the diagnosis of primary myocardial infarction can be considered. Clinical features such as a history of coronary artery disease, chest pain prior to death, or documented VF in the absence of known cardiomyopathy or channelopathies make the diagnosis more likely, but in the absence of an electrocardiogram or post-mortem investigations the diagnosis is uncertain, and the ICD-11 code of unspecified myocardial infarction (BA41.Z) should be applied to recognize this. Where investigations are incomplete with no biomarker testing or coronary imaging, the diagnosis of myocardial infarction of uncertain aetiology is applied, but where there is uncertainty post-mortem imaging or an autopsy is required to confirm the diagnosis and identify the underlying acute coronary pathology.

In cases of sudden death, clinical history and corroborative findings can be helpful and autopsy examinations are encouraged (Figure 8). Following a sudden death without any evaluation, the diagnosis of primary myocardial infarction is often considered as a possible cause of death. Clinical features such as a history of CAD, chest pain prior to death, or documented ventricular fibrillation in the absence of a known cardiomyopathy or channelopathy make the diagnosis more likely, but in the absence of an ECG or post-mortem investigation, the diagnosis remains uncertain.

When there is uncertainty, post-mortem imaging or an autopsy can confirm the diagnosis and identify the underlying aetiology for accurate classification into primary, secondary, or procedure-related myocardial infarction. Unfortunately, autopsies are performed in a minority of patients presumed to have died from myocardial infarction (91). Autopsy may exclude myocardial infarction by identifying non-cardiac causes of death or non-coronary causes of sudden cardiac death such as cardiomyopathy, including hypertrophic or arrhythmogenic cardiomyopathy, or cardiac sarcoidosis, while channelopathies are often associated with a structurally normal heart. Autopsy identification of coronary atherothrombosis, embolism, SCAD, or restenosis/thrombosis within a stent or bypass graft when it occurs more than 30 days from the index procedure, together with myocardial necrosis in the corresponding coronary artery region, confirms the diagnosis of primary myocardial infarction. However, it should be noted that histopathological features of myocardial necrosis are time dependent (92). Identification of myocardial necrosis and infarction in the absence of an acute coronary pathology, when there was clinical or autopsy evidence of an alternative acute condition that could increase myocardial oxygen demand or reduce supply, would support a diagnosis of secondary myocardial infarction. When the death arises within 30 days of a cardiac procedure with evidence of an acute coronary or graft occlusion on autopsy, the diagnosis of procedure-related myocardial infarction can be confirmed.

Contemporary alternatives to autopsy, including post-mortem computed tomography (CT) with or without coronary angiography or CMR imaging with biopsy, can inform the diagnosis of myocardial infarction (92,93). However, these approaches may not be widely available in practice. CT can identify obstructive CAD and prior myocardial infarction or suggest an alternative diagnosis, but the spatial resolution of conventional CCTA cannot reliably detect coronary plaque rupture or thrombus, or necrotic myocardium (93,94). Emerging data suggest that more contemporary CT technologies, including photon-counting CT, may have the ability to detect plaque characteristics indicative of acute rupture (95,96). Post-mortem CMR imaging has shown good correlation with histological specimens in identifying patients with myocardial infarction although its sensitivity is limited (97). Although these approaches are not routinely available in clinical practice and imaging protocols have not been standardized, the combination of CT-guided biopsy and CMR imaging has a sensitivity and specificity of >95% for the post-mortem diagnosis of acute myocardial infarction (92).

## 9. Silent or unrecognized myocardial infarction

Individuals with no known history of myocardial infarction may have pathological Q waves identified on a routine ECG. The specificity of pathological Q waves is limited (98); therefore, when available, the diagnosis of unrecognized (also termed clinically ‘silent’ or ‘old’) myocardial infarction should be confirmed by imaging, preferably with CMR with late gadolinium enhancement (see Section 15) (99,100). In some cases, regional wall motion abnormalities are detected on echocardiography, or myocardial infarction is observed on CMR imaging when these investigations have been performed for another indication and also may represent a silent or unrecognized myocardial infarction (101,102).

Although some cases may be truly ‘silent’, with no symptoms or signs even on careful consideration by the patient, the term ‘silent’ myocardial infarction can in some cases be misleading. Many patients in retrospect recall a history of symptoms but may not have considered them serious enough to seek acute care or recognize them as symptoms of a myocardial infarction, or they did seek assessment, but the diagnosis of myocardial infarction was not made. Unrecognized myocardial infarction is present in approximately 1 in 5 persons over the age of 70 years in the general population if CMR imaging is performed systematically, although most are small infarcts (103). Furthermore, in a recent study, 3% of asymptomatic middle-aged individuals (mean age 50 years) had CMR imaging evidence of unrecognized myocardial infarction (104). Among patients with chronic coronary syndromes, the frequency of unrecognized myocardial infarction is even higher and is associated with CAD in the infarct territory (105). Unrecognized myocardial infarction is relevant because it is associated with adverse prognosis (102,103,106). Furthermore, the identification of an unrecognized myocardial infarction may have important implications for medical treatment, particularly in patients without prior known CAD.

## 10. Unstable angina

In patients with new symptoms of myocardial ischaemia at rest or on minimal exertion (class 3 or 4 angina), or a sudden intensification of symptoms in those with chronic coronary syndrome, a diagnosis of unstable angina should be considered if myocardial infarction is excluded through serial cardiac troponin measurements. The diagnosis is more likely when symptoms are associated with signs of myocardial ischaemia on the ECG, and the diagnosis is confirmed if atherothrombosis or an alternative acute coronary pathology is identified on coronary angiography with or without functional testing or intravascular imaging (1).

Unstable angina and primary myocardial infarction share similar underlying pathophysiological mechanisms, and acute coronary syndrome (ACS) is still commonly used as an umbrella term to describe these diagnoses (2). Historically, unstable angina was a frequent diagnosis in practice and was associated with adverse outcomes (107). However, by measuring cardiac troponin in stored samples, studies demonstrated that a significant proportion of patients previously diagnosed with unstable angina would be reclassified as having myocardial infarction due to the lower sensitivity of the then utilized biomarker assays (108). Those with unstable angina without an elevation in cardiac troponin had a more favourable prognosis (109,110,111). In other words, the diagnosis of unstable angina has become progressively less common over time due to the introduction of high-sensitivity cardiac troponin assays and lower diagnostic thresholds for myocardial infarction (112).

While unstable angina by definition is not associated with acute myocardial injury at the time of assessment, it is not uncommon for cardiac troponin values to be *chronically* elevated in these patients, and this is associated with an adverse prognosis (113). A systematic assessment for causes of chronic myocardial injury should be performed, but differentiating this from late presentation of myocardial infarction can be difficult (see Section 14.1.4). If there is diagnostic uncertainty, cardiac imaging or repeating the cardiac troponin measurement on a subsequent visit can help to differentiate recent myocardial infarction from other types of injury.

Despite these concerns and limitations, unstable angina has not disappeared from clinical practice and is still considered part of the spectrum of ACS (1).

## 11. Myocardial injury with non-obstructive coronary arteries

The definition of MINOCA is updated to ‘myocardial *injury* with non-obstructive coronary arteries’. The term can be applied as a working diagnosis in patients who present with clinical features of possible myocardial infarction who are subsequently found to have non-obstructive coronary arteries (no stenosis ≥50%) on coronary angiography (114,115). The previous term referred to ‘myocardial infarction’ rather than ‘myocardial injury’, but this was problematic as most patients are subsequently found to have a non-coronary cardiac (e.g. myocarditis, Takotsubo syndrome, cardiomyopathy) or non-cardiac cause (e.g. pulmonary embolism) (116,117). Updating the definition of MINOCA helps to resolve this issue, emphasizing its role as a working diagnosis for those that require further investigation to establish the underlying cause and final diagnosis. For patients in whom myocardial infarction is subsequently confirmed after further invasive or non-invasive testing, the final diagnosis should be classified as primary, secondary, or procedure-related myocardial infarction depending on the setting (see Section 7.2).

In patients with MINOCA, intravascular imaging can identify subtle abnormalities of an acute coronary pathology of primary myocardial infarction, such as plaque erosion/rupture, mural thrombus without plaque rupture, or SCAD (1,117). Similarly, invasive functional testing may identify microvascular dysfunction and/or epicardial coronary spasm that may have led to a primary myocardial infarction (1). Clinical practice guidelines further recommend CMR imaging to aid in establishing the final diagnosis, ideally performed within 2 weeks after presentation, but thereafter if necessary (1,62). When CMR has been performed, myocardial infarction is the final diagnosis in 22%–27% of patients (118,119), while more often imaging identifies an alternative non-coronary cardiac diagnosis, such as acute myocarditis or Takotsubo syndrome, resulting in a change in management (120). The diagnostic yield of CMR is higher if performed within 2 weeks of presentation (121) as after this time there may be resolution of reversible myocardial changes, particularly in Takotsubo syndrome or acute myocarditis (122), while CMR findings will persist in myocardial infarction or in those with cardiomyopathy (123).

## 12. Symptoms of myocardial infarction

Chest pain is the most commonly reported presenting symptom of acute myocardial infarction (124,125,126). The sensitivity of chest pain for a diagnosis of myocardial infarction is high and is similar among women and men (127,128), but chest pain is not specific as it can be associated with multiple other conditions (62). Additionally, some patients report epigastric pain and/or referred pain to the jaw, neck, arm, or back, and some patients do not report chest pain at all (129) and present only with associated symptoms, such as breathlessness, palpitations, nausea, or vomiting (130). Patients describe the quality of ischaemic chest pain as pressure, tightness, or squeezing, and symptom severity does not associate with a diagnosis of myocardial infarction (131); thus, chest discomfort may be a more appropriate term to describe the main symptom of myocardial ischaemia (62). Use of the terms ‘typical’ and ‘atypical’ are discouraged as studies have found that symptoms labelled as atypical are more common among women evaluated for myocardial infarction and may contribute to delayed diagnoses (132). Chest discomfort is considered acute when there is an abrupt onset or change in intensity, duration, or overall pattern. Chest discomfort is considered stable when symptoms are chronic with reliable precipitating factors of exertion or emotional stress (130). Patients with STEMI are more likely to present with persistent chest discomfort and to experience vomiting, dizziness, and diaphoresis compared with patients with NSTEMI, who are more likely to present with intermittent symptoms (125,133). The diagnosis of myocardial infarction is more often delayed among patients without chest pain, in whom the risk of death in hospital is higher (129,130). Patients presenting with secondary myocardial infarction are less likely to present with chest pain and more likely to report breathlessness or other symptoms associated with the primary diagnosis (18).

Studies of symptoms in myocardial infarction have included analyses by sex, age, race, ethnicity, and comorbidities. While chest pain is the most common symptom in both women and men, women more often present with additional symptoms that are less often recognized as cardiac by either patients or clinicians, which can contribute to delays in care (126,134). Compared with men with ACS, women had higher odds of presenting with pain between the shoulder blades, nausea or vomiting, and shortness of breath, and lower odds of chest pain and diaphoresis (135). Myocardial infarction presenting without chest pain or discomfort is more common among older persons (136) and those with diabetes mellitus (137,138,139). Therefore, it is important to systematically inquire about additional symptoms, particularly in women, older persons, and people with diabetes, while noting that the majority of these patients still present with chest pain or discomfort. There are few studies examining the effects of pain perception in myocardial infarction. Persons with an unrecognized myocardial infarction had reduced pain sensitivity to cold pressor testing compared with persons who received a diagnosis of acute myocardial infarction (126,135,140). Whilst differences in symptoms have been observed by race or ethnicity (141,142), no consistent patterns have emerged to inform diagnosis (137,138).

## 13. The electrocardiogram

**Figure 9:**
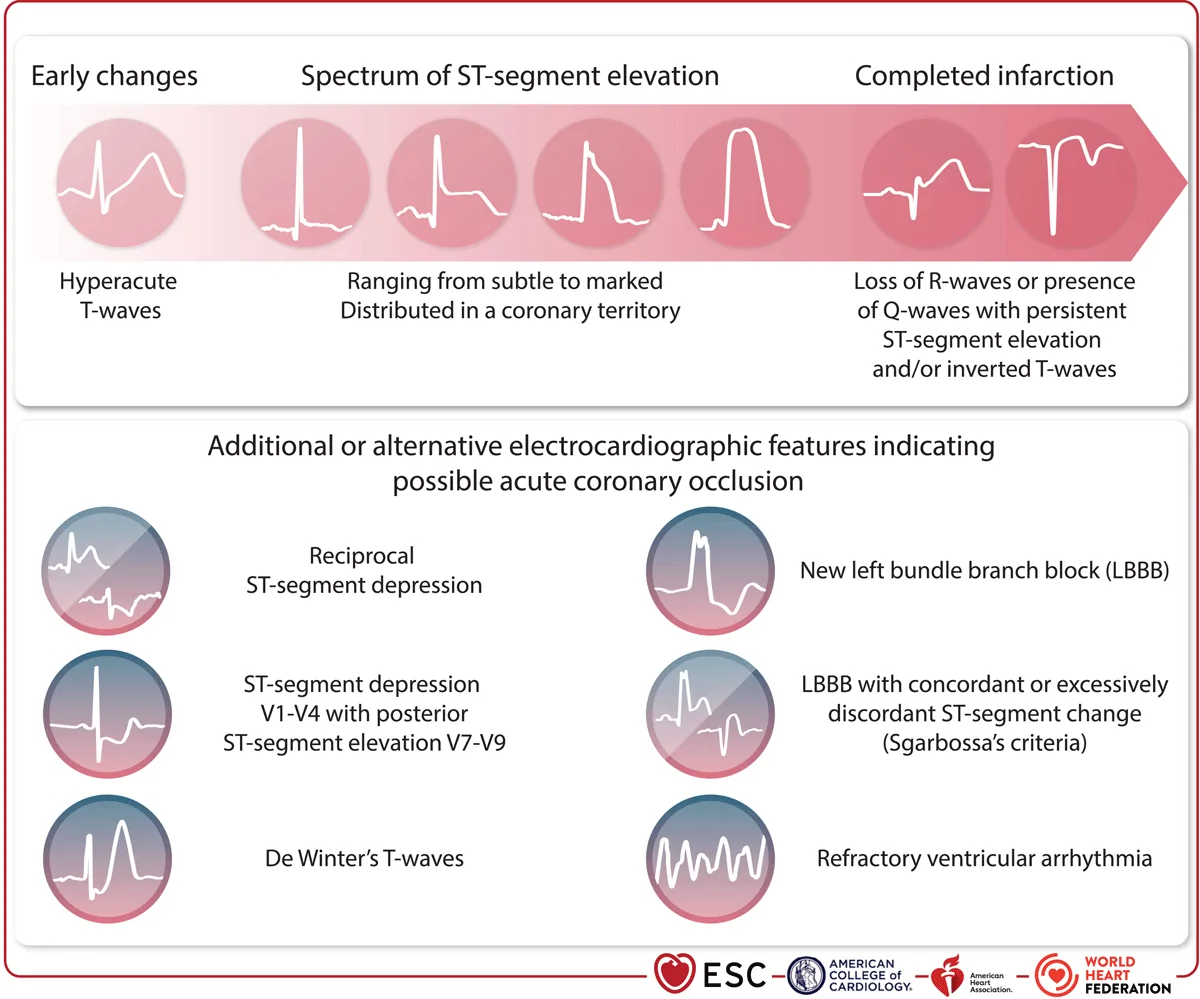
Electrocardiographic features of acute coronary occlusion. The electrocardiographic changes associated with primary myocardial infarction due to acute coronary occlusion depend on the time of presentation from the onset of persistent symptoms. The diagnosis of ST-segment elevation myocardial infarction can be made in patients with alternative electrocardiographic changes that are suggestive of acute coronary occlusion or in those who present late where the ST-segment changes have evolved.

The 12-lead ECG remains a cornerstone in the diagnosis of myocardial infarction, particularly for rapid identification of patients with symptoms of possible myocardial infarction due to acute coronary occlusion who require immediate reperfusion (Figure 9). The working diagnosis of STEMI requires new ST-segment elevation at the J-point in at least two contiguous leads: ≥2.5 mm in men <40 years, ≥2 mm in men ≥40 years, or ≥1.5 mm in women regardless of age in leads V_2_–V_3_; and/or ≥1 mm in all other leads, in the absence of left ventricular hypertrophy or left bundle branch block (LBBB) (Table 5).

**Table 5:** Electrocardiographic findings of myocardial ischaemia or infarction.


FEATURE	DESCRIPTION OF ELECTROCARDIOGRAPHIC FINDINGS OF MYOCARDIAL ISCHAEMIA OR INFARCTION

**ST-segment elevation**	New ST-segment elevation at the J-point in two contiguous leads: ≥1 mm in all leads other than leads V_2_–V_3_ where the following apply: ≥2.5 mm in males >40 years, ≥2 mm in males ≥40 years, or ≥1.5 mm in females regardless of age, in the absence of bundle branch block and/or left ventricular hypertrophy

**ST-segment depression**	New horizontal or down sloping ST-segment depression ≥0.5 mm at the J-point in two or more contiguous leads

**Hyperacute T waves**	Symmetrical, broad T waves disproportionately large to the preceding QRS complex in two contiguous leads

**de Winter T waves**	Tall, prominent, symmetrical T waves with upsloping ST segment depression in the precordial leads

**Biphasic T waves**	Initial positive deflection followed by a negative deflection

**T-wave inversion**	New or dynamic T-wave inversion ≥1 mm in two contiguous leads

**Wellens syndrome**	Biphasic or deeply inverted T waves in leads V_2_ and V_3_

**Pathologic Q waves**	Q-wave duration ≥40 ms and/or a depth of ≥25% of the R wave in two contiguous leads

**Sgarbossa criteria**	In the presence of left bundle branch block or ventricular pacing; ST-segment elevation ≥1 mm concordant (in the same direction) with the QRS complex; ST-segment depression ≥1 mm in lead V_1_, V_2_, or V_3_; ST-segment elevation ≥5 mm not concordant with the QRS complex

**Ventricular arrhythmia**	Ventricular fibrillation or ventricular tachycardia


A posterior myocardial infarction is suggested by the presence of ST-segment depression ≥1 mm in lead V_1,_ V_2_, and/or V_3_, particularly when the R-wave amplitude is greater than the S-wave (dominant) in V_1_ or V_2_, confirmed by ST-segment elevation in the posterior leads V_7_ to V_9_ (143). A right ventricular myocardial infarction is indicated by ST-segment elevation in the right precordial leads (V_3_R–V_6_R), especially when accompanied by ST-segment elevation in lead aVR.

Previous studies have shown that up to 1 in 4 patients managed as a NSTEMI without classical ST-segment elevation on conventional 12-lead ECG have an acute occlusion of the culprit artery (50,51,53). In the right clinical setting, other electrocardiographic patterns may be considered equivalent to STEMI (52). These include new or presumed new left and/or right bundle branch block when accompanied by other signs of myocardial ischaemia (144). Among patients with pre-existing LBBB or ventricular pacing the presence of ST-segment elevation ≥1 mm concordant (in the same direction) with the QRS complex; ST-segment depression ≥1 mm in lead V_1_, V_2_, or V_3_; and ST-segment elevation ≥5 mm not concordant (in the opposite direction) with the QRS complex suggest acute coronary occlusion (Sgarbossa criteria) (145). The modified Sgarbossa criteria may improve the detection of acute coronary occlusion with LBBB or ventricular pacing by replacing the absolute discordant ST-segment elevation threshold with the proportional rule of an ST/S-wave ratio ≤0.25 (146). Further electrocardiographic patterns that suggest acute coronary occlusion include marked ST-segment depression or hyperacute T waves in V_1_–V_2_ with reciprocal changes elsewhere, de Winter pattern (upsloping ST-segment depression with tall, symmetric T waves in V_2_–V_5_) (147), Wellens syndrome (biphasic or deeply inverted T waves in V_2_–V_3_ during pain-free intervals) (148), Aslanger pattern (ST-segment elevation isolated to lead III with ST-segment depression in any of leads V_4_–V_6_ with a positive T wave), and the ‘South African flag’ sign (ST-segment elevation in leads I, aVL, and V_2_ and ST-segment depression in lead III) (52).

Patients with previous coronary bypass grafts and/or complex CAD (e.g. chronic total occlusions with collateral supply) may present with acute coronary or graft occlusion without typical ST-segment elevation, and a high index of suspicion for myocardial infarction is required (144).

ST-segment elevation is not specific to myocardial infarction and can also be found in early repolarization or other cardiac (e.g. myocarditis, pericarditis, Brugada syndrome, and Takotsubo syndrome or cardiomyopathies) and non-cardiac (e.g. pulmonary embolism, hyperkalaemia, hypothermia, elevated intracranial pressure) conditions. With an acute coronary pathology, ST-segment elevation is typically regional and often accompanied by reciprocal ST-segment depression. In contrast, in other conditions such as pericarditis or early repolarization, ST-segment elevation is often diffuse or global, not confined to a single coronary territory, and occurs without reciprocal changes (149).

In addition to ST-segment elevation, electrocardiographic findings associated with acute myocardial ischaemia include ST-segment depression, T-wave inversion, hyperacute or biphasic T waves, pathological Q waves, and ventricular arrhythmias (Table 5). New horizontal or down-sloping ST-segment depression ≥0.5 mm and/or T-wave inversion >1 mm in two contiguous leads is suggestive of myocardial ischaemia. Global ischaemic changes (diffuse ST-segment depression along with ST-segment elevation in lead aVR) may reflect left main stem or balanced three vessel CAD. However, these electrocardiographic findings are not specific to myocardial infarction and can be observed in other cardiac and non-cardiac conditions.

Patients who present late following myocardial infarction due to an acute coronary occlusion are more likely to have pathological Q waves in two or more contiguous leads and biphasic T waves or T-wave inversions rather than ST-segment elevation or depression—although some patients presenting late may not necessarily have developed Q waves despite an acute coronary occlusion (150). Q waves are usually permanent but may regress and disappear over months to years. The classical definition of a pathological Q wave requires a Q-wave duration ≥40 ms and/or a depth of ≥25% of the R wave in the same lead (151). While an alternative pathological Q wave definition was suggested in prior versions of the UDMI, the classical definition has a higher correlation with transmural myocardial infarction in CMR imaging studies (98).

In the setting of secondary myocardial infarction global ischaemic changes are more common (64). More marked electrocardiographic changes, particularly ST-segment elevation and depression, that do not resolve rapidly with treatment of the underlying cause (e.g. resolution of widespread ST-segment depression after cardioversion for tachyarrhythmia) should lead to reconsideration as to whether the diagnosis is primary myocardial infarction.

Procedure-related myocardial infarction presents with similar electrocardiographic findings as primary myocardial infarction. Early stent thrombosis often presents with classical ST-segment elevation with reciprocal ST-segment depression, while smaller procedural myocardial infarcts, such as side branch occlusion, may have more subtle or no electrocardiographic changes. Comparison with the pre-procedural ECG can be helpful. New electrocardiographic changes, such as ST-segment elevation or depression and T-wave inversion, are common after cardiac surgery and do not always indicate acute graft failure (43). For example, following cardiac surgery, post-pericardiotomy syndrome (an autoimmune inflammatory response to pericardial and pleural trauma), pneumopericardium (air in the pericardial space), and metabolic disturbances (due to haemodilution, renal dysfunction, blood products, and/or diuretics) can cause ST-segment elevation, depression, and/or T-wave changes. Furthermore, Q waves that meet the definition for a pathological Q wave can be transient early after cardiac surgery (80). The early use of cardiac imaging and invasive coronary angiography is required to confirm whether these electrocardiographic changes are due to procedure-related myocardial infarction.

## 14. Cardiac biomarkers

Acute myocardial injury is integral to the diagnosis of myocardial infarction whether primary, secondary, or procedure related. Since 2007, the UDMI has recommended cardiac troponin I or T (henceforth referred to as ‘cardiac troponin’) as the preferred biomarkers for the diagnosis of myocardial infarction due to their greater specificity for myocardial injury than other biomarkers (6). The definition of acute myocardial injury as a rise and/or fall in cardiac troponin with at least one value above the 99th percentile URL has remained consistent, with the use of sex-specific 99th percentile URLs recommended by the Fourth UDMI in 2018 when using high-sensitivity assays (8). Early diagnostic pathways incorporating high-sensitivity cardiac troponin assays, with some available at the point of care, have created opportunities to accelerate the diagnosis of patients with possible myocardial infarction. A diagnosis of myocardial infarction requires an understanding of cardiac troponin assay performance, decision threshold limitations, potential analytical interference, and when serial testing or alternative biomarkers are needed to differentiate acute from chronic elevation. Alternative biomarkers for myocardial infarction are covered in Section 14.2 and implications for diagnosis of myocardial infarction in low-resource settings are discussed in Section 19.

### 14.1. Cardiac troponin

Cardiac troponin plays a key role in myocardial contraction, and immunoassays that quantify cardiac troponins in the circulation play an important role in the diagnosis of myocardial infarction and recognition of both acute and chronic cardiac conditions (152). In the sarcomere, cardiac troponin I (inhibitory), T (tropomyosin-binding), and C (calcium-binding) subunits exist in a complex, which is released into the circulation as an intact ternary complex and as subunit fragments in acute myocardial infarction (153). Cardiac troponin I and T have a half-life of 2 h and between 2 and 4 h in the circulation, respectively (154). Immunoassays are available that target amino acid sequences that are specific to the cardiac isoforms of troponin I and T, which ensures these assays provide high specificity for myocardial injury.

High-sensitivity assays have greater analytical precision to enable detection of cardiac troponin at very low concentrations and are now available on multiple central laboratory and point-of care platforms (155). For classification as a high-sensitivity assay, cardiac troponin assays should achieve a coefficient of variation of ≤10% at the sex-specific 99th percentile URL and be able to detect cardiac troponin in more than 50% of healthy females and males in the reference population (156). Greater precision at the diagnostic threshold for myocardial infarction improves the reliability of cardiac troponin testing in practice. As such, high-sensitivity assays are recommended by clinical practice guidelines (1,62) and are now used in many countries around the world (157). These assays have enabled implementation of accelerated diagnostic pathways and more precise estimates of the 99th percentile URL for the diagnosis of myocardial infarction. As cardiac troponin assays are not standardized by the manufacturers, evidence from clinical studies may not apply to all assays and clinicians should refer to the International Federation of Clinical Chemistry and Laboratory Medicine Biomarkers Reference Tables (155) and apply assay-specific thresholds in practice.

#### 14.1.1. Defining the 99th percentile upper reference limit

Since there is no gold standard for the diagnosis of myocardial infarction that is independent of cardiac troponin, it is not possible to define a decision threshold based on diagnostic performance studies. Therefore, the decision threshold is based instead on the distribution of cardiac troponin in reference populations without known cardiac conditions or cardiovascular risk factors. The 99th percentile URL is recommended to standardize the approach to recognizing those with myocardial injury across different assays, between sites, and across healthcare systems. It is conventional to use the 97.5th percentile as the URL for most biomarkers, but a higher threshold (the 99th percentile) is applied along with a rise and/or fall in cardiac troponin to increase the specificity for a diagnosis of myocardial infarction, as many other conditions also increase cardiac troponin concentrations. Given that the identification of those with myocardial injury is integral to the diagnosis of myocardial infarction, it is important that the 99th percentile is determined in sufficiently large and representative studies (158).

Cardiac troponin concentrations vary considerably by sex (159,160). For high-sensitivity cardiac troponin I and T assays, the 99th percentile URL in females is lower than the uniform URL (161,162). In contrast, the 99th percentile URL in males is above the uniform URL. As such, sex-specific 99th percentile URLs are necessary to define myocardial injury and avoid systematic bias that could contribute to the under-recognition of myocardial infarction and other cardiac conditions associated with myocardial injury in female patients (163). Historically, when using a cardiac troponin I assay the use of sex-specific URLs compared with a uniform URL reclassified a greater proportion of females as having myocardial injury than the same comparison using a cardiac troponin T assay (163,164). However, the impact of sex-specific URLs is likely to be similar for cardiac troponin I and T when using the latest generation high-sensitivity cardiac troponin T assay (165). Enhanced precision at low concentrations has increased the proportion of healthy females in whom cardiac troponin T is quantifiable, enabling the accurate determination of URLs for both sexes: the URL in females is half the URL in males for cardiac troponin T in a large and representative global healthy reference population (165).

Whilst differences between female-specific and male-specific 99th percentile thresholds are similar in magnitude across all age ranges for both cardiac troponin I and T assays (157), the age of the healthy reference population used to define the 99th percentile is particularly important as cardiac troponin increases significantly with age (159,166). Differences in cardiac troponin by age are as important as the differences observed by sex; however, unlike sex, age is a continuous measure, therefore defining appropriate age-adjusted 99th percentiles is more challenging. Most regulatory studies that define the normal reference range for cardiac troponin assays do not include a large enough sample of younger and older persons to reliably determine age-adjusted URLs, and no cardiac troponin assays have sought regulatory approval for the use of age-adjusted thresholds in clinical practice. Clinicians should be aware that 1 in 3 patients over 75 years with possible myocardial infarction have an elevated cardiac troponin concentration compared with 1 in 20 patients under 50 years old (167). This is partly because older persons are more likely to have comorbid conditions that influence cardiac troponin, such as pre-existing or subclinical cardiac conditions or kidney disease, hypertension, and diabetes, and partly because the incidence of spontaneous/primary or secondary myocardial infarction is highest in this age group. Serial measurements are helpful to differentiate acute from chronic causes of elevated cardiac troponin values when there is uncertainty (see Section 6). Further research is required to define age-adjusted 99th percentile URLs for females and males in sufficiently large reference range populations, and to determine whether their application would improve the recognition of younger and older patients with myocardial injury due to myocardial infarction or other cardiac conditions (see Section 20). In the future, the use of statistical models or machine learning tools that incorporate variables beyond age and sex to include a measure of renal function or knowledge of pre-existing conditions could improve how normal cardiac troponin values are defined (168).

#### 14.1.2. Analytical considerations

Immunoassays for cardiac troponin are no different from immunoassays for other biomarkers in that they are susceptible to pre-analytical and analytical factors that can result in false positives and negatives (169). For example, haemolysis and biotin can reduce, and fibrin micro-clots can increase, cardiac troponin concentrations (170,171). Analytical performance of assays at the point of care may be more variable in whole blood compared with plasma or serum on a central laboratory platform (172).

When the assay result is unexpected, the measurement should be repeated with a new sample. Heterophilic antibodies may interfere with immunoassays *ex vivo* resulting in analytical false positive values of cardiac troponin when in fact there is no elevation *in vivo*. However, with most cardiac troponin assays, heterophilic antibodies are an uncommon cause for false positive results (173). Similarly, immunoglobulin binding with cardiac troponin *in vivo* can result in macrotroponin complexes (174) that are slowly cleared from the circulation with a half-life of several weeks, resulting in high measurable cardiac troponin concentrations even in the absence of acute or chronic myocardial injury (175).

Interference should be suspected when elevated cardiac troponin is not consistent with the clinical findings (176), and is more likely when values increase and decrease with no apparent pattern on serial testing. Interference can be readily identified by measuring cardiac troponin using an alternative immunoassay, with discordance between cardiac troponin I and T or between cardiac troponin I assays being strongly suggestive of interference (175). Macrotroponin complexes can be identified using immunoglobulin depletion or sucrose gradient ultracentrifugation in specialist laboratories (169). The frequency of antibody-mediated interference and macrotroponin complexes in patients with possible myocardial infarction is unknown but may be responsible for as many as 1 in 2 elevated cardiac troponin I values in asymptomatic populations (177).

These analytical issues can also influence the accurate determination of the 99th percentile, which is particularly susceptible to outliers. Recent recommendations on sample processing, the size of the population, and the inclusion and exclusion criteria to define a truly healthy reference population, and the application of statistical methods to exclude outliers, should improve the reliability and consistency of studies defining the 99th percentile URL (158). Larger sample sizes are likely to improve precision.

#### 14.1.3. Serial measurements

Serial cardiac troponin testing is generally needed to differentiate acute myocardial infarction from other causes of myocardial injury in patients with possible primary, secondary, and procedure-related myocardial infarction. The timing of serial measurements should be guided by a validated diagnostic pathway (see Section 14.1.5).

**Figure 10:**
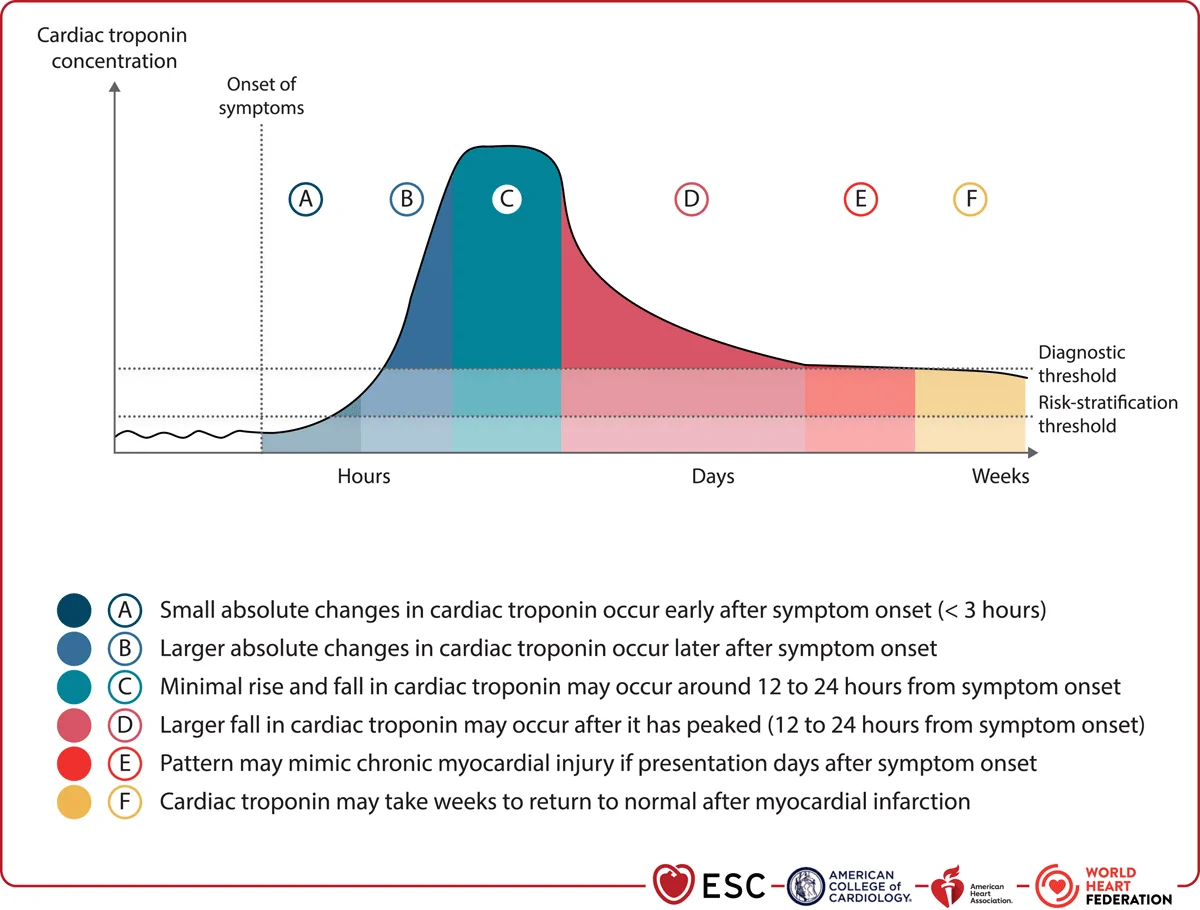
Rise and fall in cardiac troponin over time from symptom onset. Following the onset of acute myocardial infarction, changes in cardiac troponin vary over time: **(A)** Small absolute changes in cardiac troponin occur early after symptom onset (<3 h). **(B)** Larger absolute changes in cardiac troponin occur later after symptom onset. **(C)** Minimal rise and fall in cardiac troponin may occur around 12 to 24 h from symptom onset. **(D)** Larger fall in cardiac troponin may occur after it has peaked (12 to 24 h from symptom onset). **(E)** Pattern may mimic chronic myocardial injury if presentation days after symptom onset. **(F)** Cardiac troponin may take weeks to return to normal after myocardial infarction and some patients may develop chronic myocardial injury following myocardial infarction.

When using a high-sensitivity assay, very low cardiac troponin values below the limit of detection, limit of quantification, or a defined risk stratification threshold can be used to safely exclude myocardial infarction with a single measurement in many patients (66,178). In contrast, serial testing is required if higher thresholds are used to exclude myocardial infarction as the sensitivity of the 99th percentile URL at presentation is around 90% overall and as low as 70% among patients presenting within 3 h of symptom onset (179,180). The release kinetics of cardiac troponin follow a well-defined pattern in primary myocardial infarction, with increasing levels detectable within 2 to 3 h of symptom onset, peaking between 12 and 24 h, and remaining elevated for days or weeks (Figure 10). The release kinetics differ depending on whether there is acute coronary occlusion at presentation, and whether spontaneous or therapeutic epicardial and tissue level reperfusion occurs. One cannot rely on cardiac troponin results in the first few hours in patients with ST-segment elevation as levels can be below the 99th percentile URL in around 1 in 6 patients due to lack of perfusion of the infarcted territory (181). Among those with microvascular obstruction the peak cardiac troponin concentration is lower and delayed if intramyocardial haemorrhage is not present, whereas it is higher and occurs earlier in those with intramyocardial haemorrhage (182,183).

Less is known about the role of serial measurements for the diagnosis of secondary myocardial infarction due to supply–demand imbalance. In secondary myocardial infarction the presentation and maximum value of cardiac troponin may be lower than in primary myocardial infarction, but there is substantial overlap in values between these settings (71). On serial testing the absolute and relative changes in cardiac troponin concentrations are similar in both primary and secondary myocardial infarction and among those with other causes of acute myocardial injury (71). Interestingly, the initial cardiac troponin values are more similar (less than two-fold difference) than the peak values (up to five-fold difference) when comparing primary and secondary myocardial infarction. This could be due to delays in cardiac troponin release in those with acute coronary occlusion, differences in the amount of myocardium at jeopardy, or because ischaemic injury is more likely to persist in primary than in secondary myocardial infarction, where the trigger of supply–demand imbalance may be rapidly corrected (e.g. with cardioversion).

Following cardiac surgery, cardiac troponin is elevated in all patients, and in 97.5% this exceeds the previously recommended diagnostic threshold of >10 times the URL (42). Interestingly, peak cardiac troponin concentrations occur earlier after surgery (3 to 12 h) (42) than after primary myocardial infarction (12 to 24 h). This may reflect differences in cardiac troponin release following reperfusion or differences in the mechanism of myocardial injury, which in most patients undergoing cardiac surgery is due to surgical manipulation or limitations of cardioplegia without ongoing injury thereafter. Those who sustain a procedure-related myocardial infarction after cardiac surgery as identified by new late enhancement on CMR imaging are more likely to have a later peak (88). A peak in cardiac troponin 12 to 24 h after cardiac surgery may indicate a procedure-related complication, given that values typical peak within 3 to 12 h (89).

Similarly, after PCI in patients with a chronic coronary syndrome, an increase in cardiac troponin concentration above the 99th percentile URL occurs in 1 in 2 patients (184), with the majority having a peak value 12–24 h following intervention (185). The timing of peak values differs between those with and without elevated values at baseline who are undergoing intervention for acute and chronic coronary syndromes, respectively (186). New late enhancement on CMR imaging is more likely among those with elevated cardiac troponin concentrations 24 h following PCI than those without (187). Among patients with myocardial ischaemia or an overt complication of PCI, serial cardiac troponin measurements at baseline and 12–24 h after the event may be needed to recognize those with acute myocardial injury.

#### 14.1.4. Defining a significant rise and fall

No single absolute or relative value can be applied to define a significant rise or fall in cardiac troponin that applies to all clinical presentations and assays. Whilst accelerated diagnostic pathways use assay-specific thresholds combined with delta criteria to increase the likelihood that myocardial injury is due to myocardial infarction (see Section 14.1.5), clinical judgment must be used to determine whether a particular change is sufficient to confirm the diagnosis of myocardial infarction, considering several relevant factors.

The influence that the timing of testing has on the rise and fall in cardiac troponin is illustrated in Figure 10. First, the **time from symptom onset** at which samples are obtained affects the magnitude of change in cardiac troponin concentrations on serial testing (8,188,189). Smaller absolute changes are observed within 3 h of symptom onset, with a more substantial increase observed between 3 and 12 h. When samples are obtained around the time of the peak concentration (12–24 h), there may be minimal changes on serial testing. Similarly, among those who present many days after the onset of symptoms, the pattern may mimic chronic myocardial injury. Differentiating residual acute myocardial injury following a recent myocardial infarction from chronic myocardial injury is particularly important. Second, the **time interval between samples** will substantially influence the absolute value of the rise or fall in cardiac troponin concentrations. For this reason, fixed time intervals are recommended at 1 or 2 h for accelerated diagnostic algorithms to improve the reliability of these criteria (190). Third, the **baseline concentration** is important. For cardiac troponin concentrations below or around the 99th percentile URL, absolute delta values rather than relative values improve precision (190). It is important to note that absolute delta values are assay-specific, and a cut-off value cannot be transferred from one assay to another (156). For clearly elevated baseline values, relative changes are more intuitive (8). However, for very high cardiac troponin values, it may not be necessary to demonstrate a rise and/or fall, as such values are rarely observed in those with chronic myocardial injury (156). Please see Section 6 for a more in-depth discussion of the criteria used to define acute and chronic myocardial injury.

#### 14.1.5. Accelerated diagnostic pathways

One of the advantages of high-sensitivity cardiac troponin assays has been their use in accelerated diagnostic pathways. Multiple pathways have been validated for different assays, with a similar goal of improving the safety and efficiency of the assessment of patients with possible myocardial infarction without ST-segment elevation on the ECG (191,192). These pathways are illustrated in clinical practice guidelines (1,2,193) and can be broadly classified into three types: (i) single-sample rule-out pathways that classify patients into two groups (low- or high-risk); (ii) serial testing pathways with repeated measurements at 1 or 2 h that use multiple thresholds and delta values to stratify patients into three groups (low-risk, observe, or high-risk); and (iii) conventional pathways that combine a clinical risk score with the 99th percentile at 0 and 3 h. Single-sample rule-out pathways use assay-specific optimized thresholds below the 99th percentile to identify the greatest proportion of low-risk patients as potentially suitable for discharge from the Emergency Department with a high sensitivity and negative predictive value for myocardial infarction (63). A similar approach is applied in 0/1- and 0/2-hour serial testing pathways that also incorporate assay-specific optimized thresholds above the 99th percentile to identify high-risk patients with greater specificity and positive predictive value (194).

The thresholds used to triage patients with possible myocardial infarction in accelerated diagnostic pathways are often not based on the 99th percentile of the cardiac troponin assay and here uniform rather than sex-specific thresholds have been applied. For example, in the 0/1- and 0/2-hour serial testing pathways recommended by the ESC the same thresholds are applied in both male and female patients to differentiate those at low risk and suitable for discharge from those at increased risk who require further investigation or hospital admission (1). It should be noted that these thresholds are used for risk stratification, rather than to define myocardial injury and confirm a final diagnosis of myocardial infarction, which is based on demonstrating a rise and/or fall in cardiac troponin where at least one value is above the 99th percentile URL and sex-specific thresholds apply (see Section 6).

#### 14.1.6. Interpretation of cardiac troponin for diagnosis

Although the introduction of high-sensitivity assays and accelerated diagnostic pathways has contributed considerably to the early diagnosis of myocardial infarction, some additional considerations are needed when interpreting cardiac troponin values in patients with possible myocardial infarction (Box 6).

**Box 6 Interpretation of cardiac troponin for diagnosis of myocardial infarction**

- In patients with possible myocardial infarction, cardiac troponin needs to be interpreted in conjunction with clinical assessment including a history, physical examination, and 12-lead electrocardiogram.
- In patients who present early after the onset of symptoms, increases in cardiac troponin concentration may not be detected even with high-sensitivity assays, and serial cardiac troponin testing is necessary.
- In patients with recurrent or persistent symptoms of myocardial ischaemia, serial cardiac troponin testing is needed prior to excluding myocardial infarction.
- In unselected patients without a clinical indication for diagnostic testing, elevated cardiac troponin concentrations are common in multiple conditions, such as sepsis, kidney disease, or stroke.
- The 99th percentile for cardiac troponin is lower for females than males; the use of high-sensitivity assays with sex-specific thresholds will minimize the risk of underdiagnosis of myocardial injury and infarction in females compared with using a uniform threshold.
- Age is an important consideration when interpreting cardiac troponin as the prevalence of myocardial infarction increases with age, as does the prevalence of persistently elevated cardiac troponin values.
- When cardiac troponin concentrations are elevated, unless the diagnosis of myocardial infarction is confirmed, serial measurements are typically required to differentiate acute from chronic causes of myocardial injury.
- Analytical interference should be suspected when an elevated cardiac troponin value is not consistent with the clinical findings and is more likely when cardiac troponin values vary with no apparent pattern on serial testing.

### 14.2. Alternative biomarkers of myocardial injury

The most sensitive and specific biomarker for the diagnosis of myocardial infarction is cardiac troponin measured using a high-sensitivity assay. In a recent survey of practice from 663 laboratories in 76 countries from Europe, Africa, South and North America, and Australasia, cardiac troponin testing was available in 98% of hospitals, and in 92% of these hospitals, testing was with a high-sensitivity assay (157). If a high-sensitivity assay is not available, then a cardiac troponin assay that is not high-sensitivity is preferred over CK-MB, which has lower specificity and sensitivity (44). If neither type of cardiac troponin assay is available, then an alternative would be to use CK-MB measured by a mass assay (44,83). A number of alternative biomarkers of myocardial injury are in development, such as cardiac myosin-binding protein C (cMyC). cMyC is a sarcomeric protein that is loosely associated with both myosin and actin, is more abundant than cardiac troponin, and rises more rapidly after iatrogenic myocardial injury, suggesting a role in early evaluation (195). Prototype cMyC assays have comparable performance to cardiac troponin assays in accelerated diagnostic pathways for myocardial infarction (196,197), but whether testing of cMyC in combination with cardiac troponin will improve the diagnosis of myocardial infarction in patients for whom the interpretation of cardiac troponin is challenging due to analytical interference or kidney disease remains uncertain. And for these reasons, biomarkers other than cardiac troponin are not used routinely for the diagnosis of myocardial infarction.

## 15. Coronary and cardiac imaging

While a likely diagnosis of myocardial infarction can be made without imaging, confirmation of the diagnosis and determination of the underlying mechanism requires coronary angiography and/or cardiac imaging.

### 15.1. Invasive imaging

#### 15.1.1. Invasive coronary angiography

Invasive coronary angiography is the gold-standard test for the evaluation of patients with possible myocardial infarction, with the timing of coronary angiography defined in clinical practice guidelines (1,2,62). Not only can invasive coronary angiography confirm the diagnosis, but it can also determine the underlying acute coronary pathology in primary myocardial infarction. The prevalence of acute coronary pathologies other than atherothrombosis varies widely: SCAD (0.1%–4.0%), coronary embolism (0.2%–13%), epicardial coronary vasospasm (1%–20%), in-stent restenosis (1.0%–6.5%), stent thrombosis (0.6%–0.8%), and graft failure (unknown) (61,198,199,200,201). This variation is likely due to differences in the populations studied, enrolment criteria (systematic or selected), lack of central adjudication, and variation in the use of adjuvant coronary or cardiac imaging, which can help to identify the culprit lesion and underlying pathology (1,2).

Historically, many patients with myocardial ischaemia secondary to oxygen supply–demand imbalance have not undergone coronary angiography, with the diagnosis made based on clinical assessment (64). Due to minimal use of coronary angiography in this setting, patients with primary myocardial infarction can be missed, and some patients with non-ischaemic acute myocardial injury will be misdiagnosed as having secondary myocardial infarction. In a prospective study of patients with type 2 myocardial infarction defined using the previous classification who underwent systematic coronary angiography, 1 in 20 patients were found to have a primary myocardial infarction due to atherothrombosis (72). The use of more objective imaging criteria to define secondary myocardial infarction in this cohort reduced the diagnosis of myocardial infarction as most had neither obstructive CAD nor new left ventricular impairment (74).

In many parts of the world, particularly in low-resource settings, there is limited or no access to invasive coronary angiography (9). In some patients (e.g. those with a relative contraindication, limited life expectancy, or cognitive impairment), or based on patient values and preferences, invasive coronary angiography may not be performed. Consequently, many individuals with possible myocardial infarction do not undergo this key diagnostic test. In these situations, cardiac imaging can confirm the diagnosis, or when imaging is also not possible or not diagnostic, a clinical diagnosis of myocardial infarction can be made based on clinical presentation, the ECG, and cardiac biomarkers. Additionally, in some situations, such as in low-risk individuals, possible secondary myocardial infarction, or where the likelihood of revascularization is low, CCTA may be a useful alternative to aid the diagnosis and/or to guide the need for invasive coronary angiography, where available (1).

#### 15.1.2. Intravascular imaging

Intravascular imaging, comprising IVUS and OCT, if available, can be used to identify plaque rupture and abnormal coronary vessel wall morphology in patients with possible myocardial infarction (1). This is important as approximately 14% of patients presenting with NSTEMI lack a clearly identifiable culprit lesion on invasive angiography (202). Moreover, earlier studies reported that in non-Q wave myocardial infarction, more than a third of patients had no culprit lesion detected on angiography (203). Diagnostic uncertainty is more common among those without obstructive CAD (see Section 11) or with an acute coronary mechanism of primary myocardial infarction other than atherothrombosis (204,205,206). Intravascular imaging can confirm atherothrombosis and the type of plaque morphology (rupture, erosion, intraplaque cavity, mural thrombus) and/or identify an alternative acute coronary pathology, such as intramural haematoma due to SCAD, isolated thrombus in coronary embolism, in-stent restenosis, stent thrombosis, or intimal bumping consistent with coronary artery spasm (117,207).

#### 15.1.3. Invasive functional testing

Additional invasive functional testing may enhance evaluation in patients with possible myocardial infarction. In patients with spontaneous onset of symptoms and possible primary myocardial infarction, provocative testing (e.g. acetylcholine or ergonovine challenge) may aid in the recognition of epicardial or microvascular coronary spasm when coronary arteries appear angiographically non-obstructive (3). In patients with possible secondary myocardial infarction, particularly where the severity of coronary stenosis is unclear, invasive functional assessment using fractional flow reserve (FFR) or resting indices (e.g. resting full-cycle ratio [RFR] or instantaneous wave-free ratio [iFR]) may be helpful in detecting functionally significant stenosis that could cause myocardial ischaemia in the context of supply–demand mismatch (3,208,209). Quantitative coronary angiography, in combination with computationally derived FFR, may also play a role in the detection of functionally significant lesions and may be helpful in supporting a diagnosis of secondary myocardial infarction (210).

### 15.2. Non-invasive imaging

Non-invasive cardiac imaging with echocardiography, CMR imaging, cardiac CT, single-photon emission computed tomography (SPECT), or positron emission tomography (PET) is valuable to help confirm the diagnosis of myocardial infarction or to identify an alternative mechanism for acute myocardial injury. In patients with confirmed myocardial infarction based on coronary angiography, the presence of regional wall motion abnormalities, loss of viable myocardium, and assessment of infarct size can corroborate the diagnosis and inform prognosis and treatment (211,212,213,214). Additionally, non-invasive imaging can identify complications of myocardial infarction, such as mitral regurgitation due to papillary muscle rupture, left ventricular aneurysm or pseudoaneurysm, ventricular septal defect, ventricular thrombus, right ventricular infarction, or pericardial effusion. Cardiac imaging can identify alternative mechanisms of acute myocardial injury, such as myocarditis, Takotsubo syndrome, or other cardiomyopathic processes (213,215,216,217).

#### 15.2.1. Echocardiography

Echocardiography is a key bedside imaging tool in myocardial infarction, allowing assessment of regional wall motion abnormalities, infarct extent, ventricular thrombus, pseudoaneurysm, pericardial effusion/haematoma, and valvular dysfunction. It can identify alternative causes of the presentation, such as acute pericarditis, severe aortic stenosis, aortic dissection, or massive pulmonary embolism (213,215,216,217). Point-of-care, hand-held echocardiography, when performed by trained personnel, can detect regional wall motion abnormalities induced by myocardial ischaemia almost immediately (218). When accompanied by evidence of acute myocardial injury, echocardiography can confirm the diagnosis of myocardial infarction.

Echocardiography has similar utility in the diagnosis of primary, secondary, and procedure-related myocardial infarction. In primary myocardial infarction, detection of regional wall motion abnormalities that correspond to the territory of the affected coronary artery can resolve uncertainty (211,212,213). In secondary myocardial infarction, differentiating between a global reduction in left ventricular function and regional wall motional abnormalities is important and can inform the diagnosis (214). In procedure-related myocardial infarction, echocardiography can identify new regional wall motion abnormalities, assess left ventricular ejection fraction, and identify mechanical complications, and microvascular obstruction can be visualized using contrast echocardiography (213,219,220). It should be noted that immediately after cardiac surgery, adequate echo windows are not always obtainable. Further, not all myocardial infarction results in regional wall motion abnormalities, and where there is doubt, contrast echocardiography and CMR imaging can be helpful (221,222,223,224).

#### 15.2.2. Cardiac magnetic resonance imaging

CMR imaging provides detailed assessment of both reversible and irreversible myocardial injury. It can detect myocardial infarction, oedema/inflammation, microvascular obstruction, and intramyocardial haemorrhage (225,226). Comprehensive CMR protocols include cine imaging (function), T2 (myocardial oedema and inflammation, intramyocardial haemorrhage), T2* images (intramyocardial haemorrhage), first-pass perfusion and early gadolinium enhancement (microvascular obstruction), and late gadolinium enhancement (LGE) for myocardial necrosis, microvascular obstruction, and intramyocardial haemorrhage (227). Combining T2 with LGE helps discriminate between acute and chronic myocardial injury, though mild oedema may persist for weeks in some infarcts (228,229,230). In patients with MINOCA the diagnostic yield is highest when CMR is performed within 2 weeks of myocardial infarction (224), whilst in patients with a large primary myocardial infarction, oedema, microvascular obstruction and intramyocardial haemorrhage can persist for up to 6 months before resolving and evolving into fibrosis identifiable through LGE.

**Figure 11:**
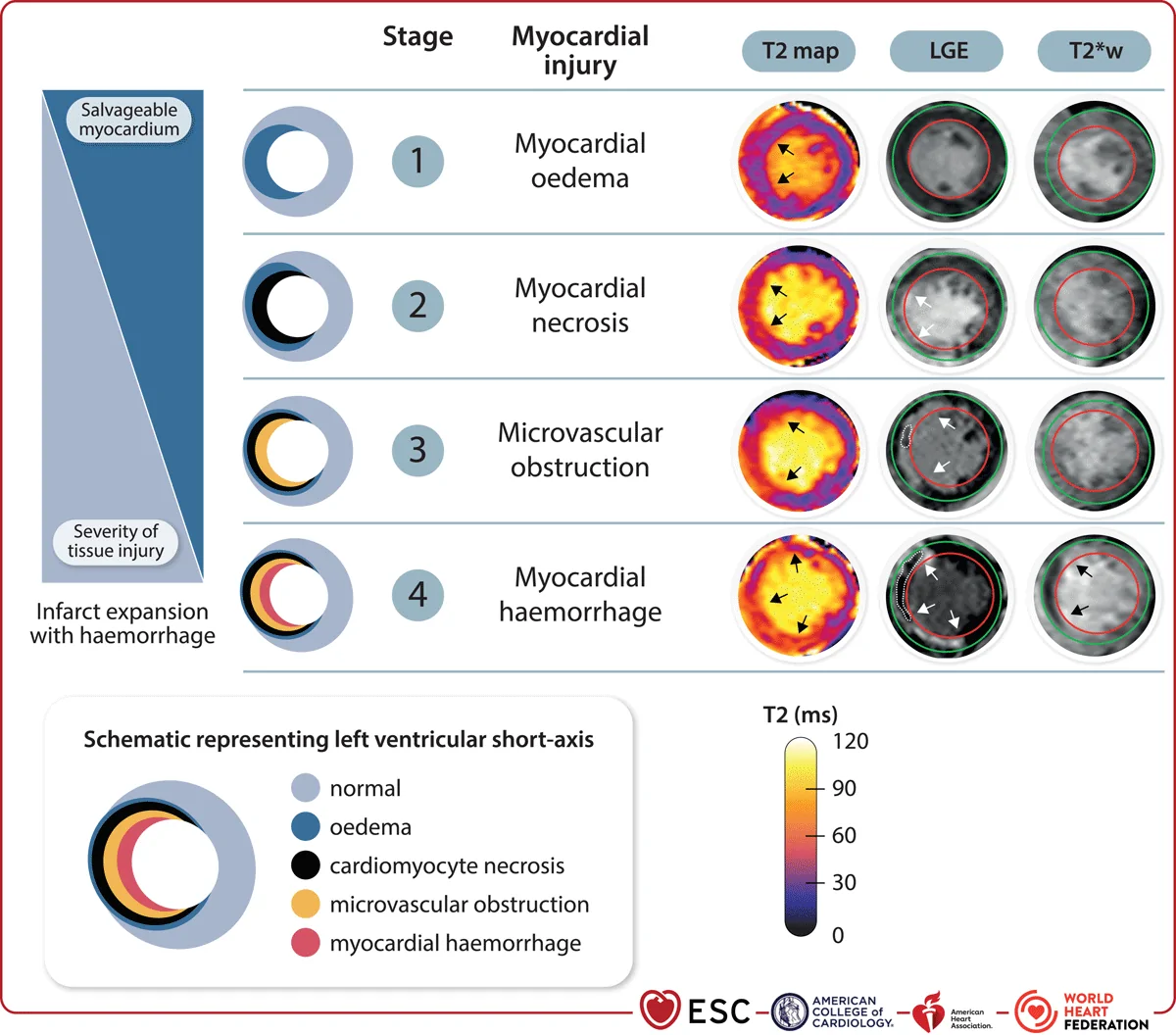
Stages of primary myocardial infarction by cardiac magnetic resonance imaging. LGE, late gadolinium enhancement. Each stage of myocardial injury is progressive and is discernible from aggregate evidence derived from T2 maps (colour bar provided), LGE, and T2*-weighted (T2*w) short-axis cardiac magnetic resonance images along each row. *Stage 1* is marked by oedema only (high signal on T2 map), absence of myocardial necrosis (lack of signal enhancement on LGE image), or myocardial haemorrhage (absence of low signal on T2*w images). *Stage 2* demonstrates both myocardial necrosis (high signal on LGE image) and oedema (high signal on T2 map) but absence of persistent microvascular obstruction (absence of low signal within the zone of LGE image) or myocardial haemorrhage. *Stage 3* highlights the presence of microvascular obstruction (presence of low signal within the zone of high signal on LGE image) along with myocardial necrosis and oedema. Note that myocardial haemorrhage is also absent at Stage 3 as evidenced by lack of low signal within the zone of infarction on T2*w image. *Stage 4* is characterized by myocardial haemorrhage (low signal within the zone of high signal in the T2*w image), along with microvascular obstruction, myocardial necrosis, and oedema. High signal (brighter than surrounding tissue) and low signal (darker than surrounding tissue). Black arrowheads point to high signal on T2 maps, white arrow heads point to high signal on LGE, and blue arrow heads point to low signal on T2*-w image. Note that low signal zones on Stage 3 and Stage 4 within LGE are demarcated by contoured zones within high signal zones in LGE. Red and green contours on LGE and T2w images are endocardial and epicardial contours segmenting the left ventricular myocardium. Adapted from Kumar *et al*. with permission (232).

**Figure 12:**
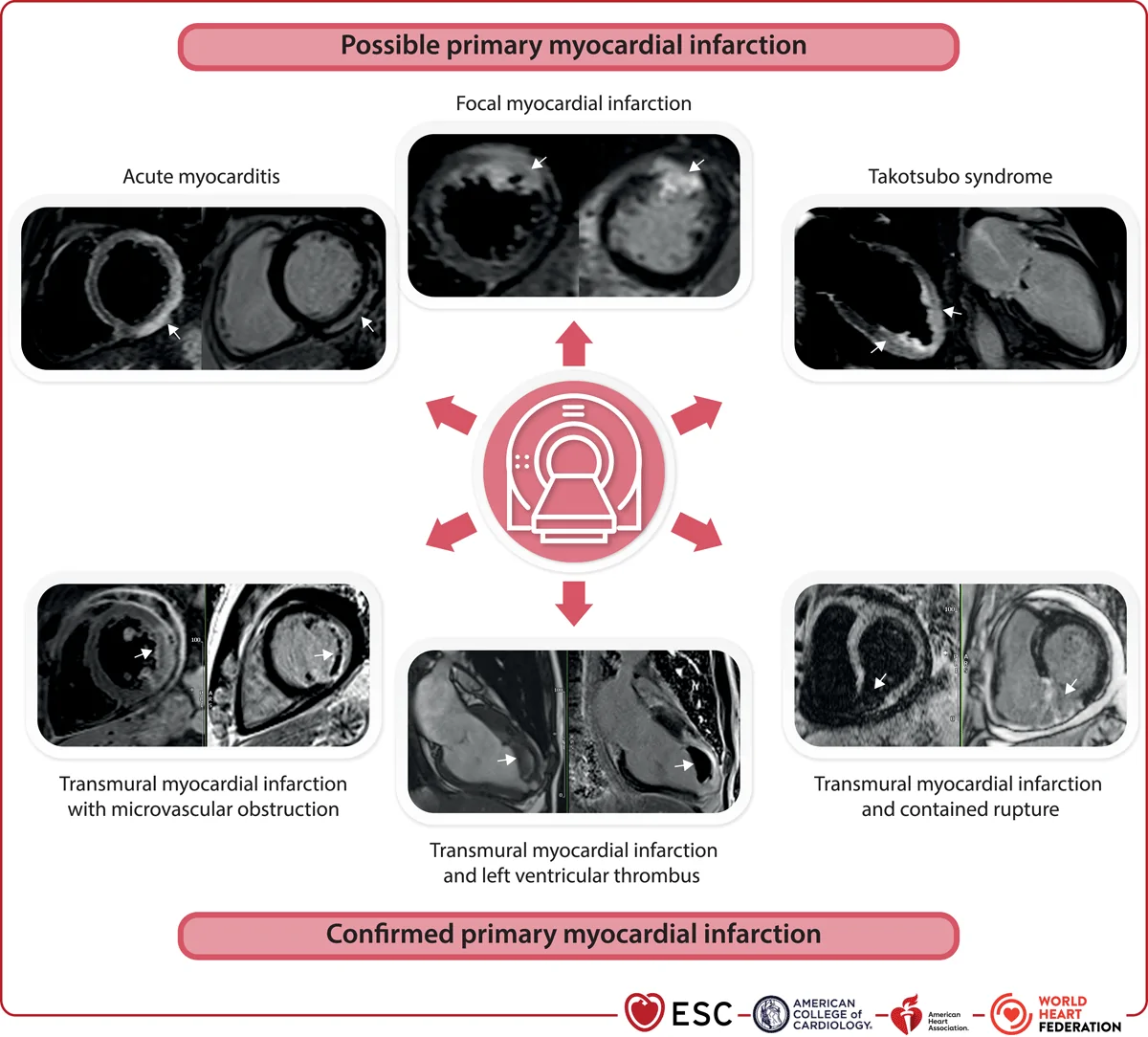
Role of cardiac magnetic resonance imaging in possible or confirmed primary myocardial infarction. Illustration of common cardiac magnetic resonance imaging findings in patients with possible or confirmed primary myocardial infarction. (*Top, left*) Acute myocarditis with myocardial oedema of the basal inferior and inferolateral wall (white arrow, left panel) with corresponding epicardial late enhancement (white arrow, right panel). (*Top, middle*) Focal myocardial infarction with transmural oedema of the mid anterolateral wall (white arrow, left panel) and corresponding transmural late enhancement (white arrow, right panel). (*Top, right*) Takotsubo syndrome with transmural oedema of the apical segments (white arrows, left panel), corresponding to the area of akinesia and in the absence of any late myocardial enhancement (right panel). (*Bottom, left*) Transmural myocardial infarction in the left circumflex territory with myocardial oedema of the lateral wall (white arrow, left panel) and corresponding transmural late enhancement with a large dark or low signal core representing microvascular obstruction (white arrow, right panel). (*Bottom, middle*) Transmural myocardial infarction in the left anterior descending artery territory with left ventricular thrombus (white arrow, both panels). (*Bottom, right*) Transmural myocardial infarction with contained rupture of the basal inferoseptal wall with myocardial oedema of the acutely injured wall (white arrow, left panel) and corresponding transmural late myocardial enhancement (white arrow, right panel).

In primary myocardial infarction, CMR identifies infarct extent as well as microvascular obstruction and intramyocardial haemorrhage (Figure 11) (231,232,233,234,235). CMR can identify complications, such as ventricular thrombus, papillary muscle infarction, right ventricular infarction, pseudoaneurysm formation (contained rupture), and pericardial inflammation, as well as assess viability for revascularization (Figure 12). CMR also aids diagnosis when coronary angiography does not identify an acute coronary pathology (see Section 6), helping to differentiate myocardial infarction from alternative diagnoses such as acute myocarditis or Takotsubo syndrome. CMR can complement echocardiography in detecting new loss of viable myocardium or regional wall motion abnormality in a pattern consistent with ischaemia (236). Similarly, in secondary or procedure-related myocardial infarction, CMR can identify new loss of viable myocardium or regional wall motion abnormality (4,72,74,187); however, image quality may be limited early after surgery due to artifact from chest drains or epicardial pacing wires (187,237).

#### 15.2.3. Coronary computed tomography angiography

CCTA is emerging as a valuable tool in the assessment of patients presenting with suspected ACS, offering sub-millimetre resolution to assess for presence of coronary atherosclerosis and plaque composition (238,239). Although current clinical guidelines do not recommend routine CCTA for suspected myocardial infarction in intermediate- and high-risk patients (1,2), it may be used to aid the diagnosis and has been found to offer prognostic information comparable to invasive coronary angiography in low-risk NSTEMI patients (240). CCTA may aid in differentiating primary from secondary myocardial infarction through plaque phenotyping (241,242) and help identify non-atherothrombotic causes for primary myocardial infarction, such as coronary artery dissection (although with limited resolution in smaller or more distal arteries) (243). In secondary myocardial infarction, CCTA may detect obstructive or flow-limiting coronary artery disease (74,75), and in procedure-related myocardial infarction, assess graft patency (244,245). Emerging applications include photon-counting CT or late contrast enhancement CT may identify loss of viable myocardium or detect alternative causes of myocardial injury, such as myocarditis, Takotsubo syndrome, or cardiomyopathy (246).

#### 15.2.4. Nuclear medicine

Nuclear medicine has advanced understanding of acute myocardial infarction but has limitations in clinical use. While SPECT and PET can assess myocardial blood flow and viability, their spatial resolution restricts detection to transmural, not subendocardial, infarcts (247,248,249). Co-registration with CT or CMR enhances resolution and enables molecular imaging. Experimental PET tracers combined with CCTA may identify atherothrombosis (250) and the source of thrombus due to coronary embolus in primary myocardial infarction (251).

## 16. Special situations

Diagnosing acute myocardial infarction can be challenging when symptoms, ECG, cardiac biomarkers, or wall motion abnormalities are difficult to interpret—such as in early recurrent myocardial infarction, critical illness, recent surgery, or structural interventions. Elevated cardiac biomarkers in heart failure, kidney disease, or cancer therapy may reflect non-ischaemic injury, often requiring careful evaluation and multimodality imaging to confirm the diagnosis.

### 16.1. Recurrent myocardial infarction

Diagnosing a new myocardial infarction within the first month of the index event may be challenging, as electrocardiographic changes, cardiac biomarkers, and imaging findings may reflect residual abnormalities (252,253,254,255). Recurrent symptoms are common (256), but may stem from complications of the index myocardial infarction, such as pericarditis, chest trauma after cardiopulmonary resuscitation, or heart failure, rather than recurrent myocardial infarction. Symptoms may differ from the index event, with ~10% of males and ~16% of females reporting different symptoms from their first versus their recurrent presentation (257). Persistent ST-segment elevation or T-wave inversion may be caused by ventricular aneurysm or pericarditis (258). Further, cardiac troponin levels often remain elevated for weeks, so serial measurements are key, as rising values suggest recurrence (258,259). The use of CK-MB adds cost without providing diagnostic clarity (44,260). Coronary angiography is often required to confirm recurrent myocardial infarction, and to differentiate primary from procedure-related myocardial infarction within 30 days (see Section 7). CMR may help to distinguish multiple infarcts, suggesting the presence of more than one culprit lesion or that the culprit was not correctly identified during the index presentation (261). Early recurrent myocardial infarction should still be classified according to the mechanism, rather than using a separate diagnostic code for recurrent myocardial infarction (Table 4).

### 16.2. Heart failure

Myocardial injury without infarction is common in acute and chronic heart failure due to systolic and diastolic dysfunction (262). Up to 90% of patients with acute heart failure have cardiac troponin T values above the URL (263), which may be misinterpreted as myocardial infarction due to overlapping symptoms (262,263). Cardiac troponin elevation can result from myocardial ischaemia, supply–demand mismatch due to hypoxia, hypotension or tachycardia, apoptosis and autophagy, and direct cellular injury due to infiltrative processes, inflammation, or neurohormones (264,265). Experimental transient increases in left ventricular preload have resulted in cardiac troponin elevation, apoptosis, and myocardial stunning in the absence of ischaemia (265), supporting preload-induced mechanical stretch as a possible mechanism for cardiac troponin release. Elevated cardiac troponins are of prognostic significance in acute and chronic heart failure (266,267) and are indicative of the severity of heart failure (268).

The spectrum of presentations with acute myocardial injury in heart failure includes primary or secondary myocardial infarction or non-ischaemic causes. In patients presenting with heart failure, chest discomfort, or equivalent symptoms with signs of myocardial ischaemia on the ECG, and/or known coronary artery disease, acute myocardial injury should raise the suspicion for primary myocardial infarction as the trigger for acute heart failure. Conditions that cause supply–demand imbalance, such as arrhythmias, hypoxia, hypotension, and anaemia, increase the likelihood of secondary myocardial infarction. Clinical practice guidelines recommend that patients presenting with new-onset or acute heart failure should have cardiac troponin measured and an ECG performed on admission to exclude acute myocardial infarction (269). Serial measurements can improve discrimination between acute and chronic myocardial injury (71). Imaging may be required to exclude the diagnosis of primary or secondary myocardial infarction. Both diagnoses require careful interpretation and comparison with prior imaging as CAD and regional wall motion abnormalities frequently exist in heart failure.

### 16.3. Chronic kidney disease

Chronic kidney disease (CKD) is common among patients with myocardial infarction, and outcomes are worse among those with both conditions (270,271). Patients with CKD have a higher incidence of myocardial infarction due to shared cardiovascular risk factors and a greater risk of secondary myocardial infarction from infection, anaemia, and haemodynamic stress associated with haemodialysis.

Cardiac troponin concentrations are frequently elevated but stable in CKD (272). Whether this reflects chronic myocardial injury due to coexisting cardiac conditions or reduced clearance due to impaired glomerular filtration, or both, is not always clear (273,274). CKD is often a consequence of diabetes mellitus or hypertension, which also increase the risk of coronary heart disease (275). The term cardiovascular–kidney–metabolic syndrome has emerged to reflect this shared pathophysiology (276). As such, elevated cardiac troponin concentrations in patients with CKD should not be dismissed as being a consequence of reduced clearance alone (277,278).

Diagnosing myocardial infarction in advanced CKD is challenging due to the frequent presence of elevated cardiac troponin concentrations. In consecutive patients with possible myocardial infarction, cardiac troponin was three times more likely to be elevated in those with CKD compared with those without for both cardiac troponin T (70% vs 24%) and cardiac troponin I (47% vs 16%) (272,279). While the final diagnosis is more likely to be primary myocardial infarction in those with CKD compared with those without (24% vs 11%), half of all patients with CKD and an elevated cardiac troponin concentration have myocardial injury without infarction (272). Therefore, serial cardiac troponin testing is particularly important to differentiate acute from chronic cardiac troponin elevations in CKD. The specificity and positive predictive value of the 99th percentile and high-risk thresholds are lower than among those without CKD (280,281). The use of higher thresholds for the diagnosis of myocardial infarction or greater absolute delta change values among those with CKD could improve the positive predictive value (279). However, these would be challenging to implement as the frequency of elevated cardiac troponin levels increases six-fold from 10% when the eGFR is 90 mL/min/1.73 m² or higher, to 66% when it falls below 30 mL/min/1.73 m² (272). Different thresholds would be required for each stage of CKD and for each assay. Adding to the challenge, patients with possible myocardial infarction and CKD are on average 20 years older than those without (279), which can also influence troponin thresholds. In the future, statistical models embedded into clinical decision support tools could address this complexity by integrating cardiac troponin and creatinine concentrations as continuous measures along with age, sex, and other comorbidities that influence cardiac troponin to estimate the probability of myocardial infarction (168). However, the pathways for diagnosis of myocardial infarction (whether primary, secondary, or procedure-related) in patients with CKD are the same as for those without (see Section 7.2).

Due to greater diagnostic uncertainty and risk in patients with CKD, imaging is important to confirm the diagnosis of myocardial infarction. Concerns about the nephrotoxic effects of contrast agents have contributed to underinvestigation and worse outcomes (282). Though the risk of contrast-induced nephropathy following coronary angiography in patients with CKD is often overstated (283), this risk, particularly in patients with advanced CKD, must be balanced against its potential benefits in guiding treatment.

### 16.4. Critical illness

Critical illness, characterized by vital organ dysfunction, places a significant burden on the cardiovascular system and often requires artificial organ function replacement, sedation, and vasopressors (284). Systemic stress responses can lead to destabilization of atherosclerotic plaques, resulting in primary myocardial infarction. More often, critical illness leads to acute myocardial injury or secondary myocardial infarction due to oxygen supply–demand imbalance, which can arise from impaired oxygenation, anaemia, impaired oxygen delivery, or a combination of these.

In analyses of discrepancies between clinical and post-mortem diagnoses in critically ill patients, myocardial infarction is among the most frequently missed diagnoses (285,286,287). Based on diagnostic criteria from previous UDMI classifications, an unrecognized myocardial infarction occurred in 1 in 4 critically ill patients with known cardiovascular disease and was associated with a lower likelihood of survival (288). Often it is not possible to determine whether patients are experiencing symptoms due to sedation. Even in those who can report chest pain or dyspnoea, these symptoms may be attributed to non-cardiac causes. Almost 40% of consecutive critically ill patients have myocardial injury and evidence of ischaemia on the ECG (289). ST-segment elevation is common in critically ill patients and less specific for primary myocardial infarction (290). Without troponin data, ECG interpretation in critically ill patients shows only moderate reliability (291). In critically ill patients with elevated cardiac troponin, a characteristic rise and fall pattern on serial testing is important to consider in the diagnosis of myocardial infarction (288,290,291). As with the diagnosis of myocardial infarction in other settings, confirmation (or exclusion) of the diagnosis requires coronary angiography and/or cardiac imaging (see Section 7.2.1 and Section 7.2.2). Left ventricular dysfunction occurs in ~25% of patients in intensive care (292) and is associated with increased mortality, but half of cases stem from non-cardiac causes and most abnormalities are reversible (293). While the overall incidence of Takotsubo syndrome in critical illness is low (around 1.5%) (294), there is some evidence that this is increasing in frequency (295), perhaps due to greater awareness. Coronary angiography may be needed to exclude myocardial infarction as a cause of regional left ventricular dysfunction.

Due to challenges in diagnosis and the high risk of primary and secondary myocardial infarction in critical illness, multidisciplinary care with cardiology input is essential. The risk of adverse outcomes from transportation of critically ill patients for coronary angiography and bleeding due to antithrombotic therapy needs careful consideration (296,297). Urgent coronary angiography may be necessary if primary myocardial infarction is likely or diagnosis will alter management. Otherwise, coronary and cardiac imaging to confirm or exclude a diagnosis of myocardial infarction may be deferred to prioritize stabilization of a critically ill patient.

### 16.5. Non-cardiac surgery

In patients undergoing non-cardiac surgery, myocardial infarction can result from a primary acute coronary pathology or be secondary to supply–demand mismatch. Any surgical procedure may increase the risk of myocardial infarction; however, the risk is greater for moderate- and high-risk surgeries, defined according to current guidelines (298), due to the greater chance for haemodynamic disturbance, blood loss, changes in body core temperature, and pro-inflammatory and prothrombotic changes (299).

Diagnosing myocardial infarction after non-cardiac surgery is challenging due to symptom masking by sedation and narcotics; most events occur within 2 days post-operatively, and up to two-thirds of patients have no ischaemic symptoms (299). For this reason, when clinical deterioration or acute myocardial injury is identified, an ECG is essential to aid the diagnosis, with coronary angiography and/or cardiac imaging (see Section 7.2.1 and Section 7.2.2) required to confirm the diagnosis of primary myocardial infarction; where ST-segment elevation is present, urgent coronary angiography is needed. In suspected secondary myocardial infarction, coronary or cardiac imaging is required to confirm the diagnosis, with timing based on its potential to impact peri-operative management. Post surgery, risks of anticoagulation and transport for coronary angiography require careful consideration. If active surveillance with cardiac troponin testing is performed, peri-operative myocardial injury (PMI) is identified in around 1 in 8 patients undergoing high-risk non-cardiac surgery (300). While the majority have peri-operative acute myocardial injury or secondary myocardial infarction due to supply–demand imbalance, a diagnosis of primary myocardial infarction occurs in 1 in 20 patients with myocardial injury in the post-operative setting (1 in 100 undergoing high-risk surgery overall).

The terms PMI and myocardial injury after non-cardiac surgery (MINS) have been used in this setting. PMI has been defined as acute myocardial injury with or without accompanying symptoms, or electrocardiographic or imaging evidence, of acute myocardial ischaemia (298). MINS has been defined as a subset of PMI, in patients in whom myocardial injury was deemed most likely due to myocardial ischaemia, in the absence of unexpected physiological stress from surgery or evidence of a cardiac non-coronary aetiology (76,298). MINS may occur with or without concomitant symptoms or signs of ischaemia and is associated with an eight-fold higher adjusted 30-day mortality (301,302). Identification of PMI or MINS should prompt an ECG, with further coronary and cardiac imaging required to confirm or exclude a diagnosis of primary or secondary myocardial infarction.

While clinical practice guidelines support routine post-operative cardiac troponin testing in high-risk patients or patients undergoing high-risk non-cardiac surgery (76,298,303), it is important to recognize that up to 40% of patients with cardiovascular disease or risk factors have baseline elevated cardiac troponin concentrations (304). For this reason, if assessment of cardiac troponin after surgery is planned, then a pre-operative value is essential in order to distinguish chronic myocardial injury from PMI or MINS.

### 16.6. Structural cardiac intervention

Structural cardiac interventions include a wide range of valve procedures, such as transcatheter aortic valve implantation (TAVI) and mitral transcatheter edge-to-edge repair (M-TEER), as well as septal/shunt closure and appendage occlusion devices. Myocardial infarction can result from several mechanisms, including supply–demand imbalance in the setting of procedure-related haemodynamic changes, obstruction of coronary ostia due to displacement of valve leaflets, and coronary embolism of device material, air, or thrombus.

Myocardial infarction complicates up to 5.6% of TAVI, though pooled estimates suggest 1.1% based on previous definitions (305). Distinguishing procedure-related myocardial infarction from acute myocardial injury is challenging. Myocardial infarction from leaflet-related coronary ostium obstruction is uncommon, occurring in 0.7% of patients undergoing TAVI, but mortality is high (306). Myocardial infarction due to embolism from the valve—often presenting with ST-segment elevation and hypotension—is infrequent, occurring in 1% of patients undergoing TAVI (307). New LGE in an ischaemic pattern was reported in as many as 18% of patients undergoing TAVI in whom CMR imaging was systematically performed (308). These lesions were typically small, multi-territorial, and unrelated to the presence of CAD, suggesting that procedure-related myocardial infarction due to embolism may be underrecognized.

Myocardial injury occurs in between 66% and 100% of patients following TAVI depending on the assay and definition used and whether transapical access was used (309,310,311,312). Myocardial injury was associated with short- and long-term mortality. Serial cardiac biomarker testing is important in patients with symptoms or signs of myocardial ischaemia or clinical deterioration following structural intervention, but cardiac biomarkers should not be used in isolation to define a procedure-related myocardial infarction. The diagnosis of procedure-related myocardial infarction following structural cardiac interventions should follow the criteria outlined above (see Section 7.2.3), with the diagnosis requiring imaging evidence of new loss of viable myocardium in an ischaemic pattern as a consequence of a procedural complication. This will facilitate comparisons between transcatheter and surgical approaches for the management of structural heart disease.

### 16.7. Cardio-oncology

Patients receiving chemotherapy, particularly with anthracyclines or immune checkpoint inhibitors, are at increased risk of cardiac complications, including myocarditis, cancer therapy-related cardiac dysfunction, and thromboembolism, which can mimic or trigger myocardial infarction (313). Cardiac troponin may rise from baseline or reflect new myocardial injury (314,315), so clinical guidelines recommend serial testing (316). Therefore, when assessing a patient who is receiving cancer-related therapies, cardiac troponin values are more likely to be elevated, and in the appropriate clinical setting (see Section 7.2.1 and Section 7.2.2) cardiac imaging is required to confirm or exclude myocardial infarction. Given that both cancer and CAD are common, atherothrombosis remains the most common cause of primary myocardial infarction, but coronary vasospasm and embolism are more common in cancer due to the pro-inflammatory and hypercoagulability state of some cancers and the effects of chemotherapeutic agents (317).

## 17. Implications for patients, secondary prevention, and cardiac rehabilitation

Accurate diagnosis of myocardial infarction is important due to its significant prognostic, psychological, social, emotional, financial, insurance, and occupational impacts on patients and their families (318). Following myocardial infarction, patients often report persistent symptoms such as fatigue, chest pain, low physical or mental energy, and reduced motivation that impact quality of life and well-being (318,319,320). Anxiety and depression are common, particularly among those with a prior history of depression, poor health, low socioeconomic status, and younger age at the time of diagnosis (320,321). Not all individuals perceive their recovery from myocardial infarction negatively; however, a small number of studies indicate that a myocardial infarction resulted in gratitude for one’s life (322), hope for the future (323), and having a second chance at life (324). Shared decision-making involving personalized discussions with patients, families, and clinicians is essential to incorporate values and concerns into diagnostic and treatment choices.

A diagnosis of myocardial infarction has lifelong treatment implications (325) and may lead to financial burdens, including impacts on insurance and health and travel coverage. For patients living in high-income countries who are employed prior to myocardial infarction, most will return to work within one year, particularly if they are well-educated with a higher salary (326). Those with heart failure, diabetes, or depression are less likely to return to work, and regulations for occupations that require driving or flying may preclude some. Patients who return to work have a better psychological status following a diagnosis of myocardial infarction (327), with evidence that encouragement from clinicians increases the likelihood (326,327,328). Conversations with patients could include probing the consequences of a diagnosis of myocardial infarction on them personally with regards to their employment, relationships, or finances.

When there is diagnostic uncertainty between clinicians, patient outcomes have been found to be worse (329). By simplifying the classification into primary, secondary, and procedure-related myocardial infarction, we aim to make the diagnosis clearer and more understandable for both patients and clinicians. This in turn may facilitate tailoring of educational materials to patients who experience myocardial infarction. Cardiac rehabilitation has demonstrated efficacy for people with myocardial infarction in clinical trials and is recommended by clinical practice guidelines. Cardiac rehabilitation is not currently tailored to the setting in which myocardial infarction arises and is less often offered to those with secondary myocardial infarction, where arguably the role for education is more important as patients are often managed outside specialist cardiac units (330).

Survey data from Europe suggest that 90% of patients feel well informed about the role of secondary prevention following a diagnosis of primary myocardial infarction, but only 1 in 3 and 1 in 10 were aware of the targets for blood pressure and cholesterol, respectively (331). Similar data from low- and middle-income countries (LMICs) suggest that less than half of patients in parts of Southern Africa are aware of secondary prevention targets (332). No data are available for people with secondary myocardial infarction, perhaps because the role of CAD in the pathophysiology of the previous classification was uncertain, and therefore, the importance of risk factor modification was less clear. The use of diagnostic criteria for secondary myocardial infarction that include CAD may encourage inclusion of these patients in cardiac rehabilitation and education programmes. This is important as patient education improves engagement in cardiac rehabilitation, medication use, lifestyle choices, and understanding of symptoms (333,334), which could reduce delays in presentation among those with recurrent events (335,336). Clear diagnostic criteria for myocardial infarction may also help with strategies to improve referrals for rehabilitation, such as automatic referral systems, which in turn could improve participation.

## 18. Implications for public health and classification of disease

Use of high-sensitivity cardiac troponin assays has increased the recognition of myocardial injury and, together with adoption of the Fourth UDMI, has led to an increase in the diagnoses of type 1 and type 2 myocardial infarction by 11% and 22%, respectively (337). However, as the Fourth UDMI was not aligned with the ICD-10 edition codes, this increase has not been reflected in morbidity and mortality data (338). For example, across two countries in Europe, fewer than 20% of patients who met the diagnostic criteria for type 2 myocardial infarction according to the Fourth UDMI received an ICD-10 code for myocardial infarction (40). The increased recognition of myocardial injury has further presented health system challenges when myocardial infarction has been incorrectly assigned to patients with non-ischaemic causes of acute myocardial injury (73).

Working together with the World Health Organization, the Fifth UDMI aims to address these challenges by including distinct ICD-11 codes for primary, secondary, and procedure-related myocardial infarction (Table 4). For the first time, the ICD-11 code for acute myocardial infarction differentiates STEMI (BA41.0) from NSTEMI (BA41.1) and additional sixth digit stem codes have been incorporated to identify the underlying acute coronary pathology in primary myocardial infarction (1 to 9) and to differentiate primary from secondary (A) and procedure-related (B or C) myocardial infarction.

When primary myocardial infarction is considered to be the most likely diagnosis, but the acute coronary pathology remains uncertain following coronary angiography or cardiac imaging, or these investigations are not available or considered appropriate, specific stem codes are applied. In this scenario, BA41.0 or BA41.1 differentiates ST-segment elevation from non-ST-segment elevation myocardial infarction, respectively, with sixth digit stem codes applied for primary myocardial infarction of undetermined aetiology following coronary imaging or for unknown aetiology (no coronary imaging).

For secondary myocardial infarction, the structure of ICD-11 enables incorporation of associated diagnostic codes to indicate the acute alternative condition responsible for supply–demand mismatch to enable a better understanding of the risk of secondary myocardial infarction associated with common presentations. For procedure-related myocardial infarction, associated diagnostic codes differentiate percutaneous interventional (BA41.0B and BA41.1B) and open cardiac surgical (BA41.0C and BA41.1C) procedures. Finally, the ICD-11 code for unrecognized myocardial infarction (BA50) can be applied to better understand the frequency of this diagnosis and implications for patients.

While the implementation of ICD-11 coding across all countries will take time, it will enable epidemiological research, healthcare system planning, and public health monitoring of each type of myocardial infarction. The use of ICD-11 codes for STEMI would aid health service planning for delivery of immediate reperfusion therapy, while implementation of sixth digit stem codes to identify the acute coronary pathology will provide insights into the incidence of the less common mechanisms of primary myocardial infarction: SCAD, vasospasm, embolism, restenosis, stent thrombosis, or graft failure. In turn this will facilitate national and international comparisons of care and outcomes for these distinct conditions using routinely collected healthcare data. For successful implementation, healthcare providers—including clinicians and hospital coders—will need training on the new clinical classification of the Fifth UDMI and the associated ICD-11 codes.

## 19. Implications and adaptions for low-resource settings

In 2021, there were 31.9 million new cases of ischaemic heart disease globally—31.8% more than in 2010 (339), driven mainly by ageing populations, urbanization, and rising risk factors of smoking, physical inactivity, obesity, and diabetes in LMICs (340,341). People in low-resource settings, within or outside of LMICs, face many challenges in the diagnosis of myocardial infarction. This can be related to socioeconomic status, education and awareness, race and ethnic differences, as well as lack of timely access to emergency services, diagnostic testing, and treatment (342). While clinical practice guidelines include access to high-sensitivity cardiac troponin testing as one of the 21 quality measures (343), in many LMICs there is limited availability (9). Guidance on the use of alternative biomarkers of myocardial injury for settings where high-sensitivity cardiac troponin testing is not available is found in Section 14.2. This further extends to electrocardiography, coronary angiography, and cardiac imaging, impacting the ability to diagnose myocardial infarction. For example, a study from Uganda reported that only 43% of healthcare facilities had access to troponin testing and 55% had access to electrocardiography (339,341,342,344). In China, the proportion of hospitals able to measure cardiac troponin increased from 25% to 84% between 2001 and 2011 (345), while in India, coordinated strategies have been made to improve emergency ambulance services with pre-hospital electrocardiography (346). In the Eastern Mediterranean region, lack of access to cardiac catheterization affected the diagnosis of myocardial infarction (347). However, even where diagnostic testing is available, the affordability of these tests, which are often paid out of pocket in many LMICs, can further limit access (348,349,350).

Solutions include improved access to affordable point-of-care troponin testing, use of telemedicine for interpretation of the ECG, and widespread education to raise awareness (9). In 2023, the United Nations high-level meeting on universal health coverage for non-communicable diseases focused on a framework to achieve this by 2030, with global commitments to translate these into concrete actions. Basic diagnostic and laboratory equipment for the diagnosis of acute myocardial infarction in urgent care and emergency care facilities should be available to all. However, until such time, clinicians working in resource-limited settings still need to be able to make a diagnosis of myocardial infarction in the absence of cardiac biomarker testing or cardiac imaging (Box 7) (351).

**Box 7 Diagnostic criteria for myocardial infarction in low-resource settings where biomarker testing and cardiac imaging are not available**

The diagnosis is **presumed** in those with symptoms consistent with acute myocardial ischaemia where one or more clinical features are present:

- New ischaemic changes on the electrocardiogram
- Development of pathological Q waves.

## 20. Implications of the definition on research

The previous classification of myocardial infarction was not always consistently applied in research, particularly in the evaluation of accelerated diagnostic pathways and in clinical trials of medical therapy and revascularization (352,353,354,355,356,357,358). The inclusion of all acute coronary mechanisms in the classification of primary myocardial infarction aims to improve the standardization of studies evaluating the performance of diagnostic pathways. Emerging artificial intelligence (AI) tools show promise in automating the interpretation of cardiac biomarkers and ECGs, enhancing the detection of ACS and supporting early myocardial infarction diagnoses (359,360,361). Machine-learning approaches are also providing new insights into the pathophysiological features of coronary atherosclerosis, which may ultimately guide personalized treatment and patient selection for invasive coronary angiography (362). Further research is needed to determine if these tools, when used with appropriate clinician oversight, can improve diagnostic sensitivity, expedite decision-making, reduce diagnostic errors, and improve patient outcomes.

Many trials of therapy or intervention included patients with presumed primary myocardial infarction without confirming the diagnosis by coronary angiography, potentially enrolling those without true myocardial infarction, reducing the observed effectiveness of the intervention (355). Routine classification of primary myocardial infarction according to the acute coronary pathology (e.g. SCAD or coronary vasospasm) will enable the study of outcomes and support trials of tailored treatment pathways, which is particularly important for those pathologies where management may differ from atherothrombosis (363). Serial cardiac troponin measurements and cardiac imaging following reperfusion may improve staging of the severity of tissue injury in primary myocardial infarction (232), improving patient selection for trials targeting events post infarction.

Embedding the UDMI classification with the associated ICD-11 codes into registries and electronic health records will facilitate enrolment and follow-up of patients with primary, secondary, and procedure-related myocardial infarction in registry-based or routine data-enabled clinical trials. Despite being common and linked to poorer outcomes, only one adequately powered randomized controlled trial has been conducted in patients with myocardial infarction and anaemia (352). This study compared liberal vs restrictive transfusion strategies and found no difference in death or recurrent infarction at 30 days. Subgroup analyses were consistent with the overall trial for those with type 2 myocardial infarction, who were expected to have the most to gain from liberal transfusion (364). These results underscore the need for clearer, objective diagnostic criteria for secondary myocardial infarction to ensure the enrolment of patients who are likely to benefit from interventions. Such criteria will facilitate enrolment into future studies that can more rigorously evaluate targeted therapies in this group of patients.

The lack of consensus between interventional and surgical groups on defining procedure-related myocardial infarction has hindered trial interpretation for decades. *Post hoc* analyses demonstrate that varying criteria to define procedure-related myocardial infarction significantly affect reported rates of myocardial infarction across studies (48,365,366). This update to the UDMI aims to reduce variation between the definition of procedure-related myocardial infarction following percutaneous intervention and cardiac surgery to enable more reliable comparisons. Further research is needed to validate this approach and determine if the prognostic implications of procedure-related myocardial infarction are similar in both settings, which in turn will guide patient-centred clinical decision-making.

One of the objectives of this update in the classification is to improve consistency in the application of the diagnosis of myocardial infarction in clinical practice and clinical research so that the findings from research can be readily adopted into practice, allowing for improved epidemiological research and between-country comparisons. However, in recognition that there are areas in which uncertainty remains, suggestions for further research are provided (Box 8).

**Box 8 Recommendations for further research**

**Research to encourage adoption:**

- Evaluate the effects of implementation of the UDMI on the incidence of myocardial infarction across different healthcare systems and in different parts of the world.
- Evaluate the impact of implementation on the use of cardiac imaging in possible primary, secondary, and procedure-related myocardial infarction.
- Compare treatment and outcomes among patients with primary myocardial infarction stratified according to the underlying coronary aetiology.
- Evaluate the impact of applying new diagnostic criteria for secondary myocardial infarction on treatment, management, and outcomes.
- Understand the prognostic implications and impact on health-related quality of life following procedure-related myocardial infarction and compare outcomes of coronary intervention with cardiac surgery.

**Research to generate new knowledge in electrocardiography and biomarkers for myocardial infarction diagnosis:**

- Determine whether strategies incorporating AI and machine learning (e.g. for electrocardiography and cardiac biomarker interpretation) can improve the accuracy and timeliness of myocardial infarction diagnosis.
- Improve our understanding of sex differences in the pathophysiology of myocardial infarction and the impact of sex-specific thresholds for cardiac troponin on investigation, treatment, and outcomes.
- Develop new approaches to define URLs for cardiac troponin with manufacturers and regulators that consider age, sex, and renal function so those that improve the recognition of myocardial injury due to myocardial infarction and/or other cardiac conditions in younger and older patients could be applied in practice.
- Determine the clinical and cost effectiveness of near-patient testing with point-of-care high-sensitivity cardiac troponin assays in the community, ambulance, and hospital settings.
- Determine whether routine cardiac troponin measurement following cardiac procedures improves the recognition of procedure-related myocardial infarction and outcomes.
- Evaluate the performance of sex-specific and uniform cardiac troponin thresholds (multiples of assay-specific URLs) to identify patients with a complication following a cardiac procedure who are unlikely to have a procedure-related myocardial infarction.
- Evaluate the utility of relative and absolute, assay-specific criteria for the recognition of chronic myocardial injury due to a cardiac condition.

**Research to generate new knowledge in imaging for myocardial infarction diagnosis:**

- Compare the diagnostic performance of echocardiography and cardiac magnetic resonance imaging in patients with possible secondary and procedure-related myocardial infarction.
- Determine the utility of CT coronary angiography with and without plaque phenotyping in patients with possible primary, secondary, and procedure-related myocardial infarction.
- Determine the utility of photon-counting CT coronary angiography with late iodine enhancement in possible myocardial infarction to identify acute coronary pathology and loss of viable myocardium.
- Determine if invasive physiological testing can improve diagnostic accuracy or outcomes in possible secondary myocardial infarction.
- Determine the diagnostic utility of cardiac imaging to investigate chronic myocardial injury in those without established cardiovascular disease.

## Additional Files

The additional files for this article can be found as follows:

10.5334/gh.1578.s1 Supplementary Data 1. Evidence Tables.

10.5334/gh.1578.s2 Supplementary Data 2. ESC Declaration of Interests Report.

## Data Availability

No new data were generated or analysed in support of this research.

## Appendix

Approved by the ESC Clinical Practice Guidelines Committee on behalf of the ESC Board,

Approved by the ACC Presidential Team,

Approved by the AHA Executive Committee,

Approved by the WHF Executive Committee on behalf of the WHF Board, with recommendation from the Science Committee.

**ESC/ACC/AHA/WHF Universal Definition Document Advisory Group** includes Document Reviewers, ESC/ACC/AHA/WHF Committee Members, and ESC National Cardiac Societies.

**Document Reviewers:** Borja Ibanez (ESC Review Co-ordinator) (Spain), Elliott Antman (ACC/AHA Review Co-ordinator) (United States of America), Junbo Ge (WHF Review Co-ordinator) (China), Tayo Addo (United States of America), Junya Ako (Japan), Mirvat Alasnag (Saudi Arabia), Rasha Al-Lamee (United Kingdom), Paulo R. A. Caramori (Brazil), Mauricio G. Cohen (United States of America), Victoria Delgado (Spain), Bernard Dennis (United States of America), Sigrun Halvorsen (Norway), Julie Harris (United Kingdom), Milosz J. Jaguszewski (Poland), Hani Jneid (United States of America), Hugo Katus (Germany), Milan Milojevic^1^ (Serbia/Switzerland), Michelle O’Donoghue (United States of America), Julie Redfern (Australia), Joseph F. Sabik III^2^(United States of America), Clara Saldarriaga (Colombia), Nizal Sarrafzadegan (Iran/Canada).

ESC CPG Committee members and WHF representatives who participated in the review of the Fifth Universal Definition of Myocardial Infarction:

Marianna Adamo (Italy), Suleman Aktaa (United Kingdom), Folkert W. Asselbergs (Netherlands), Michael A. Borger (Germany), Giuseppe Boriani (Italy), Margarita Brida (Croatia), Robert A. Byrne (Ireland), Estelle Gandjbakhch (France), Bettina Heidecker (Germany), Anja Hennemuth (Germany), Borja Ibanez (Spain), Stefan James (Sweden), Ulf Landmesser (Germany), Gregory Y.H. Lip (United Kingdom), John William McEvoy (Ireland), Borislava Mihaylova (United Kingdom), Inge Moelgaard (Denmark), Jagat Narula (United States of America), Lis Neubeck (United Kingdom), Eva Prescott (Denmark), Bianca Rocca (Italy), Xavier Rossello (Spain), Anna Sannino (Germany), Felix C. Tanner (Switzerland), Katja Zeppenfeld (Netherlands).

**ESC National Cardiac Societies** actively involved in the review process of the Fifth Universal Definition of Myocardial Infarction:

**Albania:** Albanian Society of Cardiology, Naltin Shuka; **Algeria:** Algerian Society of Cardiology, Mohammed Chettibi; **Armenia:** Armenian Cardiologists Association, Hamlet G. Hayrapetyan; **Austria:** Austrian Society of Cardiology, Georg Delle Karth; **Azerbaijan:** Azerbaijan Society of Cardiology, Abbasali Abbasaliyev; **Belgium:** Belgian Society of Cardiology, Bernhard Gerber; **Bosnia and Herzegovina:** Association of Cardiologists in Bosnia and Herzegovina, Azra Durak Nalbantic; **Bulgaria:** Bulgarian Society of Cardiology, Elina Georgieva Baltadzhieva-Trendafilova; **Croatia:** Croatian Cardiac Society, Bosko Skoric; **Cyprus:** Cyprus Society of Cardiology, Kimon Myrianthopoulos; **Czechia:** Czech Society of Cardiology, Petr Ostadal; **Denmark:** Danish Society of Cardiology, Christian Juhl Terkelsen; **Egypt:** Egyptian Society of Cardiology, Moustafa Mokarrab; **Estonia:** Estonian Society of Cardiology, Toomas Marandi; **Finland:** Finnish Cardiac Society, Pekka Porela; **France:** French Society of Cardiology, Eric Van Belle; **Georgia:** Georgian Society of Cardiology, Zviad Kereselidze; **Germany:** German Cardiac Society, Tau Hartikainen; **Greece:** Hellenic Society of Cardiology, Konstantinos Toutouzas; **Hungary:** Hungarian Society of Cardiology, Istvan Ferenc Edes; **Iceland:** Icelandic Society of Cardiology, Geir Hirlekar; **Ireland:** Irish Cardiac Society, James O’Neill; **Israel:** Israel Heart Society, David Hasdai; **Italy:** Italian Federation of Cardiology, Giuseppe Patti; **Kazakhstan:** Association of Cardiologists of Kazakhstan, Salim Berkinbayev; **Kosovo (Republic of):** Kosovo Society of Cardiology, Arlind Batalli; **Kyrgyzstan:** Kyrgyz Society of Cardiology, Olga Lunegova, **Latvia:** Latvian Society of Cardiology, Sanda Jegere; **Lebanon:** Lebanese Society of Cardiology, Ahmad Nabih Serhal; **Libya:** Libyan Cardiac Society, Aiman Smer; **Lithuania:** Lithuanian Society of Cardiology, Olivija Dobiliene; **Luxembourg:** Luxembourg Society of Cardiology, Jean Beissel; **Malta:** Maltese Cardiac Society, Matthew Mercieca Balbi; **Moldova (Republic of):** Moldavian Society of Cardiology, Aurel Andrei Grosu; **Morocco:** Moroccan Society of Cardiology, Samir S. Ztot; **Netherlands:** Netherlands Society of Cardiology, Roberto Diletti; **North Macedonia**: National Society of Cardiology of North Macedonia, Danica Petkoska Spirova; **Norway:** Norwegian Society of Cardiology, Kristin Angel; **Poland:** Polish Cardiac Society, Mariusz Tomaniak; **Portugal:** Portuguese Society of Cardiology, Rita Calé Theotónio; **Romania:** Romanian Society of Cardiology, Razvan Ilie Radu; **San Marino:** San Marino Society of Cardiology, Marco Zavatta; **Slovakia:** Slovak Society of Cardiology, Martin Studencan; **Slovenia:** Slovenian Society of Cardiology, Matjaz Bunc; **Spain:** Spanish Society of Cardiology, Rut Andrea Riba; **Sweden:** Swedish Society of Cardiology, Erik Östgärd Thunström; **Switzerland:** Swiss Society of Cardiology, Juan F. Iglesias; **Syrian Arab Republic:** Syrian Cardiovascular Association, Mohammed Yassin Bani Marjeh; **Tunisia:** Tunisian Society of Cardiology and Cardiovascular Surgery, Ben El Hadj Zied; **Türkiye:** Turkish Society of Cardiology, Ertugrul Okuyan; **Turkmenistan:** Turkmen Committee of Cardiologists, Bahram Kadyrov**; Ukraine:** Ukrainian Association of Cardiology, Alexander Parkhomenko; **United Kingdom of Great Britain and Northern Ireland:** British Cardiovascular Society, David Hildick-Smith, **Uzbekistan:** Association of Cardiologists of Uzbekistan, Saodat Abdullaeva. Contributor either withdrew or was engaged in only a part of the review process: Newton Sigrist (Australia).

## Disclaimer

This ESC/ACC/AHA/WHF Universal Definition Document represents the views of the ESC, ACC, AHA, and WHF and was produced after careful consideration of the scientific and medical knowledge and the available evidence at the time of its development. The ESC, ACC, AHA, and WHF are not responsible in the event of any contradiction, discrepancy, and/or ambiguity between the ESC/ACC/AHA/WHF Universal Definition Document and any other official recommendations or Expert Consensus Document issued by the relevant public health authorities, in particular in relation to good use of healthcare or therapeutic strategies. Health professionals are encouraged to take the ESC/ACC/AHA/WHF Universal Definition Document fully into account when exercising their clinical judgment, as well as in the determination and the implementation of preventive, diagnostic, or therapeutic medical strategies; however, the ESC/ACC/AHA/WHF Universal Definition Document does not override, in any way whatsoever, the individual responsibility of health professionals to make appropriate and accurate decisions in consideration of each patient’s health condition and in consultation with that patient and, where appropriate and/or necessary, the patient’s caregiver. Nor does the ESC/ACC/AHA/WHF Universal Definition Document exempt health professionals from taking into full and careful consideration the relevant official updated recommendations, Universal Definition Documents or Expert Consensus Documents issued by the competent public health authorities, in order to manage each patient’s case in light of the scientifically accepted data pursuant to their respective ethical and professional obligations. It is also the health professional’s responsibility to verify the applicable rules and regulations relating to drugs and medical devices prior to making any clinical decision and to check whether a more recent version of this document exists. The ESC, ACC, AHA, and WHF warn readers that the technical language may be misinterpreted and declines any responsibility in this respect.

## Permissions

The content of this ESC/ACC/AHA/WHF Universal Definition Document has been published for personal and educational use only. No commercial use is authorized. No part of the ESC/ACC/AHA/WHF Universal Definition Document may be translated or reproduced in any form without written permission from the ESC, ACC, AHA, WHF. Permissions can be obtained upon submission of a written request to Oxford University Press, the publisher of the *European Heart Journal* and the party authorized to handle such permissions on behalf of the ESC, ACC, AHA, and the WHF (journals.permissions@oup.com).
