# Supplementary material for: Fifth Universal Definition of Myocardial Infarction (2026): On behalf of the Joint European Society of Cardiology (ESC)/American College of Cardiology (ACC)/American Heart Association (AHA)/World Heart Federation (WHF) Task Force for the Universal Definition of Myocardial Infarction: Endorsed by the European Association for Cardio-Thoracic Surgery (EACTS) and the Society of Thoracic Surgeons (STS): Affirmation of Value by the Society for Cardiovascular Angiography and Interventions (SCAI)
Source: Glob Heart. 2026 Aug 28;21(1):64. doi: 10.5334/gh.1578 (PMC13523810; doi:10.5334/gh.1578)
Supplement: Supplementary file 1 [file gh-21-1-1578-s1.pdf]

Fifth Universal Definition of Myocardial Infarction (2026)

EVIDENCE TABLES

developed by the Joint European Society of Cardiology (ESC)/American College of Cardiology (ACC)/American Heart Association (AHA)/World Heart Federation (WHF) Task Force for the Universal Definition of Myocardial Infarction

Table of contents

Abbreviations 3
1. Evidence tables for sections 1 to 11 6
2. Evidence tables for section 14 76
3. Evidence tables for sections 12, 13, and 15 126
4. Evidence tables for section 16 183
5. Evidence tables for sections 17 to 20 239

Abbreviations

Abbreviation	
ACC	American College of Cardiology
ACS	Acute coronary syndrome
AF	Atrial fibrillation
AMI	Acute myocardial infarction
AUC	Area under the [ROC] curve
AVR	Aortic valve replacement
cTn	Cardiac troponin
CABG	Coronary artery bypass grafting
CAD	Coronary artery disease
CCS	Canadian Cardiovascular Society
CHD	Coronary heart disease
CI	Confidence interval
CKD	Chronic kidney disease
CK-MB	Creatine kinase-muscle/brain isoenzyme
CMR	Cardiac magnetic resonance
cMyC	Cardiac myosin-binding protein-C
CT	Computed tomography
CVD	Cardiovascular disease
DCB	Drug-coated balloon
ECG	Electrocardiogram
ED	Emergency Department
eGFR	Estimated glomerular filtration rate
ESC	European Society of Cardiology

FMC	First medical contact
hs-cTn	High-sensitivity cardiac troponin
HR	Hazard ratio
HF	Heart failure
ICU	Intensive care unit
ITT	Intention to treat
LBBB	Left bundle branch block
LGE	Late gadolinium enhancement
LMICs	Low- and middle-income countries
LV	Left ventricular
LVEF	Left ventricular ejection fraction
MI	Myocardial infarction
MACE	Major adverse cardiovascular events
MINOCA	Myocardial infarction with no obstructive coronary arteries
NPV	Negative predictive value
NSTEMI	Non-ST-segment elevation myocardial infarction
OHCA	Out-of-hospital cardiac arrest
OR	Odds ratio
PCI	Percutaneous coronary intervention
PMI	Peri-procedural myocardial injury
PPV	Positive predictive value
PROMS	Patient-reported outcomes measures
RCT	Randomized controlled trial
ROC	Receive operating characteristic
SCAD	Spontaneous coronary artery dissection

STEMI	ST-segment elevation myocardial infarction
TAVR	Transcatheter aortic valve replacement
TIMI	Thrombolysis in Myocardial Infarction
T2MI	Type 2 myocardial infarction
TTE	Transthoracic echocardiography
UDMI	Universal Definition of Myocardial Infarction
ULN	Upper limit of normal
URL	Upper reference limit
VARC	Valve Academic Research Consortium

1. Evidence tables for sections 1 to 11

**Sections**	Sections 1 to 11 (Diagnosis and classification of myocardial infarction)								
**META-ANALYSES**									
**Study first author surname, year, and acronym**	**PMID reference for study**	**Population**	**Number of participants or studies**	**Intervention**	**Key inclusion & exclusion criteria**	**Type of analysis** (random-effect, fixed-effect, Bayesian approach)	**Relevant outcome(s) and PROMS**	**Other methodological aspects**	**Key findings and interpretation**
Boeddinghaus, 2025	n/a	Patients with suspected acute coronary syndrome (ACS)	*N* = 120 734 patients with suspected ACS from 17 studies (nine cohorts from nine countries)	Stratification according to the classification proposed by the Fourth Universal Definition of Myocardial Infarction (UDMI)	Studies were included if they met the following criteria: (1) recruited patients who underwent cardiac troponin (cTn) testing for suspected ACS following presentation to the emergency department (ED) or in hospital; (2) index diagnosis adjudicated in accordance with	Systematic search to identify prospective studies where cTn was measured for suspected ACS; diagnoses were adjudicated using the UDMI and cause-specific mortality was reported at a minimum of 1 year. Meta-analysis was performed with random-effects	The primary outcome was major adverse cardiovascular events (MACE) defined as myocardial infarction (MI) or cardiovascular death. The secondary outcome was non-cardiovascular death.	Subdistribution hazard ratios (sHR) were derived to account for the competing risk of non-cardiovascular death	Type 1 MI occurred in 9.4%, type 2 MI in 3.0%, acute myocardial injury in 4.9%, and chronic myocardial injury in 4.7%. In patients with type 1 and type 2 MI, the MACE rate was 55.2 and 51.7 per 1000 patient-years, and the non-cardiovascular death rate was 25.7 and 60.1 per 1000 patient-years,

					the Universal Definition; and (3) both MI and cause-specific mortality reported at a minimum of 1 year	models.			respectively. Compared with those without myocardial injury, the risk of MACE (sHR) was 4.82 (95% confidence interval [CI] 3.55–6.57), and 3.36 (95% CI 2.92–3.86), respectively. In patients with acute and chronic myocardial injury, the MACE rate was 47.1 and 44.9 per 1000 patient-years, and the non-cardiovascular death rate was 67.0 and 46.9 per 1000 patient-years, respectively. The risk of MACE was 3.24 (95% CI 2.41–4.36) and 3.03 (95% CI 2.53–3.62), respectively.

									All patients with myocardial injury and infarction are at increased risk of future cardiovascular events. Although patients with type 1 and type 2 MI have a similar event rate, cardiovascular risk in type 2 MI is attenuated by a substantial competing risk of non-cardiovascular death.
Chapman, 2017	29127948	Patients with suspected ACS	22 475<br><br>22 cohort studies	High-sensitivity cardiac troponin (hs-cTn) I assay	22 observational cohort studies were included<br><br>Inclusion criteria:<br>• Patients with suspected ACS<br>• hs-cTn I (Abbott) at	Meta-estimates derived by modelling cohort-level proportions in a binomial-normal random-effects model. Odds ratios (ORs) and 95% CIs were used to evaluate	Primary outcome type 1 MI or cardiac death at 30 days.<br><br>Key secondary endpoints were recurrent MI and cardiac death.		Among patients with suspected ACS, a low hs-cTn I concentration identified those at low risk of MI or cardiac death within 30 days

					presentation, adjudicated diagnosis<br>Exclusion criteria:<br>• ST-segment elevation MI (STEMI)	differences in negative predictive value (NPV) by subgroups. Heterogeneity was assessed with the I^2 index.			
Mileva, 2023	36889851	Patients with myocardial infarction and unobstructed coronary arteries (MINOCA)	26 studies comprising 3624 patients	Determine the diagnostic and prognostic value of cardiac magnetic resonance (CMR) in the management of patients with MINOCA	The inclusion criteria were as follows: 1) studies comprising patients with a working diagnosis of MINOCA; 2) undergoing a CMR assessment within 10 days from the index acute event; and 3) reporting the number of patients	PRISMA (Preferred Reporting Items for Systematic reviews and Meta-Analyses).<br>QUADAS (Quality Assessment of Diagnostic Accuracy Studies).<br>Random-effects model.	The primary outcome of interest was the prevalence of confirmed MI, myocarditis, Takotsubo syndrome, or normal findings according to CMR imaging in patients with an initial working diagnosis of MINOCA		MINOCA was confirmed in only 22% (95% CI 0.17–0.26) of the cases and 68% of patients with initial MINOCA were reclassified after the CMR assessment. The pooled prevalence of myocarditis was 31% (95% CI 0.25–0.39), and Takotsubo syndrome 10% (95% CI 0.06–0.12).

					diagnosed with non-specific disease entity and the total number of patients evaluated				
Khan, 2017	29020244	Patients with non-ST-segment elevation myocardial infarction (NSTEMI)	*N* = 10 415, 7 studies	Estimate the difference in outcomes of patients with NSTEMI with and without culprit artery total occlusion	Patients with NSTEMI	Meta-analysis with generic inverse or Mantel–Haenszel method to pool relevant outcomes and the mean difference or relative risk calculated	All-cause mortality, MACE		25.5% of patients with NSTEMI had an occluded culprit artery, associated with increased risk of MACE (relative risk [RR] 1.41, 95% CI 1.17–1.70) and all-cause mortality (RR 1.67, CI 1.31–2.13)
**RANDOMIZED CONTROLLED TRIALS**									
**Study first author surname, year, and acronym**	**PMID reference for study**	**Population**	**Number of participants**	**Intervention and control**	**Key inclusion & exclusion criteria**	**Type of analysis** (intention-to-treat [ITT], per-protocol [PP], ITT or PP with adjustment	**Relevant outcome(s) and PROMS**	**Other methodological aspects** (allocation concealment, single/double	**Key findings and interpretation**

						for pre-specified covariates)		blinding, sham procedure in device trials, missing data on outcomes, outcome central adjudication…)	

Bularga, 2022	35816305	Consecutive patients with suspected ACS	6096	Implementation of an hs-cTn I assay	1. Suspected ACS.<br>2. In this secondary analysis, trial patients with an adjudicated diagnosis of type 1 or type 2 MI for whom the underlying factors associated with oxygen supply–demand imbalance were recorded, were included.	Aim: To evaluate the prevalence and outcomes of different factors associated with oxygen supply–demand imbalance among patients with type 2 MI.<br>The frequency of the primary outcome of all-cause death at 1 year was estimated using the Kaplan–Meier estimate for cumulative incidence, with the log-rank test for comparisons.<br>Competing risk methods that considered the competing risk of non-cardiovascular death were applied.	Prevalence different factors associated with oxygen supply–demand imbalance.<br>All-cause death at 1 year.	Not pre-specified secondary analysis	Tachyarrhythmia was the most common factor associated with oxygen supply–demand imbalance and was associated with better outcomes. Systemic illnesses associated with type 2 MI which presented with anaemia, hypoxaemia, hypotension, or severe hypertension were less common, but had the highest rates of all-cause death.<br>43% (281/650) had evidence of more than one cause of supply–demand imbalance.

						Univariable and multivariable logistic regression modelling were used to examine the association between the factors associated with oxygen supply–demand imbalance and the primary outcome, with type 1 MI as reference group.			
Wereski, 2021	34167318	Suspected ACS without ST-segment-elevation	46 092	Implementation of an hs-cTn I assay	1. Suspected ACS<br><br>Exclusion criteria: previously admitted during the trial period; not residents of Scotland.<br><br>In this analysis, patients with STEMI; cTn at presentation missing; adjudicators unable to arrive at a consensus for the	Aims: To evaluate the performance of recommended cTn thresholds to rule in type 1 MI at presentation.<br><br>To determine whether the kinetics of cTn differs sufficiently to discriminate between	Troponin concentration and change in troponin concentration	Not pre-specified secondary analysis	Troponin concentrations at presentation have a low positive predictive value (PPV) for type 1 MI. Change in troponin on serial testing only marginally improves PPV for type 1 MI over presenting troponin alone.<br><br>Interpretation: Cardiac troponin concentrations at presentation are

					final diagnosis.	myocardial injury and infarction.<br><br>Linear mixed-effects modelling.<br><br>Logistic regression.<br><br>Area under the receiver-operating characteristic (ROC) curve (AUC).			insufficient to distinguish type 1 MI from other causes of myocardial injury or infarction.
Arnadottir, 2021	33297742	Patients referred to elective coronary angiography because of the suspicion of coronary artery disease (CAD)	*N* = 34	Randomly assigned into four groups with different durations of induced myocardial ischaemia (0, 30, 60, 90 s)	Inclusion criteria:<br>• Referred to elective coronary angiography.<br><br>Exclusion criteria:<br>• no coronary artery stenoses or atherosclerosis could be demonstrated<br>• known cardiac diseases	Aims: To improve clinical insight into the mechanisms and diagnostics in ACS based on cTn, the early-release kinetics of cTn and copeptin in the setting of controlled, experimentally induced myocardial ischaemia for durations expected to be too short to	Release of cTn measured by three different hs-cTn I assays (and copeptin)		Increased cTn concentrations were detected by all three assays, and the magnitude of the increase was associated with the duration of ischaemia.<br><br>All assays detected a cTn increase after only 30 s of ischaemia. hs-cTn I (Siemens) rose faster and reached a higher peak.<br><br>Interpretation: cTn can be released also after short

					• known renal failure<br>• plasma creatinine >100 mmol/L.	induce myocardial necrosis were assessed and compared.<br>Comparative statistics.			episodes of ischaemia not expected to cause necrosis.
Kurz, 2000		Patients with angina without creatine kinase (CK) elevation undergoing coronary angioplasty with stent	*N* = 100	Intra-procedure plus 12-h infusion of intravenous (IV) ntitroglycerin	Acute MI, intolerance to nitroglycerin, and haemodynamic instability during angioplasty excluded	Small randomized study, placebo controlled	Minor myocardial necrosis defined as cTn I >3x the upper reference limit (URL) to a level >0.1 mcg/L (from a baseline of <0.1 mcg/L)	Side effect monitored: severe hypotension (minimal systolic arterial pressure <80 mmHg during the study period)	Minor myocardial necrosis was significantly reduced by IV nitroglycerin infusion with a risk of a severe hypotensive response
Alam, 2017	29246240	Patients with stable CAD who underwent elective coronary artery bypass	*N* = 87	CABG	Exclusion criteria included recent MI (within 1 month of surgery), emergency or concomitant valve surgery, significant renal impairment	Randomized clinical trial	The 99th percentile URLs were 34 ng/L (men) and 16 ng/L (women)	Myocardial injury, type 5 MI	Patients undergoing CABG invariably have plasma hs-cTn I concentrations >10-fold the 99th percentile that are not attributable to inflammatory or ischaemic injury alone. Peri-operative

		grafting (CABG)			(estimated glomerular filtration rate [eGFR] <40 mL/min), severe respiratory disease, severe left ventricular (LV) impairment (left ventricular ejection fraction [LVEF] <40%), contraindication to magnetic resonance (MR) scanning and ongoing treatment for chronic inflammatory disease				type 5 MI is often associated with a delayed 24h peak in plasma hs-cTn I concentration.
Morrow, 2000: TIMI-11B Substudy	11092649	Patients with unstable angina and negative serial creatine kinase-muscle/brain isoenzyme (CK-MB) measurements	359	Enoxaparin vs. unfractionated heparin (UFH)	Inclusion criteria:<br>• Symptoms at rest within the previous 24 h and evidence of ischaemic heart disease<br>Exclusion criteria:<br>• Plans for revascularization		Death or MI at 48 h and 14 days<br>Recurrent ischaemia requiring urgent revascularization by 48 h and 14 days	Substudy to a randomized controlled trial (RCT)<br>Low number of study patients and events	Elevation of cTn I among patients with non-ST segment elevation ACS and negative levels of CK-MB identifies those at higher risk for very early adverse outcomes, including severe recurrent ischaemia. Treatment with enoxaparin reduces the risk associated with

					in the first 24 h, regardless of the initial response to medical therapy<br>• CABG within the previous 2 months or percutaneous coronary intervention (PCI) within 6 months<br>• Renal insufficiency (serum creatinine >2.0 mg/dL)				elevated cTn I.
Lindahl, 2000<br>(FRISC)	11036119	Patients with unstable angina	917	Levels of C-reactive protein (CRP) and fibrinogen at enrolment and the maximal level of troponin T during the first	Inclusion criteria:<br>• Unstable angina and onset of chest pain within 72 h before ST-segment depression, T-wave inversion,	Intention to treat	Long-term risk of death from cardiac causes according to levels of troponin T and CRP	Recruitment period began in 1992.<br>The study was part of a prospective,	In unstable CAD, elevated levels of troponin T and CRP are strongly related to the long-term risk of death from cardiac causes. These markers are independent risk factors, and their effects are additive with respect to

				24 h after enrolment	or both<br>Exclusion criteria:<br>• Conditions associated with an increased risk of bleeding			randomized, multicentre trial of low-molecular-weight heparin (dalteparin sodium) in patients with unstable CAD.	each other and other clinical indicators of risk.
Diderholm, 2002<br>(FRISC) II	12040335	Patients with ACS	2457	Early invasive or non-invasive strategy in patients with unstable angina	Inclusion criteria:<br>• Symptoms of cardiac ischaemia associated with either ST depression 0.1 mV or T-wave inversion 0.1 mV in at least one lead or elevation of biochemical marker of myocardial damage with	Intention to treat	Value of troponin T level, alone and combined with ST depression, to identify more severe CAD or a greater efficacy of an early invasive strategy	Randomized trial with a 2 x 2 factorial design	Patients with unstable CAD with the combination of troponin T level ≥0.03 g/L and ST depression have a poor prognosis and, in half of the cases, three-vessel or left main disease

					plasma samples for central evaluation of the troponin T level				

**OBSERVATIONAL COHORT STUDIES**									
**Study first author surname, year, and acronym**	**PMID reference for study**	**Population and study type** (case-control, cohort, etc.)	**Number of participants**	**Stratification**	**Key inclusion & exclusion criteria**	**Type of analysis** (covariate adjustment; propensity score matching, weighting or adjustment; trial emulation; quasi-experimental design…)	**Relevant outcomes and PROMs**	**Other methodological aspects** Bias in design (i.e. immortal-time bias, survivorship bias etc.), risk of residual confounding (i.e. e-value, falsification endpoint), other issues as evaluated in specific tools (i.e. ROBIN I), generazibility.	**Key findings and interpretation**
Daniels, 2026<br><br>REF-TSIX study	41564003	Prospective, multicentre, international, observational cohort study	*N* = 7910 participants (20 to 88 years of age) were	By sex	Participants aged ≥20 years were recruited from multiple sites to ensure the study population was	Sex-specific and uniform 99th percentile URLs were determined using non-parametric methods using inverse	Distribution of cTn T in males and females; proportion of values above the	Large, global study with similar proportion of males and females and	For sample matrices combined (*n* = 8294), 81.0% and 99.2% of cTn T values were above the limit of

			recruited across 34 sites in Europe, the United States, China, and Japan		representative by age, sex, and ethnic distribution. All who met the eligibility criteria were considered healthy and included in the population to determine the reference range. Participants were excluded if they had symptoms or a history of cardiovascular disease, chronic kidney disease, hypertension requiring treatment or elevated cardiometabolic biomarkers, pregnancy, or a history of recreational substance use.	cumulative distribution with averaging discontinuities for cTn T determined using the Troponin T hs Gen 6 assay in female and male populations and the overall population	assay limit of detection	ethnic diversity across a representative age group. Main limitation is that a significant proportion of individuals were excluded post hoc to adhere to new International Federation of Clinical Chemistry and Laboratory Medicine (IFCC) recommendations for the definition of a healthy population that were published during the conduct of the study	detection in females and males, respectively. Sex-specific 99th percentile URLs (95% CI) were 18 (16–23) ng/L for females and 32 (28–35) ng/L for males; the uniform 99th percentile URL was 27 (24–31) ng/L.<br><br>In addition to the 99th percentile URL being two-fold lower in females than males with no overlap in the confidence intervals, cTn T concentrations across the normal reference range were also lower in females, with the first quartile value for males overlapping with the third quartile value for females. Importantly, differences in the distribution of cTn T

									between males and females were consistent across age groups and in all regions, with little variation observed between healthy participants enrolled in the US, Europe, Japan, or China. Sex-specific URLs are defined for use with the new Gen 6 cTn T assay to identify males and females with myocardial injury.
Lindahl, 2025	41241321	Secondary analysis of a prospective, multicentre cohort study of patients with suspected ACS	*N* = 1000 (derivation), *n* = 621 (validation) patients with suspected ACS	cTn was measured at presentation, 1, 2, and 6–36 h, and the final diagnosis adjudicated according to the Fourth UDMI. Two criteria for chronic	Patients >18 years old with suspected ACS. Exclusions: Patients with ST-segment elevation; out-of-hospital cardiac arrest; unable or unwilling to give informed consent; unable or unwilling to comply with study	Descriptive	Final diagnosis of acute myocardial injury or MI	The findings were externally validated	At presentation cTn T was elevated in 242 of 1000 (25%) patients, of whom 94/242 (39%), 13/242 (5%), and 137/242 (56%) had MI, acute or chronic myocardial injury, respectively. A relative change of ≤20% misclassified 58% (59/101) and 49%

				myocardial injury were compared: a relative change in cTn of ≤20% and an absolute change <3 ng/L.	protocol; previous enrolment in this study					(48/98) of patients with a final diagnosis of acute myocardial injury or infarction at 1 and 2 h, respectively, whereas an absolute change of <3 ng/L misclassified 22% (22/101) and 15% (15/98). In the validation cohort (*n* = 621), the relative and absolute change criteria at 1 h misclassified 43% (13/30) and 17% (5/30) of those with MI.<br><br>Chronic myocardial injury cannot reliably be differentiated from acute myocardial injury or infarction by recommended criteria over short remeasurement intervals in the Emergency Department (ED). Longer intervals between sampling and

									absolute rather than relative criteria may reduce the risk of misclassification.
Armillotta, 2025	39968630	Prospective observational cohort study	*N* = 1412 consecutive patients with NSTEMI undergoing PCI with stable or falling pre-PCI cTn I levels	Stratification according to the Fourth UDMI: (1) peri-procedural myocardial injury (PMI) with type 4a MI; (2) PMI without type 4a MI; or (3) no PMI	Exclusion criteria:<br>• Unavailability of serial cTn I measurements<br>• Detection of rising pre-PCI cTn I levels (>20%)<br>• Incomplete data at the 1-year follow-up<br>• Lack of informed consent	Pre-specified sub-analysis of the observational prospective registry AMIPE (Acute Myocardial Infarction, Prognostic and Therapeutic Evaluation; NCT03883711)	The primary endpoint was 1-year all-cause mortality, and the secondary endpoint consisted of MACE at 1 year, including all-cause mortality, non-fatal reinfarction, urgent revascularization, non-fatal ischaemic stroke, and hospitalization for heart failure (HF)	Internal validation was performed in patients enrolled between May 2022 and April 2023	240 (17%) experienced PMI with type 4a MI, 288 (20.4%) experienced PMI without type 4a MI. PMI was associated with an increased risk of adverse clinical outcomes, with patients with type 4a MI demonstrating the highest rates of 1-year all-cause mortality and MACE. A post-PCI Δ cTn I >20% but ≤40% showed similar outcomes to patients without PMI, whereas >40% was identified as the optimal threshold for prognostically relevant PMI, confirmed

									in an internal validation cohort of 305 patients.<br><br>Peri-procedural ischaemic events were frequent in patients with NSTEMI undergoing PCI with prognostic implications. A post-PCI Δ cTn I >40%, combined with an absolute post-procedural value of ≥5 times the 99th percentile URL, was identified as the optimal threshold for diagnosing prognostically relevant PMI.
Saeed, 2024	39119917	Observational cohort study	Patients from a University Hospital in Norway participating in the prospective		Patients ≥18 years (*N* = 1499) admitted to the ED with chest pain suspected of non-ST-elevation ACS. Exclusion criteria included STEMI, short life expectancy, or		Patients were followed from the 3-month sampling point for a median of 1586 (interquartile range [IQR]	Cox models were fitted unadjusted, partially adjusted, and fully adjusted	Acute myocardial injury is equally frequent, whereas chronic myocardial injury is more prevalent using hs-cTn T assay than hs-cTn I. Measuring hs-

			observational WESTCOR study, and who provided a follow-up blood sample 3 months after discharge from hospital<br>*N* = 779		being unable to provide informed consent.		1161–1786) days for a primary composite endpoint of all-cause mortality, MI, revascularization, and HF, and a secondary endpoint of all-cause mortality		cTn T 3 months after an acute chest pain episode could assist in further long-term risk assessment.
Roos,<br>2022	35948410	Observational cohort study	Cohort from seven EDs in Sweden 2009–2016, including 27 809 visits		All visits with a primary complaint of chest pain were identified in the study base. After excluding visits associated with a STEMI diagnosis, we analysed all visits with at least one hs-cTn T concentration measurement, at least one historical hs-cTn T concentration measurement available, and where there was an increase between the	The diagnostic performance of three different hs-cTn T-based diagnostic strategies for MI were evaluated, that is, using (1) only the 0-h hs-cTn T concentration, (2) using only the relative change between the historical and admission hs-cTn T levels (i.e. the Δhs-cTn T), and (3) using the	Diagnosis of MI	Taking the historical cTn T level into consideration when interpreting the admission cTn T increased the possiblity of diagnosing MI	Historical hs-cTn T levels can be used with admission hs-cTn T to improve early risk stratification of MI in the ED

					historical hs-cTn T and the first hs-cTn T concentration measured at the ED visit.	historical-adjusted 0-h hs-cTn T			
Taggart, 2024	38587840	Cohort study using data from two prospective cohorts enrolling consecutive patients with possible MI in Scotland (2013–2016) and Sweden (2011–2014) to assess accuracy of clinical diagnosis of MI recorded in hospital records for patients with an adjudicated diagnosis of type 1 or type 2 MI	Cohort from Scotland included 28 783 and the cohort from Sweden included 21 573 patients with possible MI.<br><br>Type 1 MI = 4298; type 2= 977.	UDMI diagnosis	Scotland: data from High-Sensitivity Troponin in the Evaluation of Patients With Suspected Acute Coronary Syndrome (High-STEACS) trial. Consecutive patients with suspected MI across 10 secondary and tertiary care hospitals.<br><br>Patients were eligible for inclusion if they presented with suspected MI and had paired contemporary and hs-cTn measurements. Patients were excluded if they had been admitted previously during the trial period or were not residents of Scotland.	Sensitivity, specificity, NPV, and PPV of clinical diagnosis of MI for any adjudicated diagnosis of MI and for type 1 and type 2 MI separately, were calculated.<br><br>In patients with an adjudicated diagnosis of type 1 or type 2 MI, univariable and multivariable logistic regression analyses to quantify the association between clinical characteristics and the odds of a clinical diagnosis of MI were calculated.<br><br>The cumulative incidence of the	The main outcome was the proportion of patients with a clinical diagnosis of MI recorded in the hospital records who had type 1 or type 2 MI, adjudicated by an independent panel according to the UDMI. Characteristics and risk of subsequent MI or cardiovascular death at 1 year were compared.	Consecutive patients.<br><br>Non-cardiovascular death was considered a competing risk.	A clinical diagnosis of type 1 MI was recorded in 79% and 87% of the patients with an adjudicated diagnosis of type 1 MI in Scotland and Sweden, respectively. Corresponding figures for a clinical diagnosis of type 2 MI were 17% and 23%, respectively.<br><br>Patients with an adjudicated diagnosis of type 1 MI without a clinical diagnosis were more likely to be women and older, and when adjusting for competing risk from non-cardiovascular death, were at similar

					Sweden: data from a prospective observational cohort study of patients with suspected MI who attended the ED of Karolinska University Hospital in Stockholm. All patients older than 25 years attending the ED with chest pain in whom at least one measurement of hs-cTn was available were eligible for inclusion.	primary outcome was estimated, and group comparisons were made using log-rank test. Multivariable analyses (Cox regression analyses) were conducted to evaluate the association between receiving a clinical diagnosis of MI and the primary outcome.			or increased risk of subsequent MI or cardiovascular death compared with patients with a clinical diagnosis.<br><br>Interpretation: The UDMI was not consistently applied in clinical practice, with a minority of patients with type 2 MI identified, and type 1 MI underrecognized in women and older persons, suggesting uncertainty remains regarding the diagnostic criteria or value of the classification.
Gard, 2023	37220934	Observational study. Two patient cohorts.	Cohort 1 = 300<br><br>Cohort 2 = 964	Adjudicated UDMI diagnosis	Cohort 1: Consecutive patients with elevated cTn but without a clinical MI diagnosis (no ICD-10 code: I.21)	Groups were compared using Mann–Whitney *U* test for non-normally distributed continuous	Aims (1) to describe patients with elevated cTn that were not clinically	Enrollment 2011; Consecutive patients.<br><br>Cohort 1 – single	The adjudication identified 138 and 37 cases of type 2 MI, and 86 and 185 of myocardial injury, with

					Cohort 2: Consecutive patients with a clinical MI diagnosis (with ICD-10 code: I.21).	variables, Student's t-test for normally distributed continuous variables and Pearson's χ2 test for categorical variables. Related samples were compared with McNemar's test.<br><br>Agreement between the reviewers in the MI classification was analysed with Cohen's kappa.<br><br>To compare time to all-cause mortality in patients with and without a clinical MI diagnosis at 1 year and 5 years of follow-up, Kaplan–Meier survival estimates and multivariable Cox regression models were created (two different models).<br><br>Multiple imputation of	diagnosed as having MI, and (2) to compare treatment strategies and prognosis among patients with an adjudicated diagnosis of type 2 MI and myocardial injury with and without a clinical diagnosis of MI at discharge.<br><br>The prognostic outcome was 1-year all-cause mortality.	centre<br><br>Cohort 2 – 8 different hospitals	and without a clinical MI diagnosis, respectively. In patients with type 2 MI, a clinical MI diagnosis was associated with more coronary angiography investigations (39.1% vs. 5.4%, *P* < .001) and an increased use of secondary prevention medications (all *P* < .001). Treatment at a cardiology department was rare among those without a clinical MI diagnosis both for those adjudicated as type 2 MI and myocardial injury.<br><br>No difference was observed in adjusted 5-year mortality between patients with and without a clinical MI diagnosis (hazard ratio [HR] 0.77 with 95% CI 0.43–1.38). The results

						missing values.			were similar for adjudicated myocardial injury.<br><br>Interpretation: Receiving a clinical diagnosis of MI was associated with more cardiology care, including an increased use of coronary assessments and secondary preventive therapies, both in patients adjudicated as having type 2 MI and myocardial injury, respectively. However, no long-term prognostic effect of receiving a clinical diagnosis of MI was observed for either of these conditions.
Devereaux, 2022	35235725	Observational study.<br><br>The VISION Cardiac Surgery	*N* = 13 862	Adjudicated UDMI diagnosis	Patients were eligible if they were at least 18 years of age and underwent any cardiac	Cox analyses were performed using a regression spline that explored the	A primary objective was to determine the relationship	Not consecutive patients.<br><br>The statistical analysis plan was	The threshold troponin level, measured within 1 day after CABG surgery, that was

		study was a prospective cohort study involving patients who underwent cardiac surgery.<br><br>24 hospitals in 12 countries from May 2013 through to April 2019.			surgical procedure, excluding an isolated pericardial window, pericardiectomy, or implantation of a pacemaker or defibrillator. Patients were excluded if they had a pre-operative MI on the same day or the day before their cardiac surgery or if they had a pre-operative hs-cTn I level of ≥300 ng/L within 12 h before surgery.	relationship between log-transformed peak hs-cTn I measurements within 1, 2, and 3 days after surgery and 30-day mortality, adjusting for scores on the EuroSCORE II. From the fitted model, the lowest hs-cTn I measurement that corresponded to an adjusted HR estimate of >1.00 was identified.<br><br>Multiple imputation of missing values.	between post-operative hs-cTn I levels and the risk of death within 30 days after cardiac surgery	adjusted during the study.	associated with an adjusted HR of >1.00 for death within 30 days was 5670 ng/L (95% CI 1045–8260), a level 218 times the URL.<br><br>Interpretation: The levels of hs-cTn I after cardiac surgery that were associated with an increased risk of death within 30 days were substantially higher than levels currently recommended to define clinically important PMI.
Ueki, 2022	35144742	Observational study based on data from Bern PCI registry between January 2009 and December 2018	*N* = 4404	MI by using various definition of peri-procedural MI	Consecutive patients undergoing PCI at Bern University Hospital.<br><br>Exclusion criteria:<br>• No serial cTn measurements<br>• No angiography<br>• No serial	Continuous variables are summarized as mean ± standard deviation (SD) or median (IQR) and compared by using the Student's t-test or Wilcoxon–Mann–Whitney *U* tests, as	Frequency and impact of peri-procedural MI by using various MI definitions among patients with chronic coronary syndrome undergoing PCI.	Single-centre study.<br><br>Limited number of cardiac deaths.	Among patients with chronic coronary syndrome undergoing PCI, peri-procedural MI defined according to the Academic Research Consortium 2 (ARC-2) and Society for Cardiovascular

					electrocardiogram (ECG)<br>• Concomitant procedures.	appropriate. Binary and categorical variables were calculated as frequencies and were compared with the X^2 test or the Fisher exact test. Clinical endpoints were compared between groups using Cox proportional hazards models.	Outcome:<br>Primary endpoint was cardiac death at 1 year.<br>Secondary endpoints were: all-cause death, spontaneous MI, target-vessel MI, repeat revascularization, target lesion revascularization, target vessel revascularization, non–target vessel revascularization, definite stent thrombosis, stroke, and Bleeding Academic Research Consortium 3 or 5 bleeding.		Angiography & Interventions (SCAI) criteria was 7–9 times less frequent compared with the Third and Fourth UDMI. Peri-procedural MI as defined by ARC-2 and SCAI were more prognostic for cardiac death at 1 year compared with the Third and Fourth UDMI.

DeWood, 1980	7412821	Observational study	*N* = 322	Total coronary occlusion	Patients with transmural MI undergoing coronary arteriography within 24 h	Descriptive statistics	Degree of coronary obstruction	Single-centre study	Total coronary occlusion was observed in 110 of 126 patients (87%) who were evaluated within 4 h of the onset of symptoms.<br><br>Interpretation: Total coronary occlusion is frequent during the early hours of transmural infarction and decreases in frequency during the initial 24 h.
Sarkisian, 2016	26593739	Observational study	*N* = 3762	MI, myocardial injury, and non-elevated cTn	Hospitalized patients who had cTn I measured on clinical indication.<br><br>Exclusion criteria:<br>• Aged <18<br>• Residents living outside the local catchment area.<br><br>In cases of multiple admissions, only the first	Aims: 1) to compare clinical features and mortality; 2) to clarify the potential clinical differences between patients with type 2 MI and patients with myocardial injury.<br><br>The Kaplan–Meier method was used to generate survival	All-cause death	Single-centre study. Consecutive patients.	Patients with myocardial injury frequently presented with dyspnoea, were older, and had more comorbidity than patients in the two other groups. Patients with type 2 MI and patients with myocardial injury had high long-term mortality, but there was

					was considered.	curves.<br><br>Univariable and multivariable Cox regression analyses.			no significant difference between the two groups.
Neumann, 2017	29020401	Observational study	*N* = 1548	Type 1 (NSTEMI) and type 2 MI (T2MI)	Data from the Biomarkers in Acute Cardiac Care (BACC) study were used for the analyses.<br><br>Patients who presented with suspected MIExclusion criteria: age <18 or no written informed consent.	Aims: To investigate the clinical characteristics and cardiovascular outcome of patients with T2MI in a large prospective cohort of patients with suspected MI.<br><br>Survival curves were produced using the Kaplan–Meier method. Hazard ratios for each adverse event (adjusted for age, sex, and CAD) were estimated using Cox regression.	The events of death, incident MI, PCI, and cardiac rehospitalization	Single-centre studyLimited adjustments in multivariable analysis.	Patients with T2MI were significantly older than T1MI patients, more often female, and more likely to have atrial fibrillation (AF). Not having a history of CAD, not presenting with typical radiating chest pain, and no pathological changes in echocardiography or ECG were more common in T2MI patients compared with T1MI.<br><br>The unadjusted 1-year mortality rate was higher for T1MI and T2MI patients compared with non-MI patients, while the

									difference between T1MI and T2MI was not statistically significant.<br><br>T2MI patients are a heterogeneous population with high cardiovascular risk.
Boeddinghaus, 2025	39824208	Observational study.<br><br>Prospective cohort study.	*N* = 100	Spontaneous MI, secondary MI, and myocardial injury	Inclusion criteria (all should be present:<br>• Evidence of a rise and/or fall in cTn with at least one value above the sex-specific 99th centile<br>• Symptoms or signs on the 12-lead ECG of myocardial ischaemia<br>• Objective evidence of myocardial oxygen supply or demand imbalance.<br><br>Exclusion criteria:<br>• Suspected type 1 MI	Aims: To evaluate how a new clinical classification with more objective diagnostic criteria for secondary MI using cardiac imaging would impact the diagnosis and identification of those at risk of adverse outcomes.<br><br>The frequency of the primary prognostic outcome was assessed using the Kaplan–Meier estimate, with the log-rank test for comparisons.	The diagnostic outcome was the proportion of patients with a clinical diagnosis of T2MI reclassified as having spontaneous, secondary, or no MI by the new clinical classification.<br><br>The prognostic outcome was a composite of all-cause death, any recurrent MI, or HF	Limited number of study patients and limited number of outcome events.<br><br>Selected group of patients able to participate in the study.	In 100 patients with T2MI, the new classification identified 25 and 31 patients with spontaneous and secondary MI, respectively, and 44 without MI. During a median follow-up of 4.4 years, death, MI, or HF hospitalization was more common in secondary MI compared with those without MI.<br><br>Interpretation: A new clinical classification of myocardial MI informed by cardiac imaging

					• Women who were pregnant or breastfeeding<br>• Renal impairment or severe hepatic impairment<br>• Advanced frailty<br>• Inability to self-transfer.		hospitalization.		would reduce the diagnosis of MI in acute illness and identify those patients at highest risk who are most likely to benefit from treatment.
McCarthy, 2023	37777947	Observational.<br>Prospective cohort study of consecutive patients.	*N* = 50	Type 2 MI causes	Inclusion criteria:<br>• Iinpatients with T2MI<br>• Permission from their clinical team.<br>Exclusion criteria:<br>• Other MI subtypes<br>• Haemodynamically unstable<br>• GFR <30 mL/min/1.73 m^2<br>• Pregnant or breastfeeding<br>• Contrast or nitroglycerin allergy<br>• A pre-operative MI, coronary angiogram,	Aims: To evaluate the presence and characteristics of CAD among patients with T2MI.<br>Descriptive statistics.	Degree of coronary stenosis and fractional flow reserve derived from CT (FFRCT) values	Limited number of patients.<br>Selection towards more healthy patients.<br>Single centre	Coronary CT angiography (CTA) revealed coronary plaque in 92%. Moderate or greater stenosis (≥50%) was identified in 42%, and obstructive disease was present in 26%. Prevalence of obstructive CAD did not differ according to T2MI cause (*P* = .54). A haemodynamically significant focal stenosis identified by FFRCT was present in 13 participants (26%).

					or revascularization and post-operative T2MI<br>• A significant arrhythmia precluding optimal computed tomography (CT) image acquisition<br>• Prior coronary CABG<br>• Prior PCI of the left main coronary artery or PCI of the left coronary system combined with >30% stenosis of the left main coronary artery or who had prior PCI to ≥2 major coronary vessels.				Among participants with stenosis ≥50% (*n* = 21), FFRCT excluded lesion-specific haemodynamically significant stenosis in 8 cases (38%).
Bularga, 2022	35341327	Observational study.<br>Prospective cohort study.	*N* = 100	Type 1 and 2 MI, and myocardial injury	Inclusion criteria (all should be present:<br>• Evidence of a rise and/or fall in cTn	Aims: To evaluate the prevalence of coronary artery and cardiac disease in patients with a clinical diagnosis of	The primary outcome: prevalence of obstructive or non-obstructive	Limited number of study patients and limited number of outcome events.	Coronary and cardiac imaging reclassified the diagnosis in seven patients: type 1 or 4b MI in five and acute

					with at least one value above the sex-specific 99th centile<br>• Symptoms or signs on the 12-lead ECG of myocardial ischaemia<br>• Objective evidence of myocardial oxygen supply or demand imbalance.<br>Exclusion criteria:<br>• Suspected Type 1 MI<br>• Women who were pregnant or breastfeeding<br>• Renal impairment or severe hepatic impairment<br>• Advanced frailty<br>• Inability to self-transfer.	T2MI with systematic coronary and cardiac imaging.<br>Descriptive statistics.	atherosclerotic CAD.<br>Secondary outcomes: assessment of left ventricular systolic function and the pattern of myocardial injury on cardiac imaging.	Selected group of patients able to participate in the study.	myocardial injury in two patients.<br>The prevalence of CAD was 68%, which was obstructive in 30%. Infarct-pattern late gadolinium enhancement (LGE) or regional wall motion abnormalities were observed in 42%. Only 10 patients had both normal coronary and cardiac imaging.<br>Interpretation: Unrecognized and untreated coronary or cardiac disease is seen in most patients with T2MI, presenting opportunities for initiation of evidence-based treatments with major potential to improve clinical outcomes.

Shah, 2017	29114078	Observational study.<br>Prospective cohort study.	Three independent consecutive patient populations<br>*N* = 1054<br>*N* = 5815<br>*N* = 1631	Type 1 and 2 MI	Cohort 1: Unselected patients presenting to the ED.<br>Cohort 2: Patients presenting to hospitals in the UK in whom the attending clinician requested a cTn for suspected ACS.<br>Cohort 3: All patients in whom serial cTn were ordered by the attending clinician for suspected ACS at a single centre in the US.<br>Across all three cohorts, patients with STEMI or a previous presentation during the study period were excluded.	Aims: To evaluate how selection of patients for hs-cTn I testing affects the diagnosis of MI across different healthcare settings.<br>The prevalence of the adjudicated diagnosis of type 1 MI and the subsequent PPVs for a range of pre-test probabilities.	PPV of an elevated cTn I concentration for a diagnosis of type 1 MI.<br>Prevalence of the adjudicated diagnoses.	Consecutive patients.<br>cTn I, no testing of cTn T	In the unselected population (Cohort 1), 13.7% had hs-cTn above the 99th centile, with 1.6%, 1.2%, and 10.8% classified as having type 1 MI, type 2 MI, and myocardial injury, respectively. PPV for type 1 MI of 11.8%.<br>In the selected population (Cohort 2), 4.2% had type 1 MI, 6.3% type 2, and 15.0% myocardial injury. PPV 59.7%.<br>Corresponding figures in Cohort 3 were 4.2%, 6.3%, and 15.0% and PPV 16.4%.<br>Interpretation: When hs-cTn testing is performed widely or without previous clinical assessment, elevated troponin concentrations

									are common and predominantly reflect myocardial injury rather than MI.
Steiro, 2023	37937808	Observational study.<br><br>Prospective cohort study.	1292	Three different hs-cTn assays	Patients without acute myocardial injury were extracted from the Aiming Towards Evidence-Based Interpretation of Cardiac Biomarkers in Patients Presenting with Chest Pain (WESTCOR) study.<br><br>The WESTCOR study included patients ≥18 years consecutively admitted with symptoms suggesting ACS.	Aim: To assess whether chronic myocardial injury (CMI) identified by different hs-cTn assays serves as a uniform and relevant marker of elevated cardiovascular risk.<br><br>The prognostic performances of cTn as continuous variables were assessed by the receiver operating characteristics curve (ROC) for all three endpoints.<br><br>Multivariate analyses were assessed using Cox proportional hazard regression model adjusted for	The prevalence of CMI was calculated for all three assays based on the assays' sex-specific cutoff values. The primary prognostic endpoint was a composite of cardiovascular death, acute MI (AMI) after discharge, or revascularization. The secondary endpoint was all-cause mortality, AMI, revascularization, or hospitalization		There was no major difference in prognostic accuracy between cTn T and cTn I analysed as continuous variables. The correlation between cTn T and cTn I was high (r = 0.724–0.785), but the cTn T assay diagnosed 3.9–4.5 times more patients with having CMI based on the sex-specific URLs and had higher clinical sensitivity and area under the curve (AUC) at the URL.

						age, sex, and eGFR.	for HF or stroke.		
Landesberg, 2014	24365861	Observational study.<br>Prospective cohort study.	*N* = 106		Consecutive patients with severe sepsis or septic shock.<br>Excluded were patients with greater than mild mitral and/or aortic valve disease, patients with echocardiographic evidence of regional myocardial wall motion abnormalities suggesting ischaemia or previous infarction, patients with poor-quality echocardiographic images and measurements, and patients with AF whose uneven cardiac beats interfered with echocardiographic measurements.	Aims: To study possible causes for troponin elevations in sepsis.<br>Univariate and multivariate mixed general linear models (GLMs) were used to examine correlations of echocardiographic and clinical variables with log10 (hs-cTn T).	Different measures of cardiac function obtained by echocardiography using global strain, strain-rate imaging, and 3D left and right ventricular volume analyses in addition to standard echocardiography	Lack of information on inflammatory markers, e.g. tumour necrosis factor (TNF)-alpha	Left ventricular diastolic and right ventricular systolic dysfunction seem to explain the association of troponin with mortality in severe sepsis and septic shock
Friedlich, 2023	37290439	Cohort study	Two cohorts:<br>*N* = 17	n/a	Two cohorts:<br>1: Healthy volunteers	Aims: To identify the cellular origins of cell-	Circulating cfDNA measured in		Strenuous physical exercise causes a

			N = 15		doing controlled exercise<br>2: Half-marathon runners	free DNA (cfDNA) in exercise and to study the physiological drivers leading to elevated cfDNA	blood		massive elevation in the concentration of circulating cfDNA, which correlates with effort intensity and duration.<br>cfDNA in exercise originates mostly in extramedullary polymorphonuclear neutrophils.<br>Also cardiomyocyte cfDNA concentration increases after a marathon, consistent with elevated troponin levels and indicating low-level, delayed cardiac cell death.
Kubo, 2007	17765119	Prospective cohort	30	Comparison of intravascular ultrasound (IVUS), coronary angioscopy (CAS) and	Inclusion criteria:<br>• STEMI<br>• Symptom onset ≤6 hours<br>• Identifiable culprit lesion on	Comparison of plaque characteristics with X^2 testing pairwise tests of incidence used to control for multiple comparisons.<br>Intra-observer and			Plaque rupture identified with OCT vs. CAS: 73% vs. 47%, *P* = .035.<br>Plaque rupture identified with OCT vs. IVUS: 73% vs. 40%, *P*

				optical coherence tomography (OCT) imaging	angiography<br><br>Exclusion criteria :<br>• Presence of left bundle branch block (LBBB)<br>• Paced rhythm<br>• Culprit lesion of left main artery<br>• History of MI<br>• Cardiogenic shock<br>• Thrombosis in Myocardial Infarction (TIMI) flow grade <3 after aspiration thrombectomy and before imaging<br>• Inability to advance imaging catheter to the lesion	interobserver variabilities were measured by test of concordance.			= .009.<br><br>Plaque erosion identified with OCT vs. CAS: 23% vs 3%, *P* = .022.<br><br>Plaque erosion identified with OCT vs. IVUS: 23% vs. 0%, *P* = .005.<br><br>Intracoronary thrombus identified in OCT vs. CAS: 100% vs. 100%.<br><br>Intracoronary thrombus identified in OCT vs. IVUS:_100% vs. 33% *P* < .001.<br><br>OCT also able to detect fibrous cap thickness and thin cap fibroatheroma.
Ambrose 1998	3379219	Retrospective case-controlled study	38	Comparison of angiographic findings of CAD in	Inclusion criteria for patients with MI:<br>• MI	Wilcoxon non-parametric test used to compare degree of stenosis.	Progression of stenosis to 100% on follow-up angiogram		Median percent stenosis at baseline angiogram: MI vs. no MI: 48% vs. 73.5%, *P* <

				patients with MI and those without MI who later developed 100% occlusion	• Angiographic data available before and after infarct<br>Exclusion criterion for patients with MI:<br>• Revascularization between two angiograms<br>Inclusion criteria for comparator group:<br>• Two sets of angiograms performed<br>• Total occlusion noted on second angiogram	Student's t-tests used to compare differences in continuous variables.<br>Fisher's exact test used to compare differences in discrete variables.<br>Stepwise multivariate analysis used to determine predictors of MI in follow-up.			.05<br>Percent of baseline lesions >70%: MI vs. no MI: 22% vs. 61%, $P < .01$<br>Independent predictor of progression to acute MI in lesions ≥50%: proximal lesion location
Polzl, 2021	35139192	Retrospective observational study<br>Patients with multivessel CAD undergoing CABG	*N* = 2829	None	Patients lacking serial biomarker measurements were excluded	Cox proportional hazard models with HRs and 95% CIs	Peri-operative MI defined according to SCAI criteria, the Fourth UDMI, and the ARC criteria	MACE, defined as the composite of MI, all-cause mortality, and repeat revascularization.<br>The secondary endpoints were	Significant difference in the occurrence of PMI according to different definitions. The Fourth UDMI and ARC criteria remained strong independent predictors for all-cause mortality at 30 days and during 5-

								mortality at 30 days and during 5-year follow-up.	year follow-up.
Crescenzi, 2003	15145001	Prospective observational study.<br><br>Patients undergoing cardiac surgery (for coronary and valvular disease)	*N* = 206	None	Patients with pre-operative Q waves, pre-existing or post-operative LBBB, thoracotomy, and low ejection fraction (EF) (<35%) were excluded	Logistic regression analysis. For each marker studied, ROC curves constructed by plotting sensitivity against one specificity for cardiac events	Cardiac biomarker cutoff values based on routine criteria (CK-MB >40 ng/mL and cTn I >11 ng/mL)	New Q waves; cardiac event defined as the occurrence of low output syndrome (cardiac index <2 L/min/m^2) secondary to ventricular dysfunction requiring high doses of inotropes or intra-aortic balloon pump (IABP) for >24 h	The association of a new Q wave and high levels of myocardial necrosis biomarkers is strongly associated with post-operative cardiac events
Ndrepepa, 2016	27884243	Retrospective observational study.<br><br>Patients with stable CAD undergoing	*N* = 5626	None	Patients with ACS, acute infections, pregnancy, advanced impairment of renal function (serum creatinine >2.0 mg/dL), or a known malignancy were excluded	Survival analysis performed using Kaplan–Meier method and differences in survival rates assessed according to univariable Cox	Baseline and peak post-procedural hs-cTn T measurements available (two or more post-	Mortality at 3 years	An increase in post-procedural hs-cTn T level did not offer prognostic information beyond that provided by the baseline level of the biomarker

		elective PCI.				proportional hazards model	procedural hs-cTn T measurements were performed in 98.7% of patients. The peak level was deemed as the highest post-procedural hs-cTn T concentration).		
Omran, 2022	35165695	Retrospective observational study.<br>Patients undergoing isolated CABG.	*N* = 4684	None	Exclusion criteria included urgent or emergent procedures (e.g. due to ACS), paediatric (<18 years) patients, as well as CABG combined with the valvular procedure or ablation for AF	ROC curve analysis to identify optimal cutoff values of hs-cTn I, and sensitivity, specificity, NPV and PPV calculated.<br>Cox proportional hazards regression analysis for survival analysis.	In patients with an uneventful post-operative course, hs-cTn I levels reached their peak of 90×URL at a median of 8.0 h, after which<br>they gradually decreased. In contrast, revascularized patients had a bimodal curve	Repeat revascularization, defined as angiography with consequent PCI or redo-surgery based on clinical decision of the local heart team.<br>Other outcomes: angiographic vessel occlusion, and type 5 MI according to the UDMI.	hs-cRTn I levels determined 12–16 h after surgery with a cutoff of 8000 ng/L (307×URL) correlated best with a decision to repeat revascularization.

							with a first peak of 992×URL at a median of 18.5 h (before invasive coronary angiography [ICA], which was done at a median of 20.7 h after surgery) and a second peak of 1415×URL at a median of 25.3 h post-operatively, after which hs-cTn I levels rapidly decreased. Patients with ICA not undergoing revascularization also showed a rapid increase with a peak of 1096×URL at a median time of 17 h.		

Spirito, 2024	39048279	Retrospective observational study.<br>Patients with chronic coronary syndrome and baseline troponin <1x URL or with acute spontaneous MI who underwent PCI.	*N* = 10 707	None	Patients with unstable angina or with chronic coronary syndrome and elevated (>1 URL) baseline troponin were excluded	Kaplan–Meier method with time-to-first event analysis with risks assessed using Cox proportional hazards regression models	PMI was defined as post-PCI cTn I increase >1x URL (0.4 ng/mL for the troponin I assay used between 1 January 2012 and 21 April 2019, and 0.03 ng/mL for the assay used from 22 April 2019) in patients with chronic coronary syndrome. Spontaneous MI comprised any ACS with elevated troponin.	Mortality at 1 year	Mortality at 1 year was significantly increased after post-PCI MI and spontaneous MI with troponin peak >35 URL, whereas for troponin levels <35 URL only spontaneous MI had a relevant impact on mortality.
Miller, 2006	16481332	Prospective observational study.<br>Patients referred for elective or urgent coronary	*N* = 2352	None	Patients with STEMI or with missing baseline troponin levels were excluded	Cox proportional hazard regression models	An elevated cTn T was defined as a value >0.03 ng/mL per European Society of Cardiology	Death and MI at 1 year	Neither the timing nor the magnitude of the post-procedure cTn T elevations was predictive of long-term death/MI rates when

		angiography and PCI with the diagnosis of progressive symptomatic CAD or ACS.					(ESC)/American College of Cardiology (ACC) guidelines and an elevated CK-MB mass was defined as >6.2 ng/mL		baseline elevations were included in the analysis. Similar findings were observed for baseline CK-MB levels.<br>Long-term prognosis is most often related to the baseline pre-PCI troponin value and not the biomarker response to the PCI.
Kotronias, 2025	39985506	Participants included from two studies: Oxford Acute Myocardial Infarction Study (prospective) and Oxford Risk Factors and Noninvasive Imaging study (consecutive patients)	*N* = 100 patients who had both photon-counting coronary computed tomography angiography (PC-CCTA) and ICA		Participants included from two studies at the University of Oxford who had both PC-CCTA and ICA. Exclusion of active malignancy, brittle bronchospastic disease, and eGFR <30mL/min/1.73m^2.	Bland–Altman plots with bias estimates used to evaluate agreement between modalities. Mean differences compared by Levene and Mann–Whitney *U* tests. Cohen's κ statistic used to evaluate the inter-modality agreement. Diagnostic accuracy for stenosis ≥50% by 3-dimensional	ICA analysed with 3D QCA. CCTA evaluated on syngo.via (Siemens) within the core laboratory.		Per-vessel agreement near-perfect (κ = 0.90 [Q1–Q3: 0.84–0.95], including in severely calcified arteries: κ = 0.90 (Q1–Q3: 0.83–0.97). Per-vessel diagnostic performance of PC-CCTA was excellent: AUC: 0.94 (95% CI 0.91–0.98; *P* < .001) and 0.99 (95% CI 0.98–1.00; *P* < .001).

						quantitative coronary angiography (3D QCA) measured as the area under the ROC curve (AUC).			
Gascho, 2024	36862287	Retrospective observational study	*N* = 88 post-mortem magnetic resonance imaging (MRI) examinations	None	Adult decedents (male: *n* = 69), who underwent post-mortem cardiac MRI between 2013 and 2019 because of suspected ischaemic heart disease before conventional autopsy	The post-mortem interval between time of death (estimated maximum value) and post-mortem MRI was less than 6 days. Conventional autopsy was performed less than 24 h after MRI. The results of autopsy and, if performed, histology are considered the gold standard.	Sensitivity and specificity T1- and T2-weighted 3-T MRI for post-mortem detection of MI; comparison of MRI appearance of the infarct area with age stages.	Limitation: histological examinations were not performed in all decedents who had MI at autopsy, and therefore the estimate of the age stage at autopsy could not be additionally confirmed	MRI could help to classify the age stage and may indicate the area for sampling for further microscopic examination. However, the low sensitivity requires further additional MRI techniques to increase the diagnostic value.
Jangaard, 2017	28159605	Registry-based cohort study	2766 individuals died out of 246 723 included individuals	*n* = 18 died of type 3 MI vs. *n* = 488 died of other types of MI	Data retrieved from the Danish Civil Registration System from 2010 to 2011. Type 3 MI defined as cardiac death in patients with symptoms and ECG changes	Data on causes of deaths were obtained from the Danish Register of Causes of Death, and ambulance and hospital patient files. Adjudication of	Analysis of the incidence, frequency, and characteristics of patients diagnosed as	Enrolment of consecutive individuals within a 1-year period	Type 3 MI is rare in clinical practice (annual incidence below 10/100 000 person-years, frequency of 3%–4% among all types of MI)

					indicative of myocardial ischaemia but without available measurement of cardiac biomarkers.	diagnosis was done by two local experts and one external senior cardiologist.	type 3 MI		
Wagensveld, 2018	29474537	Corss-sectional study	*N* = 100	None	Inclusion of consecutive adult patients who died in hospital, and for whom next of kin gave permission to perform both conventional autopsy and minimaly invasive autopsy. Exclusion: unnatural cause of death, body height >16 inches, known or suspected 'high-risk' infected bodies, open abdominal wounds.	Key analyses: sensitivity and specificity and ROC curves for CT and MRI as the standalone test or combined with biopsy	Assessment of the diagnostic accuracy of post-mortem MRI, CT, and CT-guided biopsy for the detection of acute and chronic myocardial ischaemia	Prospective single-centre cross-sectional study in a tertiary referral hospital	Sensitivity and specificity of MRI with biopsies for acute MI was 0.97 and 0.95, respectively and 0.90 and 0.75, respectively for chronic MI. MRI without biopsies showed a high specificity (acute: 0.92; chronic: 1.00), but low sensitivity (acute: 0.50; chronic: 0.35). CT (total Agatston calcium score) had a good diagnostic value for chronic MI [AUC 0.74], but not for acute MI (AUC 0.60).<br><br>Interpretation: the combination of MRI with biopsies had high

									sensitivity and specificity for the detection of acute and chronic myocardial ischaemia.
Gerhardt, 1991	1868602	Comparison of three tests for ischaemic myocardial injury detection		Automated enzyme immunoassay for S-troponin T	Inclusion criteria:<br>• Patients with myocardial injury or unstable angina<br>Exclusion criteria:<br>• Myocardial infarction within the last 24 hours	Cox regression models			
Eggers, 2017	28647406	Retrospective registry-based (SWEDEHEART) analysis	22 375	Unstable angina and NSTEMI	Inclusion criteria:<br>• Unstable angina or NSTEMI<br>• cTn T assay.<br>Exclusion criteria:<br>• STEMI<br>• Elective admission.	Cox regression models	Patients with unstable angina have a non-neglectable cardiovascular risk	Only peak cTn T values are registered	Proportions of unstable angina patients among those with non-ST-elevation ACS varied considerably between the hospitals, ranging from 4.0% to 22.2%

Chin, 2014	24829362	Prospective cohort study. First cohort: patients with aortic stenosis who underwent cardiac MRI and transthoracic echocardiography (TTE). Second cohort: patients with aortic stenosis followed over 10.6 years for clinical outcomes.	First cohort: *n* = 122. Second cohort: *n* = 131.	n/a	First cohort: Patients with mild to severe aortic stenosis were recruited prospectively, excluding patients with other significant (moderate or severe) valvular heart disease or cardiomyopathies. Second cohort: Patients with asymptomatic moderate to severe aortic stenosis. Excluded if aortic valve replacement (AVR) was planned, or in case of moderate or severe left ventricular (LV) systolic impairment.		First cohort: Indexed LV mass and measures of replacement fibrosis (LGE). Second cohort: Occurrence of AVR and cardiovascular death.	In the second cohort, outcomes were adjusted for age, sex, systolic ejection fraction, and aortic stenosis severity	In patients with aortic stenosis, plasma cTn I concentration is associated with advanced hypertrophy and replacement myocardial fibrosis as well as AVR or cardiovascular death
Nellessen, 2006	16739395	Prospective, observational study. Consecutive patients hospitalized for chronic HF vs.	*n* = 58 pts with chronic HF, *n* = 31 controls	Ischaemic vs. non-ischaemic dilated cardiomyopath y	Serial measurements of cTn I release were performed every 2 weeks for 3 months. After 3 years, patients' survival was analysed and compared with	No adjustments reported	Changes in troponin levels in HF patients vs. controls; survival at 3 years in relation to troponin levels	Imbalance in baseline characteristics (age, medications, cardiovascular risk factors)	Permanent cTn I release is common in patients with chronic HF and a strong prognosticator. Coronary morphology played a minor role in

		healthy volunteers serving as control group.			troponin release.		and HF phenotype	without statistical adjustments; renal function not assessed	disease progression.
Kwong, 2008	18725488	Retrospective observational cohort study	197	The absence or presence of clinical evidence of MI (clinical history of MI or Q waves on ECG)	Diabetes mellitus	Cox regression analyses were performed to associate CMR-LGE with MACE	The presence of CMR-LGE was the strongest multivariable predictor of MACE and death by stepwise selection in the study patients	CMR is a new and costly imaging modality among other available non-invasive modalities; it is possible that selection bias exists from clinicians' referral.<br>Whether any prognostic association of CMR- LGE with MACE exists in diabetics at lower pre-test likelihood of CAD requires future study.	CMR imaging can characterize MI in diabetic patients without clinical evidence of MI. This imaging finding demonstrates strong association with MACE and mortality hazards.

Nordenskjöld, 2018	29979788	Prospective cohort study	235	Long-term (mean follow-up 5.4 years) outcome of recognized and unrecognized MI assessed with CMR	Patients with stable suspected CAD scheduled for elective coronary angiography.<br>Patients with pathological Q wave on ECG, kidney failure, history of previous MI, coronary interventions or HF were excluded.	Univariate and multivariate Cox regression analyses were performed. The assumption of proportional hazards was verified by Schoenfeld residual test and time-varying covariates were assessed. Results presented as HRs with 95% CIs.	LGE-CMR-detected unrecognized MI are independently associated with an adverse prognosis. It seems reasonable to optimize treatment of cardiovascular risk factors as well as consider coronary angiography in patients with cardiac symptoms, in patients with detected unrecognized MI at cardiac CMR.	The study cohort consists of patients with suspicion of stable angina pectoris and generalizations of the results to other groups should be done with caution.<br>The number of events was low giving limited power to demonstrate significant associations.	Unrecognized MIs were present in 25% of patients with suspected CAD.<br>The presence of unrecognized MI was independently associated with an increased risk of cardiovascular events during long-term follow-up.
Barbier, 2006	16904547	Prospective cohort study	248	Recognized and unrecognized	70-year-old subjects	Descriptive statistics and the Bonferroni correction for post hoc	The subjects displaying these unrecognized MIs	Post hoc analysis of a study randomized	Unrecognized MI detected with MRI was more frequent (19.8%)

				MI assessed with CMR		analysis	may represent a previously unknown potential risk group for future cardiovascular events	population (unselected subsample from the PIVUS (Prospective Investigation of the Vasculature in Uppsala Seniors) study	than expected in 70-year-old subjects
Fernandez-Friera, 2024	38426763	Prospective cohort study	712	CMR used to assess for presence of ischaemic scar	Asymptomatic middle-aged (median 51 years, 84% male) individuals, without known prior history of cardiovascular disease (CVD)	Descriptive statistics	Unrecognized MI (ischaemic scar) detected in 2.9% on CMR	On CMR, 19.1% of participants had positive LGE and ischaemic scar (subendocardial or transmural LGE) identified in 2.9%	In middle-aged individuals, ischaemic scar on CMR is present in 2.9%
Antiochos, 2020	32819469	Retrospective multicentre cohort study	2349	Recognized and unrecognized MI during the follow-up:<br>The	Inclusion criteria: age 35–85 years; referral for evaluation of suspected CAD	Cox proportional hazards were used to assess the association of MIs with outcomes. Kaplan–Meier curves were generated by	Unrecognized MI was detected in 347 patients (14.8%) and clinically recognized MI in	LGE imaging can miss small endocardial MI after acute infarct healing, thus reducing its	Presence of unrecognized MI or clinically recognized MI has an equally significant risk for death and/or MI,

				incremental prognostic value of unrecognized MI detected during assessment of CAD by stress CMR.		plotting cumulative incidence of study outcomes by years of follow-up and compared using a log-rank test.<br><br>Multivariable clinical risk model was used with a stepwise forward Cox regression strategy.	358 patients (15.2%)	sensitivity	independently of the presence of ischaemia. Unrecognized MI presented an increased risk for HF hospitalization.
Boland, 2002	12398956	The Atherosclerosis Risk in Communities (ARIC) study is a population-based prospective cohort study	12 843	ECG evidence of MI defined as the appearance between the baseline and subsequent examinations (during 10-year follow-up) of a major Q wave or a minor Q wave with ischaemic ST-T changes	Participants in the ARIC study. Men and women, aged 45 to 65 years, who were free of clinically recognized coronary heart disease and ECG evidence of MI.<br><br>Subjects were excluded if they were not white or African American, if they had prevalent coronary heart disease at baseline, or if data were insufficient to determine baseline coronary heart	Descriptive statistics	The percentage of unrecognized MIs in men and women was similar	A greater sensitivity over time in clinical detection of MI might have resulted in observing a lower proportion of unrecognized MIs. The total number of MIs may have become less comparable across studies by	Proportion of unrecognized MI was 20%, with African Americans having a higher percentage (23%) than whites (19%).<br><br>The percentage of unrecognized MIs in the ARIC sample between 1987 and 1998 was slightly lower than previous estimates from other populations.

					disease status.			recent advances in diagnostic techniques, such as cardiac enzyme measurements that allow for greater sensitivity in documenting less severe cases of MI.	
Delewi, 2013	23433932	Prospective multicentre study	184	Standard 12-lead ECG was recorded before and 1 h after the procedure and at 1, 4, 12, and 24 months.<br>CMR examination was performed at 4 ± 2 days after reperfusion and repeated	STEMI treated by PCI.<br>Patients with a history of MI were excluded.	Univariate analyses.<br>No adjustments for multiple comparisons were made.	The Q-wave/non–Q-wave MI distinction is of clinical relevance, because Q waves are associated with a lower ejection fraction and a larger MI. Q-wave regression at 24 months was present in 40% of the patients with initial Q-wave MI and was	The study is limited by a small sample size, especially in the group with Q-wave regression	Association of Q waves with infarct size is strongest when using the classic Q-wave criteria. Q-wave regression is associated with the largest improvement of LVEF as assessed with CMR.

				after 4 and 24 months.			associated with the largest reduction of infarct size and improvement of LVEF as assessed on CMR. 92% of patients with persistent Q waves had microvascular obstruction on baseline CMR.		
Hammar, 2015	26585508	Observational study, multicentre, prospective	*N* = 235	An LGE-CMR examination was performed prior to the coronary angiography	Patients with stable angina pectoris and without prior MI, scheduled for coronary angiography.<br>• Exclusion criteria: Pathological Q wave on 12-lead ECG<br>• Previously known MI<br>• Previous PCI<br>• History of congestive HF	Data by segment were analysed with a generalized estimating equation (GEE) model that fully accounts for any dependence between segments for the same patient. The relation between severity of stenosis and unrecognized MI was analysed in a multivariate model	LGE findings indicating ischaemic myocardial necrosis, coronary stenosis	Patients with Q waves were excluded	Unrecognized MIs were found in 25% of patients. There was a strong association between stenotic lesions (≥70% stenosis) in a coronary artery and the presence of an unrecognized MI in the myocardial segments supplied by the stenotic artery; it was significantly more likely

					• eGFR <30 mL/min/1.73 m^2<br>• Contraindications for CMR examination<br>• Lack of suitability for participation.	adding age, sex, diabetes and hypertension as covariates.			to have an unrecognized MI downstream of a stenosis (≥70% vs. <70%; OR 5.1, CI 3.1–8.3, *P* < .0001).<br><br>Unrecognized MI is common in patients with stable angina and the results indicate that the majority are of ischaemic origin due to severe coronary atherosclerosis. In contrast to what is seen in recognized MIs, unrecognized MIs are predominantly located in the inferior and inferolateral myocardial segments.
Heitner, 2019	31035776	Multicentre prospective observational study	114	NSTEMI patients	Appropriate rise and fall of troponin (conventional assays, not high-sensitivity) in the absence of ST-segment	Comparisons of continuous data between groups were made using 1-way or 2-way analysis of	Identification of the IRA by CMR-LGE with ICA in patients presenting with	The relatively small elevation in initial troponins and the low incidence of ST-	Identification of the IRA by coronary angiography can be challenging in patients with NSTEMI. In nearly

					elevation in the appropriate clinical setting (typical chest discomfort or angina equivalent). Patients were included in the study if they had no contraindications to ICA, met the criteria for NSTEMI as above, and had no history of MI or previous coronary artery bypass surgery. Patients were excluded from the study if they were haemodynamically unstable or deemed to require immediate coronary revascularization, had a glomerular filtration rate <30 mL/min per 1.73 m^2, or had a contraindication to CMR.	variance (ANOVA) test as appropriate. The χ^2 test was used to make between-group comparisons of discrete data. McNemar test used to compare the proportions of patients with an infarct-related artery (IRA) identified by ICA with that identified by CMR.	NSTEMI	segment changes raises the possibility of some selection bias in the study population	half, CMR-LGE may lead to a new IRA diagnosis or elucidate a non-ischaemic pathogenesis.
Liang, 2023	37526288	Large single-centre cohort	888 patients	MINOCA endotypes	All patients included had undergone diagnostic ICA, followed by CMR.		Identify the proportion of patients with	The Bristol CMR registry is derived from patients who	In a large single-centre cohort of patients presenting with ACS

		study			Those with obstructive coronary disease (≥50% stenosis) on angiography, and those with no documented troponin result or a negative troponin on review on clinical notes, were excluded		suspected MINOCA or non-MINOCA that CMR reclassifies with an alternative diagnosis	have undergone CMR for investigation with myocardial injuries and non-obstructed coronary arteries over a period of 10 years.<br>The time period also covered the COVID-19 pandemic and thus may not have recruited all potential patients, therefore potentially underestimating the prevalence of each diagnosis.	and unobstructed coronary arteries, CMR was able to reclassify diagnosis in nearly 80% of patients. In addition, the majority of patients with suspected ACS with unobstructed coronary arteries have an alternative non-MINOCA diagnosis (73%: myocarditis, Takotsubo, cardiomyopathies, or normal CMR/non-specific findings), whilst only a minority had confirmed MINOCA (27%).
Williams, 2022	36075617	Large single-centre cohort study	710	Patients with suspected MINOCA	Consecutive patients with ACS and non-obstructive coronary arteries without an obvious cause	Logistic or Cox regression models were used to estimate predictors of a diagnosis by CMR	Primary endpoint of the study was the diagnostic yield of CMR. The Youden index	No consistent sampling strategy for troponin, and the time between symptom onset	The combination of CMR performed <14 days from presentation and peak troponin T ≥211 ng/L leads to a

					underwent CMR.<br>Inclusion:<br>• Positive dynamic biomarker<br>• Ssuspected acute MI<br>• Non-obstructive coronary arteries (<50% stenosis)<br>• No other overt cause for the presentation at the time of angiography.<br>Patients were excluded for the following reasons: 1) there was a clear cause for presentation (e.g. pulmonary embolism, tachyarrhythmia); 2) there was no diagnostic invasive or CT coronary angiogram confirming the absence of >50% epicardial coronary stenosis; 3) there was no		was used to find the optimal diagnostic cutoff point for peak troponin T to combine with CMR to improve diagnostic yield.	and presentation not recorded.<br>Study design did not include T1 and T2 mapping, which may have further increased the diagnostic sensitivity and specificity of CMR.	very high diagnostic yield (94%) on CMR. The diagnostic yield remains high (72%) even when CMR is performed ≥14 days from presentation, but reduces to 53% when peak troponin T was <211 ng/L.<br>An increase in one peak troponin decile increases the odds of the CMR identifying a diagnosis by 20% (OR: 1.20; *P* = .008, 95% CI 1.05–1.36).

					troponin T result of >14 ng/L available; and 4) there was a 'false positive' troponin (defined as a single troponin elevation <5x the upper limit of normal (ULN) that was not repeated or a single elevated troponin that returned to normal within 24 h.				
Dastidar, 2019	30772224	Observational	388	Patients with suspected MINOCA	Patient with ACS and unobstructed coronary arteries undergoing CMR. Troponin T level <14 ng/L was considered normal. The typical rise or fall of troponin T was used for the diagnosis of acute MI. False-positive troponin possibilities included a single troponin elevation (<5x ULN) that was not	In univariate analyses, the association of time variables to mortality was assessed using Kaplan–Meier curves and the log-rank test. Univariable and multivariable associations of risk covariates with mortality were assessed using Cox proportional hazard regression analyses.	The endpoint of the study was all-cause mortality		In a large cohort of patients with MINOCA, CMR (median 37 days from presentation) identified a final diagnosis in 74% of patients. Cardiomyopathy had the highest mortality, followed by MI. The strongest predictors of mortality were a CMR diagnosis of cardiomyopathy and ST-segment elevation

					repeated during admission or if a single elevated troponin was followed by a second normal troponin level within 24 h was excluded from study.				on presentation ECG.
**OTHER ARTICLES**									
**Study first author surname and year**	**PMID reference for study**	**Type of article** (Editorial, commentary, systematic review, narrative review, clinical practice guideline or consensus statement)	**Population**	**Topics**	**Key point (1)** (Evidence level if clinical practice guideline)	**Key point (2)** (Evidence level if clinical practice guideline)	**Key point (3)** (Evidence level if clinical practice guideline)	**Other considerations** (Bias, uncertainty, future research requirements, etc.)	**Interpretation**
Kumar, 2024	37906238	Expert Consensus Statement (Canadian Cardiovascular	Spontaneous MI	Staging of MI (1–4) by extent of tissue injury using biomarkers	CCS stage 1: aborted (oedema, reversible wall motion abnormality).<br>CCS stage 2: classical (oedema, cardiomyocyte	CCS stage 3: oedema, cardiomyocyte necrosis, microvascular obstruction.	Extent of tissue injury associated with prognosis	Biomarker thresholds not defined for staging; CMR required to	Current classification does not recognize extent of infarction is important; encourage serial biomarker testing

		Society [CCS])		and CMR	necrosis).	CCS stage 4: significant myocardial necrosis, intramyocardial haemorrhage.		classify limiting generalizability	in confirmed spontaneous MI and imaging to evaluate size; recognition of haemorrhage may have implications for treatment
Lindahl, 2023	37635156	Narrative review/opinion paper	n/a	Definition of MI	The current classification (especially types 2–5) has not been applied consistently in practice and is perceived by some as too complicated	A new clinical classification is proposed, recognizing that MI can arise spontaneously, secondary to another condition, or as a complication of a cardiac procedure		n/a	The classification is aligned with clinical practice and proposes more objective and specific diagnostic criteria
Gaudino, 2024	39279125	Narrative review/debate paper	n/a	Definition of MI after cardiac surgery	The authors that are pro that MI after cardiac surgery must be redefined argue that substantially elevated cutoff levels for cTn are needed (up to 170x URL)	The authors that are anti that MI after cardiac surgery must be redefined argue that substantially elevated cutoff levels for cTn are not needed		n/a	There is no consensus among experts on how MI after cardiac surgery should be defined

Jaffe, 2021	33486520	Narrative review	n/a	Use of CK-MB for diagnosis of MI	Measurement of CK-MB offers no additional value to measurement of cTn and the use of CK-MB is rapidly decreasing			n/a	Interpretation: CK-MB has no role in the diagnosis of MI
Miranda, 2018	29407007	Review	n/a	ECG criteria for diagnosing MI	To review the use of the ECG to diagnose coronary occlusion MIs, including the application of rules that allow MI to be diagnosed on the basis of atypical ECG manifestations			n/a	Interpretation: Careful interpretation of the ECG might yield subtle features suggestive of ischaemia that might facilitate more rapid triage of patients with subtle acute coronary occlusion or, conversely, in identification of ST-elevation MI mimics (pseudo ST-elevation MI patterns)
DeFilippis, 2019	31416350	Review	n/a	Type 2 MI and myocardial injury	Myocardial injury can result from a wide variety of ischaemic and non-ischaemic mechanisms. Type 2 MI and non-ischaemic myocardial	The reported prevalence of type 2 MI ranges from 2% to 58% of patients with MI.<br>Acute non-ischaemic	Differentiating myocardial injury subtypes is challenging	n/a	Type 2 MI and acute myocardial injury are common and sometimes difficult to separate

					injury encompass a heterogeneous group of mechanisms.	myocardial injury varies substantially by the population studied and has been reported to be greater than MI (types 1–5).			
Singh, 2022	35344411	Review	n/a	Takotsubo syndrome	To provide an update on the current understanding of the clinical features of this condition, and to discuss the latest theories regarding its pathogenesis and pathophysiology	The hallmark of the Takotsubo syndrome is ultimately the reversibility in systolic function that occurs within hours, days, or weeks, in the absence of infarct-specific myocardial fibrosis			The mechanisms behind this condition remain elusive. Despite substantial research, the medical community continues to have an incomplete understanding of its underlying pathogenesis and pathophysiology.
Eichhorn, 2022	35592889	Review	n/a	Myocarditis	To describe the evidence surrounding different CMR methods and image post-processing methods and highlight their value for clinical decision-making, monitoring, and risk stratification across	Myocardial tissue characteristics by CMR have demonstrated association with the clinical outcome of patients with suspected myocarditis			The updated Lake Louise Criteria (LLC), which include at least one T1-based and one T2-based CMR criterion for the diagnosis of myocarditis, constitutes the contemporary primary non-invasive

					stages of myocarditis				diagnostic approach when assessing patients in this clinical setting. CMR not only has proven its diagnostic use, but also has shown promising value in outcome prediction and monitoring of myocarditis.
Canty, 2022	35559722	Review	n/a	Myocardial injury	Evidence supporting the notion that circulating troponin levels parallel the extent of myocyte apoptosis in normal ventricular remodelling and in pathophysiological conditions not associated with infarction or necrosis				Interpretation: Diastolic myocyte strain induces calpain activation, myocyte apoptosis, and troponin I degradation and release in the absence of ischaemia. This appears to reflect a low rate of myocyte death that can lead to a significant loss of myocytes when the injury is chronic or recurrent.

Mair, 2018	29278915	Review	n/a	Release of cTn	Apoptosis may be considered as a mechanism for cTn release from injured myocardium	Experimental evidence suggests a number of possibilities for the extrusion of proteins from reversibly injured cardiomyocytes in the absence of cell death, including transient increases in cell permeability due to cell wounds, and the formation and release from membranous blebs or microparticles		n/a	Interpretation: cTn may also be released from reversibly injured cardiomyocytes and not only irreversibly injured myocytes (ischaemic necrosis or apotosis)
Stassi, 2022	35448711	Narrative review		Post-mortem CT and MRI in the diagnosis of cardiac causes of death	Coronary-targeted post-mortem CT angiography has shown to be useful in the detection of stenoses related to calcifications and/or atherosclerotic plaques and in the suspicion of thrombosis	Post-mortem MR proved suitable for the detection and ageing of infarcted areas, and for cardiomyopathies			Summary of the evidence on post-mortem CT and MRI imaging as alternatives or complementary approaches to standard autopsy and histologic investigations
Braunwald, 1989	2752565	Point of view	Unstable angina	Classification of unstable					Focuses on: 1) the severity of the clinical

				angina					manifestations, 2) the clinical circumstances in which unstable angina occurs, and 3) accompanied transient electrocardiographic changes
Braunwald, 2013	23775194	Review in cardiovascular medicine	Unstable angina	Impact of cTn for reclassification of patients with unstable angina				Redefinition of ACS when using cTn as a biomarker	
Eggers, 2017	28062620	Narrative review	Patients with CVD beyond ACS	Troponin elevation in non-ACS cardiac conditions	Increased cardiac troponin indicates increased risk for adverse outcomes in patients with various cardiovascular conditions beyond ACS	Routine measurement of cardiac troponin concentrations can not be generally recommended unless there is a suspicion of ACS	The finding of an increased cardiac troponin concentration in a non-ACS patient should prompt the search for possible underlying conditions		
Willinger,	33678488	Systemic review	Eight studies	Prognostic	Elevated hs-cTn T is an	Serial hs-cTn T	hs-cTn T level	Systematic	More research

2021		(no meta-analysis)	with 2162 clinically asymptomatic adults with congenital heart disease (ACHD).	value of cTn T for survival and cardiovascular events and its relation to clinical characteristics and cardiac function	independent predictor for survival and HF in stable adults with congenital heart disease	measurement was found to be beneficial compared with a single measurement	correlated with male sex, older age, and higher New York Heart Association (NYHA) class	literature search in PubMed and Cochrane from 2010 to May 2020 for hs-cTn T as a prognostic marker in ACHD	investigating the prognostic value of hs-cTn T in stable ACHD is needed and the clinical relevance to guide aftercare has still to be determined
Friden, 2017	28193484	Experimental, preclinical and clinical study	Patients with MI (*n* = 3), impaired renal function (*n* = 2), and HF (*n* = 3) for assessment of cTn T degradation products. Patients with hs-cTn T >14 ng/L without (*n* = 15 113) or with (*n* = 1746) a clinical	Assessment of renal vs. extra-renal clearance in the context of large vs. lower-grade, stable troponin-T elevation	At high cTn T levels, extrarenal clearance of cTn T dominates	GFR-adjusted hs-cTn T levels separated patients with NSTEMI slightly better than the measured value	At low cTn T levels, renal clearance also contributes to the clearance of cTn T		At high concentrations (as found after a large MI), extrarenal clearance of cTn T dominates, whereas at low levels of cTn T (often found in patients with stable cTn T elevations), renal clearance also contributes. This may explain why stable cTn T levels tend to be higher in patients with low kidney function.

			diagnosis of NSTEMI for assessment of the AUC for measured hs-cTn T.						
Muslimovic, 2020	32322013	Experimental study (rat model)	Male Wistar Kyoto rats (Harlan, Horst, The Netherlands)	Assessment of cTn cleared once it is released from damaged cardiac cells in an animal (rat) model	Positron emission tomography (PET) analyses showed that the fluorescence signal from fluorescently labelled cTn preparation and the radioactivity from the 18F-labelled cTn preparation mainly accumulated in the liver and kidneys of rats				The liver and the kidneys are responsible for the clearance of cTn from plasma in the rat
Agewall, 2017	28158518	ESC Position paper	Patients with suspected MINOCA	n/a	Definition of the clinical presentation of suspected MINOCA	Illustrated aetiologic differential diagnosis	n/a	n/a	ACS and unobstructed coronary arteries are prevalent (up to 13%) and possible causes can be coronary or cardiac vs. non-cardiac. Further testing needed to ascertain the

									diagnosis.
Collet, 2021	32860058	ESC Guideline	Patients with NSTEMI	n/a	Clinical practice guidelines on the diagnosis and management of patients with NSTEMI	n/a	n/a	n/a	CMR is in class IB in patient with suspected MINOCA

2. Evidence tables for section 14

Section	Section 14 (Biomarkers)								
**SYSTEMATIC REVIEWS AND META-ANALYSES**									
**Study first author surname, year, and acronym**	**PMID reference for study**	**Population**	**Number of participants or studies**	**Intervention**	**Key inclusion & exclusion criteria**	**Type of analysis** (random-effect, fixed-effect, Bayesian approach)	**Relevant outcome(s) and PROMS**	**Other methodological aspects**	**Key findings and interpretation**
Cao, 2024	39505672	Systematic review of reference range studies	19 studies	High-sensitivity cTn I (14 studies) or cTn T (9 studies) assays	Studies were included if they enrolled adults; were published in the English language; samples were taken as part of a healthy, reference population; studies using high-sensitivity troponin assay; and sample size >300. Studies were	Data were extracted from the studies through Covidence to perform a qualitative data synthesis	Female-specific, male-specific, and overall 99th percentiles for hs-cTn were compared		More than 90% (13 of 14 studies) of hs-cTn I assays found lower female 99th percentiles compared with male and overall 99th percentiles. Eight of nine hs-cTn T studies (88.9%) found lower female 99th percentiles compared with male and overall 99th percentiles.

					excluded if the reference population sample size was <300, if a conventional troponin assay was used, or if they did not include independently derived, sex-specific 99th percentiles.				
Denessen, 2022	36366960	Patients undergoing CABG	17 cohort studies with hs-cTn I (1661 patients) and 15 cohort studies (2646 patients)	hs-cTn I was 6.1×URL; and hs-cTn T 1.2×URL	Inclusion: patients undergoing isolated CABG and reporting h-cTn I and hs-cTn T concentrations	Random-effects modelling and post hoc subgroup analysis	Primary outcomes: post-CABG hs-cTn I and hs-cTn T kinetics; secondary outcomes: type 5 MI incidence and early mortality (in-hospital or 30-day)	None	Post-operative hs-cTn I and hs-cTn T following CABG surpass most current diagnostic cutoffs. hs-cTn I was three-fold higher than hs-cTn T and highly dependent on the assay used and surgical strategy. There is a need for assay-specific hs-cTn I and hs-cTn T cutoffs

									values for accurate identification of type 5 MI.
Chiang, 2022	34807719	Patients with suspected MI	32 studies (20 cohorts) with 30 066 patients	To evaluate the diagnostic accuracies of the ESC 0/1-h, 0/2-h, and 0/3-h algorithms	Cohorts of patients with suspected MI and available hs-cTn I (Architect, Centaur) and/or hs-cTn T values	Meta-analysis	Primary outcome index MI	None	The ESC 0/1-h and 0/2-h algorithms have higher sensitivities and NPVs than the 0/3-h algorithm for index AMI
Kimenai 2018,	29844245	Systematic review of 28 studies	Reference populations designed to establish 99th percentiles of hs-cTn I (Abbott) and/or hs-cTn T (Roche), published between January 2009 and October 2017, were	Sex differences in cardiac troponin assays	Of 16 hs-cTn I and 18 hs-cTn T studies, 14 (87.5%) and 11 (61.1%) studies reported lower female-specific hs-cTn cutoffs than overall clinical decision ranges,	Conversely, male-specific thresholds of both hs-cTn I and hs-cTn T were in line with currently used overall thresholds, particularly hs-cTn T (90% concordance)	The variation in estimated overall 99th percentiles was much higher for hs-cTn I than hs-cTn T (29.4% vs. 80.0% of hs-cTn I and hs-cTn T studies reported values within the current overall clinical decision range, respectively)	Only the hs-cTn I assay from Abbott was investigated. The statistical approach used for estimation of the 99th percentile URL was highly variable across studies.	Substantially lower female-specific URLs of hs-cTn I and hs-cTn T were observed compared with the overall or uniform decision limits for both assays. The statistical approach strongly affects the hs-cTn I threshold. Lower thresholds in women may reduce

			included. PubMed and EMBASE search for original studies, and by screening reference lists.		respectively				underdiagnosis of MI.
Chapman, 2017	29127948	Patients with suspected ACS	22 475<br><br>22 cohort studies	hs-cTn I assay	22 observational cohort studies were included.<br><br>Inclusion criteria:<br>• Patients with suspected ACS<br>• hs-cTn I (Abbott) level tested at presentation<br>• Adjudicate	Meta-estimates derived by modelling cohort-level proportions in a binomial-normal random-effects model. Odds ratios and 95% CIs were used to evaluate differences in NPV by subgroup. Heterogeneity was assessed with the I^2 index.	Primary outcome: type 1 MI or cardiac death at 30 days.<br><br>Key secondary endpoints were recurrent MI and cardiac death.	None	Among patients with suspected ACS, a low hs-cTn I concentration identified those at low risk of MI or cardiac death within 30 days.

					d diagnosis.<br>Exclusion criteria:<br>• STEMI.				
**RANDOMIZED CONTROLLED TRIALS**									
**Study first author surname, year, and acronym**	**PMID reference for study**	**Population**	**Number of participants**	**Intervention and control**	**Key inclusion & exclusion criteria**	**Type of analysis** (intention-to-treat, per-protocol, ITT or PP with adjustment for pre-specified covariates)	**Relevant outcome(s) and PROMS**	**Other methodological aspects** (allocation concealment, single/double blinding, sham procedure in device trials, missing data on outcomes, outcome central adjudication…)	**Key findings and interpretation**
Thulin, 2025<br>WESTCOR-POC trial	40202469	RCT of patients with possible ACS	*N* = 1494	0/1-h centralized hs-cTn T measurements (control) or 0/1-h point-	Inclusion: >18 years presenting to ED with possible ACS.	Modified ITT; patients previously included, transferred to another hospital, and missing a 0-h troponin measurement were	Primary: length of stay in the ED. Secondary efficacy: proportion discharged within 3 h and 6 h, and total hospital length of stay.	Single centre. No blinding was performed. Cross-over in 1 in 4 patients in the intervention arm (clinicians accessed	Implementing point-of-care hs-cTn I testing in the ED with a 0/1-h diagnostic algorithm did not lead to a clinically meaningful

				of-care hs-cTn I testing (intervention) with serial measurement in both arms	Exclusion: (1) STEMI; (2) clinically unstable; (3) comorbidities likely to compromise short-term survival; (4) transferred from other hospitals.	excluded	Secondary safety: (1) all-cause mortality and MI within 30 days and (2) all-cause mortality, AMI, and acute unplanned revascularization within 30 days.	central laboratory test to guide discharge). Intervention failed to report a result in 1 in 20 patients. Single-sample rule out not permitted in either arm of the trial.	reduction in length of stay (median reduction 6 (95% CI −4 to 17 mins). The incidence of MI, revascularization, or death was similar between the groups. Alternative pathways are needed to harness the potential for point-of-care testing to improve efficiency.
Anand, 2021	33752439	Patients with suspected ACS	31 492	Implement an early rule-out pathway where MI was excluded if hs-cTn I concentration s were <5 ng/L at presentation.<br><br>Versus standard path.	Inclusion criteria: consecutive patients presenting with suspected ACS and an hs-cTn I concentration within the normal reference range (less than the sex-	Stepped-wedge cluster RCT	Sequential hypothesis testing evaluated two co-primary outcomes in an a priori-defined hierarchical order: the primary efficacy outcome followed by the primary safety outcome. The primary efficacy outcome was length of stay, defined as the length of time from presentation to the ED until discharge from		Implementation of an early rule-out pathway for MI reduced length of stay and hospital admission. Although non-inferiority for the safety outcome was not demonstrated at 30 days, there was no increase in cardiac events at 1 year.

					specific 99th percentile URL) at presentation.<br><br>Exclusion criteria: if they presented with an out-of-hospital cardiac arrest or STEMI, had been admitted previously during the trial, or were not resident in Scotland.		hospital. The safety outcome was MI (type 1, type 4b, or type 4c) or cardiac death after discharge, which was evaluated at 30 days (primary) and 1 year (secondary) after presentation.		
Hara, 2020;<br><br>SYNTAX trial	33004127	Patients undergoing coronary revascularizat ion	1800	PCI or CABG	Inclusion criteria: patients with *de novo* three-vessel disease and/or left main stem (LMS) disease were	Modified ITT cohort consisting of patients in whom revascularization was performed according to the assigned procedure, and patients who did not receive the assigned	PMI defined as either:<br><br>1. SYNTAX<br><br>2. Fourth UDMI<br><br>3. ISCHEMIA<br><br>4. SCAI<br><br>5. EXCEL.	Recruitment period 2005 to 2007.<br><br>Prospective, multicentre, multinational (European and North American), randomized study.<br><br>All endpoints were	PMI rates according to the SYNTAX and Fourth UDMI, both of which required CK-MB elevation and electrocardiographic evidence of permanent myocardial damage, were 2.7%

					randomized in a 1:1 fashion either to receive PCI (*n* = 903) with TAXUS Express paclitaxel-drug eluting stents or CABG.<br><br>Exclusion criteria: previous PCI or CABG, acute MI, or the need for concomitant cardiac surgery.	procedure were excluded. The rates of PMI were the percentages of patients with a PMI. The cumulative incidences of death and major adverse cardiac or cerebrovascular events (MACCE) were calculated using the Kaplan–Meier method.	Key secondary endpoints:<br><br>MACCE, defined as all-cause mortality, stroke, MI, and repeat revascularization at 5 years in those with and without PMI.	adjudicated by a critical-events committee.<br><br>CK-MB rather than cardiac troponin used to define PMI.	and 3.0%, respectively, in the PCI arm vs. 2.4% and 2.1%, respectively, in the CABG arm. PMI rates according to the SCAI or EXCEL definition were higher in the PCI (5.7%) and CABG (16.5%) arms.<br><br>PMIs according to the SYNTAX and Fourth UDMI were more strongly associated with mortality than EXCEL and SCAI. PMIs defined by isolated enzyme elevation when CK-MB was more than 10x ULN. The impact of these ‘enzyme-driven events’ on time-to-event curves and the composite endpoints was greater in the surgical cohort. PMIs after PCI were

									associated with 10-year mortality regardless of definition, whereas their impact on mortality after CABG was limited to 1 year.
Chew, 2019	31478763	Patients with suspected ACS presenting to the ED	3378	Participants received care guided either by the 0/1-h hs-cTn T protocol or the 0/3-h standard masked hs-cTn T protocol	Inclusion: participants presenting to the ED if there was the intention to undertake troponin testing, and they had clinical features of chest pain or suspected ACS as the principal cause for investigation and a baseline ECG	Multicentre study evaluating the non-inferiority of a 0/1-h hs-cTn T protocol in comparison with a 0/3-h masked hs-cTn T protocol in patients with suspected ACS presenting to the ED	The 30-day primary endpoint was all-cause death and MI	None	This in-practice evaluation of a 0/1-h hs-cTn T protocol embedded in ED care enabled more rapid discharge of patients with suspected ACS

					interpreted as not definitive for coronary ischaemia, and they were ≥18 years of age and willing to give written consent.<br><br>Exclusion: if they presented for chest pain not suspected to be from a cardiac cause, presented as a result of a transfer from another hospital, presented for suspected ACS within 30 days of last presentation, required permanent dialysis, or					

					were unable to complete the clinical history questionnaire because of language or comorbidity.				
**OBSERVATIONAL COHORT STUDIES**									
**Study first author surname, year, and acronym**	**PMID reference for study**	**Population and study type** (case-control, cohort, etc.)	**Number of participants**	**Stratification**	**Key inclusion & exclusion criteria**	**Type of analysis** (covariate adjustment; propensity score matching, weighting, adjustment; trial emulation; quasi-experimental design…)	**Relevant outcomes and PROMs**	**Other methodological aspects** Bias in design (i.e. immortal-time bias, survivorship bias etc.), risk of residual confounding (i.e. e-value, falsification endpoint), other issues as evaluated in specific tools (i.e. ROBIN I), generalizability	**Key findings and interpretation**
Lowry, 2023	37350492	Patients with	*N* = 41 103	hs-cTn I at	Inclusion:	Comparisons were	Primary: Type 1 or 4b	41 103 of 48 282	In early presenters, a

		possible acute MI	consecutive patients and external validation cohort (7088 patients)	<3h (early presenters), 4–12h (reference group), and >12h (late presenters) from symptom onset	symptoms of suspected ACS.<br>Exclusion: time from symptom onset to troponin sampling was not known, had an adjudicated diagnosis of STEMI, missing presentation of hs-cTn measurement, or there was insufficient clinical information to adjudicate the diagnosis.	made between the distributions between cTn concentration at presentation and maximal concentration on serial measurements	MI during the index presentation. Secondary: Type 1 or 4b MI or cardiovascular death <30 days after the index presentation.	patients were enrolled from High-STEACS trial; external validation was performed using data from APACE study	single measurement of hs-cTn I below the limit of detection may facilitate the safe rule out of MI; the 99th percentile should not be used to rule out MI at presentation even in those presenting later following symptom onset
Hoeller, 2013	23604180	Prospective multicentre study	*N* = 2072	Patients presenting to ED with suspected	Inclusion: chest pain, angina pectoris at rest or other	Kaplan–Meier analysis for endpoint of death during follow-up and for acute MI	Primary endpoint: all cause-mortality and acute MI rate during follow-up	Enrolment: 2006–2011	Normal hs-cTn levels at presentation should not be used as a single parameter to

				ACS	thoracic sensation with an onset or peak within the last 12h.<br>Exclusion: terminal kidney failure requiring dialysis.	rate during follow-up			rule out acute MI. Lack of standardization among hs-cTn I assays can result in substantial differences in sensitivity and NPV at the 99th percentile.
Wereski, 2020	32785613	Prospective multicentre study	*N* = 48 282	Patients presenting to ED with suspected ACS	Inclusion and exclusion of patients were the same as High-STEACS cluster RCT	Comparisons between groups were performed using X^2 test for categorical variables and an unpaired t-test for continuous variables	Stratification of type 1 STEMI according to cTn at presentation based on threshold of 5 ng/mL, sex-specific 99th percentile, and the ESC 0–1 h pathway rule threshold of 52 ng/mL	Enrolment: 2013–2016	Troponin testing has limitations in the early assessment of patients with ST-segment elevation. Where clinical suspicion is high, cTn within the reference range should not delay the initiation of therapeutic agents or urgent coronary angiography.
Liu, 2022	34991787	Prospective single-centre	*N* = 70	STEMI patients	Inclusion: age >18, index	Tests were performed to assess whether the	Time for cTn I to peak with blood sampling at	Peak cTn values were clipped at 10,000 ng/L	cTn I concentrations peak earlier and

		study			STEMI.<br>Exclusion: contraindication for cardiac MRI (haemodynamic instability, GFR <30 mL/min)	time for cTn I peak were different between haemorrhagic and non-haemorrhagic MI	pre-PCI, 6 h, 12 h, 24 h, 72 h, and Days 5–7		higher in STEMI patients with haemorrhagic MI than those without haemorrhagic MI. The findings here suggest that extent of tissue injury post PCI is time dependent and reflective of tissue injury after reperfusion and that haemorrhage is a key determinant of MI size after reperfusion
Silvain, 2021	33257958	Individual patient-level data from registry studies	*N* = 9081	Patients undergoing elective PCI with normal pre-PCI baseline cardiac troponin levels	Inclusion: elective PCI for stable obstructive CAD; normal pre-PCI cTn and at least 1 post-PCI cTn measurement ≤48 h after PCI	Multivariate logistic regression analysis. Kaplain–Meier survival curves.	1-year all-cause mortality	All types of cTn assays. When multiple measurements performed post PCI, then peak values included for analysis.	Post-PCI cTn elevation ≥1x 99th URL occurred in 52.8% of patients and was not associated with 1-year mortality. Myocardial injury post PCI of ≥5x 99th URL occurred in 18.2% of patients and was associated with a two-fold adjusted odds of

									1-year mortality (2.29, 95% CI 1.32–3.97).
Vora, 2025	40858097	Prospective multicentre study and retrospective analysis of seven-hospital electronic medical records (EMR) in STEMI patients	*N* = 207 (prospective trial); *N* = 6180 from EMR	STEMI patients	Inclusion: age >18 years; index STEMI.<br>Exclusion: cardiogenic shock, presentation with cardiac arrest; contraindication for cardiac MRI, renal insufficiency, women pregnant or breastfeeding.	Concordance analysis between patients' haemorrhage status between discovery and validation cohorts; factors contributing to in-hospital mortality assessed using multivariable regression	Post PCI hs-cTn threshold for diagnosis of haemorrhagic MI and differential in-hospital mortality in those with and without haemorrhagic MI based on these thresholds	Diagnosis bases on discovery and validation cohort with hourly hs-cTn I threshold within first 24 h	STEMI patients can be accurately diagnosed for haemorrhagic MI based on post-PCI troponin kinetics. Haemorrhagic MI is a major predictor of in-hospital mortality.
Silvain, 2021	33257958	Retrospective cohort study	*N* = 9081	Fourth UDMI	Inclusion: chronic coronary syndrome patients admitted for elective PCI	Multivariate logic regression to evaluate association between each unit of ratio used as threshold 1-year mortality including following baseline	Incidences of procedural myocardial injury and type 4a MI in chronic coronary syndrome patients undergoing elective PCI; identification of	Large portion of missing PCI troponin measurements can add ascertainment bias although their baseline characteristics were similar	In chronic coronary syndrome patients with normal baseline cTN levels, the post-PCI cTn elevation of 5x the 99th percentile URL used to define

					stable obstructive CAD; baseline pre-PCI <1 times the 99th percentile of URL; at least one post-PCI cTn within the first 48 h of index procedure	clinical and angiographic characteristics: age, diabetes, active or prior smoking, hypertension, eGFR <60 mL/min, prior MI, prior CABG, multivessel stenting, number of stents – associated with 1-year all-cause mortality and procedural myocardial injury or Type 4a MI	optimal threshold for post-PCI cTn elevation for prognostically important procedural myocardial injury associated with 1-year all-cause mortality		type 4a MI is associated with 1-year mortality. This could be used to detect 'major' procedural myocardial injury in the absence of procedural complication or evidence of new myocardial ischaemia.
Miller, 2004	14718451	Non-randomized, non-consecutive cohort study in patients undergoing elective PCI	*N* = 57	cTn T and CK-MB measured at baseline, 2h, 4h, and 8h after PCI	Inclusion: patients undergoing elective PCI	Determination of peak values and times of cTn T and CK-MB from the temporal kinetics	Timing of peak and frequency of elevation in cTn T and CK-MB		cTn T elevations are more common than CK-MB elevation after elective PCI. Both biomarkers take longer to peak than anticipated clinically.
Selvanayagam, 2005	15723982	Cohort	*N* = 59		Inclusion: one of two-vessel PCI, planned	Paired t-test and unpaired-sample t-test for comparisons of	Global LV function, LVEF, areas of hyperenhancement	None	Post-procedural elevation in troponin I is correlated with new

					insertion of >30 mm of stent to a single vessel or planned treatment of a segment that involved at least one side branch 2.0 mm in size<br><br>Exclusion: lack of informed consent, contraindication to glycoprotein (GP) IIb/IIIa inhibitors, contraindication to cardiac MRI	means within the study group; binary logistic regression to determine which clinical and angiographic parameters predicted the likelihood of myocardial hyperenhancement	based on late-enhancement cardiac MRI. Identification of new hyperenhancement adjacent to stent injury and new hyperenhancement distal to the stent deemed as downstream injury.		irreversible myocardial injury on LGE MRI
Kristensen, 2024	39253802	Prospective cohort study of patients with primary	*N* = 25	No stratification was performed	STEMI with a stenosis ≥90%, ≥18 years, cTn T >500 ng/L on	Patients underwent plasmapheresis within 24 h of revascularization.	Elimination kinetics of cTn I and cTn T using five different assays	Some *in vitro* breakdown of cTn is likely prior to retransfusion; heparin	The half-life of cTn is 2–10 times shorter than previous observations, which

		MI presenting with ST-segment elevation			presentation or >1000 ng/L with 12 h of enrolment, <24 h from PCI, LVEF ≥45%, haemodynamic stability	Plasma stored for at least 3 weeks before retransfusion. After retransfusion, sampling performed at fixed intervals for 8 h to measure the change in cTn concentration.		was added to the transfusion bag which may have accelerated cleavage of cTn T (effect on cleavage of cTn I not known)	are confounded by both ongoing release and elimination.<br><br>Median elimination half-life ranged from 134 min for cTn T to 240 min for one of the cTn I assays.<br><br>Supports assumption that myocardial cTn release or washout from the infarct area is prolonged for days in the aftermath of MI.
Doudesis, 2023	37169863	Secondary analysis of a multicentre cluster RCT	Training dataset (*n* = 10 038), external validation (*n* = 10 286) patients with possible MI undergoing a single or serial hs-cTn	Stratification by age, sex, diabetes, known ischaemic heart disease, chronic kidney disease, myocardial ischaemia on	Inclusion criteria: (1) age ≥18 years; (2) presentation with suspected ACS; (3) cardiac troponin measured using hs-cTn I (Abbott) assay; (4) availability	Models trained and tested using four statistical methods—logistic regression, naïve Bayes, random forest, and extreme gradient boosting (XGBoost) to estimate the probability of type 1, type 4b or type 4c MI during the index hospital admission.	A clinical decision support tool called CoDE-ACS uses XGBoost to derive a score (0–100) that corresponds to an individual's probability of MI. CoDE-ACS had excellent discrimination for MI (AUC, 0.953), performed well across all subgroups and	Model training for only a single hs-cTn assay. Confirmation bias may in part explain the excellent performance as CoDE-ACS incorporates features that are integral to the diagnosis of MI. Although participants included from across seven countries, the	CoDE-ACS used as a clinical decision support system could reduce unnecessary hospital admissions and reduce inequalities in care that are evident using cardiac troponin thresholds to risk stratify patients with possible MI.

			I measure	the ECG, and time from symptom onset to presentation	of electrocardiographic and physiological data for diagnostic adjudication	External validation in seven international cohorts from Switzerland, Spain, Poland, Czech Republic, Australia, New Zealand, and the USA. Performance compared with guideline-recommended approach for risk stratification and diagnosis of MI.	identified more patients at presentation as low probability of having MI than guideline-recommended approaches using fixed cardiac troponin thresholds (61% vs. 27%) with a similar NPV and fewer identified as high probability of having MI (10% vs. 16%) with a greater PPV.	majority were white, and therefore diagnostic performance not evaluated across different ethnic groups. Prospective validation and implementation studies needed.	Regulatory clearance is needed before this decision support tool can be used in practice.
De Bakker, 2023	37114498	Population cohort study	*n* = 2142 and *n* = 5151 females and males, respectively	Stratification by sex	Ongoing longitudinal observational cohort study of civil servants in UK aged 35–55 when first recruited in 1985. Stored samples at three time points were available for	Using linear mixed-effects models, the sex-specific trajectories of cardiac troponin were evaluated, and the relationship with conventional cardiovascular risk factors determined.<br>Using multistate joint models, the	Cardiac troponin concentrations were persistently lower in females than males, with females exhibiting a relatively larger increase with advancing age. During follow-up, cardiac troponin concentrations were associated to the outcome in both sexes.	Males were over-represented. Study population is mainly white, reducing generalizability to other ethnic groups. Outcomes based on Hospital Episode Statistics and some misclassification likely although would affect males and females similarly. Only one	Trajectories of cardiac troponin differ between females and males in the general population, with differing associations to conventional risk factors and cardiovascular outcomes.<br>Findings highlight the importance of a sex-

					cardiac troponin testing using an hs-cTn I assay (Siemens). All participants with at least one measure of cardiac troponin were included.	association between sex-specific trajectories of cardiac troponin and a composite outcome of non-fatal MI, non-fatal stroke, or cardiovascular death was evaluated.	The slope of cardiac troponin was significantly associated with adverse cardiovascular outcomes in females, but not in males.	assay evaluated, which precludes extrapolation to other cardiac troponin assays.	specific approach when serial cardiac troponin testing is applied for cardiovascular risk prediction.
Neumann, 2023	37131096	Patients presenting to the ED with suspected MI; two ensembles of machine-learning models using single or serial concentrations of six different hs-cTn assays were derived	2575 patients with suspected MI		Inclusion: adult patients presenting to the ED with symptoms suggestive of MI. Exclusion: patients presenting with STEMI.	Discriminative performance of the models was assessed using area under the ROC curve (AUC) and logLoss. Model performance was validated in an external cohort with 1688 patients and tested for global generalizability in 13 international cohorts with 23 411 patients.	In the validation and generalization cohorts, excellent discriminative performance was confirmed, superior to hs-cTn only. For the serial hs-cTn measurement model, AUC ranged from 0.92 to 0.98. Good calibration was observed. Using a single hs-cTn measurement, the ARTEMIS model allowed direct rule-out		The development and validation of diagnostic models to accurately estimate the individual probability of MI, which allow for variable hs-cTn use and flexible timing of resampling. Their digital application may provide rapid, safe and efficient personalized patient care.

		to estimate the individual MI probability (ARTEMIS model)					of MI with very high and similar safety but up to tripled efficiency compared with the guideline-recommended strategy.		
McEvoy, 2023<br>1999–2004 National Health and Nutrition Examination Survey (NHANES)	37197846	Population cohort study	*N* = 12 545 of whom 2746 were classified as healthy	Stratification by assay for cTn T (Roche) and cTn I (Abbott, Siemens, and Ortho)	Participants >18 years old who were not pregnant from the US non-institutionalized, civilian population identified using a complex, stratified, multistage probability cluster sampling design. Only those who provided consent for storage of serum for	99th percentile URLs determined using the recommended non-parametric method	99th percentiles by age and sex strata for each assay	The average age of the population was just 37 years old and not representative of the population in whom cardiac troponin is tested. It is also much lower than in the populations tested by manufacturers. Analysis performed in frozen samples stored for 15 years, which may have resulted in degradation of cTn.	URLs for hs-troponin I assays were lower than manufacturer recommended 99th percentile URLs although the relevance of this observation is questionable given the age of the population studied. There were significant differences in hs-troponin T and I URLs by sex and by age group but none by race/ethnicity.

					future research and in whom hs-cTn measurement were performed were included. A healthy subgroup was selected using the Clinical and Laboratory Standards Institute (CLSI) and American Association for Clinical Chemistry (AACC)/IFCC guidelines.				
Lowry, 2022	36106552	Secondary analysis of a multicentre cluster RCT	*N* = 46 435 consecutive patients aged 18 to 108 years (mean, 61 ± 17	Stratification by age (<50, 50–74, and ≥75 years)	All patients attending ED or acute assessment area in whom the attending clinician	Proportion of patients with cardiac troponin above sex-specific 99th centile URL for each age category. Diagnostic performance was	In patients <50 (*n* = 12 379), 50–74 (*n* = 22 380), and ≥75 (*n* = 11 676) years, the sensitivity of the guideline-recommended	Although the study was representative of a wide range of ages the population is predominantly white, and findings may differ in a more ethnically	Age alters the diagnostic performance of cardiac troponin, with reduced specificity and PPV in older patients when

			years)		suspected ACS and underwent cardiac troponin sampling included. Patients excluded if admitted previously during the trial period or did not reside in Scotland.	assessed by using sensitivity, specificity, NPV, and PPV for UDMI recommended sex-specific 99th centile, age-adjusted 99th centile thresholds, and a universal rule-in threshold.	threshold was similar at 79.2% (95% CI 75.5–82.9), 80.6% (95% CI 79.2–82.1), and 81.6% (95% CI 79.8–83.2), respectively.<br><br>Specificity decreased with advancing age from 98.3% to 95.5% and 82.6%. Age-adjusted 99th centile thresholds improved specificity (91.3% vs. 82.6%) and PPV (59.3% vs. 51.5% for MI in patients ≥75 years, but failed to prevent the decrease in either parameter with increasing age and resulted in a marked reduction in sensitivity compared with the use of the guideline-recommended threshold, 55.9% vs. 81.6%.	diverse population. Single hs-cTn I assay. More challenging to accurately adjudicate diagnosis of MI in older population where coronary angiography performed less frequently.	applying the guideline-recommended or age-adjusted 99th centiles. Individualized diagnostic approaches rather than the adjustment of binary thresholds are needed in an ageing population.

Lam, 2022	35929566	Community cohort study	*n* = 188	None	Patients with an elevated hs-cTn I (Siemens) from a single laboratory handling samples from a community setting. There were 311 samples with values above the URL. Specimens were excluded if they could not be linked, had insufficient sample volume or were not admitted to hospital on the same day.	hs-cTn I was retested after immunoglobulin depletion and cTn measured on five other assays. Low recovery (<40%) following immunoglobulin depletion was considered as macrotroponin.	Half of cTn I elevations were found to have macrotroponins with multifactorial or indeterminate cause of myocardial injury in 56% of those with macrotroponin. ACS was three times less likely to be the diagnosis in those with compared to those without macrotroponin (9% vs. 28%). On repeat testing better diagnostic performance for ischaemic cardiac causes was observed with other hs-cTn I assays (0.74 and 0.75) compared with the Siemens assay (0.62).	Simplified approach to define elevated cTn as due to macrotroponin or not. This does not account for the heterogeneity of macrotroponin complexes and the varying degree of analytical reactivity to macrotroponin in different assays.	In a low-risk setting, the presence of macrotroponin was clinically associated with multifactorial or indeterminate causes of troponin elevation. Differences in discrimination for ischaemia between assays suggest variation in susceptibility to macrotroponin.
Harley, 2021	33761581	*In vitro* study of assay	Interference evaluated in	Stratification by assay type	Retained plasma that	Parallel cTn measurements in	All assays except Ortho showed haemolysis	Pharmaceutical-grade biotin used to simulate	Two cTn assays were susceptible to

		interference	nine hs-cTn assays		was surplus to requirement in a clinical laboratory in the USA	pooled lithium heparin plasma spiked with haemolysate and/or biotin using nine hs-cTn assays. Absolute and percent cTn recovery relative to baseline concentrations were determined in spiked samples and compared with manufacturer's claims.	and biotin interference thresholds equivalent to or greater than manufacturer's claims.<br><br>Increasing biotin concentration led to decreasing cTn recovery in two assays (Roche and Siemens).<br><br>Increasing haemolysis also generally led to decreasing cTn recovery for susceptible assays.<br><br>When biotin and haemolysis were present in combination, biotin interference thresholds decreased with increasing haemolysis for two assays (Roche and Siemens).	plasma biotin concentrations anticipated in patients taking high-dose supplements, which may not take into consideration effects of biotin metabolites so may not reflect *in vivo* interference	interference from *in vitro* haemolysis at levels routinely encountered in clinical laboratory samples leading to falsely low cTn recovery up to 3 ng/L or 13%. While most assays are not susceptible to biotin at levels expected with over-the-counter supplementation, severely reduced cTn recovery is possible at biotin levels of 10–2000 ng/mL for some assays.<br><br>Due to potential additive effects, analytical interferences should not be considered in isolation.
Vylegzhanina, 2019	30858159	Basic science study in	*N* = 38	No stratification	Plasma samples that	cTn I and cTn T concentrations in all	In patients with MI three troponin complexes	Free cardiac troponin C and long cardiac	The choice of antibody targets for

		samples from patients with primary MI undergoing coronary intervention		was performed	contained anti-troponin auto-antibodies were excluded	Lithium-heparin plasma samples were measured by in-house immunofluorescence assays (FIA) complemented by gel filtration, immuno-extraction, and chemiluminescence	were identified in plasma: (a) cTn I–cTn T–TnC (cardiac/slow skeletal isoform of troponin C) (ITC) composed of full-size cTn T and cTn I; (b) ITC with lower molecular weight (LMW-ITC) in which cTn T was truncated; and (c) a binary cTn I-cTn C complex with truncated cTn I. The amount of ITC decreased on serial testing and other fragments increased.	troponin I were not evaluated	troponin assays is important to detect cardiac troponin complex in MI. Degradation of ITC complex may be influenced by heparin and affect the performance of cTn T assays in patients with AMI.
Welsh, 2018<br>Generation Scotland Scottish Family Health Study	30126950	Population cohort study	*N* = 19 501	Stratification by age, sex, and assay type (cTn I or cTn T)	Participants identified at random from those 35 to 65 years of age at general medical practices in Scotland.	hs-cTn T (Roche Diagnostics) and hs-cTn I (ARCHITECT STAT, Abbott Diagnostics) were measured on stored samples. Associations of classical cardiovascular	Correlation between cTn T and cTn I concentrations.<br>99th percentiles by age and sex strata.	Weaknesses include the family structure although no clustering observed within families. Cross-sectional. 99th centiles for cTn T and cTn I are not biological equivalents, although	In the general population, cTn T and cTn I concentrations are weakly correlated and are differentially associated with cardiovascular risk factors. The 99th centiles currently in

					Participants asked to identify ≥1 first-degree relative 18 years or older who would be able to participate.	disease risk factors with cTn values above the sex-specific 99th percentile were evaluated. Sex-stratified cTn T and cTn I 99th centiles, along with associated bias corrected 90% CIs around the estimates, determined across age stratum. The method was repeated in those with no cardiovascular disease.		models using cTn as continuous variable supported findings.	use are broadly appropriate for men and women up to but not beyond the age of 60 years. Sex-differences in the 99th percentile are evident across all age strata.
Kaier, 2022	35149868	Patients with query ACS were recruited from the APACE study (Advantageous Predictors of Acute Coronary Syndrome	1954		All patients >18 years of age presenting to the ED with acute chest discomfort possibly indicating AMI were eligible for recruitment if the onset of	Discrimination power was quantified by the AUC for each biomarker with all cases available, using 1000 stratified bootstrap replicates to calculate CIs. Logistic regression was used to combine cardiac myosin-binding	This study assessed cMyC as an adjunct or alternative to cTn in the early diagnosis of AMI.<br>Concentrations of cMyC, and high-sensitivity (hs T and I) and standard-sensitivity cTn T and cTn I were measured at		cMyC at presentation provides discriminatory power comparable to hs-cTn T and hs-cTn I in the diagnosis of AMI and may perform favourably in patients presenting early after symptom onset

		Evaluation), an international multicentre diagnostic			or peak chest pain symptoms were within the preceding 12 h. Patients with terminal kidney failure on chronic dialysis were excluded.	protein-C (cMyC) levels with hs-cTn T, hs-cTn I, or s-cTn I values for the assessment of an incremental value using two biomarkers at presentation.	presentation.<br>The final diagnosis of AMI was independently adjudicated using all available clinical and biochemical information without knowledge of cMyC.<br>The prognostic endpoint was long-term mortality (3 years).		
Shah, 2015	25609052	Single-centre, prospective cohort study of patients with suspected ACS	*N* = 1126, 46% women	Stratification by sex	Consecutive patients presenting at ED or acute medical assessment area in whom the attending clinician suspected an ACS and measured	Hs-cTn measured in surplus plasma in real-time with results supressed and not reported to the clinician responsible for care, which was guided by the contemporary assay.<br>Two cardiologists independently	Proportion of male and female patients with myocardial injury and infarction adjudicated according to the UDMI.<br>Subsequent readmission with MI or death from any cause was recorded at 12 months through record linkage.	Single tertiary cardiac centre. However, prospective and included all consecutive patients with no case selection so more representative and generalizable than most studies. Single cTn I assay, although sex differences observed for all assays. Impact of	Although having little effect in males, an hs-cTn with sex-specific 99th percentile URLs could double the identification of myocardial injury and increase the recognition of MI in females.<br>Female patients were

					cardiac troponin. Patients not resident in Scotland were excluded.	adjudicated the diagnosis of MI by using an hs-cTn I assay (Abbott) with sex-specific diagnostic thresholds (men 34 ng/L, women 16 ng/L) and compared findings with the contemporary assay (cTn I with uniform threshold of 50 ng/L) used to guide care.		implementing sex-specific thresholds may be less in sites using the manufacturer-recommended URL for contemporary assay. Some misclassifications of MI likely as many patients with elevated cTn concentrations that were not identified in practice by the contemporary assay were reassured and discharged without investigation.	less likely to be referred to a cardiologist or undergo coronary revascularization in part because they were less likely to be recognized as having myocardial injury than males using a uniform threshold.<br><br>Female patients reclassified using sex-specific thresholds were at high risk of reinfarction and death at 1 year.<br><br>The application of sex-specific 99th percentiles has potential to reduce sex differences in inequalities in care.
Collinson, 2014	38580139	Global survey of laboratory and clinical	663 laboratories across 76	Stratification by geographic	Invitation to participate sent to members of	Data on 16 topics covering cardiac biomarker use,	Cardiac biomarker service; turnaround time and clinical	Selection bias as data only provided by those motivated to respond.	Recommendations from the Fourth UDMI adopted with the

CARMAGUE		practice	countries	region	the EFLM, IFCC, ESC ACVC, and ADLM from February 2022 to July 2023	methodology, and clinical application	decision pathways; thresholds; quality assurance	More responses from centres that provide cardiac services. Majority from European laboratories with greater representation from other regions needed to ensure generalizability.	primary diagnostic test being cardiac troponin with measurement by high sensitivity methods in 92% of laboratories. Sex-specific 99th percentile implemented in 73% (219/300) of laboratories using cTn I and 30.3% (71/234) using cTn T. A range of other cardiac biomarkers remain in routine clinical use. The clinical application of high-sensitivity troponin measurements that utilize the capacity for early diagnosis is not yet standardized or fully realized.
Kaier, 2017	28972002	Patients with query ACS were	1954		All patients >18 years of age presenting	Discrimination power was quantified by the AUC for each	This study assessed cMyC as an adjunct or		cMyC at presentation provides discriminatory power

		recruited from the APACE study, an international multicentre diagnostic study (nine study centres in Switzerland, Spain, Poland, the Czech Republic, and Italy) designed to advance the early diagnosis of AMI			to the ED with acute chest discomfort possibly indicating AMI were eligible for recruitment if the onset of or peak chest pain symptoms were within the preceding 12 h.<br><br>Patients with terminal kidney failure on chronic dialysis were excluded.	biomarker with all cases available, using 1000 stratified bootstrap replicates to calculate CIs. Logistic regression was used to combine cMyC levels with hs-cTn T, hs-cTn I, or s-cTn I values for the assessment of an incremental value using two biomarkers at presentation.	alternative to cTn in the early diagnosis of AMI.<br><br>Concentrations of cMyC, and high-sensitivity (hs T and I) and standard-sensitivity cTn T and cTn I, were measured at presentation.<br><br>The final diagnosis of AMI was independently adjudicated using all available clinical and biochemical information without knowledge of cMyC.<br><br>The prognostic endpoint was long-term mortality (3 years)		comparable to hs-cTn T and hs-cTn I in the diagnosis of AMI and may perform favourably in patients presenting early after symptom onset.
Baker, 2015	25837837	STEMI, septal ablation and CABG	60	In patients, with STEMI (*n* = 20), undergoing therapeutic ablation of septal	STEMI patients were recruited from St Thomas' Hospital, London (UK) or the coronary	Comparison of time to peak was carried out using a paired, two-tailed Student t-test after testing for normality using a Kolmogorov–Smirnov	Time to peak cMyC compared with cTn T			Following defined myocardial injury, the rise and fall in the serum of cMyC is more rapid than that of cTn T

				hypertrophy (TASH, *n* = 20) or having CABG (*n* = 20), serum was collected prospectively and frequently	care unit of the Dijon University Hospital, Dijon (France).<br>The CABG cohort comprised patients in the control group of a completed randomized trial to examine the potential cardioprotective effects of ciclosporin using biomarkers of necrosis.	test			
Kellens, 2016	25666938	This prospective single-centre cohort study was conducted at	203		Study patients were aged 18+ and presented with chest pain at the ED.<br>ACS, or suffer	ROC curve analysis with non-parametric distribution assumption was used to compare the predictive value of	Blood samples for biomarker assessment were taken immediately after PCI, 6 h later and again after 15 h. Thereafter, daily		Point-of-care H-FABP testing has lower diagnostic accuracy compared with hs-cTn T assessment in patients with high pre-

		a tertiary care hospital (Ziekenhuis Oost-Limburg, Genk, Belgium) from March 2012 until March 2013			from typical chest pain according to the treating cardiologist in the presence of at least one major cardiovascular risk factor (current smoking, diabetes, arterial hypertension and/or dyslipidaemia). Exclusion criteria were: (1) time since onset of chest pain >24 h; (2) repeat inclusion in the study; and (3) inability to provide written informed	heart-type fatty acid binding protein (H-FABP) versus hs-cTn T for MI and the combined endpoint of any cardiac diagnosis or pulmonary embolism.<br>Discriminant analysis was conducted to evaluate the added value of combining both biomarker assessments for the diagnosis of MI.	samples were taken until hs-cTn T values decreased for at least two consecutive samples. H-FABP point-of-care testing was performed concomitantly with the first three blood samples.		test ACS probability, but might be of interest when assessment is possible early after chest pain onset

					consent.				
Harada, 2018	29292015	Retrospective study of patients with NSTEMI treated with early PCI in two hospitals: Deutsches Herzzentrum München (between October 2009 and December 2014) and Bern University Hospital, Switzerland (between September 2010 and June 2014)	3783	Patients were divided into three groups: a group with post-procedural hs-cTn T in the 1st tertile (hs-cTn T <105 ng/L; *n* = 1264); a group with post-procedural hs-cTn T in the 2nd tertile (105–470 ng/L; *n* = 1258); and a group with post-procedural hs-cTn T in the 3rd tertile (>470 ng/L; *n*	NSTEMI was diagnosed in the presence of clinical symptoms suggestive of myocardial ischaemia and elevation of hs-cTn T exceeding the 99th URL.<br>The diagnosis was confirmed by angiography.	Survival analysis was performed using the Kaplan–Meier method and differences between groups were quantified by univariable Cox proportional hazards model.<br>The multiple linear regression model was used to assess variables that were independently associated with elevated post-procedural hs-cTn level. Cox proportional hazard model was used to identify the independent correlates of all-cause and cardiac mortality.	This study assessed the prognostic value of hs-cTn T in NSTEMI patients undergoing early PCI.<br>Pre-procedural and peak post-procedural hs-cTn T were measured.<br>The primary outcome was 1-year all-cause mortality.		In patients with NSTEMI undergoing early PCI, post-procedural hs-cTn T was independently associated with increased risk of mortality up to 1 year after PCI.<br>After adjustment post-procedural hs-cTn T was independently associated with the risk of all-cause mortality (adjusted HR 1.22).<br>Post-procedural hs-cTn T improved the risk prediction of the model of all-cause mortality (the C statistic increased from 0.759 to 0.772).

				= 1261).						
Coelho-Lima, 2021	34544804	Retrospective observational longitudinal study including consecutive patients with STEMI treated with primary PCI at a university hospital in the northeast of England (June 2010–December 2014).	3113		Diagnosis of STEMI was based on the presence of chest pain suggestive of myocardial ischaemia lasting longer than 30 minutes accompanied by ST-segment elevation or new LBBB on the ECG. Patients were considered for primary PCI if they presented within 12 h of symptom onset.	Comparisons in distributions of baseline variables between hs-cTn T quartile groups were performed with the Kruskal–Wallis test with Dunn's correction for multiple comparisons for continuous variables or with the X^2test for categorical variables. Survival distributions between distinct admission and 12 h hs-cTn T quartile groups were estimated by Kaplan–Meier survival analysis (log-rank test).	This study assessed the prognostic value of pre-reperfusion (admission) and post-reperfusion (12-h) hs-cTn T in patients with STEMI treated with primary PCI.<br>hs-cTn T was measured at admission to the catheterization laboratory and 12 h after primary PCI.<br>Clinical, procedural, and laboratory data were prospectively collected during patient hospitalization.<br>Mortality data were obtained from the UK Office of National Statistics. The study endpoints were in-hospital and overall mortality.			Admission, but not 12-h post-reperfusion, hs-cTn T predicted mortality and improved risk stratification in the primary PCI era.<br>These results support a prognostic role for admission hs-cTn T while challenging the cost-effectiveness of routine 12-h hs-cTn T measurements in patients with STEMI.

							Median follow-up was 53 months.		
Ndrepepa, 2018	29599097	Retrospective analysis of STEMI patients who underwent primary PCI within 24 h from the symptom onset in two university hospitals in Munich, Germany, between October 2009 and January 2016	818		STEMI was diagnosed according to the UDMI expert consensus document criteria.<br>The diagnosis was confirmed by coronary angiography in all patients.<br>Patients with cardiogenic shock were excluded.	Survival analysis was performed with the Kaplan–Meier method and the differences in survival were compared with univariable Cox proportional hazards model. Multivariable Cox proportional hazards model was used to assess correlates of 3-year mortality.	This study assessed the prognostic value of pre-procedural (admission) and peak post-procedural hs-cTn T in 818 patients with STEMI treated with primary PCI.<br>The primary outcome was 3-year all-cause mortality.		Either admission or peak post-procedural hs-cTn T were independently associated with the risk for 3-year mortality in patients with STEMI undergoing primary PCI
Reichlin, 2012	22892889	Prospective multicentre study	87		Patients with acute chest pain presenting to the ED. hs-cTn T was measured in a		AMI was the final diagnosis in 17% of patients. After applying the hs-cTn T algorithm developed in the derivation cohort to the		Using a simple algorithm incorporating hs-cTn T baseline values and absolute changes within the first hour

					blinded fashion at presentation and after 1 h. The final diagnosis was adjudicated by two independent cardiologists. An hs-cTn T algorithm incorporating baseline values as well as absolute changes within the first hour was derived from 436 randomly selected patients and validated in the remaining 436 patients. The primary prognostic endpoint was		validation cohort, 259 patients (60%) could be classified as 'rule-out', 76 patients (17%) as 'rule-in', and 101 patients (23%) as in the 'observational zone' within 1 h. Overall, this resulted in a sensitivity and NPV of 100% for rule-out, a specificity and PPV of 97% and 84%, respectively, for rule-in, and a prevalence of AMI of 8% in the observational zone group. Cumulative 30-day survival was 99.8%, 98.6%, and 95.3% (*P* < .001) in patients classified as rule-out, observational zone, and rule-in, respectively.		allowed a safe rule-out as well as an accurate rule-in of AMI within 1 h in 77% of unselected patients with acute chest pain. This novel strategy may obviate the need for prolonged monitoring and serial blood sampling in 3 of 4 patients.

					death during 30 days of follow-up.					
Shah, 2015	26454362	Prospective cohort study	6304		Patients with suspected ACS presenting to four secondary and tertiary care hospitals in Scotland. Plasma troponin concentrations were measured at presentation using an hs-cTn I assay. In derivation and validation cohorts, NPV of a range of troponin concentrations for the primary outcome of		782 (16%) of 4870 patients in the derivation cohort had index MI, with a further 32 (1%) re-presenting with MI and 75 (2%) with cardiac death at 30 days. In patients without MI at presentation, troponin concentrations were <5 ng/L in 2311 (61%) of 3799 patients, with an NPV of 99.6% (95% CI 99.3–99.8) for the primary outcome. The NPV was consistent across groups stratified by age, sex, risk factors, and previous cardiovascular disease. In two independent validation cohorts, troponin concentrations		Low plasma troponin concentrations identify two-thirds of patients at very low risk of cardiac events who could be discharged from hospital. Implementation of this approach could substantially reduce hospital admissions and have major benefits for both patients and healthcare providers.

					index MI, or subsequent MI or cardiac death at 30 days, was evaluated.		were <5 ng/L in 594 (56%) of 1061 patients, with an overall NPV of 99.4% (98.8–99.9). At 1 year, these patients had a lower risk of MI and cardiac death than did those with a troponin concentration of ≥5 ng/L (0.6% vs. 3.3%; adjusted HR 0.41, 95% CI 0.21–0.80; *P* < .0001).		
Wang, 2014	24576774	Patients admitted to Auckland City Hospital for acute coronary catheterization with a diagnosis of STEMI between October 2010 and September	173		All patients who had a hs-cTn T (Roche Elecsys 5th generation assay) measurement on admission prior to cardiac catheterization were included	Univariate analyses were performed using Mann–Whitney *U* test and Fisher's exact test or Spearman correlation co-efficient (r).<br>ROC analysis was performed for admission hs-TnT levels at detecting adverse outcomes greater than 10 cases.	This study assessed the prognostic utility of admission hs-cTn T to detect outcomes after primary angioplasty for ST-elevation/new LBBB MI (STEMI).<br><br>The primary outcome was MACE, consisting of death (all-cause), recurrent MI and repeat revascularization at 30		Admission hs-cTn T measured in primary angioplasty is strongly prognostic of MACE at 30 days and 1 year, even following adjustment for potential confounding variables

		2011 were identified, and included if hs-cTn T levels were measured at admission.<br><br>Retrospective cohort study.				C-statistics and 95% CI, and optimal cutoff points with maximum sensitivity and specificity are reported.<br><br>Stepwise multivariate analyses using logistic regression or Cox-proportional hazards regression were performed to identify predictors of MACE.	days, as per the ARC definition.<br><br>Secondary outcome measures included MACE at 1 year, death, MI and revascularization at 30 days and 1 year, ejection fraction, and length of hospital stay.		
Boden, 2013	23465094	Single-centre, retrospective cohort study of patients with STEMI who were treated with primary PCI from March 2010 to March 2011 from an ongoing	188		Consecutive patients with STEMI were eligible for inclusion when presenting with symptoms of acute MI, with an ECG demonstrating STEMI (ST-segment elevation of	The correlations were evaluated using Spearman's correlation. Linear regression analysis was performed to examine the association between the cTn T values and infarct size and LV function.	Sought to investigate the use of peak and serial 6-h fixed-time hs-cTn T for estimation of infarct size, LV function, and prognosis in consecutive patients with STEMI.<br><br>Infarct size was expressed as the 48-h cumulative creatine kinase release. LV function at 3 months		In conclusion, not only peak, but all fixed-time hs-cTn T values were associated with infarct size, LV function at 3 months, and adverse outcomes 1 year after STEMI. The value 24 h after the onset of symptoms had the closest associations with all outcomes. Therefore, serial

		clinical registry			≥0.2 mV in ≥2 contiguous leads in V1 through V3 or ≥0.1 mV in other leads) and a typical rise and/or fall course of cardiac biomarker levels.<br><br>At least one value of hs-cTnT had to exceed the diagnostic decision limit (0.05 mg/L) in the present study based on clinical experience.<br><br>The exclusion criteria were a history of acute MI and severe		was assessed using the echocardiographic wall motion score index and LVEF using radionuclide ventriculography.		sampling for a peak value might be redundant.

					renal failure (serum creatinine >2.5 mg/dL).				
Eggers, 2013	23500239	Prospective Investigation of the Vasculature in Uppsala Seniors study (PIVUS).<br>Cohort study.	Measurements were performed at baseline (*n* = 1004) and after 5 years (*n* = 814).		Community dwellers aged 70 years from the PIVUS registry	Independent predictors of baseline cTn I levels were identified by multiple linear regressions	Assess changes in troponin levels, underlying conditions, and the prognostic implications in elderly subjects from the community.<br>Total follow-up was 8.0 years.		cTn I levels were detectable in 968 (96.4%) subjects at baseline and independently predicted all-cause mortality (adjusted HR 1.44, 95% CI 1.18–1.77) and cardiovascular mortality (adjusted HR 1.66, 95% CI 1.20–2.29) when levels from baseline and 5-year follow-up were used as updated covariates
Welsh, 2019	31014085	Generation Scotland Scottish Family Health	19 501		Participants (≥18 years old) from general medical practices in	Associations of each troponin with a composite CVD outcome (1177 events), CVD death (*n*	To compare and contrast (1) the association of cTn T and cTn I with CVD and non-CVD outcomes		Both cTn I and cTn T were strongly associated with CVD risk in unadjusted models. After

		Study (GS:SFHS).<br><br>Cohort study.			Scotland between 2006 and 2010 were invited to participate	= 266), non-CVD death (*n* = 374), and HF (*n* = 216) were determined using Cox models. A genome-wide association study was conducted using a standard approach developed for the cohort.	over median of 7.8 years, and (2) their determinants in a genome-wide association study		adjusting for classical risk factors, the HR was 1.24 for cTn I and 1.11 for cTn T.<br><br>cTn I, but not cTn T, was associated with MI and coronary heart disease (CHD).<br><br>Both cTn I and cTn T had strong associations with CVD death and HF.<br><br>By contrast, cTn T, but not cTn I, was associated with non-CVD death; ratio of HRs 0.77 (0.67–0.88).<br><br>Five identified loci (53 individual single-nucleotide polymorphisms [SNPs]) that had genome-wide significant associations with cTn I, and a different set of

									four loci (four SNPs) for cTn T.
Reichlin, 2011	21709058	Prospective, multicentre study	*N* = 836	Clinical exam, physical examination, 12-lead ECG, continuous ECG, pulse oximetry, standard blood test and chest radiography. Standard cTN levels were measured at presentation and after 6–9 h.	Inclusion: patients presented to ED with symptoms suggestive of acute MI.<br>Exclusion: patients with terminal kidney failure requiring dialysis.	Two independent cardiologists blinded to cTn T or cTn I values reviewed all available medical records and acute MI was diagnosed when there was evidence of myocardial necrosis with a significant rise and/or fall consistent with myocardial ischaemia. Cardiac necrosis was diagnosed when there was at least one value of the local standard cTn >99th percentile.	Diagnostic accuracy of absolute and relative changes in cTn in patients presenting to the ED with symptoms suggestive of acute MI	Enrolment period: 2006–2009	Absolute changes of cTn levels have a higher diagnostic accuracy for acute MI than relative changes. The absolute changes are suggested to be the preferred criteria to distinguish acute MI from other causes.
**OTHER ARTICLES**									
**Study first author surname and**	**PMID reference**	**Type of article** (editorial, commentary,	**Population**	**Topics**	**Key point (1)** (evidence level if clinical	**Key point (2)** (evidence level if clinical practice	**Key point (3)** (evidence level if clinical practice	**Other considerations** (bias, uncertainty, future research	**Interpretation**

**year**	**for study**	systematic review, narrative review, clinical practice guideline, or consensus statement)			practice guideline)	guideline)	guideline)	requirements, etc.)	
Bulluck, 2022	34059914	Expert Consensus Statement (ESC Working Group on Cellular Biology of the Heart and European Association of Percutaneous Cardiovascular Interventions [EAPCI])	Procedure-related myocardial injury and infarction following PCI in CCS patients	Prognostically relevant peri-procedural myocardial injury and infarction associated with PCI	Due to lack of scientific data, the cutoff thresholds of post-PCI cTn elevation used for defining peri-procedural myocardial injury and infarction, have been selected based on expert consensus opinions, and their	Confirm the prognostic relevance of the post-PCI cTn elevation >5x 99th percentile URL threshold used to define type 4a MI	In the absence of peri-procedural angiographic flow-limiting complications or ECG and imaging evidence of new myocardial ischaemia, we propose the cTn cutoff threshold (>5x 99th percentile URL) to be used to define prognostically relevant 'major' peri-procedural myocardial injury	Further research is needed to evaluate treatment strategies for reducing the risk of major peri-procedural myocardial injury, type 4a MI, and MACE in CCS patients undergoing PCI	As both type 4a MI and major peri-procedural myocardial injury are strong independent predictors of all-cause mortality at 1 year post PCI, they may be used as quality metrics and surrogate endpoints for clinical trials

					prognostic relevance remains unclear				
Jaffe, 2014	25009249	Position paper (Acute CardioVascular Care Association)	Acute MI	Defining changes in cardiac troponin for discriminating between acute MI and other diseases	To ensure the changes in delta cTn are translatable, studies should ensure that: patients who present with chest pain should are serially enrolled; patients with STEMI and those in shock or haemodynamic compromise are not included	Patients diagnosed acutely or subsequently with other diagnoses that cause a change in cTn pattern are included as a subset	Patients should be categorized into groups based on the time of onset of symptoms that led to the patient seeking care; five additional criteria are proposed for studies		The proposed gaps in information have the capacity to overcome challenges in changing pattern of cTn values in the diagnosis of acute MI
Hollander, 2016	27528647	State of the art	ACS	Improvement in cardiac	In patients in whom	Once a process that took ≥24 h, CCTA can			

				troponin assays, especially in conjunction with well-validated clinical decision algorithms, now enables the clinician to rapidly exclude MI	unstable angina remains a concern or there is a desire to evaluate for underlying CAD, coronary computed tomography angiography (CCTA) can be used in the ED	now rapidly exclude MI and CAD in patients in the ED			
Lowry, 2021	34479874	Acute MI	To understand the evidence and guideline recommendations for use of hs-cTn with early rule- out pathways for MI	The evidence for the implementation of hs-cTn assays and early rule-out pathways for AMI was limited to observational studies where	Recent randomized trials have provided important insights into the safety and effectiveness of these pathways in	Current diagnostic and practice guidelines recommend a range of assays and validated pathways, and the choice of approach should be informed by local priorities and the multidisciplinary team, with a continuing programme of			

				care was not guided by the diagnostic approach in practice	practice	education provided to all users			
Jaffe, 2023	37380305	Review	Patients suspected of ACS and >2 h from the onset of ischaemic symptoms	Single troponin measurement to rule out MI	Rapid, safe, and accurate exclusion of AMI can facilitate triage of patients in the ED	Observational and randomized studies have found that a single low assay-dependent, hs-cTn measurement taken >2 h after symptom onset in a patient with non-ischaemic ECG can effectively exclude AMI		Additional studies are needed to assess the utility of this approach in patients undergoing evaluation earlier after the onset of symptoms to establish optimum blood level threshold for women and patients with specific comorbidities	Single-sample rule-out approach for MI needs to ensure safety first while attempting to maximize efficiency. Application of clinical judgement and presence of non-ischaemic ECG findings should be carefully considered.
Thygesen, 2012	22723599	Current opinion	AMI	Use of hs-cTn assays for accurate diagnosis of AMI and interpretation of hs-cTn values during a period of limited	Use the 99th percentile concentration of the reference population as cTn URL; diagnosis of acute myocardial	Additional testing of other biomarkers (myoglobin or CK-MB) is no longer needed; blood sampling in suspected acute MI patients should be performed at admission and 3 h later	Cardiac troponin is a marker of myocardial necrosis and not a specific marker of acute MI; stable or inconsistently variable cTn values without significant dynamic changes are likely markers of chronic		While hs-cTn assays permit measurement of cTn concentration in healthy individuals, if used appropriately they can improve acute MI care

				evidence	necrosis requires significant change with serial testing		structural heart disease		

0

3. Evidence tables for sections 12, 13, and 15

Section	Sections 12, 13, and 15 (symptoms, electrocardiogram, imaging)								
**META-ANALYSES**									
**Study first author surname, year, and acronym**	**PMID reference for study**	**Population**	**Number of participants or studies**	**Intervention**	**Key inclusion & exclusion criteria**	**Type of analysis** (random-effect, fixed-effect, Bayesian approach)	**Relevant outcome(s) and PROMS**	**Other methodological aspects**	**Key findings and interpretation**
Kumar, 2023	37080764	People with MI presenting to healthcare services	*N* = 232 519 participants from 22 studies	Aim: Compare the prevalence of chest pain in those with and without diabetes mellitus (DM) who had an MI	Absence of chest pain during MI, comparing those with and without DM.<br>After initial independent screening, 87 reports were assessed for eligibility against the inclusion criteria, leaving 22 studies.	Pooled ORs, weights, and 95% CIs were calculated using a random-effects model	Absence of chest pain during MI, comparing those with and without DM	Medline and Embase databases were searched to 18 October 2021, identifying 9272 records. After initial independent screening, 87 reports were assessed for eligibility against the inclusion criteria, quality, and risk of bias assessment (Strengthening the Reporting of Observational	There was an increased likelihood of no chest pain during an MI for those with DM, compared with those without. This was 43% higher in patients with DM in the cohort and cross-sectional studies (OR 1.43; 95% CI 1.26–1.62), and 44% higher in case-control studies (OR 1.44; 95% C: 1.11–1.87).<br>Conclusion: In patients with an MI, patients

								Studies in Epidemiology and Newcastle–Ottawa criteria)	with DM are less likely than those without to have chest pain recorded.
Van Oosterhout, 2020	32363989	Studies that reported on symptom presentation in both women and men with confirmed ACS	*N* = 1 226 163	Aim: To assess the extent of sex differences in symptom presentation in patients with confirmed ACS	Included all studies that reported on symptom presentation in both women and men with confirmed ACS	Aggregated and cumulative meta-analyses were performed for both the crude and adjusted OR using a random-effects model with inverse variance weighting. To assess when a possible significant observed sex difference was established in literature, a cumulative meta-analysis was performed for symptoms that showed a significant sex difference in the	Compared with men, women with ACS had higher odds of presenting with pain between the shoulder blades (OR 2.15; 95% CI 1.95–2.37), nausea or vomiting (OR 1.64; 95% CI 1.48–1.82), and shortness of breath (OR 1.34; 95% CI 1.21–1.48).<br>Women had lower odds of presenting with chest pain (OR 0.70; 95% CI 0.63–0.78) and diaphoresis (OR 0.84; 95% CI 0.76–0.94).<br>Both sexes presented most often with chest pain (pooled	An adapted version of the Newcastle–Ottawa Scale was used to assess the quality of the included studies. The scoring system is based on three components: selection, comparability, and outcome.	Women with ACS do have different symptoms at presentation than men with ACS, but there is also considerable overlap. Since these differences have been shown for years, symptoms should no longer be labelled as ‘atypical’ or ‘typical’.

						aggregated meta-analysis.	prevalences, men 79%, 95% CI 72–85; pooled prevalences, women 74%, 95% CI 72–85).<br>Other symptoms also showed substantial overlap in prevalence. The presence of sex differences has been established since the early 2000s.		
Diao, 2017	28855658	Patients with MI	11 trials with 765 patients were included	Global longitudinal strain (GLS) using speckle tracking echocardiography	The studies quantitatively estimated the correlation between strain results analysed by echocardiography and scar size measured by CMR	PRISMA	The pooled correlation was 0.7 between two-dimensional GLS and LGS, and 0.55 for 3D GLS		This approach can serve as a good diagnostic index for assessing infarction area
Jinatongthai, 2017	28831992	Adults with STEMI	128 071 participants, 40 trials	Fibrinolytic therapy with streptokinase, tenecteplase, reteplase, and	Inclusion: RCTs of fibrinolytic therapy in STEMI patients. Exclusion: Trials without mortality	A direct meta-analysis was used to pool RRs using a random-effects model. To rank the	The primary efficacy outcome of interest was all-cause mortality within 30–35 days.	Inclusion criterion for reperfusion in the RCTs was ST-segment elevation	Accelerated infusion alteplase plus parenteral anticoagulants reduced the risk of mortality by

				alteplase either alone, or in combination, compared with other fibrinolytics, placebo or no treatment	outcomes or non-comparable dosing.	intervention hierarchy in the network meta-analysis, the rankograms, surface under the cumulative ranking (SUCRA) curves, and mean ranks were estimated.			0.79 (95% CI 0.69–0.91) compared with streptokinase and parenteral anticoagulants, and by 0.88 (0.81–0.95) compared with non-accelerated infusion alteplase plus parenteral anticoagulants
Vyas, 2022	39132339	STEMI patients	18 studies totalling 2824 patients	PCI	RCTs, cohort studies, case-control studies, and case series of patients with access to determination of intramyocardial haemorrhage (IMH)	Random-effects model to calculate the aggregated OR and corresponding 95% CI for MACE, all cause-mortality, and TIMI 3 flow after PCI	Outcomes studied were MACE, all-cause mortality, MI size, LV end diastolic volume (EDV), LVE,F and presence of TIMI <3 after PCI	None	IMH is (i) present in ~40% of STEMI patients; (ii) associated with MACE in STEMI population; (iii) associated with large MI size, low LVEF, and large LV EDV; more frequent TIMI <3 after GP IIb/IIIa inhibitors; and is predictable in males sex, left anterior descending infarct, and smokers

Mileva, 2023	36889851	Patients with MINOCA	26 studies comprising 3624 patients	Determine the diagnostic and prognostic value of CMR in the management of patients with MINOCA	The inclusion criteria were as follows: 1) studies comprising patients with a working diagnosis of MINOCA; 2) undergoing a CMR assessment within 10 days from the index acute event; and 3) reporting the number of patients diagnosed with a specific disease entity and the total number of patients evaluated	PRISMA<br>QUADAS<br>Random-effect model	The primary outcome of interest was the prevalence of confirmed MI, myocarditis, Takotsubo syndrome, or normal findings according to CMR imaging in patients with an initial working diagnosis of MINOCA		MINOCA was confirmed in only 22% (95% CI 0.17–0.26) of the cases and 68% of patients with initial MINOCA were reclassified after the CMR assessment. The pooled prevalence of myocarditis was 31% (95% CI 0.25–0.39), and Takotsubo syndrome 10% (95% CI 0.06-0.12).
**RANDOMIZED CONTROLLED TRIALS**									
**Study first author surname, year, and acronym**	**PMID reference for study**	**Population**	**Number of participants**	**Intervention and control**	**Key inclusion & exclusion criteria**	**Type of analysis** (ITT, PP, ITT or per-protocol with adjustment for pre-specified covariates)	**Relevant outcome(s) and PROMS**	**Other methodological aspects** (allocation concealment, single/double	**Key findings and interpretation**

								blinding, sham procedure in device trials, missing data on outcomes, outcome central adjudication…)	
Puchner, 2014	25125300	Patients with chest pain, a clinical suspicion for ACS, and cardiovascular risk factors presenting to the ED	472	CT coronary angiography (CTCA) vs. invasive angiography (standard care arm)	Patients with chest pain, a clinical suspicion for ACS, and cardiovascular risk factors presenting to the ED.<br>Exclusion criteria: patients not undergoing CTCA and non-diagnostic image quality.	multivariable logistic regression analyses, AUC) using the DeLong algorithm	To determine whether high-risk plaque features, as detected by CTCA in the ED, may improve diagnostic certainty of ACS independently and incrementally to the presence of significant CAD and clinical risk assessment in patients with acute chest pain but without objective evidence of myocardial ischaemia or MI	Qualitative assessment of stenosis and high-risk plaque	In patients presenting to the ED with acute chest pain but negative initial ECG and troponin, presence of high-risk plaques on CCTA increased the likelihood of ACS independent of significant CAD and clinical risk assessment
Gray, 2021	34588162	Adults with suspected or a provisional diagnosis of	1748	Early CTCA and standard of care compared with	Symptoms of suspected ACS or those with a provisional	Cox proportional hazards regression adjusted for study site (used to stratify	Primary endpoint was all-cause death or subsequent type 1 or		In intermediate-risk patients with acute chest pain and suspected ACS, early

		ACS and one or more of previous coronary heart disease, raised levels of cTn, or abnormal ECG		standard of care only	diagnosis of ACS and one or more of previous coronary heart disease, raised levels of cTn, or abnormal ECG	the randomization)	4b MI at 1 year.		CTCA did not alter overall coronary therapeutic interventions or 1 year clinical outcomes, but reduced rates of invasive angiography while modestly increasing length of hospital stay
**OBSERVATIONAL COHORT STUDIES**									
**Study first author surname, year, and acronym**	**PMID reference for study**	**Population and study type** (case-control, cohort, etc.)	**Number of participants**	**Stratification**	**Key inclusion & exclusion criteria**	**Type of analysis** (covariate adjustment; propensity score matching, weighting, adjustment; trial emulation, quasi-experimental design…)	**Relevant outcomes and PROMs**	**Other methodological aspects** (bias in design (i.e. immortal-time bias, survivorship bias, etc.), risk of residual confounding (i.e. e-value, falsification endpoint), other issues as evaluated in specific tools (i.e. ROBIN I), generazibility)	**Key findings and interpretation**

Cho, 2025	40703113	Single-centre derivation cohort study	*N* = 300 627	Diagnosis of MI (stratified by ST-segment elevation or no ST-segment elevation)		Transformer-based deep learning model trained on the ECG	Index diagnosis of AMI	External validation in independent centre	By integrating self-supervised learning to train the artificial intelligence (AI) model, AMI detection performance was enhanced, as demonstrated by an increase in the area under the ROC curve (AUROC) from 0.910 (95% CI 0.904–0.915) to 0.968 (95% CI 0.965–0.971) in the external validation set. For STEMI and NSTEMI detection, the AUROCs were 0.991 (95% CI 0.989–0.993) and 0.947 (95% CI 0.942–0.952) in the external validation set, respectively.
Angerud, 2019	29111768	Multicentre, observational study included MI	*N* = 694	STEMI vs. NSTEMI	Eligible: patients (a) with a diagnosis of STEMI or NSTEMI; (b) who were willing	A multiple logistic regression model was used to determine the	STEMI, NSTEMI, age, cold sweat, anxiety, nausea, blood pressure, sex, numeric	Data were collected using a previously validated questionnaire. In	Patients with STEMI differed from those with NSTEMI in symptom presentation, first

		patients from five hospitals			to participate; (c) who were able to fill in the questionnaire; and (d) haemodynamically stable and pain-free	association between type of MI and symptom presentation, adjusting for sociodemographic characteristics and comorbidities. Inclusion in the multiple logistic regression model was restricted to independent variables associated with the dependent variable at the significance level of 0.10, with the exception of age and sex, which were forced into the model.	rating scale (NRS), jaw or teeth pain, shoulder pain, stroke, smoking, 'come and go' pain, MI, and symptoms	collaboration with the original developer, both a new literature search and expert validation were conducted. This validation procedure resulted in minor changes. The modified questionnaire included 35 items, covering four domains: (a) background characteristics; (b) symptoms; (c) course of events; and (d) mode of transport to hospital.	medical contact (FMC) and pre-hospital delay times. Even though chest pain was just as common in STEMI as in NSTEMI, STEMI patients were more likely to have a cold sweat and to suffer from nausea or jaw pain. Use of emergency medical services (EMS) was higher among patients admitted with STEMI. The pre-hospital delay time from symptom onset to FMC was significantly longer in NSTEMI (2:05 h vs. 1:10 h, P = .001).
Ferry, 2019	31431112	Prospective observational cohort study of adult patients	N = 1941 patients (39% women)	Women vs. men; 'typical' vs. 'atypical' symptoms. Typical pain	All patients aged 18+ in whom the attending clinician requested cardiac troponin for	Likelihood ratios (LRs) with 95% CIs were calculated to assess the predictive value of	Patient-reported symptoms	All patients with cardiac troponin I concentrations above the sex-specific 99th percentile were	Typical symptoms are more common and have greater predictive value in women than in men with MI whether or

		presenting to ED in whom troponins were ordered		was classified in patients reporting the presence of chest, arm, or jaw pain with descriptors of dull, heavy, tight, pressure, ache, squeezing, crushing, or gripping.	suspected ACS were eligible for inclusion.<br>Excluded were patients with STEMI, those who were unable to provide consent, or those with previous involvement in the trial.	typical symptom characteristics (pain nature, pain location, radiation, additional features) for the diagnosis of type 1 MI in men and women.<br>Multivariate logistic regression modelling was then used to calculate ORs with 95% CIs for the number of typical features present based on the categorization.		adjudicated and classified as having type 1 MI, type 2 MI, or myocardial injury, in accordance with the Third UDMI	not they are diagnosed using sex-specific criteria
King-Shier, 2019	30867199	Cross-sectional survey.<br>Patients hospitalized at one of 12 hospitals in Canada with a diagnosis of ACS.	*N* = 1334:<br>*n* =30 white, *n* = 488 South Asian, *n* = 216 Chinese	By ethnicity	Inclusion criteria were age >19 years; admission to hospital with a confirmed diagnosis of ACS.<br>Self-reported ethnicity for either white (European),	Descriptive statistics and X^2tests were used to identify differences in participant characteristics, as well as symptoms and location(s) of pain/discomfort	The presence of classic (typical) ACS presentation was defined as having midsternal or midsternal with radiating left neck, shoulder or arm pain/discomfort of any	Enrolment occurred from September 2010 to December 2015	Most common presenting symptom across ethnic groups was midsternal pain/discomfort of any intensity (89.3%) followed by left shoulder pain/discomfort (46.6%).

					South Asian, or Chinese	between ethnic groups	or at least moderate intensity		>78% of all patients reported the classic presentation of at least moderate intensity midsternal pain/discomfort.<br>The majority of patients described their pain as pressure, squeezing, or burning (81.3% whites).<br>Other symptoms prevalent among all ethnic groups: shortness of breath (62.4%), diaphoresis (49.8%), dizziness (49.3%), and nausea/vomiting (33.1%).
Nanna, 2019	31607145	Prospective observational study.<br>Participants enrolled in SILVER-AMI (Comprehens	*N* = 3041	Stratified by AMI subtype, STEMI and NSTEMI	Adults hospitalized for AMI	Categorical variables expressed as percentages and continuous variables as means.	Evaluated for sex-based differences in clinical presentation, functional impairments, management, and in-hospital complications	The in-hospital assessment (baseline interview) was complemented with a detailed medical record abstraction	Among older adults hospitalized with AMI, women had a higher prevalence of age-related functional impairments and, among the STEMI

		ive Evaluation of Risk Factors in Older Patients With Acute Myocardial Infarction), consisting of older patients (44% women) hospitalized for AMI.				Chi-squared or Fisher's exact test for categorical variables and the t-test or Wilcoxon rank sum test for continuous variables.<br>Adjustment for multiple comparisons was conducted using the Benjamini–Hochberg approach to multiple testing for the three bleeding sub-groups (major bleeding events, non-major bleeding events, and bleeding events following PCI), in both the NSTEMI and STEMI strata.		performed by a site research coordinator that included details of initial presentation (blood pressure, heart rate), comorbidities, laboratory results, and in-hospital adverse events	subgroup, a higher incidence of overall bleeding events, which was driven by higher rates of non-major bleeding events and bleeding following PCI
Bjorck, 2018	30564376	Observational study	*N* = 172 981 patients	With chest pain versus	All AMI cases registered in the	HRs with 95% CIs were obtained from	Chest pain and 5-year mortality rates	All data were from the national quality of	Absence of chest pain in patients with AMI

			(33.5% women)	without chest pain	SWEDEHEART registry between 1996 and 2010	Cox regression to assess the relative risk of mortality over time for patients with AMI without chest pain relative to those with chest pain.<br>The incidence rate for death was calculated as the ratio of death and person-years of follow-up. Rates were calculated for the age groups 18–64 and 65–84 years by symptoms. All-cause mortality and 30-day, 1-year, and 5-year mortality rates were calculated within each age group in relation to symptoms. 5-year survival was		care register SWEDEHEART. The register includes all patients who are treated for ACS in coronary care units or other specialized facilities in Sweden. The register started in 1995 with 19 participating hospitals, increasing gradually to 72 hospitals, and currently includes all Swedish hospitals treating acute cardiac diseases. The register includes information on clinical characteristics, type of infarction, symptoms, diagnosis, medication, and procedures on admission, during	was associated with more complications and higher short-term and long-term mortality rates, particularly in younger patients, and in those without previous CVD

						estimated by the Kaplan–Meier method. The log-rank test was conducted to compare survival at 30 days, 1 year, and 5 years between patients with AMI presenting with or without chest pain.<br><br>Logistic regression models were used to evaluate symptom differences in older and younger women, adjusting for ACS diagnosis, functional status, body mass index (BMI), and comorbid conditions. Analyses of symptoms were stratified by age,		hospitalization and at discharge. The register is monitored regularly with a 95%–96% agreement between the registry and health records.<br><br>Symptoms are subjective.	

						and interaction of symptom by age was tested.			
DeVon, 2018	30237934	Patients were enrolled at five large referral centres in the Midwest, West, South-west, and Pacific Northwest regions of the United States.<br>Prospective, observational study.	*N* = 400 women	Two groups: 237 women <65 years and 163 women ≥65 years	Women presenting to the ED with symptoms triggering a cardiac evaluation, 21 years old, fluent in English, and who arrived by private transportation or EMS were eligible	Multivariable hierarchical logistic regression models, which accounted for clustering effects of patients within sites (using a random effect) and adjusted for sociodemographic and clinical characteristics, to evaluate the relationship between sex and AMI presentation without chest pain, pressure, tightness, or discomfort for the overall cohort and among pre-specified subgroups defined	Symptoms of ischaemia/angina	Subjective data	Older women were more likely to have hypertension, hypercholesterolaemia, never smoked, lower BMI, more comorbid conditions, and lower functional status.<br>Younger women had higher odds of experiencing chest discomfort, chest pain, chest pressure, shortness of breath, nausea, sweating, and palpitations.

						by type of AMI and age			
Lichtman, 2018	29459463	The observational VIRGO study recruited patients hospitalized with AMI between 21 August 2008 and 5 January 2012 from 103 geographically diverse academic and non-academic hospitals across the United States.<br>Used a 2:1 female: male enrolment design.	*N* = 2985	Women vs. men.<br>Chest pain vs. no chest pain.<br>In post hoc comparisons, data were stratified and analysed by symptom duration >1 day, prior CAD/angina (prior AMI, PCI, CABG surgery, or angina), education beyond high school, and diabetes mellitus.	Eligible patients were 18 to 55 years of age and had increased cardiac biomarker levels, with at least one of these biomarkers >99th percentile of the URL at the recruiting centre within 24 h of admission. Additional evidence of acute myocardial ischaemia was required,	A logistic regression model was constructed to determine if symptoms were predictive of an ACS diagnosis and an interaction term for Black race by each symptom was added.	Patient-reported information on symptoms experienced before hospital presentation, perceived cause of symptoms, and prior interactions with the healthcare system for similar symptoms	Insufficient number of other minority groups (besides Blacks) to include in the analysis.<br>Generalizable to Blacks and Whites only.	Presentation of AMI symptoms was similar for young women and men, with chest pain as the predominant symptom for both sexes. Women presented with a greater number of additional non-chest pain symptoms regardless of the presence of chest pain, and both women and their healthcare providers were less likely to attribute their prodromal symptoms to heart disease in comparison with men.

DeVon, 2014	24992880	Observational study	*N* = 663	Non-Hispanic Blacks compared with non-Hispanic Whites	Included if presenting to the ED with symptoms triggering a cardiac evaluation, 21 years old, fluent in English, and arrived by private transportation or EMS.<br>Excluded if they had an exacerbation of HF, were transferred from a haemodialysis facility, were referred for evaluation of a dysrhythmia, or had cognitive impairment.		PROMs: Symptoms of ischaemia/angina	Symptoms are subjective	Blacks with confirmed ACS were younger; had lower income; less education; more risk factors; more symptoms, and longer pre-hospital delay at presentation compared with Whites.<br>Blacks experiencing palpitations, unusual fatigue, and chest pain were more than three times as likely as Whites to have ACS confirmed.<br>Blacks with ACS had more clinic visits and more symptoms 1 month following discharge.<br>Significant racial disparities remain in clinical presentation and outcomes for Blacks compared with Whites presenting to

									the ED with symptoms suggestive of ACS.
DeVon, 2014	24695650	Prospective, observational study	*N* = 736<br>Patients (272 women, 464 men) admitted to the ED with symptoms suggestive of ACS and receiving a cardiac work-up	Ruled in for ACS vs. ruled out for ACS	Age ≥21 years.<br>Cardiac evaluation in the ED.<br>HF.<br>Were eligible if fluent in English and arrived by private transportation or EMS.<br>Excluded if they had an exacerbation of HF, were transferred from a haemodialysis facility, were referred for evaluation of a dysrhythmia, or had cognitive impairment.	Sensitivity defined as the probability of the presence of a symptom among patients with a confirmed diagnosis of ACS. Specificity defined as the probability of the absence of a symptom among patients ruled out for ACS. Sensitivity and specificity computed for each of the 13 symptoms.<br>Mixed-effects logistic regression models, including random intercepts to control for site differences, were used to estimate the sensitivity and	PROMs: Symptoms suggestive of MI		Chest pressure, chest discomfort, and chest pain demonstrated the highest sensitivity for ACS in both women (66%, 66%, and 67%) and men (63%, 69%, and 72%).<br>Six symptoms were specific for a non-ACS diagnosis in both women and men.<br>The predictive value of shoulder (OR 2.53; 95% CI 1.29–4.96) and arm pain (OR 2.15; 95% CI 1.10–4.20) in women was nearly twice that of men (OR 1.11; 95% CI 0.67–1.85 and OR 1.21; 95% CI 0.74–1.99). Shortness of breath (OR 0.49; 95% CI 0.30–0.79)

						specificity of each symptom for a confirmed ACS diagnosis.<br>Models were tested for the entire sample and then stratified by sex.			predicted a non-ACS diagnosis in men.
Canto, 2012	22520522	Observational study using National Registry of Myocardial Infarction (NRMI) data	*N* = 1 143 513 patients with MI from 1994 to 2006	STEMI vs. NSTEMI and chest pain vs. no chest pain	Included: patients admitted with MI at 1977 hospitals.<br>Excluded: (1) all transferred patients because of incomplete reporting, (2) patients with a secondary diagnosis of MI, and (3) patients with missing information on sex, age, or symptom presentation of chest pain or discomfort.	Multiple logistic regression models with mortality as outcome were stratified by (1) MI type and (2) atypical presentation (four models in total) and included baseline demographics, presenting characteristics, invasive cardiac procedures (coronary angiogram, PCI, CABG), pharmacologic	Chest pain at presentation, MI type, hospital care, and mortality	The NRMI was funded by Genentech (Roche) and represents the largest registry of its kind in the world, which has enrolled 2 160 671 patients admitted with MI at 1977 hospitals from 1994 to 2006. The diagnosis of acute MI was based on a clinical presentation consistent with acute MI at the time of hospitalization and was determined by each local hospital.	Hospitalized patients with NSTEMI were nearly two-fold more likely to present without chest pain than patients with STEMI.<br>Patients with MI without chest pain were less quickly diagnosed and treated and had higher adjusted odds of hospital mortality, regardless of whether they had ST-segment elevation.

						treatments received within 24 h, hospital characteristics, and the year a patient was enrolled in the study (the latter to account for temporal changes in care and definition of MI)			
Kirchberger, 2011	21338424	Observational study	*n* = 889 with STEMI and *n* = 1268 with NSTEMI	STEMI vs. NSTEMI	Since 1 January 2001, all patients with AMI diagnosed according to the ESC and ACC criteria have been included in the Coronary Event Registry	The relationship between AMI type (STEMI and NSTEMI) and the occurrence of the 13 symptoms was examined by multiple logistic regression modelling. Models were adjusted for clinical characteristics and symptoms.	Patient-reported symptoms on MI	Patients were interviewed during their hospital stay after transfer from the intensive care unit using a standardized questionnaire. The interviews were made by trained study nurses and included questions about demographic characteristics as well as cardiovascular risk factors, medical	Only half of all patients correctly attributed their symptoms to the heart. Patients with STEMI and NSTEMI showed differences regarding several presenting symptoms. Further research is needed to replicate the results, and public awareness of AMI symptoms needs to be improved.

								history, comorbidities (including diabetes), and symptoms associated with the AMI event	
Geers, 2024	8860493	Cohort study	*N* = 2157		Patients with ACS	Kappa statistics	Agreement between hand-held echo and TTE was good to excellent (Kappa 0.6–1.0).<br>Hand-held echo had an NPV of 95%.		In patients with ACS, hand-held echocardiography provides comparable results to TTE
Thune, 2006	17138030	Cohort study	*N* = 262		Patients with LV dysfunction, HF or both after MI in the Valsartan in Acute MI Trial (VALIANT)	AUC, accuracy, sensitivity, and specificity of the machine learning system	LVEF and wall motion index (WMI) were independent predictors of adverse outcome after MI		Assessment of regional dysfunction by WMI has slightly more prognostic value than LVEF in patients with LV dysfunction, HF, or both after MI.<br>Regional assessment might be a more sensitive predictor of outcome than global assessment in patients with MI.

Korosoglou, 2005	16188510	Cohort study	*N* = 610		Patients undergoing elective PCI	Diagnostic performance was assessed by sensitivity, specificity, ROC, and performance recall analysis and t-distribution tests	Eight patients with elevated troponin T demonstrated impaired microvascular integrity		Impaired microvascular integrity is partially present in patients with stable angina post PCI. It is partially reversible and closely related to the release of troponin T. Myocardial echo contrast echocardiography may be useful to monitor such alterations in myocardial tissue perfusion.
Zamorano, 1997	9043850	Cohort study	*N* = 19		Patients undergoing cardiac catheterization	AUC	There was good correlation between the time intervals assessed haemodynamically and those based on the different velocity interfaces obtained with M mode tissue Doppler echocardiography		Tissue Doppler echocardiography has the potential to accurately measure the different phases of the cardiac cycle, which until now could only be determined invasively
Laumer, 2022	35353118	Cohort study	*N* = 224		TTE results of patients with AMI		This approach achieved an AUC of		A real-time system for fully automated

					from the Zurich ACS registry and patients with Takotsubo syndrome from seven centres in the International Takotsubo Registry		0.79 and an overall accuracy of 74.8.<br>The cardiologists achieved a mean AUC of 0.71 and an accuracy of 64.4.		interpretation of echocardiograms was established and trained to differentiate Takotsubo from AMI
Upton, 2021	34922865	Dataset from the EVAREST trial (Echocardiography Value and Accuracy at REst and STress)	578 studies		Stress echocardiogram collected as a part of large UK-based prospective multicentre, multivendor study		Acceptable classification accuracy for identification of patients with severe CAD was achieved with a specificity of 92.7% and a sensitivity of 84.4%		Automated analysis of stress echocardiograms is possible using AI and the provision of automated classifications to clinicians when reading stress echoes could improve accuracy, inter-reader agreement, and reader confidence
Kusunose, 2020	31103590	Cohort study	*N* = 300		History of MI		For the detection of wall motion abnormalities, the AUC produced by the deep learning algorithm was similar to that produced by the cardiologists and sonographer		The results support the possibility of using a deep convolutional neuro network for automated diagnosis of regional wall motion abnormalities in the

							readers		field of echocardiography
Spirito, 2023	36725173	Cohort study of consecutive patients with unconscious out-of-hospital cardiac arrest (OHCA) without ST-segment elevation who were undergoing coronary angiography, at Bern University Hospital, Switzerland	*N* = 386	Patients with and without acute coronary occlusion on coronary angiography	Unconscious OHCA patients without ST-segment elevation	Multivariable logistic regression comparing patients with AMI with and without chest pain. Adjustment was made for sex, age, year of hospitalization, and comorbidities. Interaction analysis for sex.	Frequency and predictors of acute coronary occlusions and their impact on all-cause and cardiovascular mortality at 1-year using logistic regression model	Enrolment 2011–2019; consecutive patients	In patients with OHCA acute coronary occlusions found in 19.5% of stable and 24.0% of unstable patients (who did not have ST elevation). Acute coronary occlusion associated with increased risk of cardiovascular death (adjusted HR 2.74; 95% CI 1.22–6.15) but not of all-cause death.
Sgarbossa, 1996	8559200	Patients enrolled in the GUSTO-1 (Global Utilization of	*n* = 131 (0.5%) of 26 003 North American patients from	Patients with LBBB and AMI, compared with patients with	Only patients with LBBB on ECG included	Univariate and multivariable analysis to identify ECG features associated with	Primary outcome of AMI	Blinded assessment of ECGs. Diagnostic performance of model tested in an independent	Three electrocardiographic criteria independently predicted the diagnosis of AMI in

		Streptokinase and Tissue Plasminogen Activator for Occluded Coronary Arteries) trial who had LBBB and AMI, compared with control patients with chronic CAD and LBBB	the GUSTO-1 trial	LBBB and chronic CAD (no AMI)		AMI. ROC curves to evaluate diagnostic performance.		population of *n* = 45 patients with LBBB, acute chest pain and high likelihood of CAD (validation sample).	patients with LBBB: ST-segment elevation of 1 mm or more that was concordant with (in the same direction as) the QRS complex; ST-segment depression of 1 mm or more in lead V_1, V_2, or V_3; and ST-segment elevation of 5 mm or more that was discordant with (in the opposite direction from) the QRS complex
Smith, 2012	22939607	Case control study, three institutions	33 patients with an acutely occluded artery and LBBB; 129 control patients with LBBB without coronary occlusion	Patients with LBBB who met Sgarbossa criteria versus Smith–Sgarbossa criteria (ST-segment elevation with an ST/S ratio ≤ −0.25 instead	All patients had coronary angiography	Area under the ROC curve. McNemar's rule to compare sensitivity and specificity between pairs of rules with 95% CIs.	Acute coronary occlusion	ECGs obtained from 1994–2008. ECG measurements blinded to outcome.	Smith–Sgarbossa criteria more accurate than both the weighted (16% difference; 95% CI 5%–27%) and unweighted (12% difference; 95% CI 2%–22%) Sgarbossa criteria

				of ST-segment elevation ≥5 mm)					
Widimsky, 2012	21890488	Cohort study, retrospective registry	6742 patients with AMI at eight hospitals	Patients with AMI and new/presumed new right bundle branch block (RBBB) (irrespective of ST-segment deviations), compared with patients with AMI without new RBBB	Patients without coronary angiography excluded	Multiple logistic regression and Cox's proportional hazards models used. Adjusted for age, sex, indicators of diabetes, previous MI, Killip class.	Occluded coronary artery (TIMI 0 flow). Secondary outcome of PCI use, mortality outcome.	Enrolment 2006–200.	Occluded coronary artery occurred in a high proportion of patients with AMI and new/presumed new RBBB (51.7%). Primary PCI was performed in 80.1% of RBBB patients vs. 68.3% of LBBB patients (adjusted OR 1.71; 95% CI 1.19–2.45).<br><br>In-hospital mortality of patients with AMI and RBBB was similar to patients with AMI and LBBB.
Krone, 1983	6869266	Cohort study	593 consecutive patients aged ≤75 with first MI	Patients with and without Q waves on ECG after the MI	Aged ≤75 years with first MI. Excluded if LBBB.	Breslow's test for equality of survivorship functions. Multivariable analysis using	Primary outcome of cardiac mortality at mean follow-up post discharge	Enrolment 1971–1975, consecutive patients	Patients with Q-wave MI had higher cardiac mortality compared with non-Q wave MI

						Cox regression model.			
Raitt, 1995	7897120	Pooled cohort from four RCTs of thrombolytic therapy	695	Patients with no past history of MI with an admission ECG	Excluded if admission ECG could not have a Aldrich score calculated due to bundle branch block or left ventricular hypertrophy (LVH)	Two-tailed and X^2tests to assess differences in baseline variables. Analysis of variance used to determine statistical significance of the effects of time to therapy and the number of abnormal Q waves on infarct size.	Presence/absence of Q waves. Infarct size measured by radionuclide studies.		Of patients admitted within 1 h of symptoms, 53% had abnormal Q waves on ECG. Infarct size larger in patients with abnormal Q waves but the presence of Q waves did not eliminate the effect of thrombolytic therapy on reducing infarct size.
Delewi, 2013	23433932	Cohort study, seven Dutch primary PCI centres	184 patients with STEMI treated with primary PCI	Patients with and without Q waves on ECG, defined according to classic criteria versus the TIMI criteria and 2000 and 2007 consensus	Patients with STEMI treated with primary PCI. Exclusion if prior MI.	ROC curves were constructed to assess association of Q-wave MI distinction based on the four different criteria with infarct size	Infarct size on CMR at 4 ± 2 days after reperfusion and repeated after 4 and 24 months	No adjustments for multiple comparisons were made	The classic ECG criteria for Q waves showed strongest correlation with infarct size as measured by CMR. Patients with a Q-wave MI had larger infarct size and lower LVEF on baseline CMR than patients with non–Q-wave MI.

				criteria					
Crescenzi, 2004	15145001	Cohort study	*N* = 206 patients undergoing cardiac surgery	Patients with and without new Q waves after cardiac surgery	Patients with ECGs post-cardiac surgery and cTn I and CK-MB before and after surgery	Logistic regression analysis with ROC curves for sensitivity and specificity for cardiac events	Primary outcome of post-operative cardiac events	Further divided into four groups according to the presence or absence of new Q waves and peak cardiac marker elevations	15 new Q waves (7.3% of 206 patients): these new Q waves were found in 11 CABG patients out of 136 (8.0%) and in four valvular patients out of 70 (5.7%). Isolated presence of a new Q wave following cardiac surgery was not associated with cardiac events, while peak biochemical marker release coupled with a new Q wave was predictive for post-operative cardiac events.
Iqbal, 2013	23685296	This was a retrospective analysis of prospectively collected data from consecutive	*N* = 5833	Stent thrombosis (ST) cases were divided into definite and probable ST, according	All patients undergoing PCI with stenting. No exclusion criteria.	Univariate predictors were identified using Student's t-test and X^2 test, and entered into a Cox proportional	Aimed to examine the incidence of stent thrombosis and associated risk factors in a ‘real-world, all-comers’ study		For 5833 PCI patients followed up for 2 years, the incidence of definite and probable ST together was 1.9%; of these 73% were early, 11% late, and 16% very

		patients undergoing PCI with stent deployment at the South Yorkshire regional cardiothoracic centre between 2007 and 2010		to the ARC criteria		hazards model to identify factors independently associated with ST			late ST. Cardiogenic shock, STEMI, lack of dual antiplatelet treatment, diabetes mellitus, stent length, and stent diameter were the independent predictors of ST.
Popovic, 2018	29311288	This retrospective observational cohort study included all STEMI patients admitted to a single centre from 2006 to 2015 who underwent early interventional coronary	*N* = 1232		This retrospective observational cohort study included all STEMI patients admitted to the centre from 2006 to 2015 who underwent early interventional coronary procedure.<br>STEMI was defined according to the universal definition by (1) continuous	Kaplan–Meier survival curves were compared using the log-rank test. Crude HR for all-cause mortality was calculated using Cox semiparametric regression models. Proportional hazard assumption was assessed using Schoenfeld residuals. Because	This study assessed the clinical characteristics and long-term outcomes and identified risk factors of coronary embolism (CE) in a large consecutive series of STEMI patients		The aetiology of STEMI secondary to CE is diverse, ranging from cardiac to systemic disease, and patient long-term survival is worse than expected according to the baseline cardiovascular risk

		procedure			chest pain for at least 20 minutes, (2) ST-segment elevation ≥1 mm (0.1 mV) in ≥2 contiguous leads on the 12-lead ECG, and (3) new-onset LBBB and treated within 12 h of pain onset.	of the small sample size and outcome events, multivariable adjustment was considered inappropriate.			
Moussa, 2020	32972528	Retrospective analysis of data collected in the Diagnostic Catheterization and Percutaneous Coronary Intervention (CathPCI) registry of the National Cardiovascular Data Registry	*N* = 5 100 394		All PCI procedures performed between 2009 and 2017. Patients who underwent salvage PCI or those presenting with cardiogenic shock or cardiac arrest were excluded.	To account for the non-random differences between in-stent restenosis (ISR) and non-ISR patients, propensity score matching was used. Variables that were thought to be potential confounders were selected for inclusion into the propensity model,	This study aims to provide an analysis of the temporal trends, clinical presentation, treatment strategies, and in-hospital outcomes of patients undergoing PCI for ISR in the United States		ISR represents approximately 10% of all PCI and is treated most commonly with another stent. Approximately 25% of patients present with acute MI. In-hospital outcomes of patients with ISR PCI are comparable with those undergoing non-ISR PCI.

		(NCDR) between 2009 and 2017				based on clinical subject matter expertise. Multivariable logistic regression model conditioned on the following variables: age, sex, glomerular filtration rate, hypertension, dyslipidaemia, family history of CAD, previous MI, previous HF, previous valve surgery, previous CABG, CAD presentation, angina classLV systolic dysfunction, number of diseased vessels, saphenous vein graft (SVG) involvement, bifurcation lesion, chronic total occlusion (CTO),			

						teaching hospital, average hospital PCI volume, urban/rural hospital.			
Wang, 2021	34430594	Retrospective cohort of patients from the database of 'Acute Myocardial Infarction in Tianjin' (TAMI), from 1 January 2018 to 31 August 2018	Enrolled 1032 cases of type 1 MI and 42 cases of type 4c MI	Type 1 MI and type 4C MI	Patients meeting the diagnostic criteria for type 1 or 4c MI, as per the Fourth UDMI and age >18 years. The exclusion criteria were as follows: (1) patients who did not undergo coronary angiography; (2) unwillingness to cooperate with the treatment regime; and (3) pregnant or lactating women.	The incidence of MACE was calculated, and Kaplan–Meier survival curves were drawn to analyse the prognosis of patients with type 4c MI. The risk ratios (RR) for the incidences of clinical events were calculated using the generalized linear model.<br>The Cox proportional hazard regression model was employed to analyse the relationship	This study assessed the prognosis and treatment of type 4c MI in patients with NSTEMI.<br>All cases were followed up for 1 year.<br>The outcome was MACE (including all-cause deaths, non-fatal MI, HF necessitating hospitalization, uncontrollable angina pectoris, and revascularization of the target vessels).		Type 4c MI has a poorer prognosis than type 1 MI. DCB angioplasty and DES implantation show similar efficacy in the treatment of type 4C MI.

						between the vascular interventional therapy strategy (drug-coated balloon [DCB] or drug-eluting stent [DES]) and MACE events in the type 4c MI cohort. ROC curve analyses were conducted to test the predictive ability of prognostic factors, with their predictive power indicated by the AUC.			
Saw, 2014	24726091	Retrospective review of all coronary angiograms of women aged ≤50 at Vancouver General	*N* = 7605		Women aged ≤50 who underwent coronary angiography for ACS or stable symptoms	Baseline continuous variables were expressed as mean ± SD, and baseline discrete variables were expressed as mean and percentages	This study assessed the prevalence of non-atherosclerotic coronary artery disease (NACAD) in young women		NACAD was not rare among young women undergoing coronary angiography and was an important cause of MI, accounting for 30%. Spontaneous coronary artery dissection (SCAD) was the most

		Hospital from 1 December 2009 to 30 November 2011							commonly encountered NACAD in young women, causing 24% of MIs.
Kerensky, 2002	11985907	Coronary angiograms from 350 patients randomized to the early invasive strategy in the Veterans Affairs Non–Q-Wave Infarction Strategies in-Hospital (VANQWISH) trial were systematicall y analysed in an angiographic core laboratory	*N* = 350		Clinical presentation of evolving acute MI, CK-MB isoenzyme >1.5 times ULN and no new Q waves on serial ECGs.<br>Reasons for exclusion from the trial were cardiogenic shock, severe HF, persistent or recurrent ischaemia at rest, or ventricular tachyarrhythmias.	Pearson's X^2test also was employed to compare the proportion of patients with angiographically normal coronaries, three-vessel CAD, severe (>70%) proximal stenosis, and total (100%) stenosis between those who had no and ≥1 culprit lesion. Patients with no culprit lesion, a single incomplete occlusion, a single abrupt occlusion or multiple culprit lesions were	This study assessed the underlying coronary anatomy and characterized the culprit lesion after non–Q-wave myocardial infarction (NQWMI)		Coronary angiography early after NQWMI frequently identifies severe obstructive CAD, but a single identifiable culprit lesion was identified in <50% of patients. Multiple culprit lesions were seen in 14% of patients. An angiographic culprit lesion could not be identified in more than one-third of patients undergoing coronary angiography as part of an invasive strategy.

						compared with respect to treatment with revascularization (coronary angioplasty or CABG). These four groups of patients were analysed using Pearson's test.			
Gehrie, 2009	19781432	NSTEMI patients enrolled in CRUSADE from 2001 to 2005	*N* = 55 514		Patients included in CRUSADE had ischaemic symptoms for >10 minutes within the previous 24 h and at least one of the following: CK-MB or cTn I or T above the ULN or dynamic ST-segment ECG changes including ST-segment depression ≥0.5 mm or transient	For the descriptive analysis, patient baseline demographics, clinical characteristics, care patterns, and in-hospital outcomes were compared between women and men. Medians and 25th and 75th percentiles were reported for continuous	This study assessed the sex-specific characteristics and outcomes of patients without obstructive CAD.<br>Using data from NSTEMI patients enrolled in CRUSADE from 2001 to 2005 differences in clinical features and in-hospital outcomes between men and women with no obstructive CAD were evaluated.		Among NSTE ACS patients undergoing coronary angiography, absence of obstructive CAD is more common in women than men. Although non-obstructive CAD was twice as common among women with NSTEMI, sex differences in characteristics and outcomes were similar to those found with obstructive CAD.

					ST-segment elevation of >0.5 mm (lasting <10 minutes)	variables, and percentages were reported for categorical variables.<br>Continuous and ordinal categorical variables were compared using stratum-adjusted Wilcoxon rank sum tests, whereas nominal categorical variables were compared using stratum adjusted χ2 tests (in which stratification was by hospital).			Unadjusted in-hospital outcomes of NSTEMI patients with non-obstructive CAD are favourable in both sexes. Whether the underlying pathophysiology of NSTE ACS without documentation of obstructive CAD is different between women and men requires further study.
Andersson, 2018	29029035	ST-elevation ACS (STE-ACS) and triaged for acute coronary angiography (CAG)	*N* = 4793		STE-ACS if they presented with symptoms suggestive of MI together with significant ST-elevation in their ECG, as defined in	The association between the severity of CAD and the hazard of death during follow-up was assessed by univariate and multivariate Cox	This study assessed survival and causes of death in patients with STE-ACS with and without obstructive CAD.		STE-ACS patients without obstructive CAD had a long-term risk of death similar to or higher than patients with obstructive CAD. Causes of death were less often

		between November 2009 and December 2014 at the Heart Centre, Rigshospitalet, Copenhagen University Hospital. The patients were identified from the Eastern Danish Heart Registry, a clinical registry where all CAG and PCI procedures performed in eastern Denmark are prospectively registered.			current guidelines.<br><br>Only patients admitted within 12 h from symptom onset included. Patients in cardiogenic shock on presentation as well as patients resuscitated after cardiac arrest were included if STEMI was the suspected cause. If a patient was admitted more than once during the study period, only the first admission was included.<br><br>Exclusion criteria included patients who died before the CAG could be performed and patients where it was not technically	regression analysis			cardiovascular. This suggests that STE-ACS patients without obstructive CAD warrant medical attention and close follow-up.

					possible to perform CAG and foreign residents.				
Larsen, 2013	23261001	STEMI patients recruited from the HORIZONS-AMI study (a prospective, open-label, multicentre, RCT of patients with STEMI undergoing primary PCI	*N* = 3602		Aged >18 years who presented within 12 h of symptom onset with ST-segment elevation of >1 mm in >2 contiguous leads, new LBBB, or true posterior MI on the ECG were considered for enrolment	Comparison between the early (30-day) and late (3-year) rates of death, reinfarction, major bleeding not related to CABG, and revascularization of any vessel for ischaemia subsequent to the index procedure in patients with CAD vs. no CAD	This study assessed the outcomes of patients with STEMI in the HORIZONS-AMI trial according to the presence or absence of significant CAD		3.5% of patients with STEMI had no significant CAD. The 3-year prognosis for these patients was favourable compared with that of patients with STEMI and obstructive CAD
Reynolds, 2021	33191769	Prospective, multicentre, international, observational study	*N* = 301		Prospective, multicentre, international, observational study of women with a clinical diagnosis of MI. If ICA revealed <50% stenosis in all major arteries,	Evaluated differences in study variables between patients with and without OCT culprit lesions and with or without CMR abnormalities using the χ2 or Fisher	This study assessed coronary OCT and CMR imaging to assess mechanisms of MINOCA		Multimodality imaging with coronary OCT and CMR identified potential mechanisms in 84.5% of women with a diagnosis of MINOCA, 75.5% of which were ischaemic and 24.5% of which were non-

					multivessel optical coherence tomography (OCT) was performed, followed by CMR (cine imaging, LGE, and T2-weighted imaging and T1 mapping).	exact test for categorical variables and Mann–Whitney *U* test for continuous variables. The likelihood of establishing a diagnosis of an ischaemic or non-ischaemic cause of MINOCA by OCT and CMR was compared with the likelihood of making such a diagnosis by OCT alone and by CMR alone using the McNemar test. Multivariable logistic regression modelling was performed to assess the odds of an OCT-defined culprit lesion, adjusting for demographics,			ischaemic, alternate diagnoses to MI. Identification of the cause of MINOCA is feasible and has the potential to guide medical therapy for secondary prevention.

						clinical characteristics, and laboratory values.			
Abdel-Aty, 2004	15123531	Cohort	*N* = 73; first group15 reperfused MI patients studied at two time points, one at Day 1 and the other at 3 months after MI; and second group 58 patients who underwent only one cardiac MRI scan at acute (<2 weeks of MI) or chronic (>4 weeks of MI) phase	MI diagnosis was based on ECG changes and >2-fold elevation of creatine kinase and/or positive troponin T	Exclusion criteria: clinically unstable, severe arrhythmia, contraindication to cardiac MRI	In the first group, quantitative segmental and region of interest-based analyses were performed to observe signal changes between the acute and chronic phases. In the second group, T2-weighted and LGE images were examined visually for corresponding segments for hyperintensity	Image contrast between remote and infarcted segments dropped significantly between Day 1 and 3 months	The methodology did not segregate MI patients based on index tissue injury, hence the differences in delayed infarct healing were not investigated	Acquiring native T2-based cardiac MRI and LGE cardiac MRI can differentiate acute from chronic lesions with a specificity of 96%
Francone, 2009	19942086	STEMI cohort	*N* = 70	Four study groups differing based	Inclusion: first STEMI undergoing primary PCI <12 h	Image analysis of MI size based on mean + 5 SD;	Shorter time to reperfusion was associated with smaller	Time of study: October 2007 to May 2008. Salvaged area	In STEMI patients treated with PCI, time to reperfusion

				on time to PCI: ≤90 min to PCI (*n* = 19); 90–150 min to PCI (*n* = 17); 150–360 min to PCI (*n* = 17); and >360 min to PCI (*n* = 17)	after onset of symptoms<br>Exclusion: unsuccessful primary PCI, rescue PCI, facilitated PCI, contraindication to GP IIb/IIIa, non-STEMI, previous MI, previous CABG, and contraindication to cardiac MRI.	microvascular obstruction defined as dark zone within infarcted segments on LGE MRI	infarct size and microvascular obstruction and larger salvaged myocardium. Mean MI size increased with increasing time to PCI. Microvascular obstruction was larger in patients receiving late reperfusion. Salvaged myocardium decreased when time to PCI was >90 min.	was computed as difference between T2-weighted cardiac MRI and MI size (measured from LGE MRI).	determines the extent of reversible and irreversible myocardial injury assessed by cardiac MRI
Wu, 1998	9498540	STEMI Cohort	*N* = 44	Two groups, those with microvascular obstruction and those without microvascular obstruction		Covariate adjustment	Risk of adverse events increased with MI size but even when MI size was controlled for, presence of microvascular obstruction remained a prognostic marker of post-infarction complications	In this early study, the type of microvascular injury was not discriminated. That is it was not clear which patients had only microvascular obstruction and which patients had microvascular obstruction with intramyocardial	Microvascular obstruction predicts more frequent complications. Microvascular obstruction status is a strong prognostic marker independent of MI size.

								haemorrhage	
Lechner, 2024	38777509	STEMI cohort, multicentre study	*N* = 1109	Those without microvascular injury and those with microvascular injury. In those with microvascular injury (MVO-/IMH-) patients were divided into those with microvascular obstruction but no intramyocardial haemorrhage (MVO+/IMH-) and those with MVO and IMH (MVO+/IMH+)	Inclusion criteria: acute STEMI and treated with PCI. Exclusion criteria: contraindications for cardiac MRI, previous MI, haemodynamic instability	Cox proportional hazard regressions were used to determine the prognostic significance of microvascular injury and type	MACE-free survival and survival probability	None	Microvascular injury is associated with MACE only in haemorrhagic MI patients. Patients with only MVO (i.e. no IMH) had prognosis similar to those without microvascular injury.
Boeddinghaus, 2025	39824208	Patients with type 2 MI	*N* = 100	Patients identified for type 2 MI underwent invasive	Inclusion: evidence of rise and/or fall in cTn with at least one value above sex-specific 99th	Kaplan–Meier analysis and group-wise comparison using Fisher's exact test	Proportion of patients with type 2 MI reclassified as having spontaneous, secondary, or no MI by	Secondary analysis of a prospective observation cohort study	New clinical classification of MI informed by cardiac imaging would reduce the diagnosis of MI in

				angiography or CT, cardiac MRI, and TTE for potential reclassification	percentile, and symptoms on 12-lead ECG with objective evidence of supply/demand imbalance. Exclusion: patients with type 1 MI, women pregnant or breastfeeding, renal or hepatic impairment, and those with advanced frailty and inability to self-transfer.		the new clinical classification. Prognostic outcome was all-cause death, recurrent MI, or HF hospitalization.		acute illness and identify those patients at highest risk who are most likely to benefit from treatment
Bularga, 2022	35436138	Prospective cohort	*N* = 8064 screened for type 2 MI; adjudication based on imaging (coronary angiograms, cardiac MRI) for reclassificatio	Initial stratification based on cTn	Inclusion: type 2 MI based on cTn.<br>Exclusion: frailty, renal/hepatic failure.	Group comparisons were performed with Fisher's exact, X^2, Kruskal–Wallis, or 1-way ANOVA tests	Primary: prevalence of obstructive or non-obstructive CAD; secondary: LV dysfunction and pattern of myocardial injury	None	Coronary and cardiac imaging in patients with type 2 MI identified CAD in 2/3 and LV dysfunction in 1/3 of patients. Unrecognized or untreated coronary or cardiac disease is seen in most patients with type 2 MI.

			n						
Selvanayagam, 2005	15723982	Cohort	*N* = 59		Inclusion: one of two-vessel PCI, planned insertion of >30 mm of stent to a single vessel, or planned treatment of a segment that involved at least one side branch of 2.0 mm size.<br>Exclusion: lack of informed consent, contraindication to GP IIb/IIIa, contraindication to cardiac MRI.	Paired t-test and unpaired-sample t-test for comparisons of means within the study group; and binary logistic regression to determine which clinical and angiographic parameters predicted the likelihood of myocardial hyperenhancement	Global LV function, LVEF, areas of hyperenhancement based on LGE cardiac MRI. Identification of new hyperenhancement adjacent to stent injury and new hyperenhancement distal to the stent deemed as downstream injury.	None	Post-procedural elevation in troponin I is correlated with new irreversible myocardial injury on LGE MRI
Kofoed, 2021	33632478	Observational study	*N* = 978	Patients with NSTEACS undergoing CTCA prior to invasive angiography	Age ≥18 years, clinical suspicion of NSTEACS, and ≥1 of the following high-risk criteria: 1) ECG changes indicating new ischaemia (new ST-segment	Intention-to-diagnose principle	The primary endpoint was a composite of all-cause death, non-fatal recurrent MI, hospital admission for refractory myocardial ischaemia, or HF	Observational component of the VERDICT (Very Early Versus Deferred Invasive Evaluation Using Computerized Tomography in Patients With Acute	Coronary CTA is equivalent to ICA for the assessment of long-term risk in patients with NSTEACS

					depression, horizontal or downsloping ≥0.05 mV in two consecutive leads, and/or T-wave inversion >0.01 mV in two leads with prominent R wave or R/S ratio >1); and 2) an increase in circulating troponin levels. Exclusion criteria were pregnancy; patient inability to understand the trial information; an indication for acute ICA; expected survival <1 year; or known intolerance to platelet inhibitors, heparin, or X-ray contrast, which could not be remedied medically. For			Coronary Syndromes) trial	

					coronary CTA, patients with previous CABG, plasma creatinine >140 µmol/L, known AF, or women <45 years of age were not considered eligible.				
Meah, 2022	36339063	Observational	*N* = 422		In appendix, not accessible	Logistic regression analyses were performed to identify predictors of type 1 MI	Plaque features in CTA in type 1 and type 2 MI	Secondary analysis of two prospective studies (RAPID CTA and DEMAND-MI)	Higher low-attenuation coronary plaque burden in patients with type 1 MI may help distinguish these patients from those with type 2 MI
Serruys, 2024	38583086	Multicentre, prospective, proof-of-concept trial	*N* = 114	Patients with CCS or ACS	Equivalent angina, silent ischaemia, and chronic or stabilized ACS (Braunwald's classification) who had been referred by the ICA-Heart Team for surgical revascularization	Concordance and agreement between treatment strategies and executions; Cohen's kappa was used	The primary feasibility endpoint was the percentage of operations performed without access to the ICA. The primary safety endpoint was graft patency on 30-day follow-up CCTA. Secondary endpoints included topographical adequacy of grafting,	Bias: pre-knowledge that these patients had three-vessel disease with/without left main CAD, were amenable to surgical revascularization.<br>Due to the first-in-human nature of this trial, it included selective patients	CABG guided by CCTA is feasible and has an acceptable safety profile in a selected population of complex CAD

							MACCE, and major bleeding events at 30 days.	who mainly had CCS and were of low surgical risk.	
Dai, 2023	36808235	Single-centre observational retrospective	*N* = 359	Patients with CCS	Consecutive patients with normal baseline (pre-procedural) hs-cTn T levels who were referred for elective PCI and underwent coronary CTA within 90 days before the procedure	Multivariable logistic regression analysis, sensitivity, specificity, PPV and NPV. Multivariable Cox proportional hazard regression was used to calculate adjusted HR.	Coronary plaque characteristics and physiologic disease patterns (focal vs. diffuse) assessed by CTA in predicting PMI and adverse events	Prospective studies with larger sample size are needed to confirm findings. Second, only relatively low-risk patients in stable conditions and with normal pre-PCI hs-cTn T values were considered.	Coronary CTA can evaluate plaque characteristics and physiologic disease patterns simultaneously, which plays an important role for risk stratification before PCI
Palmisano, 2022	34874200	Single-centre, observational	*N* = 84	Consecutive patients (age, >18 years) presenting at the ED for acute chest pain or anginal equivalents who were candidates for triple rule-out CT	Inclusion criteria were (a) mild hs cTn rise (>14 ng/L) at single sample or mild hs-cTn rise stable at two samples, and (b) absence of diagnostic criteria for AMI at multimodality evaluation (clinical	The Cohen coefficient (k) was calculated for the assessment of segmental agreement between late contrast enhancement (LCE) CT and LGE MRI and the determination of	Rate of diagnosis identified by CTA		A CT protocol including triple rule-out and late contrast enhancement CT scans improved diagnostic rate in participants presenting with acute chest pain syndrome

				examination were screened for enrolment	evaluation, cardiac biologic markers, electrocardiography, and echocardiography)	interobserver agreement in scar detection			
Liang, 2023	37526288	Large single-centre cohort study	888 patients	MINOCA endotypes	All patients included had undergone diagnostic ICA, followed by CMR at a tertiary cardiology centre. Those with obstructive coronary disease (≥50% stenosis) on angiography, and those with no documented troponin result or a negative troponin on review on clinical notes, were excluded.		Identify the proportion of patients with suspected MINOCA or non-MINOCA that CMR reclassifies with an alternative diagnosis	The Bristol CMR registry is derived from patients who have undergone CMR for investigation with myocardial injuries and non-obstructed coronary arteries over a period of 10 years.<br>The time period also covered the COVID-19 pandemic and thus may not have recruited all potential patients, therefore potentially underestimating the prevalence of each diagnosis.	In a large single-centre cohort of patients presenting with ACS and unobstructed coronary arteries, CMR was able to reclassify diagnosis in nearly 80% of patients. In addition, the majority of patients with suspected ACS with unobstructed coronary arteries have an alternative non-MINOCA diagnosis (73%) (myocarditis, Takotsubo, cardiomyopathies, or normal CMR/non-specific findings), whilst only a minority had confirmed MINOCA (27%).

Joshi, 2014	24224999	Prospective cohort	*N* = 80; 40 with STEMI or NSTEMI and 40 with stable CAD	MI based on Third UDMI; stable CAD based on ICA	Inclusion: STEMI or NSTEMI, or stable angina pectoris undergoing elective ICA.Exclusion: ≤50 years, type 2 diabetes, women of childbearing age not using contraception, several renal failure, allergic to contrast agents, and inability to provide consent.	Parametric and non-parametric tests depending on normality of data. Fisher's or X^2 test for analysis of categorial variables.	Primary: comparison of F18-fluoride tissue-to-background ratio of culprit and non-culprit plaques of patients with acute MI.<br><br>Secondary: comparative imaging and histological characterization of F18-fluoride positive and negative atherosclerotic plaques in patients with coronary and carotid artery disease.	None	F18-NaF and PET-CT can identify and localize ruptured and high-risk coronary plaque. Further studies are needed to establish the method.
Tzolos, 2023	36526577	Single-centre case-control study	*N* = 99; 49 with spontaneous AMI and 50 with AMI	Fourth UDMI and cTn >50x the URL within 7 days of presentation to hospital. Patients with stable CAD had no angina or recent MI.	Inclusion: >40 years of age.<br><br>Exclusion: inability to provide informed consent, pregnancy or breastfeeding, contraindications to iodinated contrast, use of anticoagulant	Multivariable linear regression analysis		Coronary artery F18-GP1 uptake	Coronary F18-GP1 PET/CT angiography was able to identify *in vivo* coronary thrombosis in patients with AMI. This approach may help define the role of thrombosis within the heart.

					therapies, GFR <30 mL/min/1.73 m^2, metastatic malignancy, inability to tolerate supine position.				
Çitaku, 2021	34511594	STEMI registry-based cohort	*N* = 80		Inclusion: patients treated at the Clinica of Cardiac Surgery and Invasive Cardiology<br>Exclusion: patients with missing records or missing data	Correlation analysis; multiple linear regression analysis to assess potential predictor for MI size	Correlation between the size of MI and time of initiation of primary PCI	2019–2021	A significant correlation between size of MI and time of initiation of PCI was found based on perfusion myocardial scintigraphy
**OTHER ARTICLES**									
**Study first author surname and year**	**PMID reference for study**	**Type of article** (editorial, commentary, systematic review, narrative review,	**Population**	**Topics**	**Key point (1)** (evidence level if clinical practice guideline)	**Key point (2)** (evidence level if clinical practice guideline)	**Key point (3)** (evidence level if clinical practice guideline)	**Other considerations** (bias, uncertainty, future research requirements, etc.)	**Interpretation**

		clinical practice guideline or consensus statement)							
McLaren, 2024	39435181	Narrative review	Acute Coronary Occlusion Myocardial Infarction (ACOMI)	ACOMI in ST-elevation versus non-ST-elevation MI	25% of 'non-STEMI' have acute coronary occlusion, leading to delayed reperfusion and higher mortality	A paradigm shift to ACOMI, instead of STEMI, may be needed	Adjunctive use of AI for ECG interpretation and bedside cardiac imaging may offer better risk stratification for who needs urgent reperfusion	Uncertainty still exists in the need for urgent reperfusion for ACOMI instead of STEMI, as trials of reperfusion used STEMI criteria	While a shift in focus to ACOMI from STEMI is pragmatic, further research is required to determine a benefit of urgent reperfusion
Ricci, 2025	39818676	Narrative review	ECG patterns of occlusion myocardial infarction (OMI)	Current limitations of the STEMI vs. NSTEMI framework: need for emerging paradigm distinguishing OMI from non-occlusion myocardial infarction (NOMI)	25% to 34% of NSTEMI cases may also include acute coronary occlusion	Distinguishing OMI from NOMI	Reperfusion therapy for high-risk ECG patterns not covered by current STEMI criteria	Further validation is required particularly of benefit of reperfusion for OMI	Implications for clinical practice

Chapman, 2025	39658094	Narrative review	Type 2 MI, challenges in diagnosis	Type 2 MI encompasses a range of underlying causes	No clinical trials of treatment for type 2 MI currently performed	The adoption of type 2 MI in clinical practice has been hampered by the heterogeneity of causes for type 2 MI	Lack of objective diagnostic criteria for type 2 MI have restricted adoption of its use in clinical practice	Patients with type 2 MI have poor prognosis	Randomized trials of investigation and treatment for patients with type 2 MI are needed
Gaudino, 2024	39279125	Review	A debate to suggest that MI after cardiac surgery should be redefined	Issues for definition of MI after cardiac surgery	Issues include arbitrary cardiac biomarker thresholds, which occur in large proportions of post cardiac surgery patients and new post-operative ECG changes seen in a large proportion of patients	Biomarkers do associate with prognosis	Propose a new definition based on cTn levels versus Fourth UDMI	Using outcome-based criteria may still miss clinically significant adverse events	Advocate to redefine the biomarker threshold to at least 130–170× URL in the first 24 h, even in the absence of electrocardiographic evidence of ischaemia, as the most prognostically relevant criterion for the diagnosis of MI after cardiac surgery
Raber, 2019	31112213	Expert consensus document of the European Association of Percutaneous		Use of intracoronary imaging including intravascular ultrasound (IVUS), OCT,	Beyond guidance of stent selection and optimization of deployment, invasive imaging facilitates angiographic	Intravascular imaging can provide additional important diagnostic information when confronted with	This document focuses on useful imaging features to identify culprit and vulnerable coronary plaque, which offers the interventional cardiologist guidance		Continued advancements in the technology, with higher resolution, faster image acquisition times, combined OCT/IVUS catheters, and more

		Cardiovascular Interventions		and near-infrared spectroscopy (NIRS)-IVUS in ACS patients	interpretation and may guide treatment in ACS	angiographically ambiguous lesions and allows assessment of plaque morphology enabling identification of vulnerability characteristics	on when to adopt an intracoronary imaging-guided approach to the treatment of CAD and provides an appraisal of intravascular imaging-derived metrics to define the haemodynamic significance of coronary lesions		sophisticated co-registered image analysis, will facilitate greater adoption through ease of use and interpretation. Intracoronary imaging-guided PCI has an exciting future.
Ibanez, 2019	31296297	JACC Expert Panel – State of the Art	Spontaneous MI	Cardiac MRI based detection of tissue injury (oedema, inflammation, microvascular obstruction, haemorrhage, cardiomyocyte necrosis, and fibrosis) associated with MI	Discussion of cardiac MRI methods for detection and sizing of myocardial infarction, oedema, microvascular obstruction, and haemorrhage	Standardization of cardiac MRI methods to improve clinical translation		Recommendation for cardiac MRI endpoint selection for clinical trials based on pathophysiology and its association with hard outcomes	Number of cardiac methods have been proposed for characterizing tissue in reperfused MI. This JACC Expert Panel provides insight into which methods are sensitive and specific to detect key clinical endpoints in experimental and clinical trials.
Vora, 2024	38613553	State-of-the-Art review,	Spontaneous MI	Summary of pathophysiolo	Appropriate use of non-invasive	Summarizes past and ongoing		Identifies potential future directions of	Outlines the role of imaging in identification

		JACC Imaging		gy of microvascular injury in reperfused MI	imaging modalities for invasive and non-invasive detection of microvascular injuries	interventional trials aiming to mitigate adverse consequences of microvascular injury based on imaging		investigations to enable improved detection of the progression of tissue-specific changes in the post-MI period	of microvascular injury and how it can be used to develop novel therapies to improve patient management in the post-MI period
Kolossvary, 2017	29255692	Narrative review			Comprehensive coronary plaque assessment holds potential to significantly improve individual risk assessment incorporating adverse plaque characteristics and the extent and severity of atherosclerotic plaque burden	Coronary CTA became a promising modality for the evaluation of functional significance of coronary lesions using FFR-CT and/or rest/dynamic myocardial CT perfusion			The evaluation of lesion-specific ischaemia using combined functional and morphological plaque information could ultimately improve the diagnostic performance of CTA and thus patient care
Pergola, 2023	37082977	Narrative review	Patients with SCAD	n/a	n/a	n/a	n/a	n/a	llustrates the current and potential role of CCTA in the diagnosis of SCAD, highlighting advantages and disadvantages of this

									imaging modality compared with ICA.<br>CCTA may be helpful to screen and evaluate extracoronary arteriopathies closely related to SCAD.
Tzolos, 2021	34364527	Case report	*N* = 1, spontaneous MI (STEMI)	ICA and F18-GP1 PET/CT			F18-GP1 PET/CT uptake in coronary thrombus		Coronary thrombus imaging with F18-GP1 PET/CT may be useful in discriminating between demand ischaemia and spontaneous acute MI
Wells, 2016	27072005	Systematic review	Broader instrumentation-related specifications	Limits of detection in nuclear medicine	Nuclear medicine methods, single photon-emission CT (SPECT) and PET, provide excellent sensitivity but have poor spatial resolutions compared with other methods such as MRI and PET				For accurate visualization of molecular processes nuclear medicine methods are typically combined with MRI or CT, which have significantly higher spatial resolution

Stehli, 2019	31092091	Cohort study of patients with STEMI and NSTEMI from 30 hospitals in Australia	*N* = 13 451	Stratification by sex and type of MI (NSTEMI and STEMI)	All patients were treated with PCI	Adjusted log-transformed symptom-to-door and door-to-balloon times analysed using linear regression. Logistic regression analysis to determine predictors of mortality.	Time to presentation and treatment. All-cause 30-day mortality.	Observational study with risk of residual confounding	The adjusted geometric mean symptom-to-door and door-to-balloon times were 28.8 and 7.7 minutes longer, respectively, in females than males with STEMI. Female sex independently predicted higher 30-day mortality (OR 1.67; 95% CI 1.11–2.49) in STEMI.

3

4. Evidence tables for section 16

Section	Section 16 (Special Considerations)								
**META-ANALYSES**									
**Study first author surname, year, and acronym**	**PMID reference for study**	**Population**	**Number of participants or studies**	**Intervention**	**Key inclusion & exclusion criteria**	**Type of analysis** (random-effect, fixed-effect, Bayesian approach)	**Relevant outcome(s) and PROMS**	**Other methodological aspects**	**Key findings and interpretation**
Genereux, 2012	22503058	Studies of patients undergoing transcatheter aortic valve replacement (TAVR)	Studies: 16<br>Patients: 3519	TAVR	Inclusion criteria: patient undergoing TAVR reporting ≥1 Valve Academic Research Consortium (VARC) outcome	Random-effects model to report summary rate estimates with 95% CI	30-day and 1-year VARC outcomes		Overall device success:92.1% (95% CI 88.7%–95.5%).<br>All-cause 30-day mortality: 7.8% (95% CI 5.5%–11.1%).<br>Myocardial infarction: 1.1% (95% CI 0.2%–2.0%).<br>Acute kidney injury:20.4% (95% CI 16.2%–25.8%).<br>Life-threatening bleeding:15.6% (95%

									CI 11.7%–20.7%).<br><br>Vascular complications: 11.9% (95% CI 8.6%–16.4%).<br><br>All neurologic events: 5.7% (95% CI 3.7%–8.9%).<br><br>Permanent pacemaker: 13.9% (95% CI 10.6%–18.9%).<br><br>1-year all-cause mortality: 22.1% (95% CI 17.9%–26.9%).<br><br>Cardiovascular mortality: 14.4% (95% CI 10.6%– to 19.5%).
Aimo, 2018	29335288	Studies of patients with chronic HF	10 studies (11 cohorts), 9289 patients		Inclusion criteria:<br>• English language<br>• Age ≥18 years<br>• Diagnosed with HF<br>• Enrolment of outpatients or	Random-effects model and the generic inverse variance method reporting HR and 95% CIs	All-cause death, cardiovascular death, cardiovascular hospitalization	Data for cardiovascular death available for 6 cohorts and data for cardiovascular hospitalization available for 6 cohorts	hs-cTn T independently associated with all-cause death, cardiovascular death, and cardiovascular hospitalization;<br>(all *P* values < .001).<br><br>hs-cTn I independently

					patients undergoing elective admission<br>• Reported use of a hs assay for cTn T and cTn I<br>• Prognostic information available<br>• Authors' availability to provide individual patient data				associated with all-cause death (*P* = .005).<br>hs-cTn T concentration of 18 ng/L identified as the optimal threshold for the prediction of all-cause death.
Smilowitz, 2019	30985328	Studies of patients undergoing non-cardiac surgery	169 studies, 530 867 patients	Non-cardiac surgery	Key inclusion criteria: studies reporting incidence, management, and outcomes with myocardial injury after non-cardiac surgery. Key exclusion criteria: small studies (*n* <50), no clearly	Random-effects models reporting relative risks and 95% CIs	In-hospital and 1-year mortality.		Patients with vs. without myocardial injury had higher mortality in hospital both in hospital (relative risk 8.3, 95% CI 4.2–16.6, *P* < .001) and at 1 year (relative risk 4.1, 95% CI 3.0–5.6, *P* < .001).

					defined biomarker thresholds.				
Murata, 2022	34861515	Critically ill patients	1898	Intrahospital transport	Adults aged 16+. The investigation of safety and adverse events during intrahospital transport of critically ill patients, defined as patients admitted to an intensive care unit (ICU).	Quantitative analysis using a random-effects model was performed to pool measured effects	The results could assist in risk-benefit analysis of diagnostic or therapeutic procedures requiring intrahospital transport of critically ill patients	Because of the high risk of bias, the internal validity of the results was low.<br>Judgements about life-threatening adverse events could be subjective and may have low reproducibility because many studies included in the meta-analysis did not define life-threatening adverse events and the studies assessed the frequency of life-threatening events based on discussion between authors.	Adverse events can occur during intrahospital transport of critically ill patients, but the frequency of critical adverse events is relatively low
**RANDOMIZED CONTROLLED TRIALS**									
**Study first author**	**PMID reference for**	**Population**	**Number of**	**Intervention**	**Key inclusion &**	**Type of analysis** (ITT,	**Relevant outcome(s)**	**Other methodological aspects**	**Key findings and**

surname, year, and acronym	study		participants	and control	exclusion criteria	PP, ITT or PP with adjustment for pre-specified covariates)	and PROMS	(allocation concealment, single/double blinding, sham procedure in device trials, missing data on outcomes, outcome central adjudication…)	interpretation
**OBSERVATIONAL COHORT STUDIES**									
**Study first author surname, year, and acronym**	**PMID reference for study**	**Population and study type** (case-control, cohort, etc.)	**Number of participants**	**Stratification**	**Key inclusion & exclusion criteria**	**Type of analysis** (covariate adjustment; propensity score matching, weighting, adjustment; trial emulation; quasi-experimental design…)	**Relevant outcomes and PROMs**	**Other methodological aspects** Bias in design (i.e. immortal-time bias, survivorship bias, etc.), risk of residual confounding (i.e. e-value, falsification endpoint), other issues as evaluated in specific tools (i.e. ROBIN I), generazibility.	**Key findings and interpretation**
Roosen, 2000	10852416	Observational retrospective	*N* = 100	Clinical diagnosis vs. post-mortem	• Medical ICU (MICU) population	Univariate comparison			MI recognized as one of the most frequent missed major

		comparative		diagnosis	• Death<br>• Autopsy performed				diagnoses
Gibson, 2003	12849652	Retrospective observational: patients enrolled in the TIMI 4, 9, and 10B and Intravenous NPA for the Treatment of Infarcting Myocardium Early (InTIME-II) acute MI trials	20 101<br>Recurrent MI (*n* = 836)<br>No recurrent MI (*n* = 19 265)	Recurrent MI after fibrinolytic administration during the index hospitalization	STEMI	Adjustment for variables previously identified as associated with mortality and recurrent MI (e.g. age, sex, anterior MI, pulse rate on admission, history of hypertension, previous angina, current smoker, previous MI, weight, time from symptom onset to treatment)	Long-term mortality up to 2 years	Multiple definitions were used for recurrent MI. Data were unavailable as to which definition was used for each specific case of recurrent MI.	Early recurrent MI is associated with increased mortality up to 2 years. However, most deaths occur early, and the risk of additional deaths between the index hospital period and 2 years was not significantly increased among patients with recurrent MI. PCI during the index hospitalization was associated with a lower risk of recurrent MI and 2-year mortality.

Kernis, 2003	14522475	STEMI patients who underwent primary PCI in the Primary Angioplasty in Acute Myocardial Infarction (PAMI) studies	3646: early reinfarction, *n* = 77; without early reinfarction, *n* = 3569	30-day reinfarction	STEMI treated with primary PCI.<br>The trials excluded patients presenting with cardiogenic shock or high bleeding risk.	Multivariate logistic regression to determine independent correlates of reinfarction-MI. Clinical and angiographic variables that showed a univariate relation with reinfarction were included in the multivariate analysis.	Impact of 30-day reinfarction on 6-month clinical outcome	Retrospective analysis design.<br>Results may be less representative of contemporary practice, due to lower rates of GP IIb/IIIa and thienopyridine medications, and lower stent use.	Early reinfarction after an AMI treated with primary PCI is relatively uncommon (2.1%) and strongly associated with increased mortality and revascularization at 6 months.<br>Optimal revascularization during primary PCI may decrease reinfarction-MI rates.
Perkins, 2003	14624686	Retrospective observational cohort study	49	Clinical diagnosis vs. post-mortem diagnosis	• ICU (medical and surgical) population<br>• Death<br>• Autopsy performed	Univariate comparison	Significant discrepancies between clinical diagnoses before death and post-mortem findings,<br>Low incidence of post-mortem examination.	A small study with a low post-mortem examination rate.<br>Selection bias of patients for post-mortem examination at the discretion of the intensive care	MI the most frequent missed major diagnosis

								physician.	
Cardinale, 2004	15148277	Observational, single-centre cohort study	703	Patients stratified based on cTn I levels after high-dose chemotherapy.<br><br>In 495 patients, both early and late cTn I values were <0.08 ng/mL (cTn I −/− group); in 145, there was only an early increase (cTn I +/− group); and in 63 patients, both values increased (cTn I +/+ group).<br><br>Patients	Inclusion criteria: consecutive cancer patients undergoing high-dose chemotherapy.<br><br>Exclusion criteria: presence of ischaemic or valvular heart disease, LVEF <50%, age ≥70 years, abnormal renal or hepatic function, hypertensive patients treated with β-blocking agents and angiotensin-converting enzyme inhibitors.	Comparison between early-Tn I and late-Tn I values were made with the ANOVA method. Kaplan–Meier analysis was used to compare the time-to-event rate among the three groups.	The primary endpoint was the occurrence during follow-up of one of the following cardiac events: (1) death resulting from a cardiac cause; (2) acute pulmonary oedema; (3) overt HF; (4) asymptomatic LVEF reduction (≥25% from baseline); or (5) life-threatening tachyarrhythmias and conduction disturbances requiring a permanent pacemaker		In the cTn I −/− group, no significant reduction in ejection fraction was observed during the follow-up, and there was a very low incidence of cardiac events (1%). In contrast, a greater incidence of cardiac events occurred in cTn I-positive patients, particularly in the cTn I+/+ group (84% vs. 37% in the cTn I+/− group; *P* < .001).<br><br>cTn I release pattern after high-dose chemotherapy identifies patients at different risks of cardiac events in the 3 years thereafter. This stratification allows differentiation in the

				further stratified on early vs. late cTn I rise after high-dose chemotherapy .<br><br>cTn I was measured before and soon after each cycle of high-dose chemo (early troponin; E-Tn I). Determination of E-Tn I consisted of a curve of assays: a baseline and five successive samples during the 3 days after chemotherapy					monitoring programme and to plan, in selected patients, preventive strategies aimed at improving clinical outcome.

				infusion (immediately after and 12, 24, 36, and 72 h later). This sequence was repeated after each cycle of therapy. For each patient, only the highest E-Tn I value was considered.					
Apple, 2005	15563477	Observation Case-control	*N* = 9	Serial ECG and cTn I determinations over 12–24 h after presentation for both the initial or reinfarction events	In-hospital myocardial reinfarction (MI extension) within 4 days of the initial MI event	The serial cTn I and CK-MB concentrations vs. time profiles for each patient during both their initial MI presentation and the reinfarction events	Biomarkers in diagnosis of early reinfarction	More extensive clinical information was not available	cTn I is sufficient as an individual cardiac biomarker to rule in and rule out MI and/or reinfarction in clinical practice.<br>CK-MB analysis is not clinically relevant, or cost-effective, in the differential diagnosis of reinfarction when cTn monitoring is available.

									The peak cTn I at 24 h after onset of chest pain returned to normal 3 days after the event.
Lim, 2006	16557160	Prospective cohort study	*N* = 115	The intrarater and inter-rater reliability of ECG interpretation	All consecutive critically ill patients admitted to a 15-bed general medical-surgical ICU	Chance- and crude-corrected agreement	In critically ill patients, the reliance on the ECG for the diagnosis of myocardial ischaemia and infarction is problematic	The reliability of ECG interpretation by a group of intensivists may generate different results	ECG interpretation in critically ill patients for the presence of myocardial ischaemia or infarction showed moderate reliability at best; however, there was high reliability for specific ECG changes. Knowledge of the patient's troponin values increased the reliability for all studied ECG changes and resulted in a statistically significant increase in the inter-rater reliability for diagnosing myocardial ischaemia or infarction.
Pastores,	17448238	Retrospective	86	Clinical diagnosis vs.	All patients who died in the medical-	Univariate	Most common discrepancies caused	Selection bias that may have occurred;	Discrepancy rate of 26% between the

2007		cohort study		post-mortem diagnosis	surgical ICU and had an autopsy performed	comparison	by cardiac complications: thrombotic endocarditis, MI, and HF	physicians and family members of patients with pre-mortem diagnostic uncertainty would have been more likely to pursue an autopsy than in cases in which all parties were certain of the diagnoses and the outcome was predictable	clinical and autopsy diagnoses in critically ill cancer patients.<br>Opportunistic infections and cardiac complications were the most commonly missed major diagnoses.
Lim, 2008	18318915	Prospective cohort study	103	Screening cTn measurement s and ECGs	All consecutive patients admitted to the ICU	Univariate comparisons, multivariable regression analysis, and a sensitivity analysis	Screening detected additional MIs not diagnosed in practice, reflecting 62.2% of MIs ultimately diagnosed	Relatively small number of patients in each subgroup	Systematic screening detected elevated cTn measurements and MI in more patients than were found in routine practice. Elevated cTn was an independent predictor of hospital mortality.
Peacock, 2008	18480204	Retrospective observational cohort study	84 872	Cardiac troponins at admission and adverse events	Inclusion criteria:<br>• Admission for HF<br>• cTn I or T measured at	Univariate comparisons, logistic regression	All-cause in-hospital mortality		Positive troponin associated with higher in-hospital mortality and higher troponin was associated with higher

					initial evaluation<br>Exclusion criteria:<br>• Serum creatinine level >2.0 mg /dL (176.8 μmol/L)				mortality
Marcelino, 2009	19393476	Observational cohort study	704	Presence of TTE-confirmed cardiac pathology	General ICU hospitalization	Descriptive statistics and a multivariate logistic regression analysis	LV systolic dysfunction was one of the more frequently detected abnormalities	Only patients with known diagnoses, previous to admission, were considered to have suspected cardiac abnormalities	One-third of the patients studied presented with cardiac structural or functional alterations and 7.5% with severe previously unknown diagnoses
Rennyson, 2010	20890196	Retrospective observational cohort	2243	ECG diagnosed STEMI	Medical, surgical, trauma, and neurosurgical ICU patients	Univariate analysis	The cardiologist disagreed with the computer interpretation in 60.9% of cases	The timing of troponin testing was not standardized nor were the reasons for the ECG acquisition.<br>The majority of patients did not undergo coronary angiography.	STEMI in the ICU is a relatively common electrocardiographic reading both by standard interpretation software and by expert evaluation.<br>The electrocardiographic STEMI diagnosis

									seems to be a non-specific finding in the ICU that is frequently the result of a variety of non-ischaemic processes.
Szummer, 2010	20210836	Nationwide coronary care unit registry in Sweden	*N* = 54 477	Renal function staged according to the National Kidney Foundation's Kidney Disease Outcomes Quality Initiative (NKF KDOQI) definition (normal, mild, moderate, severe, renal failure requiring dialysis)	Consecutive patients admitted with a MI to a coronary care unit between 2003 and 2006 with available creatinine measurement or information on dialysis (90.9% of all patients)	Logistic regression models using multivariable analyses to assess the association between renal function and in-hospital therapies. Logistic regression model to examine the association between renal function stage and in-hospital complications.	Clinical presentation (Killip class; STEMI vs. NSTEMI), therapy, and in-hospital complications in relation to renal function		Renal insufficiency influences the presentation and reduces the likelihood of receiving treatment according to current guidelines. Short-term prognosis remains poor.

Devereaux, 2011	21502650	Prospective cohort study	8351 patients undergoing non-cardiac surgery	Occurrence vs. no occurrence of post-operative MI; MI with vs. without ischaemic symptoms	Patients who underwent non-cardiac surgery; aged ≥45 years; expected length of hospital stay of ≥24 h; with or at risk for atherosclerotic disease	Multivariable regression analysis adjusting for pre-operative characteristics (statin use, age >70 years, emergency or urgent surgery, serum creatinine >175 μmol/L, history of congestive HF) and intra-/post-operative events (clinically important hypotension, serious bleeding, stroke, or clinically important bradycardia)	30-day mortality		30-day mortality was higher in patients with vs. without peri-operative MI (11.6% vs. 2.2%, *P* < .001) and did not differ between patients with peri-operative MI with vs. without ischaemic symptoms (9.7% vs. 12.5%).
Rodes-Cabau 2011	21565636	Prospective registry	*N* = 101	Elevated biomarkers	Inclusion criteria:<br>• Undergoing	Stepwise linear regression	Assess incidence and predictors of large		Any myocardial injury:<br>• 99% (transfemoral:

				and size of elevation	TAVR.<br>Exclusion criteria:<br>• Death within 24 h before biomarkers obtained<br>• Major procedural complications	analysis used to identify factors associated with greater myocardial injury.<br>ROC curve analysis performed to discriminate power of the cardiac enzyme rise for LVEF decrease in follow-up.<br>Multivariate Cox regression analysis used to determine the predictors of mortality in long term follow-up.	elevations and clinical relevance of elevated biomarkers after TAVR		97%, transapical: 100%)<br>Predictors of large rise in biomarkers:<br>• Transapical approach<br>• Baseline renal dysfunction<br>Degree of rise in cTn independent predictor of cardiac mortality at 9–10 months of follow-up (HR 1.14 per each increase of 0.1 g/L, 95% CI 1.02–1.28.
deFilippi, 2012	22791885	Cross-sectional study	*N* = 148	Elevated cTn T or I	Outpatients with stable chronic kidney disease (CKD) not requiring	Simple and multiple linear regression (adjusting for	CAC score; LVMI; renal function (eGFR and UACR)	n/a	Prevalence of prior coronary disease events, CAC score, and LVMI were higher with

					renal replacement therapy. Eligibility: age ≥30 years and sustained (≥3 months) reduction in eGFR <60 mL/min/1.73 m²). Exclusion: stage 5 kidney disease or renal replacement therapy; history of MI within 90 days of enrolment; history of CABG.	age, sex, race, diabetes, CAD history, smoking, eGFR using serum cystatin C, and urine albumin-creatinine ratio [UACR] estimated the association between cardiac imaging (coronary artery calcium [CAC] and left ventricular mass index [LVMI]) and hs cTn I or cTn T. Univariate and multivariate linear regression (adjusting for age, sex, race, diabetes, coronary disease history,			increasing concentrations from both hs-cTn I and T (*P* < .05 for all). After adjustment for demographics and risk factors, both remained associated with LVMI as well as eGFR and UACR.

						and LVMI) analyses determined correlations between renal function and hs-cTn concentrations.			
Keddis, 2013	23137067	Prospective registry.	*N* = 1206	Elevated vs. non-elevated pre- or post-transplant cTn T	Adult recipients of kidney grafts between 2000 and 2010 included. Recipients of other allografts prior to, simultaneous, or after the kidney transplant were excluded.	Survival analyses were performed using Kaplan–Meier plots and Cox proportional hazard models	Risk of post-transplant death/cardiac events.		Higher cTn T (pre) was associated with increased risk of post-transplant death/cardiac events independent of cardiovascular risk factors. Elevated post-transplant cTn T was associated with reduced patient survival at 3 weeks and 1 year. Negative/positive predictive values for high cTn T were 91.4%/50% respectively.
Nagele, 2013	23895816	Prospective	*N* = 608 patients	Pre-operative hs-cTn T	Patients with known CAD or	Multivariate linear regression	3-year mortality		3-year mortality was higher in patients with

		cohort study	undergoing major non-cardiac surgery	concentration < vs. >14 ng/L	multiple risk factors for CAD scheduled for major non-cardiac surgery under general anaesthesia	model adjusting for 14 pre-specified predictors of cardiac risk (forced entry)			elevated (>14 ng/L) vs. non-elevated (<14 ng/L) pre-operative hs-cTn T (adjusted HR 2.17, 95% CI 1.19–3.96)
Parmentier-Decrucq, 2013	23587445	Prospective, observational study.	*N* = 184	Adverse events during intrahospital transport	Consecutively critically ill patients on mechanical ventillation	Univariate analysis and a logistic regression with Wald method analysis as independent variables	Adverse events occurred in 45.8% of intrahospital transports	The main bias is the fact that some patients in need of a procedure requiring transport may have had their transport denied by senior physicians because it was estimated to be too dangerous given the severity of the patient	The intrahospital transports of critically ill patients leads to a significant number of adverse events
Riberio, 2013	23954337	Multicentre retrospective registry of patients with coronary obstruction after TAVR	*N* = 44	Coronary obstruction after TAVR vs. no coronary obstruction	TAVR	Propensity matching using the Bootstrap technique to compare patients with obstruction vs. those without	Predictors, causes, and outcomes of patients with coronary obstruction after TAVR		Coronary obstruction: 44/6688 (0.66%).<br>Variables associated with coronary obstruction: older age; female sex; no previous CABG; balloon-expandable valve; prior surgical aortic

									bioprosthesis; low lying coronary ostia; shallow coronary sinus<br><br>30-day mortality: 40.9%.<br><br>Intermediate-term mortality (mean 12 months, range 2–18 months: 45.5%.
Kim, 2014	25060368	Prospective cohort of TAVR patients	*N* = 61	New LGE on MRI after TAVR vs. no new late enhancement	Inclusion criteria:<br>• Severe aortic stenosis scheduled for TAVR.<br><br><u>Exclusion criteria:</u><br>• Contraindicatio n to CMR<br>• Concomitant PCI<br>• Presence of intracardiac thrombi<br>• Conversion to open heart	ROC curve used to analyse for discrimination of new MIs with biomarkers.<br><br>Threshold for hs-cTn T calculated using the Youden index.		Assess incidence of and predictors of myocardial injury as defined by LGE on MRI after TAVR	Prevalence of new myocardial late enhancement with an ischaemic pattern: 11/61 patients (18%).<br><br>Significant change in LVEF pre- and post procedure new myocardial late enhancement but no change in patients without new enhancement.<br><br>ROC curve of maximal hs-cTn T maximum values for detection of

					surgery<br>• Major complications associated with myocardial injury<br>• Need for CPR<br>• Need for extracorporeal circulation.				new late enhancement with AUC of 0.76 (95% CI 0.57–0.96).<br>hs-cTn T 209 ng/L with sensitivity of 71.4% and specificity of 78.8%.
Champion, 2015	25202025	Retrospective observational study.	*N* = 1314	With vs. without stress cardiomyopathy	Critically ill patient admitted to the ICU	Univariate analysis	Haemodynamic impairment is the leading symptom that suggests the diagnosis	Without systematic echocardiographic screening and definitive diagnostic criteria, the 1.5% frequency observed in the study remains imprecise	Stress cardiomyopathy is an infrequent (1.5%) transient LV impairment in the critical care setting
Felker, 2015	26333655	Prospective cohort	*N* = 1074	Baseline hs-cTn T above or below the median and by change in troponin	Inclusion criteria:<br>• Acute heart failure<br>• Mild-moderate renal dysfunction<br>• Systolic blood	Linear regression, Cox proportional hazards	Association between hs-cTn T and change in patient-reported dyspnoea from baseline (VAS AUC), cardiovascular death or hospitalization due to		Higher baseline or peak hs-cTn T and greater peak change in hs-cTn T were each associated with worse outcomes at 60 and 180 days and less improvement in

					pressure >125 mmHg<br>• Receiving IV furosemide ≥40 mg (or equivalent).<br>Exclusion criteria:<br>• Treatment with other intravenous HF drugs or mechanical support within 2 h of screening<br>• Active infection<br>• Pulmonary or valvular disease<br>• Acute HF due to significant arrhythmias<br>• ACS within 45 days<br>• A troponin concentration three or more		HF/renal failure at 60 days, and cardiovascular death at 180 days		dyspnoea

					times higher than the level diagnostic of MI.				
Ribeiro, 2015	26541917	Prospective registry	*N* = 1131	Cardiac CK-MB elevations	Inclusion criteria:TAVR.<br><br>Exclusion criteria: death within 24 h precluding CK-MB assessment.	Predictors of large rise in biomarker determined using a linear regression analysis.<br><br>Univariate and multivariate logistic regression analyses used to determine the predictors of 30-day mortality.<br><br>Univariate and multivariate Cox proportional hazard models used to determine the predictors of	To determine the incidence, clinical impact, and factors associated with cardiac biomarker elevation post TAVR		Any myocardial injury: 66%.<br><br>Predictors of large myocardial injury:<br>• Transapical approach<br>• Early TAVR experience<br>• Procedural complications<br>• Major/life-threatening bleeding<br>• Conversion to open-heart surgery<br><br>Overall and cardiovascular higher CK-MB was associated with impaired LV function at 6 and 12

						cumulative late overall and cardiac mortality.<br><br>Kaplan–Meier estimates used to compare mortality rates between groups using the log-rank test.<br><br>Correlation between LVEF and CK-MB rise with the Pearson's correlation.			months.<br><br>Higher CK-MB independently associated with 30-day and late overall mortality and cardiac mortality.
Sinning, 2016	25671517	Prospective registry	276	VARC-2 defined myocardial injury	• Inclusion criteria:TAVR.<br><br>Exclusion criteria:<br><br>• Procedural death<br>• Conversion to open surgery		To assess timing, predictors, and prognostic value of PMI		VARC-2 injury: 143/276, 51.8%.<br><br>Predictors of VARC injury:<br><br>• Self-expanding prosthesis<br>• CAD<br>• Higher LVEF

									• Procedure time<br>VARC-2 defined injury was not associated with 30-day or 1-year mortality.<br>Chronic troponin elevation was associated with increased 1-year mortality risk (HR 4.5, 95% CI 2.0–10.0).
Devereaux, 2017	28444280	Prospective cohort study	*N* = 21 842 patients	Peak post-operative hs-cTn T levels <5 ng/L vs. 20–65 ng/L, vs. 65–1000 ng/L, vs ≥1000 ng/L	Inclusion: patients aged ≥45 years, underwent non-cardiac surgery under general or regional anaesthesia. Exclusion: no informed consent.	Cox proportional hazards model. Independent variables included pre-operative and surgical variables previously associated with 30-day mortality.	30-day mortality		Peak post-operative hs-cTn T during the first 3 days after surgery was associated with 30-day mortality; elevated post-operative hs-cTn T without an ischaemic feature was also associated with 30-day mortality
van der Linden, 2017	28893963	Case-control study	*N* = 44	24 subjects with impaired	Not reported	Rhythmic diurnal oscillation	Diurnal troponin T curves fitted and		Impaired renal elimination is not the

				renal function (eGFR <60) vs. 20 reference subjects with preserved renal function		pattern of troponin T was used as a model to assess the contribution of impaired renal elimination to persistently elevated cTn levels in patients with CKD. Hourly blood sampling over 24 h was conducted.	expressed as deviation from their 24-hour midline estimating statistic of rhythm (MESOR) troponin concentration. A validated mathematical framework on the dynamics of diurnal rhythms examined the hypothetical effect of prolonged elimination times (caused by impaired renal function) on the rhythmic oscillation of cTn T.		main driver behind persistently elevated cTn T levels in patients with CKD. This emphasizes the importance of extensive diagnostic workup in all patients with elevated cTn concentrations regardless of their eGFR.
Docherty, 2018	30374693	Prospective cohort study	*N* = 273	hs-cTn I values and dynamic ECG changes	Critically ill patients with co-existing CVD	Univariate comparison, regression and mediation analyses were used to explore relationships between acute physiological derangements,	Peak cTn I for most patients (73%) occurred within 2–3 days of admission to ICU.<br>There was excellent agreement between cardiologists regarding the presence/absence of acute myocardial ischaemia on serial	The power of analyses was restricted by sample size and could be subject to both type I and II errors	Undiagnosed MI occurs in a quarter of critically ill patients with co-existing CVD, which is usually unrecognized in routine care, and is associated with lower long-term survival

						MI, and mortality	ECGs.		
Miller-Hodges, 2018	28978551	Prospective multicentre study. Consecutive patients with suspected ACS.	*N* = 4,726	Renal impairment (eGFR <60 mL/min/1.73m^2): 19% of patients	Inclusion: all patients in whom the attending clinician requested cTn for suspected ACS.<br>Exclusion: diagnosis of STEMI; previous admission during the study period; not resident in Scotland (no hospital records linked with outcomes).	Multivariable Cox proportional hazards models were performed to evaluate the association of eGFR and outcomes	NPV and sensitivity of troponin concentrations below the risk stratification threshold (5 ng/L) at presentation were reported for a primary outcome of index type 1 MI, or type 1 MI or cardiac death at 30 days. PPV and specificity at the 99th centile diagnostic threshold (16 ng/L in women; 34 ng/L in men) were determined for index type 1 MI.		In suspected ACS, hs-cTn identified fewer patients with renal impairment as low risk and more as high risk, but with lower specificity for type 1 MI. Patients with renal impairment and elevated cTn concentrations had a two-fold greater risk of a major cardiac event than those with normal renal function.
Sheth, 2018	29576113	Prospective cohort study	*N* = 60	Matched patients with peri-operative vs. non-operative MI	Patients aged >18 years with peri-operative MI (within 2 weeks after non-cardiac surgery) or non-operative MI that fulfilled the UDMI and	Differences between peri-operative and non-operative MI were assessed using paired Student's t-test	Presence of thrombus at the culprit lesion assessed by OCT		Thrombosis was less common in peri-operative (13.3%) than non-operative MI (66.7%) despite similar underlying plaque morphology

					underwent cardiac catheterization within 3 days of the MI	(continuous variables) or McNemar's test (categorical variables)			(fibroatheroma in 60% vs. 66.7%, respectively)
Strömbäck, 2018	29749753	Retrospective cohort study	*N* = 820	Typical symptoms at both MI events	Patients affected by two MIs	Comparisons between groups (men and women).<br>Multiple logistic regression was used to estimate the association between pre-hospital delay of ≥2 h at the second MI and age, sex, diabetes at the second MI, type of symptoms at the second MI, months between first and second MI, and pre-hospital delay at	The type of symptoms and the time from onset of symptoms FMC in first and second MIs in men and women	The variables registered in the MONICA registry were based on data extracted from medical records, which means that the data were collected by multiple physicians, and this might have influenced the data.<br>42% of the recorded patients have ‘insufficient data’ or ‘not known’ for the variables type of symptoms and time from onset of symptoms to FMC.	Significantly more women (16.2%) than men (10%) reported atypical symptoms at the second MI compared with the first. The time from onset of symptoms to FMC was shorter at the second MI.

						the first MI.			
Twerenbold, 2018	29101287	Prospective multicentre diagnostic study. Unselected patients presenting to the ED with suspected NSTEMI.	*N* = 3254	Renal dysfunction (eGFR <60 mL/min/1.73m^2): 15% of patients	Inclusion: adult patients with symptoms suggestive of MI with an onset or peak within the last 12 h. Exclusion: terminal kidney failure on chronic dialysis, STEMI, missing creatinine measurement, no available hs-cTn T on presentation and after 1 h.	ROC curves assessed the discriminative performance throughout hs-cTn concentrations (at presentation and absolute changes in ≤1 h) to diagnose NSTEMI	Safety of rule-out, accuracy of rule-in, and overall efficacy of the ESC 0/1-h algorithm in patients with renal dysfunction. Application of the official ESC assay-specific cutoff criteria for rule-out or rule-in to calculate diagnostic performance parameters and their 95% CIs.	Final diagnosis was centrally adjudicated by two independent cardiologists using all available information, including cardiac imaging	In patients with renal dysfunction, the safety of the ESC 0/1-h algorithm is high, but specificity of rule-in and overall efficacy are decreased. The safety or overall efficacy of the 0/1-h algorithm was not improved by modifications of the rule-in and rule-out thresholds.
Vallabhajosyula, 2018	30371194	Retrospective cohort study	7.1 million admissions	With vs. without stress cardiomyopathy	Consecutive adults hospitalized with severe sepsis	Regression analysis and a propensity-matched sample.<br>An exploratory analysis for mortality predictors.	Diagnosis of Takotsubo cardiomyopathy was associated with a significantly lower in-hospital mortality compared with patients without Takotsubo, being more prominent in females	Use of an administrative database and ICD-9-CM codes is associated with selection and informational biases.<br>The lack of detailed echocardiographic and coronary angiography	Takotsubo cardiomyopathy is observed with increasing frequency in severe sepsis

								data that are used to define Takotsubo can result in significant overlap with septic cardiomyopathy.<br><br>Coronary angiography is infrequently performed (≈5%) in septic patients because of concomitant haemodynamic instability and kidney injury.	
Weil, 2018	Pe29929614	Non-clinical study (pigs)	13	cTn I levels following increased LV end diastolic pressure (EDP) (IV phenylephrine [PE])	n/a	Repeated measures ANOVA, *post hoc* Holm–Sidak, non-parametric testing	PE infusion resulted in transient LV overload and dysfunction not due to myocardial ischaemia.<br><br>Circulating cTn I rose above the 99th percentile within 30 min and remained elevated at 24 h after increase in LV EDP		cTn I elevation in the absence of ischaemia may be explained by preload-induced myocyte injury

Adamson, 2019	31337669	Prospective multicentre observational cohort study (the Coronary Disease Cohort Study)	*N* = 2140	cTn I concentration at 4 and 12 months following the index event (*n* = 1776)	Hospitalization with an ACS	Cox regression results are reported as HRs per doubling of troponin concentration, both unadjusted and following adjustment for the Global Registry of Acute Coronary Events risk score at time of discharge following index hospitalization	Measurement of cTn during convalescence from an ACS can identify nearly half of patients as low-risk, with 0.5% annual incidence of cardiovascular death. A further 1 in 10 patients have persistent myocardial injury and are identified as very high risk, with annual event rates approaching 10%.	The biomarker used for the index diagnosis of MI varied between study centres	Convalescent cTn concentrations predict long-term cardiovascular death following ACS.<br>Recognizing this risk by monitoring troponin may improve targeting of therapeutic interventions.
Ekström, 2019	31005537	CMR substudy of the third DANAMI (DANish Study of Optimal Acute Treatment of	*N* = 704	CMR 1 day and 3 months after primary PCI	• STEMI<br>• Primary PCI<br>• CMR exclusion criteria	Cox proportional hazards regression models were used to determine the associations among number of LGE areas,	At 3 months, 8.4% of patients had multiple scars.<br>Multiple culprits in STEMI were detected in seven patients (1%), and development of a second non-culprit scar at follow-up occurred in	Only approximately 50% of patients in the DANAMI-3 population included. Results may therefore not necessarily reflect the true incidence of multiple culprits and reinfarctions in an	8.4% of STEMI patients showed signs of multiple myocardial scars, primarily due to known or unknown pre-existing ischaemic heart disease, whereas the incidence of multiple culprits and *de novo*

		Patients with ST-segment Elevation Myocardial Infarction) trial				risk of all-cause mortality, and hospitalization for HF	10 patients (1.4%).	unselected STEMI cohort.	reinfarction was low: approximately 1% each. The presence of multiple scars was independently associated with an increased risk of death and hospitalization for heart failure during 3 years' follow-up.
Tzolos, 2019	31813662	Multicentre prospective observational cohort study	*N* = 78	No stratification applied	Female patients with newly diagnosed invasive breast cancer scheduled to receive anthracycline-based (epirubicin) chemotherapy.<br><br>No exclusion criteria listed.	Correlation and non-linear regression	hs-cTn I concentration at baseline (24 h before cycle 1), during (24 h after anthracycline infusion), and before each treatment cycle.<br><br>The study was underpowered to determine an association between troponin concentrations and clinical events, including death and HF.	In the absence of follow-up cardiac imaging, unable to assess the correlation between increasing troponin concentrations following anthracycline therapy and changes in cardiac function on serial imaging	Following an initial 33% fall 24 h after anthracycline dosing (*P* <.001), hs-cTn I concentrations increased by a median of 50% (*P* < .001) with each successive treatment cycle. In total, 45 patients had troponin measured immediately before the sixth treatment cycle, 21 (46.6%) of whom had hs-cTn I concentrations ≥16 ngLl, indicating

									myocardial injury. Plasma hs-cTn I concentrations before the second treatment cycle were a strong predictor of subsequent myocardial injury.<br><br>Cardiotoxicity arising from anthracycline therapy is detectable in the earliest stages of breast cancer treatment and is cumulative with each treatment cycle. This injury is most reliably determined from blood sampling carried out before rather than after each treatment cycle.
Brunner, 2020	33032237	Prospective cohort study	*N* = 2209	Impaired renal function (eGFR <60 mL/min/1.73 m^2)	Inclusion: patients with suspected stable CAD undergoing ICA. Exclusion: patients with ACS, heart	Ordinal logistic regression for the severity of CAD adjusted by classical cardiovascular	Severity of CAD: number of affected major coronary vessels (CAD classification), SYNTAX, and Gensini score in relation to cTn	n/a	In patients with impaired renal function, blood levels of hs-cTn T/I were significantly associated with the severity of stable CAD

					transplant recipients, and missing troponin values.	risk factors and eGFR were performed with each troponin assay as an independent variable	T/I levels		
Cavefors, 2021	34605611	Prospective observational cohort study	*N* = 411	LV dysfunction	Consecutively critically ill patients	Univariate analysis.<br><br>Logistic regression was used for calculation of the risk of death.	Regional hypokinesia is more common than global hypokinesia in this group and is frequently reversible	The lack of invasive diagnostics for CAD in the majority of the patients with regional hypokinesia	LV systolic dysfunction is commonly triggered by critical illness, is frequently seen as regional hypokinesia, and is linked to an increased risk of death
Song, 2020	32938773	The China PEACE-Prospective AMI Study: prospective cohort study that consecutively recruited patients from 53 hospitals	*N* = 3387	Recurrent AMI classified as early (1–30 days), late (31–180 days), and very late (181–365 days)	Patients admitted for AMI and discharged alive	Time-dependent Cox regression.<br><br>Multivariable modelling was used to identify factors associated with recurrent AMI.	The association of recurrent AMI with 1-year mortality	The study could only include patients who consented to participate.<br><br>Authors did not have information about the quality of care during the recurrent AMI hospitalization, which has important implications for	One-third of recurrent AMI events occurred early. Recurrent AMI is strongly associated with 1-year mortality, particularly if early.

								subsequent outcome.	
Gallacher, 2021	34096969	Consecutive patients with suspected ACS	*N* = 46 927 of whom 4220 had kidney impairment	Cluster-randomized clinical trial with randomization at hospital level (*n* = 10 sites). Random allocation to early vs. late use of hs-cTn I testing, for which results from contemporary assay (standard care) were suppressed and care was guided by the hs-cTn I assay.	All patients whom the treating physician requested a cTn measurement for suspected ACS with valid paired cTn I measurements on both the contemporary and high-sensitivity assay during the index presentation were included. Patients not resident in Scotland were excluded (follow-up via routinely collected sources not captured).	The primary endpoint (12-month event-free survival: reinfarction and cardiovascular death) compared before and after implementation of the high-sensitivity assay using a logistic regression generalized linear mixed model	Primary outcome: occurrence of MI or CV death in relation to study phase (before vs. after use) and kidney impairment		While the frequency of elevated hs-cTn I increased six-fold as kidney function declined from an eGFR of ≥90 to <30 mL/min/1.73 m^2, the proportion attributable to type 1 MI halved. hs-cTn I use did not improve outcomes in patients with elevated levels whether they had kidney impairment or not.

Nair, 2021	34333986	Retrospective observational cohort study	*N* = 6626	Recurrent MI within 90 days of discharge (*n* = 168)	All patients admitted with an early recurrent MI within 90 days of discharge after an AMI between 1 January 2010 and 1 January 2017 at the Cleveland Clinic main campus	Multivariate analysis	5-year survival	Readmissions to hospitals outside the selected health system were not included.<br>The causes of readmission were determined by principal diagnosis billing codes.	Patients who are admitted with an AMI are at the highest risk of developing an early recurrent MI during the first 2 days after discharge. This risk remains elevated during the initial 2 weeks post discharge.<br>Patients who were medically managed had a higher chance of developing reinfarction within 90 days than patients who were revascularized.<br>Predominant mechanisms for reinfarction were stent thrombosis (17%), disease progression (12%), and unchanged CAD (11%).<br>Early recurrent MI has 50% mortality within

									5 years.
Packer, 2021	34053177	Prospective cohort	3636	Baseline hs-cTn T.<br><br>Normal hs-cTn T (≤14 ng/L) compared with abnormal levels divided into tertiles.	Inclusion criteria: Class II-IV HF and EF ≤40% on appropriate therapy	Time to event.<br><br>Covariate adjustment.<br><br>Linear regression, weighted by the number of patients in each group.	Aims: Evaluate the clinical and prognostic significance of hs-cTn T. Evaluate the impact of hs-cTn T level on the efficacy of empagliflozin.<br><br>Primary endpoint of the trial: composite of cardiovascular death or hospitalization for HF.<br><br>Secondary endpoints: HF hospitalizations; slope of the change in eGFR.	Preferentially enrolled patients with EF ≤30%.<br><br>Follow-up hs-cTn T measurements not analysed.<br><br>1% of patients with extreme values for cTn T excluded due to skewness.	hs-cTn T concentration associated with severity and instability of HF, lower Kansas City Cardiomyopathy Questionnaire (KCCQ) scores, higher NYHA class, and worse renal function.<br><br>Linear association between hs-cTn T and combined risk of CV death or HF hospitalization, however troponin levels did not attenuate benefit of therapy.
Wereski, 2021	34167318	Suspected ACS without ST-segment elevation	*N* = 46 092	Implementation of an hs-cTn I assay	1. Suspected ACS<br><br>Exclusion criteria: previously admitted during the trial period; not residents of	Aims:<br><br>• To evaluate the performance of recommend	Troponin concentration and change in troponin concentration	Not pre-specified secondary analysis	Troponin concentrations at presentation have a low PPV for type 1 MI. Change in troponin on serial testing only marginally improves

					Scotland.<br>In this analysis, patients with STEMI; cTn at presentation missing; adjudicators unable to arrive at a consensus for the final diagnosis.	ed cTn thresholds to rule in type 1 MI at presentation.<br>• To determine whether the kinetics of cTn differ sufficiently to discriminate between myocardial injury and infarction.<br>Linear mixed-effects modelling; logistic regression; AUC.			PPV for type 1 MI over presenting troponin alone.<br>Interpretation: Cardiac troponin concentrations at presentation are insufficient to distinguish type 1 MI from other causes of myocardial injury or infarction.
Gallacher, 2022	35271932	Consecutive patients with	*N* = 46 927, of whom 9080	Kidney impairment:	Patients eligible for inclusion:	The index diagnosis and	Type 1 and type 4b MI or cardiovascular death		In patients with hs-cTn I above the 99th centile,

		suspected ACS (pre-specified secondary analysis of a randomized trial)	(19%) had kidney impairment	eGFR <60 mL/min/1.73m^2	presentation with suspected ACS and paired cTn measurements using the contemporary and trial assays. Exclusion: patients who had been admitted previously during the trial, not residing in Scotland, measure of serum creatinine not available.	primary outcome of type 1 and type 4b MI or cardiovascular death at 1 year were compared in patients with and without kidney impairment following implementation of hs-cTn I assay with 99th centile sex-specific diagnostic thresholds	at 1 year		the primary outcome occurred twice as often in those with kidney impairment compared with those without (24% vs. 12%). hs-cTn I testing increased the identification of myocardial injury and infarction but failed to address disparities in management and outcomes between those with and without kidney impairment.
Doudesis, 2023	37169863	Secondary analysis of a multicentre cluster RCT	Training dataset (*n* = 10 038), external validation (*n* = 10 286); patients with possible MI undergoing a	Stratification by age, sex, diabetes, known ischaemic heart disease, CKD, myocardial ischaemia on	Inclusion criteria: (1) age ≥18 years; (2) presentation with suspected ACS; (3) cTn measured using hs-cTn I (Abbott) assay; (4) availability of	Models trained and tested using four statistical methods: logistic regression, naïve Bayes, random forest, and extreme gradient	A clinical decision support tool called CoDE-ACS uses XGBoost to derive a score (0–100) that corresponds to an individual's probability of MI. CoDE-ACS had excellent discrimination	Model training for only a single hs-cTn assay. Confirmation bias may in part explain the excellent performance as CoDE-ACS incorporates features that are integral to the diagnosis of MI.	CoDE-ACS used as a clinical decision support system could reduce unnecessary hospital admissions and reduce inequalities in care that are evident using cTn thresholds to risk stratify patients with

			single or serial hs-cTn I measure	the ECG, and time from symptom onset to presentation	electrocardiographic and physiological data for diagnostic adjudication	boosting (XGBoost) to estimate the probability of type 1, type 4b or type 4c MI during the index hospital admission. External validation in seven international cohorts from Switzerland, Spain, Poland, Czech Republic, Australia, New Zealand and the USA. Performance compared with guideline-recommended approach for risk stratification and diagnosis of MI.	for MI (AUC, 0.953), performed well across all subgroups, and identified more patients at presentation as low probability of having MI than guideline-recommended approaches using fixed cTn thresholds (61% vs. 27%) with a similar NPV and fewer as high probability of having MI (10% vs. 16%) with a greater PPV	Although participants included from across seven countries, the majority were white, and therefore diagnostic performance not evaluated across different ethnic groups. Prospective validation and implementation studies needed.	possible MI. Regulatory clearance is needed before this decision support tool can be used in practice.

Eggers, 2023	37137939	Registry-based cohort study of type 1 and type 2 MI patients managed at Swedish coronary care units	Type 2 MI = 14 833<br><br>Type 1 MI = 184 329	UDMI diagnosis	SWEDEHEART is a Swedish nationwide registry prospectively collecting data from all patients admitted to coronary care units or other specialized facilities because of suspected ACS	Changes in management were assessed using multivariable logistic regression. Adjustment was made for hospital, sex, age, current smoking, diabetes, previous MI, previous coronary revascularization, previous HF, previous stroke, AF upon admission, chronic obstructive pulmonary disease, previous or present cancer, and peripheral	Prognostic outcome was 1-year all-cause mortality	Enrolment 2010–2022; consecutive patients	Compared with type 1 MI patients those with type 2 MI less often had diagnostic examinations and cardioprotective medications. Increases in the use of echocardiography (OR 1.08 [1.06–1.09]) and coronary assessment (OR 1.06 [1.04–1.08]) were smaller compared to type 1 MI ($P_{interaction}$ < .001). The provision of medications did not increase in type 2 MI. All-cause mortality rate in type 2 MI was 25.4% without temporal change. Taken together, the provision of medications and all-cause mortality did not improve in type 2 MI despite modest increases in diagnostic procedures. This

						artery disease. Sub-analyses were conducted in men and women, respectively.			emphasizes the need to define optimal care pathways in these patients.
Holzmann, 2023	37385732	Retrospective cohort study.<br>Patient-level data.	*N* = 7467	Return to the ED with chest pain (*n* = 2017)<br>Recurrent AMI (*n* = 432)	AMI patients discharged alive	Patient characteristics were analysed descriptively	Return to the ED for chest pain in the year following AMI discharge.<br>Recurrent MI within 1 year.<br>Prognosis.	The source population does not include: (1) all patients diagnosed with AMI; (2) all patients presenting to the ED with other symptoms and diagnosed with AMI; or (3) patients who bypassed the ED going directly to the cardiac ICU	3 in 10 AMI survivors returned to the ED for chest pain in the year following AMI discharge.<br>Over 10% of patients with return ED visits were diagnosed with recurrent AMI.<br>One-year all-cause mortality 3.1% in the AMI cohort, 11.6% in the recurrent AMI cohort.
Puelacher, 2023	36705050	Prospective cohort study	7754	Peri-operative MI or injury (PMI) vs. no PMI following non-cardiac	Consecutive patients undergoing major non-cardiac surgery (expected post-operative stay	Multivariable Cox proportional hazards analysis adjusted for age, sex, ESC/ESA	MACE and all-cause death at 1 year		MACE and all-cause death were significantly higher in patients with vs. without PMI, consistently for most

				surgery; aetiology of PMI	of ≥2 days); increased mortality risk defined as ≥65 years of age OR ≥45 years with history of CAD, peripheral arterial disease, or stroke	surgery risk (low, medium, high risk of cardiac events), revised cardiac risk index (RCRI) class, urgency of surgery, post-operative complications. (sepsis, stroke, bleeding), centre, and surgical speciality				PMI aetiologies
Real, 2023	37225294	Observational cohort using a prospective database of TAVR patients at two centres	*N* = 1394	PMI vs. no PMI	Inclusion criteria:<br>• TAVR with new-generation device.<br>Exclusion criteria:<br>• Procedural death<br>• Received first-	Logistic regression analysis to determine predictors of PMI.<br>Cox regression analysis to determine factors	30-day and 1-year mortality using VARC-2 and VARC-3 definitions of PMI			VARC-3 defined MI: 14.0%.<br>Independent predictors of MI:<br>• Female sex<br>• Peripheral artery disease.<br>30-day mortality, MI vs. no MI: HR 2.69, 95% CI

					generation valve<br>• Transaortic or transapical approach	associated with 30-day and 1-year all-cause mortality.<br>Kaplan–Meier estimates to build survival curves for PMI vs. no PMI.<br>Restricted cubic splines analysis to model the association between the fold of troponin increased and 1-year all-cause mortality.			1.50–4.82; *P* < .001).<br>1-year mortality MI vs. no MI: HR 1.54, 95% CI 1.04–2.27; *P* = .032.<br>Primary PMI according to VARC-2 criteria had no impact on mortality.
Gallacher, 2024	38426727	Retrospective national data linkage study	N = 16 050 patients with kidney failure, of whom 1992 had incident MI and 996 had incident stroke during	Patients with vs. without incident MI or stroke	All patients with kidney failure aged ≥18 included in the Scottish Renal Registry and receiving KRT (haemodialysis, peritoneal dialysis,	Incidence rates for MI and stroke in patients with kidney failure were estimated according to age, sex, and	Incidence of MI and stroke, and subsequent cardiovascular death		Between 1996 and 2015, the age-standardized incidence of MI per 100 000 decreased in men (from 4376 to 1835) and women (from 3268 to

			the study		or kidney transplantation) for ≥90 days between 1 January 1996 and 31 December 2016 were eligible for inclusion	kidney replacement therapy modality at the time of the event using generalized additive models. Annual incidence rates were calculated for the period 1996–2014.			1369).<br>Predicted probability of cardiovasvular death within 1 year of MI for a 66-year-old patient with kidney failure fell in men (76.6% to 38.6%) and women (76.8% to 38.8%).<br>The incidence of MI and stroke has halved in patients with kidney failure over the past 20 years but remains significantly higher than in the general population. Prognosis remains poor.
Knott, 2024	38954535	Prospective cohort study	*N* = 1992	Presence vs. absence of CKD (eGFR <60 mL/min/1.73 m^2)	ED patients undergoing clinically indicated hs-cTn T measurement	Comparison of diagnostic performance of hs-cTn T-based on adjudication following Fourth UDMI	Diagnostic performance (PPV) of baseline and serial 2 h hs-cTn T thresholds for ruling-in acute MI in patients with vs. without CKD		Diagnostic performance of standard baseline and serial 2 h hs-cTn T thresholds to rule-in MI is suboptimal in CKD patients but improves when using higher baseline thresholds and

									delta values
**OTHER ARTICLES**									
**Study first author surname and year**	**PMID reference for study**	**Type of article** (editorial, commentary, systematic review, narrative review, clinical practice guideline or consensus statement)	**Population**	**Topics**	**Key point (1)** (evidence level if clinical practice guideline)	**Key point (2)** (evidence level if clinical practice guideline)	**Key point (3)** (evidence level if clinical practice guideline)	**Other considerations** (bias, uncertainty, future research requirements, etc.)	**Interpretation**
Kociol, 2010	20863950	Narrative review	HF	Mechanisms and significance of troponin elevations in HF					Cardiac troponin is a prognostic marker in both acute and chronic HF. Mechanisms of myocardial injury in HF warrant further study.

Januzzi, 2012	22745356	Consensus statement	HF	Causes of troponin elevations in HF	In HF, there are multiple causes of coronary and non-coronary troponin elevations and interpretation should consider clinical context	In acute HF, troponin should be measured to exclude type I MI	Troponin is prognostic in patients with chronic HF		
Davenport, 2020	31961246	Consensus Statements from the American College of Radiology and the National Kidney Foundation		Use of intravenous iodinated contrast media in patients with kidney disease					The risk of acute kidney injury in patients with reduced kidney function following exposure to intravenous iodinated contrast media has been overstated (historic lack of control groups sufficient to separate contrast-induced vs. contrast-associated acute kidney injury).<br>Prophylaxis with intravenous normal saline is indicated for patients with eGFR <30 mL/min/1.73 m^2 not

									undergoing maintenance dialysis, and may be considered in patients with an eGFR of 30–44 mL/min/1.73 m^2.
Jaffe, 2021	33486520	Narrative review	n/a	Use of CK-MB for diagnosis of MI	Measurement of CK-MB offers no additional value to measurement of cTn and the use of CK-MB is rapidly decreasing			n/a	Interpretation: CK-MB has no role in the diagnosis of MI
Kwok, 2021	33606411	Systematic review	Patients with misdiagnosed AMI	AMI diagnosis					1%–2% missed AMI. Reasons include atypical presentation, incorrect ECG interpretation, and failure to order appropriate diagnostic tests
McDonagh, 2021	34447992	Guidelines	Acute and chronic HF	Guidelines for the management	Recommend measurement of cTn T in patients	Patients receiving cardiotoxic			

				of acute and chronic HF	with new-onset acute HF to exclude ACS	cancer treatment should be monitored with troponin			
Halvorsen, 2022	36017553	Clinical practice guidelines	Patients undergoing non-cardiac surgery	Guidelines intended for physicians, healthcare workers, and collaborators involved in the pre-operative, operative, and post-operative care of patients undergoing non-cardiac surgery. The objective is to endorse a standardized and evidence-based approach to peri-operative cardiovascula	These guidelines recommend a stepwise evaluation of the patient that integrates clinical risk factors and test results with the estimated stress of the planned surgical procedure and the risks involved with the discontinuation of drugs	Overall goal: individualized risk assessment, with the opportunity of initiating medical therapy, coronary interventions, and specific surgical and anaesthetic techniques, or withholding medical therapy, in order to optimize the patient's peri-operative condition			

				r management.					
Lyon, 2022	36017568	Guideline	Patients with cancer undergoing anticancer treatments which may have an effect in the cardiovascular system	Help all the healthcare professionals providing care to oncology patients before, during, and after their cancer treatments with respect to their cardiovascular health and wellness. Guideline on the definitions, diagnosis, treatment, and prevention of cancer	Cardiovascular toxicity risk stratification before starting potentially cardiotoxic anticancer therapy is recommended in all patients with cancer (Class IB)	An ECG is recommended in all patients starting cancer therapy as part of their baseline cardiovascular risk assessment (Class IC). ECG, natriuretic peptide (NP), and cTn measurements are recommended in all patients before starting immune checkpoint inhibitor therapy (Class IB). Baseline ECG, NP, and cTn are recommended in	Baseline measurement of NP and cTn is recommended in high- and very high-risk patients prior to anthracycline chemotherapy (Class IB) and anti-HER2-targeted therapies (Class IC). cTn and NP monitoring before every cycle during anthracycline chemotherapy and 3 and 12 months after therapy completion is recommended in high- and very high-risk patients (class IB). NP and cTn measurements are recommended at baseline and every 3–6 months in patients with	Baseline echocardiography is recommended in all patients before anthracycline chemotherapy (Class IB), before HER2-targeted therapies (Class IB), in high- and very high-risk patients treated with vascular endothelial growth factor (VEGF) inhibitors or bevacizumab (Class IC), and high-risk patients scheduled to receive Bruton's tyrosine kinase (BTK) inhibitors (Class IC).	n/a

				therapy-related cardiovascular toxicity (CTR-CVT), and the management of CVD caused directly or indirectly by cancer.		all patients with cancer before starting chimaeric antigen receptor T-cell (CAR-T) and tumour-infiltrating lymphocyte (TIL) therapies (Class IC).	AL-cardiac amyloidosis (Class IB).		
Maslove, 2022	35715504	Review	New conceptual model of critical illness						
Matsushita, 2022	36104509	Review article		CVD in patients with CKD					CKD increases the risk of several CVD outcomes. The broad impact of CKD on the cardiovascular system reflects several pathophysiological mechanisms that link CKD to CVD (shared

									risk factors, altered bone mineral metabolism, anaemia, volume overload, the presence of uraemic toxins). Implementation gaps to be addressed include albuminuria screening, inadequate implementation of guideline-directed medical therapies, and access to evidence-based medications.
Akuka, 2023	37611416	Case series and review article		Summary of patients with coronary embolism					Summary of data:<br>• Coronary embolism as cause of MI is infrequent, occurring in about 1% of cases<br>• Majority occur intra-procedure<br>• Majority present with STEMI and/or hypotension

Ndumele, 2023	37807920	Scientific statement from the AHA		Cardiovascular-kidney-metabolic syndrome	Inter-relatedness of metabolic risk factors (obesity, diabetes), CKD, and CVD				Important management considerations for patients with established CVD and coexisting metabolic risk factors, CKD, or both. Gaps in knowledge include mechanisms of disease development; heterogeneity within clinical phenotypes; optimal clinical care in terms of early-life prevention, interdisciplinary care models, and emerging cardioprotective and kidney-protective therapies.
Jiang, 2024	38135114	Review	Patients undergoing cancer treatment and developing CAD	Effects of various chemotherapeutic agents on the coronary arteries and	The most frequent pathological manifestations of chemotherapy-mediated coronary damage include	Potential screening techniques for CAD patients include baseline risk factor	Cardiology-oncology guidelines offer some suggestions for the care of CAD, which might involve medication, lifestyle	Further *in vivo* and *ex vivo* experimental studies are needed to understand the coronary toxicity and mechanisms of	n/a

				pathological manifestations of chemotherapy-mediated coronary damage	acute vasospasm, acute thrombosis, accelerated atherosclerosis development, and microvascular dysfunction	evaluation, polygenic risk factors, and coronary artery calcium scores	changes, and coronary revascularization	chemotherapeutic agents	
Thompson, 2024	39316661	AHA/ACC Guideline	Patients undergoing non-cardiac surgery	Recommendations to guide clinicians in the peri-operative cardiovascular evaluation and management of adult patients undergoing non-cardiac surgery	A stepwise approach to peri-operative cardiac assessment assists clinicians in determining when surgery should proceed or when a pause for further evaluation is warranted	Cardiovascular screening and treatment of patients undergoing non-cardiac surgery should adhere to the same indications as non-surgical patients, carefully timed to avoid delays in surgery and chosen in ways to avoid overscreening and overtreatment	Myocardial injury after non-cardiac surgery is a newly identified disease process that should not be ignored because it portends real consequences for affected patients		

6

7

5. Evidence tables for sections 17 to 20

Section	Sections 17 to 20 (Implications for patients, public health, low-resource settings, and research)								
**RANDOMISED CONTROLLED TRIALS**									
**Study first author surname, year, and acronym**	**PMID reference for study**	**Population**	**Number of participants**	**Intervention and control**	**Key inclusion & exclusion criteria**	**Type of analysis** (ITT, , ITT or PP with adjustment for pre-specified covariates)	**Relevant outcome(s) and PROMS**	**Other methodological aspects** (allocation concealment, single/double blinding, sham procedure in device trials, missing data on outcomes, outcome central adjudication…)	**Key findings and interpretation**
Kunadian, 2024	39225274	Patients aged ≥75 with NSTEMI at 48 sites in the United Kingdom	*N* = 1518	Invasive strategy of angiogram and revascularization and best medical therapy vs. best medical therapy only	Patients with a type 1 NSTEMI during the index hospitalization were included. Patients were excluded if they had STEMI, unstable angina or cardiogenic	ITT	The primary outcome, assessed in a time-to-event analysis, was a composite of cardiovascular death or non-fatal MI, as defined by the Fourth UDMI	Cumulative incidence was estimated with Kaplan–Meier methods, and the assigned treatment strategies were compared with the use of a log-rank test stratified according to the Rockwood frailty status at baseline. The effect of an invasive strategy as compared with a conservative strategy was estimated	In older adults with NSTEMI, an invasive strategy did not result in a significantly lower risk of cardiovascular death or non-fatal MI (the composite primary outcome) than a conservative strategy over a median follow-up of 4.1 years

					shock, had a life expectancy of less than 1 year, were previously included in the SENIOR-RITA trial, or were deemed to be unable to undergo ICA			with the use of a proportional-hazards model, with adjustment for Rockwood frailty status, and the results are presented as HRs and 95% CIs. There was no adjustment for multiplicity, and the widths of the confidence intervals should not be used to infer treatment effect.	
Carson, 2023	37952133	MI patients with Third UDMI definition with haemoglobin <10 g/dL	*N* = 3504	Transfusion at either <8 or 7 g /dL with angina or <10 g/dL	Patients with type 1, 2, 4b, or 4c MI were eligible for enrolment; diagnosis and categorization of MI were performed by site investigators. Patients were ineligible for enrolment if	All the analyses were conducted in the ITT population with two-sided hypothesis tests for superiority	The primary outcome was a composite of MI or death from any cause up to 30 days after randomization. Death was ascertained from medical records during the index hospitalization and by telephone follow-up at 30 days after randomization, with subsequent review of medical records.	After randomization, the transfusion strategy was not masked to site investigators or patients	In patients with AMI and anaemia, a liberal transfusion strategy did not significantly reduce the risk of recurrent MI or death at 30 days

					they had uncontrolled bleeding, were receiving palliative treatment, were scheduled for cardiac surgery during the current admission, or had declined to receive a blood transfusion.				
Chaitman, 2021; ISCHEMIA trial	33267610	Patients with moderate or severe ischaemia on stress testing	*N* = 5179	Early invasive strategy with revascularization and medical therapy vs. medical therapy alone	Patients with positive stress test and absence of LMS >50%. Exclusion criteria included ACS in the previous 2 months,	ITT for primary trial. This was a secondary analysis to assess ISCHEMIA pre-specified aim that the primary and major secondary composite endpoints be analysed using two MI definitions. For procedural MI, the	Compared MI type, frequency, and prognosis by treatment assignment using both MI definitions	Cox regression modelling was used to characterize the association between randomized treatment strategy and time to first occurrence of an MI. Unadjusted and adjusted HRs (95% CI) and *P* values are reported for comparing	In ISCHEMIA, type 1 MI events using the primary and secondary definitions during 5-year follow-up were more frequent with an initial conservative strategy and associated with subsequent cardiovascular death. Procedural MI rates

					angina that could not be controlled with medical therapy, ejection fraction <35%, eGFR <30 mL/min, or severe valvular disease.	primary MI definition used CK-MB as the preferred biomarker, whereas the secondary definition used cTn. Procedural thresholds were >5 times the URL for PCI and >10 times for CABG. Procedural MI definitions included (1) a category of elevated biomarker-only events with much higher biomarker thresholds, (2) new ST-segment depression of ≥1 mm for the primary and ≥0.5 mm for the secondary definition, and (3) new coronary dissections >National Heart, Lung, and Blood Institute grade 3. They compared MI type, frequency, and prognosis by		invasive vs. conservative strategies. Each model was adjusted for a set of pre-specified prognostically important baseline covariates that included age at randomization, sex, eGFR, LVEF, and diabetes. To allow for non-linear covariate effects, the continuous variables of age, LVEF, and eGFR were modelled as restricted cubic splines with knots at the approximate 10th, 50th, and 90th percentiles of each variable's empirical distribution.	were greater in the invasive strategy and with the use of the secondary MI definition.

						treatment assignment using both MI definitions.			
Gregson, 2020; EXCEL	33004126	Patients with left main CAD	1858 in trial with 90 with PMI	PCI vs. CABG	Inclusion with LM CAD >70% or 50% and haemodynamically unstable	ITT for main EXCEL trial. This study sought to determine the rates and clinical relevance of PMI using different definitions in patients with LM CAD randomized to PCI vs. CABG surgery in EXCEL	The principal outcome of interest was cardiovascular mortality; all-cause mortality was a secondary outcome given the expected weaker relationship of PMI with non-cardiovascular compared with cardiovascular death.<br>The pre-specified protocol definition of PMI (PMI Prot) required a large elevation of CK-MB, with identical threshold for both procedures. The Third UDMI (types 4a and 5) required lesser biomarker elevations but with supporting evidence of myocardial		The rates of PMI after PCI and CABG vary greatly with different definitions. In the EXCEL trial, the pre-specified PMI protocol was associated with similar hazard after PCI and CABG, whereas PMI using the UDMI definition was strongly associated with mortality after CABG but not after PCI.

							ischaemia, different after PCI and CABG. For the PMI UD, troponins were used preferentially (available in 49.5% of patients), CK-MB otherwise. The multivariable relationship between each PMI type and 5-year mortality was determined.		
**OBSERVATIONAL COHORT STUDIES**									
**Study first author surname, year, and acronym**	**PMID reference for study**	**Population and study type** (case-control, cohort, etc.)	**Number of participants**	**Stratification**	**Key inclusion & exclusion criteria**	**Type of analysis** (covariate adjustment; propensity score matching, weighting, adjustment; trial emulation; quasi-experimental design…)	**Relevant outcomes and PROMs**	**Other methodological aspects** Bias in design (i.e. immortal-time bias, survivorship bias, etc.), risk of residual confounding (i.e. e-value, falsification endpoint), other issues as evaluated in specific tools (i.e. ROBIN I), generalizability	**Key findings and interpretation**

Dang, 2025	40049585	Multicentre cohort study	*N* = 587 patients from 23 Australian and New Zealand sites	SCAD confirmed on core laboratory adjudication	ACS due to SCAD at local site	Multivariable Cox proportional hazard models analysed predictors for the primary MACE outcome	Death from any cause, non-fatal MI (either secondary to SCAD or non-SCAD related MI), non-fatal stroke, HF, or coronary revascularization	Likely to be a selected cohort as no angiographic review of consecutive ACS patients	During a median of 21 months follow-up, MACE and SCAD recurrence occurred in 8.6% and 3.6% of patients, respectively. Oral anticoagulation on discharge (adjusted HR 3.8, 95% CI 1.6–9.3, *P* = .003), ticagrelor combined with aspirin (aHR 1.8, 95% CI 1.04–3.2, *P* = .037), fibromuscular dysplasia (aHR 2.2, 95% CI 1.05–4.5, *P* = .037), and history of stroke (aHR 3.8, 95% CI 1.2–12.2, *P* = .03) were independently associated with higher MACE.
Sepehrvand, 2016	26289822	Cohort from an RCT: Providing Rapid Out of Hospital Acute	*N* = 477	ED physician diagnosis vs. adjudication committee diagnosis	Included: patients with either chest pain or shortness of	The level of agreement between ED and adjudication committee diagnosis was evaluated using	Clinical outcomes (30-day rehospitalization, 30-day and 1-year mortality). No PROMs.	The sample size is modest (based on 55 disagreements and 422 agreements) and should be considered	Despite substantial agreement between the diagnosis of ED physicians and the adjudication committee,

		Cardiovascular Treatment-3 (PROACT-3) Trial. Randomized patients with chest pain or shortness of breath for biomarker testing in the ambulance.			breath were randomized to usual care or assessment of biomarkers analysed on a point-of-care device in the ambulance.<br>Excluded: patients with STEMI.	kappa coefficient and compared with clinical outcomes (30-day rehospitalization, 30-day and 1-year mortality)		hypothesis-generating.<br>In PROACT-3, the adjudication committee had access to similar information available to ED physicians, so the findings may not be generalizable to all adjudication committees using other methods for adjudication.	in the subgroup of patients where there was disagreement, there was significantly worse short-term and long-term mortality
Zeymer, 2024	38768234	The prospective GULLIVE-R study aimed to evaluate adherence to guideline-recommended secondary prevention, physicians' and patients' estimation of cardiac risk,	*N* = 2509.<br>Outpatients were enrolled in 150 German centres 10 months after AMI.	STEMI vs. NSTEMI.<br>Physician vs. patient's perspective of cardiovascula r risk.	Included: signed written informed consent, aged >18 years, had AMI 9–12 months before baseline visit, and did not simultaneousl y participate in any randomized trial. There	The statistical analysis of the GULLIVE-R was exploratory. For metric variables, the mean values, SD, median, maximum, and minimum were stated, where appropriate. Ordinal data were described by the median and quartiles. For categorical data,	Adherence to guideline-recommended secondary prevention, physicians' and patients' estimation of cardiac risk, and patients' knowledge about target values of risk factors after AMI	Registry data subject to measurement error.<br>GUideline adherence and risk assessment after acute myocardial infarction in real LIfe in Germany—a quality improVEment and awareness Registry of the German Cardiac Society (GULLIVE-R) was a prospective registry conducted by	Guideline-recommended secondary drug therapies were prescribed in over 80% of patients, while only about 50% received all five recommended drugs, and regular exercise was performed by only one-third. About 90% of patients felt well informed about secondary prevention,

		and patients' knowledge about target values of risk factors after AMI			were no other exclusion criteria.	absolute numbers and relative frequencies were determined.		the Center for Health Services Research of the German Cardiac Society, the Bundesverband Niedergelassener Kardiologen, and the Institut für Herzinfarktforschung Ludwigshafen. GULLIVE-R has been registered at the German Registry for Clinical studies.	but the correct target value for blood pressure was known in only 37.9% and for low-density lipoprotein (LDL)-cholesterol in only 8.2%. Both physicians and patients underestimated the objective risk of future AMIs as determined by the TIMI risk score.
McCarthy, 2019	30846342	Retrospective study.<br>Aim was to confirm correct diagnosis.<br>Cardiac rehab referral, scheduling, and attendance rates were also recorded	*N* = 359.<br>Patients hospitalized with type 2 MI at Massachusetts General Hospital between October 2017 and May 2018.	Patients referred to cardiac rehab vs. those who were not referred to cardiac rehab	Patients with type 2 MI were included	Descriptive statistics and mean differences were used in the analyses	Referral to cardiac rehab and participation in cardiac rehab.<br>No PROMs.	Retrospective study relied on accuracy of medical records.<br>Extremely low referral rate to cardiac rehab and no participation make findings potentially less reliable.	Of the 321 patients only 21 (6.5%) were referred to cardiac rehab. There was no difference in baseline characteristics, diagnostic testing, or treatment strategies among patients who were or were not referred to cardiac rehab.

		on physician chart review.<br>Differences in baseline characteristics among patients who were referred/not referred were compared.							Of the 21 patients referred to cardiac rehab, none attended.
Murphy, 2019	32063868	Observational study	*N* = 911<br>Admissions were to four hospitals in metropolitan Melbourne, Victoria, and two hospitals in regional Victoria, Australia.	Early convalescence (2–4 months post discharge) vs. late convalescence (6–12 months post discharge) on measures of depression and anxiety	Eligible: those admitted to hospital after AMI, ACS or unstable angina, and those on the waiting list for elective CABG.<br>Excluded: patients with a current episode of severe psychiatric	Decision tree analysis using X^2automatic interaction detection to determine clinically relevant predictors of anxiety or depression at late convalescence was performed	PROMs: depression and anxiety.<br>Secondary outcomes: predictors of depression and anxiety.	Subjective data. There was substantial attrition of participants over time, from 911 at baseline to 723 (79%) by the late convalescent follow-up. There was also considerable missing data for several independent variables, most notably history of depression, financial strain, socio-economic status, and obesity.<br>No record of patients'	Anxiety rates were 43%, 28%, and 27% at the time of the event, early, and late convalescence. Depression rates were 22%, 17%, and 15%, respectively.<br>Factors consistently associated with increased anxiety and depression risk were history of depression, financial strain, poor self-rated health, low socio-economic status,

					illness, those with intellectual disability, and those who did not have sufficient English language to complete the questionnaires.			treatment, if any, for anxiety or depression, including the use of antidepressive or anti-anxiolytic medication, cognitive behavioural therapy, or other treatments.	younger age (<55 years), and smoking.<br>Obesity, diabetes, and social isolation (living alone or being unpartnered) were identified as important albeit less significant risk factors. Neither<br>sex nor event type were predictive of anxiety or depression.
Smedegaard, 2017	28978528	Nationwide retrospective cohort study in Denmark	*N* = 22 394.<br>Patients of working age (30–65 years) discharged after first-time MI in the period 1997 to 2012, who were employed before	Return to work (n = 20 415) vs. no return to work *N* = 1979).<br>Also stratified by age ranges.<br>Incidences were compared with population controls	Only patients who were employed for at least 3 consecutive weeks in a 5-week period before the day of admission with MI were included. The patients were included 30 days after admission.	Logistic regression was applied to estimate the ORs with 95% CIs for risk factors associated with not returning to work, and patients who died during follow-up were considered as not returning to work in this model. Estimates for age and sex were adjusted for comorbidities (HF,	Outcomes were return to work within 1 year after admission with first-time MI and detachment from employment following return to work, respectively	(1) Observational study, and the results represent associations and not causations; (2) return to work and long-term outcomes such as job satisfaction, job type, health- and job-related quality of life, and physical capacity were not measured; (3) other unmeasured potential confounders included smoking, drinking habits, physical	Within 1 year 91.1% of subjects had returned to work, but 1 year after their return 24.2% were detached from employment and received social benefits.<br>Detachment rates were highest in patients aged 60–65 and 30–39, and significantly higher in patients with MI

			admission.	matched on age and sex.	Patients with a previous discharge diagnosis of MI were excluded.	arrhythmia, diabetes mellitus, cerebrovascular disease, CKD, chronic obstructive pulmonary disease, and depression), living alone, invasive coronary procedures (modelled as a categorical variable with four levels: no procedure, coronary angiography, PCI, CABG), education level, income, and time of index admission.<br><br>Stepwise multivariate logistic regression models were used to assess factors independently associated with knowledge level. Variables shown to be significantly associated with		activity levels, BMI, and LVEF; (4) the reason for not returning to work or becoming detached from employment was also unknown. Patients who were close to 65 years of age at time of admission may have retired because of eligibility for early retirement and pension and not because of poor function. Non-randomized groups.<br><br>Data were collected retrospectively from the patients, and thus there is potential for recall bias.<br><br>Relatively small numbers of women, so replications of these results in larger datasets are warranted.<br><br>The psychometric properties for the	compared with population controls.<br><br>Predictors of detachment were HF, diabetes mellitus, and depression. High education level and high income favoured continued employment.

						knowledge level by bivariate analysis were included as potential confounders in the model.		German modified version of the knowledge test have not been tested.	
Albarquoni, 2016	27387122	Cross-sectional design.<br>Patients who participated in the cross-sectional Munich-Examination-of-Delay-in-Patients-Experiencing-Acute-Myocardial-Infarction (MEDEA) study.	*N* = 486<br>Patients with AMI.	Women vs. men. High knowledge vs. low knowledge.	The main inclusion criterion was diagnosis of STEMI as evidenced by typical clinical symptoms, ECG changes, and myocardial biomarker levels.<br>Exclusion criteria were in-hospital STEMI, resuscitation at AMI onset, and language barriers or cognitive	Comparisons were conducted using X^2test for categorical variables and paired t-test for continuous variables. The *P* level was set at .05 for all comparisons. The total number of heart attack warning signs or symptoms and the total number of actions included in the intervention figure identified by participants were also calculated. Multiple linear regression analysis was used to determine the independent predictors of symptom	Knowledge scores. Prehospital delay. Clinical and behavioural variables.	This study used a pre-post design, so it cannot be presumed to illustrate cause and effect as patients' knowledge on the topic may have changed as a result of other factors	High AMI knowledge shortened median delay time in men and women. Almost half of patients demonstrated high AMI knowledge.<br>High-knowledge were independently associated with male sex and general practitioner as a knowledge source, and previous AMI or stent placement were significantly associated with lower knowledge.<br>Although the majority (98%) correctly recognized at least one AMI symptom, 14.2% patients correctly identified all AMI

					impairment impeding patients to answer the questionnaires properly. There were no age restrictions.	knowledge at outcome, using the backwards technique.			symptoms. Additionally, one in three believed that heart attack is always accompanied with severe chest pain.
Gallagher, 2013	22366084	Quasi-experimental, pre-test/post-test design	*N* = 137. Cardiac rehabilitation participants at five hospitals were assessed at programme entry and tailored education was provided using the Heart Foundation of Australia's Heart Attack	Pre vs. post intervention	Patients were considered eligible for the study if they had been discharged from hospital with a CHD diagnosis including AMI, PCI or angina, and were able to understand and speak English sufficiently to respond to the survey and		Knowledge of heart attack warning signs and specific chest pain action plans, for people with CHD attending cardiac rehabilitation	Although the sample is typical of patients recovering from cardiac events in terms of age, sex and marital status, the results cannot be presumed to be generalized to all CHD patients or beyond the current context.<br>Whilst knowledge has improved, it cannot be presumed to equal behaviour change in the face of AMI symptoms.<br>No data such as age, sex, ethnicity etc. are	There were statistically significant improvements in the reporting of 11 of the 14 warning signs of heart attack, with patients reporting 2.56 more warning signs on average at outcome ($P < .0001$). Patients reported more heart attack warning signs if they had completed high school education (˘ = 1.14) or had better knowledge before the intervention (˘ = 0.57).

			Warning Signs campaign educational tool		provide informed consent. Patients who had CABG surgery were excluded.			reported to evaluate generalizability.	There were statistically significant improvements in reporting of all appropriate actions in response to potential AMI symptoms, with patients reporting an average of 1.3 more actions at outcome ($P < .001$), with no change in the median time they would tolerate symptoms ($P = .16$).
Mittag, 2001	11286367	Observational study	*N* = 154. Male patients participating in a cardiac rehabilitation programme in either one of two hospitals in northern Germany served as	Patient viewpoint vs. physician viewpoint on return to work following MI	Patients were selected consecutively on the basis of having suffered from MI or having undergone CABG for the first time. The age group was limited to patients aged	Stepwise multiple regression, or stepwise logistic regression analysis for categorical variables, respectively were used to analyse differences between patients and physicians' viewpoints on return to work. To answer the second question, to	Vocational status 12 months after discharge from the rehabilitation hospital	Recruitment from a cardiac rehabilitation programme only, which excludes the majority of AMI patients, who do not participate in cardiac rehabilitation	Patients' and physicians' views on disabilities and re-employment are based on different factors. The physicians derive their estimates mainly from medical variables (cardiac status and comorbidity), whereas the patients' views are based on the overall health status, their

			subjects.		40–59. 132 of the 154 subjects had been employed prior to the coronary incident (i.e. they had not been retired, unemployed, or ill for more than 6 months).	determine the factors related to the return to work after 12 months, multiple logistic regression analyses were implemented.			former job status, job satisfaction, and negative incentives for the return to work.
Boudrez, 2000	28978528	Observational study.	*N* = 227. Consecutive patients (90 after a first AMI; 137 after a first CABG) were selected for participation. Referral centre for cardiac rehabilitation	Return to work vs. no return to work	All patients were >60 years old and in a social state that still allowed return to work	Descriptive statistics, multiple regression, and logistic regression models	During hospitalization, a set of questionnaires, validated as well as self-developed, was presented, measuring psychological and social variables. Medical variables were collected from the medical records. One year later, a follow-up questionnaire was sent by mail, measuring		Three variables predicted re-employment in 85% of all cases: (1) age, (2) patients' feelings about the extent to which they are disabled by their cardiac problem, and (3) the physicians' views on the extent to which the patient is vocationally disabled by

			at the university hospital in Ghent.				return to work, reasons for not returning, morbidity, and psychological well-being.		their overall medical situation.<br>Return to work was observed in 83.3% of the total study group; 87.2% of the AMI patients and 80.8% of the CABG patients. The mean delay for return to work was 14.8 weeks. After 1 year, patients who returned to work showed more positive affect, less negative affect, fewer somatic complaints, and fewer cognitive complaints. Variables predicting return to work in CABG patients were different from those in AMI patients. Only good LV function and a larger degree of revascularization predicted return to work for CABG patients. Trust, job security,

									positive expectations concerning return, no attribution to stress, fewer somatic complaints, and less physical exertion on the job had predictive power.
McCarthy, 2019	30879022	Observational study	*N* = 633.<br>Patients who were coded as having a type 2 MI between October 2017 and May 2018 at Massachusetts General Hospital.	Comparison of type 2 MI vs. myocardial injury	Inclusion: all those with type 2 MI ICD-10 code I.21.A1.<br>Exclusion: all those with different ICD 10 codes.	Clinical adjudication using strict Fourth UDMI definition	42% of the population had myocardial injury on not true type 2 MI		A large proportion of patients diagnosed with type 2 MI actually had myocardial injury. However, despite differences in definitions, both groups had high readmission rates and mortality rates. This misclassification can have implications on reimbursement penalties for readmission.
Kibirige, 2017	28836972	Observational hospital and pharmacy study	45 Public and private hospitals and 100 private	Assess the availability, affordability, and cost of 37	Sites were chosen mostly from the central region	Proportions of hospitals and pharmacies that had available the studied	Availability of drugs ranged from 21% for heparin to 100% for oral diabetic agents.	Not fully representative of the country and may not be generalizable to other countries	Highlights that many cardiac tests, including troponin, may not be available or affordable

			pharmacies	medicines and 19 diagnostic tests in Uganda	with lesser representation from the eastern, western, and northern regions	medicines and diagnostic tests. Costs were compared with an international standard price. Affordability calculated by number of days wages required for a month of medication or cost of the diagnostic test.	Tests were as low as 6.1% for microalbuminuria. Six of the 19 tests and 10 of 37 medicines were affordable defined as less than 3 days' wages.		in low- and middle-income countries (LMICs)
Chapman, 2017	28034899	Observational study	1218	Patients with suspected ACS underwent hs-cTn I measurement at presentation and 3 and 6 or 12 h	All patients in whom the attending clinician requested cTn for suspected ACS were eligible for inclusion. Did not enrol patients with STEMI, those who were unable to provide consent, or	Baseline characteristics are summarized as mean (SD) or median (IQR) as appropriate. Patients with maximal cTn concentrations ≤99th centile were compared with those >99th centile with the use of a $\chi 2$ test or the Wilcoxon rank-sum test. The primary outcome was the NPV of each pathway using the composite	The primary outcome was a composite of index type 1 MI or type 1 MI or cardiac death at 30 days	Used a bayesian approach with a Jeffreys prior (β distribution with both shape parameters equal to 0.5) because this is more robust when CIs approach 0 or 1	The primary outcome occurred in 15.7% (191 of 1218) patients. In those <99th centile at presentation, the ESC pathway ruled out MI in 28.1% (342 of 1218) and 78.9% (961 of 1218) at presentation and 3 h, respectively, missing 18 index and two 30-day events (NPV, 97.9%; 95% CI 96.9–98.7). The High-STEACS pathway ruled out 40.7% (496 of

					those from outside the region to ensure complete follow-up.	endpoint of index type 1 MI, subsequent type 1 MI or cardiac death at 30 days.			1218) and 74.2% (904 of 1218) at presentation and 3 h, missing 2 index and two 30-day events (NPV, 99.5%; 95CI 99.0–99.9; *P* < .001 for comparison).
Zhan, 2015	25893247	Observational	18 631 AMI admissions from nationally representative sample of hospitals	Availability of biomarkers in Chinese hospitals for AMI and comparison of use of standards for diagnosis of AMI	Random sample of hospitals from five regions in 2011 and traced back to 2006 and 2001. Patients were included if AMI using ICD 9–10, which does not require biomarkers.	Continuous variables were median values. Wilcoxian rank sum and X^2testing. Two-side significance ($P < .05$).	57% of hospitals had biomarkers in 2001 and 96% by 2011. Rates higher in urban areas. Only half of hospitals with troponin used it for more than 80% of eligible patients.		Use of troponin is not universally available or used consistently when available
Dang, 2025	40049585	Cohort study of patients with SCAD from hospitals in Australia and	*N* = 505	None	All patients had ACS and core laboratory adjudication of	Multivariable Cox proportional hazard models analysed predictors of the primary outcome of	Primary MACE outcome (death from any cause, non-fatal MI, non-fatal stroke, HF, or coronary	Observational study with risk of residual confounding	Oral anticoagulation on discharge (adjusted HR 3.8, 95% CI 1.6–9.3) and ticagrelor combined with aspirin

		New Zealand			ICA	MACE	revascularization).		(aHR 1.8, 95% CI 1.04–3.2) were independently associated with higher MACE. These findings support the hypothesis that SCAD is primarily caused by intramural bleeding, with a harmful association of more potent antiplatelet therapy and anticoagulation with adverse cardiovascular outcomes.
**Study first author surname, year, and acronym**	**PMID reference for study**	**Population and study type** (case-control, cohort, etc.)	**Number of participants**	**Stratification**	**Key inclusion & exclusion criteria**	**Type of analysis** (covariate adjustment; propensity score matching, weighting, adjustment; trial emulation; quasi-experimental design…)	**Relevant outcomes and PROMs**	**Other methodological aspects** Bias in design (i.e. immortal-time bias, survivorship bias etc.), risk of residual confounding (i.e. e-value, falsification endpoint), other issues as evaluated in specific tools (i.e. ROBIN I), generalizability	**Key findings and interpretation**

Mueller, 2015	26794254	Observational	*N* = 1282	Patients enrolled if suspected acute myocardial injury and symptoms	Patients presenting to the ED with symptoms suggestive of AMI (such as acute chest pain and angina pectoris) with an onset or maximum of discomfort or pain within the previous 6 h were identified by study personnel and recruited after written informed consent had been obtained. A threshold of <6 h was chosen to enrich the	Prospective international multicentre diagnostic study conducted at 12 sites on three continents to evaluate the test characteristics of definition of suspected MI at admission based on hs-CTn assays at presentation and at 1 h.<br>The NPV and sensitivity for AMI of the hs-cTn T 0-/1-h rule-out rule were the primary outcome measures. Secondary outcome measures included the PPV and specificity of the hs-cTn T 0/1-h rule-in rule and the percentage of patients assigned to	Use of hs-cTn assays at presentation and 1 h later in a population with a 17% rate of AMI classified 63% of patients as having no AMI, with a 99.1% NPV (95% CI 98.2%–99.7%); 14% as having AMI, with a PPV of 77% (95% CI 70.4%–83.0%); and 22.5% as having an indeterminate classification after 1 h of testing		This study validated previous work that serial high-sensitivity troponin values can rapidly help determine likelihood of AMI

					study population with the particularly challenging early presenters. Patients with renal failure requiring long-term haemodialysis; those with trauma, cardioversion, defibrillation, or thrombolytic therapy before inclusion; individuals receiving CABG within the last month or hospitalized for AMI within the last 3 weeks; and	the observational zone.			

					pregnant and breastfeeding women were excluded.				
Bueno, 2018	29663124	Prospective registry study (Europe, Latin America, Asia)	*N* = 11 931 NSTE-ACS hospital survivors	Non-fatal NSTE-ACS	Observational	Kaplan–Meier survival curves. Poisson regression models to estimate 2-year mortality rates in each region, adjusted for 16 known predictors previously obtained from a stepwise Cox regression model.	2-year total mortality	Multiple adjustments for baseline comorbidities, age, sex, medications, and in-hospital complications	Variations in dual antiplatelet therapy at discharge, coronary angiography, and PCI observed. Two-year, risk-adjusted mortality rates ranged between 5.1% (95% CI 2.9%–7.3%) in South Korea, Hong Kong and Singapore to 10.5% (8.3%–12.7%) in Latin America.
Xavier, 2017	28750924	Prospective registry study (Europe, Latin America, Asia)	11 559 STEMI – ACS survivors	Consecutive STEMI patients within 24 or 48 h of symptom onset, who survived to hospital discharge	Observational	Kaplan–Meier survival curves. Poisson regression models to estimate 2-year mortality rates in each region, adjusted for known predictors previously obtained from a stepwise Cox regression model.	2-year total mortality	Multiple adjustments for baseline comorbidities, age, sex, medications, and in-hospital complications	Reperfusion rates ranged between 53.9% and 81.2%, and primary PCI 24.8%–65.6%. 2-year mortality 2.5%–7.4% with regional variations persisting after adjustment.

Xavier, 2008	18440425	Prospective analysis of CREATE Registry Data	20 937 ACS patients, 12 405 with STEMI	STEMI vs. NSTEMI vs. unstable angina	Consecutive patients admitted with ACS, suspected STEMI, NSTEMI, or unstable angina with ECG changes and suspicion of ACS. Excluded patients treated elsewhere first and transferred.	Descriptive statistics for clinical outcomes. All-cause mortality rates at 30 days. Between group comparisons with t-tests or X_2test for continuous and categorical variables. Multivariate logistic regression for predictors of outcomes.	Median time to hospital from symptoms was 6 h. Use of treatments and access differed by income as did outcomes.		Access to diagnostics and treatments limited by income
Yusuf, 2004	15364185	Case control	15 152 cases and 14 820 controls from 52 LMICs as well as high income from all inhabitable continents.	Admission for MI vs. no prior diagnosis	Inclusion: first case of MI admitted to cardiology ward/CCU with symptoms and ECG consistent	Frequency matching of cases and controls based on age-sex strata. Estimated ORs using both conditional and unconditional logistic regression models. Population attributable risks	Nine risk factors (smoking, ApoB/ApoA1, hypertension, diabetes, abdominal obesity, fruit and vegetable consumption, alcohol, and psychosocial factors) accounted for 90% of the population		Impact of the risk factors was consistent across all regions of the world

					with MI. Controls: no prior diagnosis or acute diagnosis of MI.	adjusted for confounders.	attributable risks in men and women.		
**OTHER ARTICLES**									
**Study first author surname and year**	**PMID reference for study**	**Type of article** (editorial, commentary, systematic review, narrative review, clinical practice guideline or consensus statement)	**Population**	**Topics**	**Key point (1)** (evidence level if clinical practice guideline)	**Key point (2)** (evidence level if clinical practice guideline)	**Key point (3)** (evidence level if clinical practice guideline)	**Other considerations** (bias, uncertainty, future research requirements, etc.)	**Interpretation**
**Qualitative Method Articles**									

Gwaltney, 2017	27637486	Qualitative interview study: individual face-to-face interviews were conducted	*N* = 38. Participants who had experienced an MI (with or without ST-segment elevation) within the past 0–6 months (*n* = 17) or 6–12 months (*n* = 21).	Study designed to highlight the symptoms and functional limitations experienced by patients in the year following an MI. This information can support the use or development of PRO instruments in the post-MI population.	Nearly half of all symptom expressions were related to fatigue	Patients reported experiencing general tiredness, as well as a loss of physical energy, mental energy, and/or motivation. Chest pain and discomfort, sleep disruption, and shortness of breath were also frequently reported.	Additionally, patients reported emotional effects, including worry and depression, as well as a negative impact on relationships and social activities	Qualitative methods not designed for generalizability	Patients reported a wide variety of symptoms and functional limitations after an MI. Fatigue was the most commonly reported symptom and included several specific dimensions related to tiredness. Consideration of these concepts associated with the patient's experience following an MI may yield novel endpoints for use in clinical trials and better therapies.
Junehag, 2014	23958430	Qualitative, descriptive design. Individual interviews were conducted.	*N* = 20 Participants (14 men and six women) who lived in rural areas and had experienced	The aim was to describe individuals' perceptions of the psychosocial consequence s of an AMI	Three themes were identified: 1) having a different life, 2) having to manage the situation, and	During their recovery, the participants experienced psychosocial consequences, consisting of anxiety and the fear of being afflicted again	Participants were often more dissatisfied than satisfied with the follow-up provided during recovery	Qualitative methods not designed for generalizability. The interviews were subjected to careful and scrupulous analysis to prevent losing the core elements, and the	After an AMI, follow-up is important during recovery, but the standardized information provided is inadequate. During recovery, people need help dealing with

			their first AMI. Eleven were offered contact with a mentor. The participants were interviewed 1 year after their AMI in Sweden.	and of their access to support 1 year after the event	3) having access to support. Eleven subthemes were labelled.			authors reflected on and discussed every stage of the study to guarantee the trustworthiness of the findings.	existential crises. After discharge, receiving peer support from lay people with similar experiences could be valuable. The knowledge gleaned from this study could be used in education at CCUs and in healthcare outside the hospital setting.
Beza, 2021	34195352	Systematic review	29 studies in LMICs of patients with ACS	Determine the mean length of time from symptom onset until treatment	Mean time from symptom onset to first evaluation by medical provider was 12.7 h	Being older, female, illiterate, rural, and financially limited led to increased delays	Lack of transport systems, poor communication and organization between community facilities, and facilities with appropriate treatment delayed care	Heterogeneity prevented meta-analysis. Some retrospective studies introduce potential bias. English language articles only.	Pre-hospital delays remain a problem in LMICs and lead to poorer outcomes and higher mortality form MI
Lichtman, 2014	24566200	Systematic review and recommendations	A total of 53 individual studies (32 reported on associations with all-	Understanding the association between psychological health and	Clear associations between psychological health and	Increasing evidence that psychological health may be causally linked to biological processes and behaviors that	Interventions to improve psychological health can positively impact CVD risk	Review of literature was not exhaustive and unclear what the method was for limiting studies within the search to review	Reasonable data exist to show associations between psychological and CVD health even if not all mechanisms are clear. The

			cause mortality, 12 on cardiac mortality, and 22 on composite outcomes) and four meta-analyses met inclusion criteria	cardiovascular health and disease	CVD and risk of CVD	contribute to and cause CVD			preponderance of evidence supports the recommendation that the AHA should raise depression to the status of a risk factor for adverse medical outcomes in patients with ACS.
Mendis, 2011	20926369	WHO definition of MI 2008–09 revision	Review of ESC, ACC, AHA and WHF guidelines	Provide guidance for MI definition when biomarkers and ECG not available	Use Category A ESC, ACC, AHA, WHF when all resources are available	Category B when symptoms and ECG Q wave unequivocal	Category C probable when A or B are not an option based on diagnostics		The World Health Organization (WHO) recognizes that in most countries the resources for ECG and biomarkers to make MI diagnosis are frequently not available
Sjöström-Strand, 2011	21175730	Original research.<br><br>Phenomenological study (*n* = 12 women).	Patients who had previously been in a CCU for MI in Southern Sweden	Aim was to explore and describe how women conceived their health and daily life 5	First category identified: consequences of MI	Second category identified: adjustment to a new life	Category 1 included fear and anxiety for the future; suffering from other serious illness; medication and secondary effects; rehabilitation; fatigue	Phenomenological, qualitative methods.<br><br>Small sample.<br><br>Not generalizable.	Women 5 years post MI still struggled with consequences of their MI but reported gratitude for having a second chance at life

				years after an MI			and other health complaints; fearing another MI; downgrading.<br><br>Category 2 included moving forward with difficulties; gratefulness; taking responsibilities for lifestyle changes; recovery process; interaction with family and friends; being aware of the heart; financial stress.		
Legler, 2021	31217805	Prospective observational design (secondary analysis of GRACE data)	*N* = 152 patients with recent ACS	Aims: examine the association between state- and domain-specific gratitude with medical outcomes	State gratitude 2 weeks post ACS was associated with physical activity	Gratitude for one's life was associated with increased self-reported adherence to treatment at 6 months post ACS	Dispositional gratitude was less dynamic and responsive to change over 6 months and not associated with physical activity	Limitations:<br>• Those reporting greater gratitude may, in fact, have been healthier<br>• Single site<br>• Primarily white males	Patients post ACS expressed gratitude for life which was associated with physical activity and adherence to medical recommendations

Dreyer, 2021	34581198	Participatory Action Research design.<br><br>Analysed using a phenomenological approach.	*N* = 42 participants who experienced MI.<br><br>In-depth interviews audio recorded.	Objective was to characterize patients' experience of treatment to develop a new conceptual framework of patient-centred recovery in cardiology	Patients identified both losses and gains post AMI	Losses included the AMI threatened their sense of safety and security and led to social isolation, fragility, uncertainty about the future, and difficulty expressing emotions	Gains included emotional presence, connection, purpose, and a sense of hope and optimism	Limitations:<br>• Theory development requires testing<br>• Single site (Yale-New Haven Health)<br>• Some interviews lasted <20 minutes<br>• Interviews occurred 1–24 months post AMI thus patients were at different points in their recovery	Main finding: feeling like a 'different person' following the AMI
**Modelling Studies**									
Global Burden of Disease study, 2019	33069326	Modelling study	Estimates based on all countries in the world	Burden of disease by mortality, disability, and geography for CVD and other diseases	Global 31.9 million incident cases of ischaemic heart disease	Incidence increased by 32% from 2010 to 2021	Predominant growth in incidence occurred in LMICs	Relies heavily on modelling algorithm for estimates and some countries have limited data for model and thus there are assumptions and imputations that could lead to error	Large increases in ischaemic heart disease, including MI, with the majority estimated to be occurring in LMICs based on population

									growth and ageing in these regions
**Editorials**									
Thygesen, 2021	33602467	Editorial	MI coding	Conflict with ICD codes and Fourth UDMI	In the USA it was advised that STEMI and NSTEMI should all be type 1, leading to a deviation from the UDMI, which acknowledges STEMI and NSTEMI can occur with both type1 and type 2. Need for improvement of ICD-10 coding for type 2 MI for Medicare patients.	No code for non-ischaemic myocardial injury, but 'Non-MI troponin elevation due to underlying cause' R79.89 (other specified abnormal finding). No distinguishing between type 2 STEMI and type 2 NSTEMI.			Need for the Fifth UDMI to be aligned as best possible with ICD-11 coding

Mehta, 2017	29455776	Opinion paper	STEMI patients in India	Overcoming barriers to appropriate STEMI care in India. Comparing LMICs with developed countries.	Major barriers include patient education, insurance, transport, limited capable hospitals and doctors who care for MI	Transport systems could decrease delays to care significantly. Coordination between hospitals and community ambulance is necessary.	Seven key areas for progress could improve access to AMI care, including diagnostics such as troponin		

11
