# Supplementary material for: Fifth Universal Definition of Myocardial Infarction (2026): On behalf of the Joint European Society of Cardiology (ESC)/American College of Cardiology (ACC)/American Heart Association (AHA)/World Heart Federation (WHF) Task Force for the Universal Definition of Myocardial Infarction: Endorsed by the European Association for Cardio-Thoracic Surgery (EACTS) and the Society of Thoracic Surgeons (STS): Affirmation of Value by the Society for Cardiovascular Angiography and Interventions (SCAI)
Source: Glob Heart. 2026 Aug 28;21(1):64. doi: 10.5334/gh.1578 (PMC13523810; doi:10.5334/gh.1578)
Supplement: Supplementary file 2 [file gh-21-1-1578-s2.pdf]

ESC
European Society
of Cardiology

ESC Declaration of Interests Report

Fifth Universal Definition of Myocardial Infarction (2026)

on behalf of the Joint European Society of Cardiology (ESC)/American College of Cardiology (ACC)/American Heart Association (AHA)/World Heart Federation (WHF) Task Force for the Universal Definition of Myocardial Infarction

The framework agreed by the ESC, ACC, AHA and WHF required that at least 50% of the writing task force and 50% of the review panel comply with the *ESC Declaration and Management of Conflicts of Interest Policy* (Appendix 3), including the applicable criteria as of the year preceding their appointment through 2026, to ensure transparency and ethical integrity throughout the process.

Each DOI undergoes a review based on the established criteria, enabling continuous assessment and management of potential conflicts of interest.

Expert		Type of Relationship with Industry
Almahmeed Wael Abdulrahman	**2023**	**Payment from healthcare industry**<br>Pfizer : Atrial Fibrillation (AF)<br>Menarini : Coronary Artery Disease (Chronic) / Chronic Coronary Syndromes (CCS)<br>Servier : Hypertension<br>Bristol Myers Squibb : Myocardial Disease<br>Amgen : Risk Factors and Prevention<br>Novartis : Risk Factors and Prevention<br>Novo-Nordisk : Risk Factors and Prevention<br>**Any other interest (financial or otherwise) that should be declared in view of holding an ESC position**<br>Associate editor for Global Heart and Frontiers in Diabetes, and Journal of Asia Pacific Society of Cardiology<br>**Total impact on personal income or income to an entity controlled personally**<br>0 (none) - 10,000EUR/year
	**2024**	**Payment from healthcare industry**<br>Menarini : Coronary Artery Disease (Chronic) / Chronic Coronary Syndromes (CCS)<br>Servier : Hypertension<br>Bristol Myers Squibb : Myocardial Disease<br>Novartis : Risk Factors and Prevention<br>Abbott Laboratories : Risk Factors and Prevention<br>Novo-Nordisk : Risk Factors and Prevention<br>**Any other interest (financial or otherwise) that should be declared in view of holding an ESC position**<br>Associate editor for Global Heart and Frontiers in Diabetes, and Journal of Asia Pacific Society of Cardiology<br>**Total impact on personal income or income to an entity controlled personally**<br>0 (none) - 10,000EUR/year
	**2025**	Nothing to be declared
Bucciarelli-Ducci Chiara	**2023**	**Payment from healthcare industry**<br>Siemens Healthcare : Cardiac Magnetic Resonance (CMR)<br>GE Healthcare : Cardiac Magnetic Resonance (CMR)<br>Bayer AG : Cardiac Magnetic Resonance (CMR)<br>**Receipt of royalties for intellectual property**<br>Oxford University Press : Cardiac Magnetic Resonance (CMR)

Expert		Type of Relationship with Industry
Bucciarelli-Ducci Chiara	**2023**	**Direct financial interest in healthcare, media, education companies or in companies related to or in competition with the ESC and its mission**<br>Healthcare - Cardiac Magnetic Resonance (CMR) - CEO (part-time) for the Society for Cardiovascular Magnetic Resonance.<br><br>**Total impact on personal income or income to an entity controlled personally**<br>0 (none) - 10,000EUR/year
	**2024**	**Payment from healthcare industry**<br>Siemens Healthcare : Cardiac Magnetic Resonance (CMR)<br>GE Healthcare : Cardiac Magnetic Resonance (CMR)<br>Bayer AG : Cardiac Magnetic Resonance (CMR)<br>**Receipt of royalties for intellectual property**<br>Oxford University Press : Cardiac Magnetic Resonance (CMR)<br>**Direct financial interest in healthcare, media, education companies or in companies related to or in competition with the ESC and its mission**<br>Healthcare - Cardiac Magnetic Resonance (CMR) - CEO (part-time) for the Society for Cardiovascular Magnetic Resonance.<br><br>**Total impact on personal income or income to an entity controlled personally**<br>0 (none) - 10,000EUR/year
	**2025**	**Payment from healthcare industry**<br>Philips : Cardiac Magnetic Resonance (CMR)<br>Siemens Healthcare : Cardiac Magnetic Resonance (CMR)<br>GE Healthcare : Cardiac Magnetic Resonance (CMR)<br>Bayer AG : Cardiac Magnetic Resonance (CMR)<br>Myocardial Solutions : Cardiac Magnetic Resonance (CMR)<br>**Receipt of royalties for intellectual property**<br>Oxford University Press : Cardiac Magnetic Resonance (CMR)

Expert		Type of Relationship with Industry
Bucciarelli-Ducci Chiara	**2025**	**Direct financial interest in healthcare, media, education companies or in companies related to or in competition with the ESC and its mission**<br>Healthcare - Cardiac Magnetic Resonance (CMR) - CEO (part-time) for the Society for Cardiovascular Magnetic Resonance. No competition but synergy with ESC.<br>Other - CEO as contractor for management and leadership activities.<br><br>**Positions of influence in non-ESC organisations and non-ESC journals with a focus in the field of cardiovascular medicine/sciences**<br>Leadership positions:CEO (part-time) for the Society for Cardiovascular Magnetic Resonance. No competition but synergy with ESC.<br>Positions as Editor in Chief:n/a<br>Other editorial positions:n/a<br>**Any other interest (financial or otherwise) that should be declared in view of holding an ESC position**<br>- Minority equity holder, Mycardium AI (start-up) - Minority equity holder, Area19 (start-up) - Minority equity holder, Guilford Street Laboratories (GSL) (start-up)<br>**Total impact on personal income or income to an entity controlled personally**<br>0 (none) - 10,000EUR/year
Colvin Monica	**2023**	**Payment from healthcare industry**<br>Medscape : Chronic Heart Failure, Acute Heart Failure<br>Ebsco : Device Therapy , Chronic Heart Failure , Acute Heart Failure<br>**Research funding from healthcare industry received by department/institution or an entity controlled by department/institution, under direct/personal responsibility**<br>Natera : 1. Funding for investigator initiated research study in heart transplant 2. Participation in multi-center trial in heart transplant , Principal investigator<br>**Any other interest (financial or otherwise) that should be declared in view of holding an ESC position**<br>Associate Editor, Circulation-Heart Failure Clinical lead, Scientific Registry of Transplant Recipients Editorial Board, ACC.org Natera, Steering Committee<br>**Total impact on personal income or income to an entity controlled personally**<br>0 (none) - 10,000EUR/year

Expert		Type of Relationship with Industry
Colvin Monica	**2024**	**Payment from healthcare industry**<br>WebMD : Chronic Heart Failure, Acute Heart Failure<br>Ebsco : Chronic Heart Failure, Acute Heart Failure<br>Seastar : Device Therapy<br>Natera : Other<br>**Payment from healthcare industry to department or institution or any other body for personal services**<br>Natera : Other<br>BrioHealth : Other<br>**Any other interest (financial or otherwise) that should be declared in view of holding an ESC position**<br>Associate Editor, Circulation-HF Editorial board, JACC-HF<br>**Total impact on personal income or income to an entity controlled personally**<br>0 (none) - 10,000EUR/year
	**2025**	**Payment from healthcare industry**<br>Medscape : Chronic Heart Failure, Acute Heart Failure<br>**Research funding from healthcare industry received by department/institution or an entity controlled by department/institution, under direct/personal responsibility**<br>Natera : Heart transplant research comparing cfDNA to biopsies, Site PI<br>**Positions of influence in non-ESC organisations and non-ESC journals with a focus in the field of cardiovascular medicine/sciences**<br>Leadership positions:NA<br>Positions as Editor in Chief:NA<br>Other editorial positions:Associate Editor-Circulation-HF Editorial board JCC-HF<br>**Total impact on personal income or income to an entity controlled personally**<br>0 (none) - 10,000EUR/year
Dangas George	**2023**	**Funding from healthcare industry for travel to, and attendance of events and meetings, unrelated to any specific activity**<br>Daiichi Sankyo : Atrial Fibrillation (AF)<br>**Research funding from healthcare industry received by department/institution or an entity controlled by department/institution, under direct/personal responsibility**<br>Medtronic : RENAL DENERVATION, Site P.I.

Expert	Type of Relationship with Industry	
Dangas George	**2023**	**Membership or affiliation in political or advocacy groups working in the field of cardiovascular medicine/sciences**<br>PRESIDENT OF S.C.A.I. 2022-24 (SOCIETY FOR CARDIOVASCULAR ANGIOGRAPHY & INTERVENTIONS) CHAIR, INTERVENTIONAL CARDIOLOGY COMMITTEE OF THE A.H.A. (AMERICAN HEART ASSOCIATION)<br>**Total impact on personal income or income to an entity controlled personally**<br>>10,000EUR/year
	**2024**	**Payment from healthcare industry**<br>Daiichi Sankyo : Atrial Fibrillation (AF), Valvular Heart Disease<br>**Total impact on personal income or income to an entity controlled personally**<br>>10,000EUR/year
	**2025**	**Payment from healthcare industry**<br>Daiichi Sankyo : Atrial Fibrillation (AF), Valvular Heart Disease<br>**Payment from healthcare industry to department or institution or any other body for personal services**<br>Daiichi Sankyo : Valvular Heart Disease<br>Vascular solutions : Valvular Heart Disease<br>Medtronic : Valvular Heart Disease, Hypertension<br>**Direct financial interest in healthcare, media, education companies or in companies related to or in competition with the ESC and its mission**<br>Healthcare - Coronary Artery Disease (Chronic) / Chronic Coronary Syndromes (CCS) - AMERICAN COLLEGE OF CARDIOLOGY, AMERICAN HEART ASSOCIATION, CARDIOVASCULAR RESEARCH FOUNDATION, SOCIETY FOR CARDIOVASCULAR ANGIOGRAPHY AND INTERVENTIONS<br>**Employment (including part time) in healthcare industry**<br>Mount Sinai Hospital : Professor<br>**Positions of influence in non-ESC organisations and non-ESC journals with a focus in the field of cardiovascular medicine/sciences**<br>Leadership positions:Chair, ACC Audit & Compliance Committee<br>**Total impact on personal income or income to an entity controlled personally**<br>>10,000EUR/year
DeVon Holli	**2023**	**Ongoing research funding from public and/or not-for-profit organisations**<br>National Institute for Nursing Research (USA)<br>**Total impact on personal income or income to an entity controlled personally**<br>0 (none) - 10,000EUR/year

Expert		Type of Relationship with Industry
DeVon Holli	**2024**	**Ongoing research funding from public and/or not-for-profit organisations**<br>National Institute of Nursing Research<br>**Membership or affiliation in political or advocacy groups working in the field of cardiovascular medicine/sciences**<br>AHA<br>**Total impact on personal income or income to an entity controlled personally**<br>0 (none) - 10,000EUR/year
	**2025**	**Ongoing research funding from public and/or not-for-profit organisations**<br>National Institutes of Health<br>**Membership or affiliation in political or advocacy groups working in the field of cardiovascular medicine/sciences**<br>Membership in the American Heart Association and the American Academy of Nursing both of whom are involved in advocacy.<br>**Positions of influence in non-ESC organisations and non-ESC journals with a focus in the field of cardiovascular medicine/sciences**<br>Leadership positions:Member of the AHA Scientific Publishing Committee 2025-2027/<br>Positions as Editor in Chief:No<br>Other editorial positions:Editorial Board Member - JAHA<br>**Total impact on personal income or income to an entity controlled personally**<br>0 (none) - 10,000EUR/year
Dharmakumar Rohan	**2023**	**Ongoing research funding from public and/or not-for-profit organisations**<br>NIH<br>**Payment from healthcare industry**<br>Cardio-Theranostics, LLC : Basic Science<br>**Direct financial interest in healthcare, media, education companies or in companies related to or in competition with the ESC and its mission**<br>Healthcare - Chronic Heart Failure - I co-founded cardio-theranostics, LLC in 2019. The company is in its early phases of developing the science and does not have any products/revenues for the foreseeable future. Should this change in the future I will report it in the subsequent DOIs.<br>**Total impact on personal income or income to an entity controlled personally**<br>0 (none) - 10,000EUR/year
	**2024**	**Research funding from healthcare industry received by department/institution or an entity controlled by department/institution, under direct/personal responsibility**<br>National Institutes of Health : Academic research funding to support cardiovascular research, Principal Investigator

Expert	Type of Relationship with Industry	
Dharmakumar Rohan	**2024**	**Direct financial interest in healthcare, media, education companies or in companies related to or in competition with the ESC and its mission**<br>Healthcare - Acute Coronary Syndromes - Ownership interest in Cardio-Theranositics, LLC (company does not have any revenue / assets)<br><br>**Total impact on personal income or income to an entity controlled personally**<br>0 (none) - 10,000EUR/year
	**2025**	**Ongoing research funding from public and/or not-for-profit organisations**<br>National Institutes of Health (NIH)<br>**Direct financial interest in healthcare, media, education companies or in companies related to or in competition with the ESC and its mission**<br>Healthcare - Chronic Heart Failure - Healthcare - Chronic Heart Failure - I co-founded cardio-theranostics, LLC in 2019. The company is in its early phases of developing the science and does not have any products/revenues for the foreseeable future. Should this change in the future I will report it in the subsequent DOIs.<br><br>**Total impact on personal income or income to an entity controlled personally**<br>0 (none) - 10,000EUR/year
Gaziano Thomas Andrew	**2023**	**Ongoing research funding from public and/or not-for-profit organisations**<br>US National Institutes Of Health<br>**Payment from healthcare industry**<br>Ferrer Internacional : Pharmacology and Pharmacotherapy<br>Multiply Labs : Pharmacology and Pharmacotherapy<br><br>**Membership or affiliation in political or advocacy groups working in the field of cardiovascular medicine/sciences**<br>Chair of Science Committee of the World Heart Federation<br>**Any other interest (financial or otherwise) that should be declared in view of holding an ESC position**<br>I have received consultancy fees from Copenhagen Consensus to edit a document on a health policy document related to the cost-effectiveness of various interventions. It is not a health care company.<br>**Total impact on personal income or income to an entity controlled personally**<br>0 (none) - 10,000EUR/year
	**2024**	**Ongoing research funding from public and/or not-for-profit organisations**<br>US National Institutes Of Health<br>**Payment from healthcare industry**<br>Multiply Labs : Pharmacology and Pharmacotherapy

Expert	Year	Type of Relationship with Industry
Gaziano Thomas Andrew	2024	**Membership or affiliation in political or advocacy groups working in the field of cardiovascular medicine/sciences**<br>Chair of Science Committee of the World Heart Federation<br>**Any other interest (financial or otherwise) that should be declared in view of holding an ESC position**<br>I have received consultancy fees from Copenhagen Consensus to edit a document on a health policy document related to the cost-effectiveness of various interventions. It is not a health care company.<br>**Total impact on personal income or income to an entity controlled personally**<br>0 (none) - 10,000EUR/year
	2025	**Ongoing research funding from public and/or not-for-profit organisations**<br>US National Institutes of Health<br>**Total impact on personal income or income to an entity controlled personally**<br>0 (none) - 10,000EUR/year
Hausenloy Derek John	2023	Nothing to be declared
	2024	Nothing to be declared
	2025	Nothing to be declared
Jeilan Mohamed	2023	**Payment from healthcare industry**<br>Medtronic : Syncope and Bradycardia<br>**Total impact on personal income or income to an entity controlled personally**<br>0 (none) - 10,000EUR/year
	2024	**Payment from healthcare industry**<br>Medtronic : Syncope and Bradycardia<br>**Total impact on personal income or income to an entity controlled personally**<br>0 (none) - 10,000EUR/year
	2025	**Payment from healthcare industry**<br>Medtronic : Syncope and Bradycardia<br>**Membership or affiliation in political or advocacy groups working in the field of cardiovascular medicine/sciences**<br>Secretary general of pan African Society of cardiology appointed Sept 2025<br>**Total impact on personal income or income to an entity controlled personally**<br>0 (none) - 10,000EUR/year

Expert		Type of Relationship with Industry
Koskinas Konstantinos	**2023**	**Payment from healthcare industry**<br>Amgen : Risk Factors and Prevention<br>Daiichi Sankyo : Risk Factors and Prevention<br>Sanofi Aventis : Risk Factors and Prevention<br>**Total impact on personal income or income to an entity controlled personally**<br>0 (none) - 10,000EUR/year
	**2024**	**Payment from healthcare industry**<br>Sanofi Aventis : Risk Factors and Prevention<br>Merck Sharp & Dohme : Risk Factors and Prevention<br>**Payment from healthcare industry to department or institution or any other body for personal services**<br>Daiichi Sankyo : Risk Factors and Prevention<br>Sanofi Aventis : Risk Factors and Prevention<br>**Total impact on personal income or income to an entity controlled personally**<br>0 (none) - 10,000EUR/year
	**2025**	**Payment from healthcare industry**<br>Sanofi Aventis : Risk Factors and Prevention<br>Merck Sharp & Dohme : Risk Factors and Prevention<br>**Payment from healthcare industry to department or institution or any other body for personal services**<br>Daiichi Sankyo : Risk Factors and Prevention<br>Sanofi Aventis : Risk Factors and Prevention<br>**Total impact on personal income or income to an entity controlled personally**<br>0 (none) - 10,000EUR/year
Lindahl Bertil	**2023**	**Ongoing research funding from public and/or not-for-profit organisations**<br>Swedish heart and lung foundation<br>**Total impact on personal income or income to an entity controlled personally**<br>0 (none) - 10,000EUR/year
	**2024**	**Ongoing research funding from public and/or not-for-profit organisations**<br>Swedish heart and lung foundation<br>**Total impact on personal income or income to an entity controlled personally**<br>0 (none) - 10,000EUR/year

Expert		Type of Relationship with Industry
Lindahl Bertil	**2025**	**Ongoing research funding from public and/or not-for-profit organisations**<br>Swedish heart and lung foundation<br>**Total impact on personal income or income to an entity controlled personally**<br>0 (none) - 10,000EUR/year
Mills Nicholas	**2023**	**Ongoing research funding from public and/or not-for-profit organisations**<br>British Heart Foundation Fellowships (FS/IBSRF/23/25161, FS/CRTF/23/24491)<br>**Payment from healthcare industry**<br>Roche Diagnostics : Acute Cardiac Care<br>**Research funding from healthcare industry received by department/institution or an entity controlled by department/institution, under direct/personal responsibility**<br>Roche Diagnostics : Investigator Initiated Study Agreement for analysis of cardiac biomarkers in stored sample from ongoing study (POCET), Chief Investigator<br>**Any other interest (financial or otherwise) that should be declared in view of holding an ESC position**<br>I am an Associate Editor at the American Heart Association journal Circulation for which I recieve modest renumeration for my time commitment (<10k per annum)<br>**Total impact on personal income or income to an entity controlled personally**<br>0 (none) - 10,000EUR/year
	**2024**	**Ongoing research funding from public and/or not-for-profit organisations**<br>British Heart Foundation Fellowships (FS/IBSRF/23/25161, FS/CRTF/23/24491)<br>**Any other interest (financial or otherwise) that should be declared in view of holding an ESC position**<br>I am an Associate Editor at the American Heart Association journal Circulation for which I recieve modest renumeration for my time commitment (<10k per annum)<br>**Total impact on personal income or income to an entity controlled personally**<br>0 (none) - 10,000EUR/year
	**2025**	**Ongoing research funding from public and/or not-for-profit organisations**<br>British Heart Foundation Chair Award (CH/F/21/90010), Research Excellence Award (RE/24/130012), and Programme Grant (RG/F/25/110169)<br>**Total impact on personal income or income to an entity controlled personally**<br>0 (none) - 10,000EUR/year
Motovska Zuzana	**2023**	**Payment from healthcare industry**<br>Idorsia : Acute Coronary Syndromes<br>Boehringer-Ingelheim : Acute Heart Failure

Expert	Year	Type of Relationship with Industry
Motovska Zuzana	2023	**Total impact on personal income or income to an entity controlled personally**<br>0 (none) - 10,000EUR/year
	2024	**Payment from healthcare industry**<br>Janssen-Cilag : Acute Coronary Syndromes<br>Idorsia : Acute Coronary Syndromes<br>TIMI group : Acute Heart Failure<br>**Total impact on personal income or income to an entity controlled personally**<br>0 (none) - 10,000EUR/year
	2025	**Payment from healthcare industry**<br>Janssen-Cilag : Acute Coronary Syndromes<br>Novo-Nordisk : Acute Coronary Syndromes<br>Viatris Innovation GmbH : Acute Coronary Syndromes<br>TIMI group : Acute Heart Failure<br>Novartis : Risk Factors and Prevention<br>**Total impact on personal income or income to an entity controlled personally**<br>0 (none) - 10,000EUR/year
Newby Kristin	2023	**Payment from healthcare industry to department or institution or any other body for personal services**<br>Roche Diagnostics : Acute Cardiac Care<br>Medtronic : Device Therapy<br>Boehringer-Ingelheim : Other<br>**Total impact on personal income or income to an entity controlled personally**<br>0 (none) - 10,000EUR/year
	2024	**Payment from healthcare industry**<br>Boehringer-Ingelheim : Other<br>Bristol Myers Squibb : Other<br>**Payment from healthcare industry to department or institution or any other body for personal services**<br>Roche Diagnostics : Acute Cardiac Care<br>Medtronic : Device Therapy<br>**Total impact on personal income or income to an entity controlled personally**<br>0 (none) - 10,000EUR/year

Expert		Type of Relationship with Industry
Newby Kristin	**2025**	**Payment from healthcare industry to department or institution or any other body for personal services**<br>Roche Diagnostics : Acute Cardiac Care<br>Medtronic : Device Therapy<br>**Total impact on personal income or income to an entity controlled personally**<br>0 (none) - 10,000EUR/year
Rubini Gimenez Maria	**2023**	**Payment from healthcare industry**<br>Beckman Coulter : Acute Heart Failure, Acute Coronary Syndromes<br>**Total impact on personal income or income to an entity controlled personally**<br>0 (none) - 10,000EUR/year
	**2024**	**Payment from healthcare industry**<br>Beckman Coulter : Acute Coronary Syndromes<br>quidel : Acute Coronary Syndromes<br>**Funding from healthcare industry for travel to, and attendance of events and meetings, unrelated to any specific activity**<br>Daiichi Sankyo : Acute Coronary Syndromes<br>**Total impact on personal income or income to an entity controlled personally**<br>0 (none) - 10,000EUR/year
	**2025**	**Payment from healthcare industry**<br>SpinChip : Acute Coronary Syndromes<br>**Total impact on personal income or income to an entity controlled personally**<br>0 (none) - 10,000EUR/year
Salvati Ana Maria	**2023**	Nothing to be declared
	**2024**	Nothing to be declared
	**2025**	Nothing to be declared
Tamis-Holland Jacqueline	**2023**	**Any other interest (financial or otherwise) that should be declared in view of holding an ESC position**<br>JACC Senior Editor-In this, I simply review articles forwarded to me by the associate editors on occasions when they are unsure of the destination for the article to assisst in determining if they should be sent out for review<br>**Total impact on personal income or income to an entity controlled personally**<br>0 (none) - 10,000EUR/year
	**2024**	Nothing to be declared

Expert	Type of Relationship with Industry	
Tamis-Holland Jacqueline	**2025**	**Positions of influence in non-ESC organisations and non-ESC journals with a focus in the field of cardiovascular medicine/sciences**<br>Leadership positions: Vice Chair-AHA Council of Clinical Cardiology<br>Positions as Editor in Chief:None<br>Other editorial positions:Section Editor for Progress in Cardiovascular Disease Guest Editor for special issue on ACS in Current Cardiology Reports<br>**Total impact on personal income or income to an entity controlled personally**<br>0 (none) - 10,000EUR/year
Zaman Sarah	**2023**	**Payment from healthcare industry**<br>Novartis : Interventional Cardiology<br>**Total impact on personal income or income to an entity controlled personally**<br>0 (none) - 10,000EUR/year
	**2024**	**Payment from healthcare industry**<br>Boston Scientific : Interventional Cardiology<br>Novartis : Interventional Cardiology<br>**Total impact on personal income or income to an entity controlled personally**<br>0 (none) - 10,000EUR/year
	**2025**	**Ongoing research funding from public and/or not-for-profit organisations**<br>National Health and Medical Research Council<br>National Heart Foundation of Australia<br>**Payment from healthcare industry**<br>Novo-Nordisk : Other<br>Amgen : Risk Factors and Prevention<br>Novartis : Risk Factors and Prevention<br>**Total impact on personal income or income to an entity controlled personally**<br>0 (none) - 10,000EUR/year

Expert	Type of Relationship with Industry	
Addo Tayo	**2024**	Nothing to be declared
	**2025**	**Employment (including part time) in healthcare industry**<br>University of Texas Southwestern : Professor<br>**Total impact on personal income or income to an entity controlled personally**<br>0 (none) - 10,000EUR/year
Ako Junya	**2024**	Nothing to be declared
	**2025**	Nothing to be declared
Alasnag Mirvat	**2024**	Nothing to be declared
	**2025**	**Positions of influence in non-ESC organisations and non-ESC journals with a focus in the field of cardiovascular medicine/sciences**<br>Leadership positions:Consultant for Ministry of Health, Saudi Arabia<br>Positions as Editor in Chief:Saudi Medical Journal<br>**Total impact on personal income or income to an entity controlled personally**<br>0 (none) - 10,000EUR/year
Al-Lamee Rasha	**2024**	**Payment from healthcare industry**<br>Johnson & Johnson : Acute Coronary Syndromes<br>Medtronic : Coronary Artery Disease (Chronic) / Chronic Coronary Syndromes (CCS)<br>Philips : Coronary Artery Disease (Chronic) / Chronic Coronary Syndromes (CCS)<br>Abbott Vascular : Coronary Artery Disease (Chronic) / Chronic Coronary Syndromes (CCS)<br>Shockwave : Coronary Artery Disease (Chronic) / Chronic Coronary Syndromes (CCS)<br>Cathworks : Coronary Artery Disease (Chronic) / Chronic Coronary Syndromes (CCS)<br>**Total impact on personal income or income to an entity controlled personally**<br>>10,000EUR/year

Expert		Type of Relationship with Industry
Al-Lamee Rasha	**2025**	**Payment from healthcare industry**<br>Johnson & Johnson : Acute Coronary Syndromes<br>Astra Zeneca : Coronary Artery Disease (Chronic) / Chronic Coronary Syndromes (CCS)<br>Medtronic : Coronary Artery Disease (Chronic) / Chronic Coronary Syndromes (CCS)<br>Philips : Coronary Artery Disease (Chronic) / Chronic Coronary Syndromes (CCS)<br>Abbott Vascular : Coronary Artery Disease (Chronic) / Chronic Coronary Syndromes (CCS)<br>Shockwave : Coronary Artery Disease (Chronic) / Chronic Coronary Syndromes (CCS)<br>Cathworks : Coronary Artery Disease (Chronic) / Chronic Coronary Syndromes (CCS)<br>**Any other interest (financial or otherwise) that should be declared in view of holding an ESC position**<br>Company director of ORBITA Systems Ltd<br>**Total impact on personal income or income to an entity controlled personally**<br>>10,000EUR/year
Antman Elliott	**2024**	**Ongoing research funding from public and/or not-for-profit organisations**<br>Horizon Europe--Digital Twin grant<br>**Payment from healthcare industry**<br>Pfizer Medical Affairs----I am authoring a patient resource to navigate and AF Ctr of Excellence---no specific products or treatments are endorsed; this for patient support purposes only. : Atrial Fibrillation (AF)<br>**Receipt of royalties for intellectual property**<br>Elsevier : Pharmacology and Pharmacotherapy<br>**Employment (including part time) in healthcare industry**<br>American Heart Association : Scientific Officer<br>**Total impact on personal income or income to an entity controlled personally**<br>>10,000EUR/year
	**2025**	**Ongoing research funding from public and/or not-for-profit organisations**<br>Horizon Europe--Digital Twin grant<br>**Payment from healthcare industry**<br>Pfizer Medical Affairs----I am authoring a patient resource to navigate and AF Ctr of Excellence---no specific products or treatments are endorsed; this for patient support purposes only. : Atrial Fibrillation (AF)<br>**Receipt of royalties for intellectual property**<br>Elsevier : Pharmacology and Pharmacotherapy

Expert	Year	Type of Relationship with Industry
Antman Elliott	2025	**Employment (including part time) in healthcare industry**<br>American Heart Association : Scientific Officer<br>**Total impact on personal income or income to an entity controlled personally**<br>>10,000EUR/year
Caramori Paulo	2024	**Payment from healthcare industry**<br>Boston Scientific : Device Therapy<br>Medtronic : Valvular Heart Disease<br>**Payment from healthcare industry to department or institution or any other body for personal services**<br>CSL Behring : Risk Factors and Prevention, Pharmacology and Pharmacotherapy<br>**Direct financial interest in healthcare, media, education companies or in companies related to or in competition with the ESC and its mission**<br>Education - Founder and CSO of the e-learning plataform CAAT Medicina (caat.online e App CAAT Med).<br>**Total impact on personal income or income to an entity controlled personally**<br>>10,000EUR/year
	2025	**Payment from healthcare industry**<br>Boston Scientific : Device Therapy<br>Medtronic : Valvular Heart Disease, Hypertension<br>**Direct financial interest in healthcare, media, education companies or in companies related to or in competition with the ESC and its mission**<br>Education - Partner and CMO of CAAT Med, a digital platform and application for physicians and healthcare professionals, focused on Cardiovascular Medicine, offering updated content, risk scores, algorithms, videos, and debates<br>**Positions of influence in non-ESC organisations and non-ESC journals with a focus in the field of cardiovascular medicine/sciences**<br>Leadership positions:Member of the Board of Directors of the Brazilian Society of Cardiology<br>**Total impact on personal income or income to an entity controlled personally**<br>>10,000EUR/year

Expert	Type of Relationship with Industry	
Cohen Mauricio	**2024**	**Payment from healthcare industry**<br>Cordis : Interventional Cardiology<br>Accumed Radial Systems : Interventional Cardiology<br>Asahi Intecc Medical : Interventional Cardiology<br>Edwards Lifesciences : Valvular Heart Disease<br>Abbott Vascular : Valvular Heart Disease<br>**Payment from healthcare industry to department or institution or any other body for personal services**<br>Abbott Vascular : Interventional Cardiology<br>**Any other interest (financial or otherwise) that should be declared in view of holding an ESC position**<br>Editorial Board, Circulation Editorial Board, Circulation Cardiovascular Interventions Editorial Board, American Heart Journal Editorial Board, Journal of the Society for Cardiovascular Angiography and Interventions Editorial Board, Catheterization and Cardiovascular Interventions<br>**Total impact on personal income or income to an entity controlled personally**<br>>10,000EUR/year
	**2025**	**Payment from healthcare industry**<br>Boston Scientific : Interventional Cardiology<br>Cordis : Interventional Cardiology<br>Accumed Radial Systems : Interventional Cardiology<br>Edwards Lifesciences : Valvular Heart Disease<br>Abbott Vascular : Valvular Heart Disease<br>**Payment from healthcare industry to department or institution or any other body for personal services**<br>Abbott Vascular : Interventional Cardiology<br>**Total impact on personal income or income to an entity controlled personally**<br>>10,000EUR/year

Expert		Type of Relationship with Industry
Delgado Victoria	**2024**	**Payment from healthcare industry**<br>Novartis : Chronic Heart Failure<br>Siemens Healthcare : Cross-Modality and Multi-Modality Imaging Topics<br>Philips : Echocardiography<br>GE Healthcare : Echocardiography<br>Merck Sharp & Dohme : Risk Factors and Prevention<br>Abbott : Valvular Heart Disease<br>Edwards Lifesciences : Valvular Heart Disease<br>**Research funding from healthcare industry received by department/institution or an entity controlled by department/institution, under direct/personal responsibility**<br>Astra Zeneca : Research project funded by Astra Zeneca providing an ultraosund system and software for analysis of the data (a bit more than 12000 euros to purchase the material; 2 years= 6K/y) , principal investigator<br>**Membership or affiliation in political or advocacy groups working in the field of cardiovascular medicine/sciences**<br>I am member of AHA I am companion member of PCR<br>**Any other interest (financial or otherwise) that should be declared in view of holding an ESC position**<br>I am senior editor of Circulation - journal of the AHA<br>**Total impact on personal income or income to an entity controlled personally**<br>0 (none) - 10,000EUR/year
	**2025**	**Payment from healthcare industry**<br>Siemens Healthcare : Cross-Modality and Multi-Modality Imaging Topics<br>Philips : Echocardiography<br>GE Healthcare : Echocardiography<br>Abbott : Valvular Heart Disease<br>Edwards Lifesciences : Valvular Heart Disease<br>Products&Features : Valvular Heart Disease<br>**Research funding from healthcare industry received by department/institution or an entity controlled by department/institution, under direct/personal responsibility**<br>Astra Zeneca : 3 year research project on cardiac amyloidosis and use of AI for detection on echocardiography, Principal investigator

Expert		Type of Relationship with Industry
Delgado Victoria	**2025**	**Membership or affiliation in political or advocacy groups working in the field of cardiovascular medicine/sciences**<br>I am member of AHA I am companion member of PCR<br>**Positions of influence in non-ESC organisations and non-ESC journals with a focus in the field of cardiovascular medicine/sciences**<br>Other editorial positions:Senior editor of Circulation (until June 2026)<br>**Total impact on personal income or income to an entity controlled personally**<br>0 (none) - 10,000EUR/year
Dennis Bernard	**2024**	Nothing to be declared
	**2025**	**Positions of influence in non-ESC organisations and non-ESC journals with a focus in the field of cardiovascular medicine/sciences**<br>Leadership positions:Former National Board Chairman AHA<br>Positions as Editor in Chief:none<br>Other editorial positions:none<br>**Total impact on personal income or income to an entity controlled personally**<br>0 (none) - 10,000EUR/year
Ge Junbo	**2024**	Nothing to be declared
	**2025**	Nothing to be declared
Halvorsen Sigrun	**2024**	**Payment from healthcare industry**<br>Sanofi Aventis : Acute Coronary Syndromes<br>Bristol Myers Squibb : Atrial Fibrillation (AF)<br>Pfizer : Cardiovascular Disease in Special Populations<br>Astra Zeneca : Chronic Heart Failure<br>Boehringer Ingelheim : Chronic Heart Failure<br>Novartis : Risk Factors and Prevention<br>Novo-Nordisk : Risk Factors and Prevention<br>**Total impact on personal income or income to an entity controlled personally**<br>>10,000EUR/year

Expert		Type of Relationship with Industry
Halvorsen Sigrun	**2025**	**Payment from healthcare industry**<br>Sanofi Aventis : Risk Factors and Prevention<br>**Payment from healthcare industry to department or institution or any other body for personal services**<br>Bristol Myers Squibb : Acute Coronary Syndromes<br>Boehringer-Ingelheim : Chronic Heart Failure<br>Pharmacosmos : Chronic Heart Failure<br>Novo Nordisk : Risk Factors and Prevention<br>**Total impact on personal income or income to an entity controlled personally**<br>>10,000EUR/year
Harris Julie	**2024**	Nothing to be declared
	**2025**	Nothing to be declared
Ibanez Borja	**2024**	**Any other interest (financial or otherwise) that should be declared in view of holding an ESC position**<br>My center (CNIC) is a public non-profit institution that receives Royalties for the sale of a polypill containing aspirin, ramipril and atorvastatin (trinomia, sincronium). I am not involved in the development I do NOT receive any personal benefit (neither direct nor indirect).<br>**Total impact on personal income or income to an entity controlled personally**<br>0 (none) - 10,000EUR/year
	**2025**	**Positions of influence in non-ESC organisations and non-ESC journals with a focus in the field of cardiovascular medicine/sciences**<br>Leadership positions:member of the BHF Chairs and Grants Committee<br>**Any other interest (financial or otherwise) that should be declared in view of holding an ESC position**<br>My institution (Spanish National Center for Cardiovascular Research, CNIC) is a public non-profit center that received royalties for the sales of a polypill (trinomia). No researcher/worker of the center receives any direct or indirect compensation. I have no personal benefit (direct or indirect) from this initiative<br>**Total impact on personal income or income to an entity controlled personally**<br>0 (none) - 10,000EUR/year

Expert		Type of Relationship with Industry
Jaguszewski Milosz Jaroslaw	**2024**	**Ongoing research funding from public and/or not-for-profit organisations**<br>Medical Research Agency<br>Ministry of Science and Higher Education<br>National Science Centre Poland<br>**Payment from healthcare industry**<br>Ferrer Internacional : Acute Coronary Syndromes<br>AOP Orphan Pharmaceuticals : Acute Heart Failure<br>Pfizer : Atrial Fibrillation (AF), Acute Coronary Syndromes, Pulmonary Circulation, Pulmonary Embolism, Right Heart Failure<br>Adamed : Coronary Artery Disease (Chronic) / Chronic Coronary Syndromes (CCS)<br>Berlin Chemie AG : Coronary Artery Disease (Chronic) / Chronic Coronary Syndromes (CCS)<br>Krka Pharma : e-Cardiology/Digital Health<br>Biotronik : Interventional Cardiology<br>Novartis : Risk Factors and Prevention<br>Novo-Nordisk : Risk Factors and Prevention<br>Polpharma : Training and Education<br>**Funding from healthcare industry for travel to, and attendance of events and meetings, unrelated to any specific activity**<br>Pfizer : Atrial Fibrillation (AF), Interventional Cardiology, European Society of Cardiology<br>Biotronik : Interventional Cardiology, Training and Education<br>Polpharma : Training and Education<br>**Any other interest (financial or otherwise) that should be declared in view of holding an ESC position**<br>I am an Editor-in-Chief of Cardiology Journal.<br>**Total impact on personal income or income to an entity controlled personally**<br>>10,000EUR/year

Expert		Type of Relationship with Industry
Jaguszewski Milosz Jaroslaw	**2025**	**Ongoing research funding from public and/or not-for-profit organisations**<br>Medical Research Agency<br>Ministry of Science and Higher Education<br>National Science Centre Poland<br>**Payment from healthcare industry**<br>AOP Orphan Pharmaceuticals : Acute Heart Failure<br>Pfizer : Atrial Fibrillation (AF), Acute Coronary Syndromes, Pulmonary Circulation, Pulmonary Embolism, Right Heart Failure<br>Adamed : Coronary Artery Disease (Chronic) / Chronic Coronary Syndromes (CCS)<br>Berlin Chemie AG : Coronary Artery Disease (Chronic) / Chronic Coronary Syndromes (CCS)<br>Medtronic : Interventional Cardiology<br>Polimed Research : Interventional Cardiology<br>Novartis : Risk Factors and Prevention<br>Novo-Nordisk : Risk Factors and Prevention<br>Polpharma : Training and Education<br>**Funding from healthcare industry for travel to, and attendance of events and meetings, unrelated to any specific activity**<br>Pfizer : Atrial Fibrillation (AF), Interventional Cardiology, European Society of Cardiology<br>Polpharma : Training and Education<br>**Positions of influence in non-ESC organisations and non-ESC journals with a focus in the field of cardiovascular medicine/sciences**<br>Positions as Editor in Chief:Cardiology Journal<br>**Total impact on personal income or income to an entity controlled personally**<br>>10,000EUR/year
Jneid Hani	**2024**	Nothing to be declared
	**2025**	**Positions of influence in non-ESC organisations and non-ESC journals with a focus in the field of cardiovascular medicine/sciences**<br>Leadership positions:AHA Joint commission on practice guidelines member SCAI publications and research committee<br>Other editorial positions:editorial board JAHA and Current Opinion cardiology<br>**Total impact on personal income or income to an entity controlled personally**<br>0 (none) - 10,000EUR/year

Expert		Type of Relationship with Industry
Katus Hugo	**2024**	**Employment (including part time) in healthcare industry**<br>AaviGen (a gene therapy start up without any income) : chief medical officer<br>**Total impact on personal income or income to an entity controlled personally**<br>0 (none) - 10,000EUR/year
	**2025**	**Employment (including part time) in healthcare industry**<br>AaviGen (a gene therapy start up without any income) : chief medical officer<br>**Positions of influence in non-ESC organisations and non-ESC journals with a focus in the field of cardiovascular medicine/sciences**<br>Leadership positions:Chair of Scientific Advisory Board of the German Heart Foundation and Member of the Board of the German Heart Foundation<br>**Total impact on personal income or income to an entity controlled personally**<br>0 (none) - 10,000EUR/year
Milojevic Milan	**2024**	**Payment from healthcare industry**<br>CORCYM : Other<br>**Any other interest (financial or otherwise) that should be declared in view of holding an ESC position**<br>Director of Clinical Practice Guidelines Program at EACTS<br>**Total impact on personal income or income to an entity controlled personally**<br>0 (none) - 10,000EUR/year
	**2025**	**Payment from healthcare industry**<br>CORCYM : Cardiovascular Surgery<br>**Positions of influence in non-ESC organisations and non-ESC journals with a focus in the field of cardiovascular medicine/sciences**<br>Leadership positions:Director of Clinical Practice Guidelines Program at EACTS<br>Positions as Editor in Chief:NA<br>Other editorial positions:Associate Editor in EJCTS<br>**Total impact on personal income or income to an entity controlled personally**<br>0 (none) - 10,000EUR/year

Expert		Type of Relationship with Industry
O'Donoghue Michelle	**2024**	**Ongoing research funding from public and/or not-for-profit organisations**<br>Amgen<br>AstraZeneca/ medimmune<br>Novartis<br>**Payment from healthcare industry**<br>Astra Zeneca : Other<br>Janssen-Cilag : Other<br>Novo-Nordisk : Other<br>Verve : Other<br>Novo-Nordisk : Pharmacology and Pharmacotherapy<br>**Total impact on personal income or income to an entity controlled personally**<br>>10,000EUR/year
	**2025**	**Ongoing research funding from public and/or not-for-profit organisations**<br>Amgen<br>AstraZeneca/ medimmune<br>Novartis<br>**Payment from healthcare industry**<br>Astra Zeneca : Other<br>Janssen-Cilag : Other<br>Novo-Nordisk : Other<br>Verve : Other<br>Eli Lilly : Pharmacology and Pharmacotherapy<br>Novo-Nordisk : Pharmacology and Pharmacotherapy<br>**Total impact on personal income or income to an entity controlled personally**<br>>10,000EUR/year
Redfern Julie	**2024**	**Ongoing research funding from public and/or not-for-profit organisations**<br>National Health and Medical Research Council, Australia<br>**Any other interest (financial or otherwise) that should be declared in view of holding an ESC position**<br>Associate Editor, npj Digital Medicine Editorial Board Member, Heart Lung Circulation<br>**Total impact on personal income or income to an entity controlled personally**<br>0 (none) - 10,000EUR/year

Expert		Type of Relationship with Industry
Redfern Julie	**2025**	**Ongoing research funding from public and/or not-for-profit organisations**<br>National Health and Medical Research Council<br>**Membership or affiliation in political or advocacy groups working in the field of cardiovascular medicine/sciences**<br>World Heart Federation, Cardiac Society of Australia and New Zealand, Australian Cardiovascular Alliance<br>**Positions of influence in non-ESC organisations and non-ESC journals with a focus in the field of cardiovascular medicine/sciences**<br>Leadership positions:Board Member, World Heart Federation<br>Positions as Editor in Chief:Editor, npj Digital Medicine Editor, Heart Luncg Circulation<br>**Total impact on personal income or income to an entity controlled personally**<br>0 (none) - 10,000EUR/year
Sabik Joseph	**2024**	**Employment (including part time) in healthcare industry**<br>University Hospitals Cleveland Medical Center : Chair, Department of Surgery<br>**Total impact on personal income or income to an entity controlled personally**<br>0 (none) - 10,000EUR/year
	**2025**	**Payment from healthcare industry**<br>Abbott : Valvular Heart Disease<br>Medtronic : Valvular Heart Disease<br>**Employment (including part time) in healthcare industry**<br>University Hospitals Cleveland Medical Center : Chair, Department of Surgery<br>**Positions of influence in non-ESC organisations and non-ESC journals with a focus in the field of cardiovascular medicine/sciences**<br>Leadership positions:Society of Thoracic Surgeons, President<br>**Total impact on personal income or income to an entity controlled personally**<br>0 (none) - 10,000EUR/year

Expert		Type of Relationship with Industry
Saldarriaga Clara Ines	**2024**	**Payment from healthcare industry**<br>Novo-Nordisk : Acute Coronary Syndromes<br>Astra Zeneca : Chronic Heart Failure<br>Bayer : Chronic Heart Failure<br>Boehringer-Ingelheim : Chronic Heart Failure<br>Eli Lilly : Chronic Heart Failure<br>Novartis : Chronic Heart Failure<br>Pfizer : Chronic Heart Failure<br>Merck Sharp & Dohme : Chronic Heart Failure<br>**Funding from healthcare industry for travel to, and attendance of events and meetings, unrelated to any specific activity**<br>Bayer : Chronic Heart Failure<br>Boehringer-Ingelheim : Chronic Heart Failure<br>**Total impact on personal income or income to an entity controlled personally**<br>0 (none) - 10,000EUR/year
	**2025**	**Payment from healthcare industry**<br>Novo-Nordisk : Acute Coronary Syndromes<br>Astra Zeneca : Chronic Heart Failure<br>Bayer : Chronic Heart Failure<br>Boehringer-Ingelheim : Chronic Heart Failure<br>Eli Lilly : Chronic Heart Failure<br>Novartis : Chronic Heart Failure<br>Pfizer : Chronic Heart Failure<br>Merck Sharp & Dohme : Chronic Heart Failure<br>**Funding from healthcare industry for travel to, and attendance of events and meetings, unrelated to any specific activity**<br>Bayer : Chronic Heart Failure<br>Boehringer-Ingelheim : Chronic Heart Failure<br>**Total impact on personal income or income to an entity controlled personally**<br>0 (none) - 10,000EUR/year
Sarrafzadegan Nizal	**2024**	Nothing to be declared

Expert	Type of Relationship with Industry
Sarrafzadegan Nizal	**2025** Nothing to be declared

Expert		Type of Relationship with Industry
Abbasaliyev Abbasali	**2024**	Nothing to be declared
	**2025**	Nothing to be declared
Abdullaeva Saodat	**2024**	**Payment from healthcare industry**<br>Sharq Clin Trials : Acute Coronary Syndromes<br>**Employment (including part time) in healthcare industry**<br>Republican Specialized Scientific and Practical Center of Cardiology : Cardiologist in Resuscitation<br>**Any other interest (financial or otherwise) that should be declared in view of holding an ESC position**<br>Works as part of the working group for the development of training programs 'Home Rehabilitation' for family doctors and nurses<br>**Total impact on personal income or income to an entity controlled personally**<br>0 (none) - 10,000EUR/year
	**2025**	**Payment from healthcare industry to department or institution or any other body for personal services**<br>GM Pharmaceuticals Ltd. : Coronary Artery Disease (Chronic) / Chronic Coronary Syndromes (CCS)<br>**Employment (including part time) in healthcare industry**<br>Republican Specialized Scientific and Practical Center of Cardiology : Cardiologist in Resuscitation<br>**Total impact on personal income or income to an entity controlled personally**<br>0 (none) - 10,000EUR/year
Adamo Marianna	**2024**	**Payment from healthcare industry**<br>Edwards Lifesciences : Valvular Heart Disease<br>Abbott Vascular : Valvular Heart Disease<br>**Total impact on personal income or income to an entity controlled personally**<br>0 (none) - 10,000EUR/year
	**2025**	**Payment from healthcare industry**<br>Roche Diagnostics : Chronic Heart Failure<br>Abbott : Valvular Heart Disease<br>Edwards Lifesciences : Valvular Heart Disease<br>Medtronic : Valvular Heart Disease<br>**Total impact on personal income or income to an entity controlled personally**<br>0 (none) - 10,000EUR/year
Aktaa Suleman	**2024**	Nothing to be declared
	**2025**	Nothing to be declared

Expert		Type of Relationship with Industry
Andrea Riba Rut	**2024**	**Ongoing research funding from public and/or not-for-profit organisations**<br>Public Funding for Research Fondos de Investigaciones Sanitarias del Instituto de Salud Carlos III (PI23/00846)<br>**Payment from healthcare industry**<br>Daiichi Sankyo : Acute Coronary Syndromes<br>Novartis : Acute Coronary Syndromes<br>**Payment from healthcare industry to department or institution or any other body for personal services**<br>Amgen : Acute Coronary Syndromes<br>Astra Zeneca : Acute Coronary Syndromes<br>Daiichi Sankyo : Acute Coronary Syndromes<br>Novartis : Acute Coronary Syndromes<br>Novo-Nordisk : Acute Coronary Syndromes<br>**Funding from healthcare industry for travel to, and attendance of events and meetings, unrelated to any specific activity**<br>Menarini : Coronary Artery Disease (Chronic) / Chronic Coronary Syndromes (CCS)<br>**Research funding from healthcare industry received by department/institution or an entity controlled by department/institution, under direct/personal responsibility**<br>Ferrer Internacional : Participation in the HERMES Registry, Site investigator<br>**Total impact on personal income or income to an entity controlled personally**<br>0 (none) - 10,000EUR/year
	**2025**	**Ongoing research funding from public and/or not-for-profit organisations**<br>Public Funding for Research Fondos de Investigaciones Sanitarias del Instituto de Salud Carlos III (PI23/00846)<br>**Payment from healthcare industry**<br>Johnson & Johnson : Acute Cardiac Care<br>Daiichi Sankyo : Acute Coronary Syndromes<br>Novartis : Acute Coronary Syndromes<br>**Payment from healthcare industry to department or institution or any other body for personal services**<br>Amgen : Acute Coronary Syndromes<br>Daiichi Sankyo : Acute Coronary Syndromes<br>Novartis : Acute Coronary Syndromes<br>Novo-Nordisk : Acute Coronary Syndromes<br>**Funding from healthcare industry for travel to, and attendance of events and meetings, unrelated to any specific activity**<br>Daiichi Sankyo : Acute Coronary Syndromes

Expert	Type of Relationship with Industry	
Andrea Riba Rut	**2025**	**Positions of influence in non-ESC organisations and non-ESC journals with a focus in the field of cardiovascular medicine/sciences**<br>Leadership positions:Member of the Board of the Ischemich Heart Disease and Acute Cardiac Care Association of the Spanish Society of Cardiology<br>Positions as Editor in Chief:NA<br>Other editorial positions:NA<br>**Total impact on personal income or income to an entity controlled personally**<br>0 (none) - 10,000EUR/year
Angel Kristin	**2024**	Nothing to be declared
	**2025**	Nothing to be declared
Asselbergs Folkert	**2024**	**Research funding from healthcare industry received by department/institution or an entity controlled by department/institution, under direct/personal responsibility**<br>Novartis : Data standardisation quality registry, principal investigator<br>Astra Zeneca : Nudging trial to improve guideline adherence, principal investigator<br>**Total impact on personal income or income to an entity controlled personally**<br>0 (none) - 10,000EUR/year
	**2025**	**Positions of influence in non-ESC organisations and non-ESC journals with a focus in the field of cardiovascular medicine/sciences**<br>Other editorial positions:Editorial board | Current Heart Failure Reports - Springer<br>**Total impact on personal income or income to an entity controlled personally**<br>0 (none) - 10,000EUR/year
Bani Marjeh Mohammed Yassin	**2024**	Nothing to be declared
	**2025**	Nothing to be declared
Batalli Arlind	**2024**	Nothing to be declared
	**2025**	Nothing to be declared
Beissel Jean	**2024**	Nothing to be declared
	**2025**	Nothing to be declared

Expert		Type of Relationship with Industry
Berkinbayev Salim	**2024**	**Employment (including part time) in healthcare industry**<br>Scientific Research Institute of Cardiology : chief consultant<br>**Total impact on personal income or income to an entity controlled personally**<br>0 (none) - 10,000EUR/year
	**2025**	Nothing to be declared
Borger Michael A	**2024**	**Payment from healthcare industry to department or institution or any other body for personal services**<br>Artivion (formerly CryoLife) : Diseases of the Aorta<br>Abbott : Valvular Heart Disease<br>Edwards Lifesciences : Valvular Heart Disease<br>Medtronic : Valvular Heart Disease<br>**Total impact on personal income or income to an entity controlled personally**<br>0 (none) - 10,000EUR/year
	**2025**	**Payment from healthcare industry to department or institution or any other body for personal services**<br>Artivion (formerly CryoLife) : Diseases of the Aorta<br>Abbott : Valvular Heart Disease<br>Edwards Lifesciences : Valvular Heart Disease<br>Medtronic : Valvular Heart Disease<br>**Positions of influence in non-ESC organisations and non-ESC journals with a focus in the field of cardiovascular medicine/sciences**<br>Leadership positions:Councillor-at-Large, EACTS<br>Other editorial positions:Associate Editor, JACC<br>**Total impact on personal income or income to an entity controlled personally**<br>0 (none) - 10,000EUR/year
Boriani Giuseppe	**2024**	**Payment from healthcare industry**<br>Bristol Myers Squibb : Arrhythmias, General<br>Daiichi Sankyo : Atrial Fibrillation (AF)<br>Boston Scientific : Ventricular Arrhythmias and Sudden Cardiac Death (SCD)<br>**Any other interest (financial or otherwise) that should be declared in view of holding an ESC position**<br>Member of the Editorial Board of Acta Cardiologica and European Journal of Internal Medicine<br>**Total impact on personal income or income to an entity controlled personally**<br>0 (none) - 10,000EUR/year

Expert		Type of Relationship with Industry
Boriani Giuseppe	**2025**	**Payment from healthcare industry**<br>Boston Scientific : Device Therapy<br>Lilly : Risk Factors and Prevention<br>Daiichi Sankyo : Stroke<br>**Total impact on personal income or income to an entity controlled personally**<br>0 (none) - 10,000EUR/year
Brida Margarita	**2024**	Nothing to be declared
	**2025**	**Positions of influence in non-ESC organisations and non-ESC journals with a focus in the field of cardiovascular medicine/sciences**<br>Leadership positions:One of the five deputy editors for International Journal of Cardiology Congenital Heart Disease<br>**Total impact on personal income or income to an entity controlled personally**<br>0 (none) - 10,000EUR/year
Bunc Matjaz	**2024**	**Payment from healthcare industry**<br>Meril : Valvular Heart Disease, Interventional Cardiology<br>**Research funding from healthcare industry received by department/institution or an entity controlled by department/institution, under direct/personal responsibility**<br>Meril : Landmark trial, principal invest<br>**Total impact on personal income or income to an entity controlled personally**<br>0 (none) - 10,000EUR/year
	**2025**	Nothing to be declared
Byrne Robert	**2024**	**Research funding from healthcare industry received by department/institution or an entity controlled by department/institution, under direct/personal responsibility**<br>Biosensors : Clinical trial activity, Principal investigator<br>Boston Scientific : Research grant to institution of employment to support a clinical trial, Principal investigator<br>Abbott Vascular : Research/educational grant to institution of employment to support fellowship programme, Clinical director<br>Translumina : Research/educational grant to institution of employment to support research nurse for patient outcomes, Clinical director

Expert		Type of Relationship with Industry
Byrne Robert	**2024**	**Membership or affiliation in political or advocacy groups working in the field of cardiovascular medicine/sciences**<br>Member of Irish Heart Attack Audit Governance Committee Member Health Products Regulatory Authority (HPRA; Irish Competent Authority) Advisory Committee on Medical Devices<br>**Any other interest (financial or otherwise) that should be declared in view of holding an ESC position**<br>Deputy Editor at EuroIntervention Guest Editor at Circulation<br>**Total impact on personal income or income to an entity controlled personally**<br>0 (none) - 10,000EUR/year
	**2025**	**Research funding from healthcare industry received by department/institution or an entity controlled by department/institution, under direct/personal responsibility**<br>Terumo Inc : Clinical trial enrollment, Local PI in clinical trial<br>TELEFLEX : Investigator-initiated clinical trial of stenting plus DCB in patients with diabetes, Coordinating PI<br>Boston Scientific : Investigator-initiated clinical trial on calcium modification algorithm, Coordinating PI<br>Abbott Vascular : Research/educational grant to support research fellowship position, Diretor of hospital department<br>**Positions of influence in non-ESC organisations and non-ESC journals with a focus in the field of cardiovascular medicine/sciences**<br>Leadership positions:Editor-in-chief, PCR textbook of interventional cardiology<br>**Total impact on personal income or income to an entity controlled personally**<br>0 (none) - 10,000EUR/year
Chettibi Mohamed	**2024**	**Payment from healthcare industry**<br>Novo-Nordisk : Coronary Artery Disease (Chronic) / Chronic Coronary Syndromes (CCS) , Risk Factors and Prevention<br>**Total impact on personal income or income to an entity controlled personally**<br>0 (none) - 10,000EUR/year
	**2025**	**Payment from healthcare industry**<br>Boehringer-Ingelheim : Clinical Skills<br>Abbott : Device Therapy<br>Amgen : Risk Factors and Prevention<br>Novo-Nordisk : Risk Factors and Prevention<br>**Total impact on personal income or income to an entity controlled personally**<br>0 (none) - 10,000EUR/year

Expert		Type of Relationship with Industry
Delle-Karth Georg	2024	**Ongoing research funding from public and/or not-for-profit organisations**<br>Sigmund Freud University Vienna<br>**Payment from healthcare industry**<br>Biotronik : Coronary Artery Disease (Chronic) / Chronic Coronary Syndromes (CCS)<br>Amarin : Coronary Artery Disease (Chronic) / Chronic Coronary Syndromes (CCS)<br>Edwards Lifesciences : Valvular Heart Disease<br>Medtronic : Valvular Heart Disease<br>**Payment from healthcare industry to department or institution or any other body for personal services**<br>Edwards Lifesciences : Valvular Heart Disease<br>**Funding from healthcare industry for travel to, and attendance of events and meetings, unrelated to any specific activity**<br>Abiomed : Acute Coronary Syndromes<br>**Total impact on personal income or income to an entity controlled personally**<br>>10,000EUR/year
	2025	**Payment from healthcare industry**<br>Abiomed : Acute Heart Failure<br>Boehringer-Ingelheim : Chronic Heart Failure<br>Edwards Lifesciences : Interventional Cardiology<br>**Payment from healthcare industry to department or institution or any other body for personal services**<br>Edwards Lifesciences : Valvular Heart Disease<br>**Total impact on personal income or income to an entity controlled personally**<br>0 (none) - 10,000EUR/year
Diletti Roberto	2024	**Payment from healthcare industry**<br>Boston Scientific : Interventional Cardiology<br>Abbott Vascular : Interventional Cardiology<br>**Total impact on personal income or income to an entity controlled personally**<br>0 (none) - 10,000EUR/year
	2025	**Payment from healthcare industry**<br>Abbott : Coronary Artery Disease (Chronic) / Chronic Coronary Syndromes (CCS)<br>Boston Scientific : Coronary Artery Disease (Chronic) / Chronic Coronary Syndromes (CCS)<br>**Total impact on personal income or income to an entity controlled personally**<br>0 (none) - 10,000EUR/year

Expert	Type of Relationship with Industry	
Dobiliene Olivija	**2024**	**Payment from healthcare industry**<br>Servier : Coronary Artery Disease (Chronic) / Chronic Coronary Syndromes (CCS)<br>Berlin Chemie AG : Coronary Artery Disease (Chronic) / Chronic Coronary Syndromes (CCS)<br>Novartis : Risk Factors and Prevention<br>Viatris : Risk Factors and Prevention<br>Parexel : Risk Factors and Prevention<br>**Funding from healthcare industry for travel to, and attendance of events and meetings, unrelated to any specific activity**<br>Novartis : Risk Factors and Prevention<br>**Total impact on personal income or income to an entity controlled personally**<br>>10,000EUR/year
	**2025**	**Payment from healthcare industry**<br>Astra Zeneca : Chronic Heart Failure<br>Boehringer-Ingelheim : Chronic Heart Failure<br>Servier : Coronary Artery Disease (Chronic) / Chronic Coronary Syndromes (CCS)<br>Amgen : Risk Factors and Prevention<br>Novartis : Risk Factors and Prevention<br>Novo-Nordisk : Risk Factors and Prevention<br>Viatris : Risk Factors and Prevention<br>Parexel : Risk Factors and Prevention<br>**Total impact on personal income or income to an entity controlled personally**<br>>10,000EUR/year
Durak Nalbantic Azra	**2024**	**Payment from healthcare industry**<br>Bayer : Chronic Heart Failure<br>Novo-Nordisk : Chronic Heart Failure<br>Novartis : Pharmacology and Pharmacotherapy<br>**Funding from healthcare industry for travel to, and attendance of events and meetings, unrelated to any specific activity**<br>Roche Diagnostics : Cardiovascular Disease in Special Populations<br>**Total impact on personal income or income to an entity controlled personally**<br>0 (none) - 10,000EUR/year

Expert		Type of Relationship with Industry
Durak Nalbantic Azra	**2025**	**Payment from healthcare industry**<br>Bayer : Chronic Heart Failure<br>Novo-Nordisk : Chronic Heart Failure<br>Krka Pharma : Hypertension<br>Novartis : Pharmacology and Pharmacotherapy<br>**Funding from healthcare industry for travel to, and attendance of events and meetings, unrelated to any specific activity**<br>Berlin Chemie AG : Atrial Fibrillation (AF)<br>Krka Pharma : Training and Education<br>**Total impact on personal income or income to an entity controlled personally**<br>0 (none) - 10,000EUR/year
Edes Istvan Ferenc	**2024**	**Payment from healthcare industry**<br>Boston Scientific : Interventional Cardiology<br>Biotronik : Interventional Cardiology<br>**Total impact on personal income or income to an entity controlled personally**<br>0 (none) - 10,000EUR/year
	**2025**	**Payment from healthcare industry**<br>Boston Scientific : Interventional Cardiology<br>Biotronik : Interventional Cardiology<br>Abbott Vascular : Interventional Cardiology<br>Argon Medical Devices : Interventional Cardiology<br>**Total impact on personal income or income to an entity controlled personally**<br>0 (none) - 10,000EUR/year

Expert	Type of Relationship with Industry	
Gandjbakhch Estelle	**2024**	**Payment from healthcare industry**<br>Boston Scientific : Device Therapy<br>Medtronic : Device Therapy<br>Biotronik : Device Therapy<br>Zoll Medical : Device Therapy<br>Microport : Device Therapy<br>Abbott : Interventional Cardiology<br>Tenaya : Myocardial Disease<br>**Research funding from healthcare industry received by department/institution or an entity controlled by department/institution, under direct/personal responsibility**<br>Biotronik : research grant for academic research on inherited arrhythmia and catheter ablation, investigator<br>Boston Scientific : research grant for academic research on inherited arrhythmia and catheter ablation, principal investigator<br>Medtronic : research grant for academic research on inherited arrhythmia and catheter ablation, principal investigator<br>Microport : research grant for academic research on inherited arrhythmia and catheter ablation, principal investigator<br>**Total impact on personal income or income to an entity controlled personally**<br>0 (none) - 10,000EUR/year
	**2025**	**Payment from healthcare industry**<br>Boston Scientific : Device Therapy<br>Medtronic : Device Therapy<br>Microport : Device Therapy<br>**Research funding from healthcare industry received by department/institution or an entity controlled by department/institution, under direct/personal responsibility**<br>Biotronik : research grant for academic research on inherited arrhythmia and catheter ablation, investigator<br>Boston Scientific : research grant for academic research on inherited arrhythmia and catheter ablation, principal investigator<br>Medtronic : research grant for academic research on inherited arrhythmia and catheter ablation, principal investigator<br>Microport : research grant for academic research on inherited arrhythmia and catheter ablation, principal investigator<br>**Positions of influence in non-ESC organisations and non-ESC journals with a focus in the field of cardiovascular medicine/sciences**<br>Leadership positions:Vice -president of the French Rhythm society<br>**Total impact on personal income or income to an entity controlled personally**<br>0 (none) - 10,000EUR/year

Expert		Type of Relationship with Industry
Gerber Bernhard Lothar Marie	**2024**	**Payment from healthcare industry**<br>Bristol Myers Squibb : Myocardial Disease<br>Novartis : Risk Factors and Prevention<br>**Membership or affiliation in political or advocacy groups working in the field of cardiovascular medicine/sciences**<br>Belgian Alliance for Cardiovacular Health (BACH)<br>**Total impact on personal income or income to an entity controlled personally**<br>0 (none) - 10,000EUR/year
	**2025**	**Payment from healthcare industry**<br>Novartis : Coronary Artery Disease (Chronic) / Chronic Coronary Syndromes (CCS)<br>Cytokinetics : Myocardial Disease<br>**Total impact on personal income or income to an entity controlled personally**<br>0 (none) - 10,000EUR/year
Grosu Aurel	**2024**	Nothing to be declared
	**2025**	Nothing to be declared
Hartikainen Tau	**2024**	**Payment from healthcare industry**<br>Astra Zeneca : Interventional Cardiology<br>Amgen : Risk Factors and Prevention<br>**Funding from healthcare industry for travel to, and attendance of events and meetings, unrelated to any specific activity**<br>Philips : Interventional Cardiology<br>Novartis : Training and Education<br>Boston Scientific : Valvular Heart Disease<br>**Total impact on personal income or income to an entity controlled personally**<br>0 (none) - 10,000EUR/year
	**2025**	**Payment from healthcare industry**<br>Astra Zeneca : Interventional Cardiology<br>**Funding from healthcare industry for travel to, and attendance of events and meetings, unrelated to any specific activity**<br>Novartis : Training and Education<br>Boston Scientific : Valvular Heart Disease

Expert		Type of Relationship with Industry
Hartikainen Tau	**2025**	**Any other interest (financial or otherwise) that should be declared in view of holding an ESC position**<br>Grant from the EAPCI (sponsored by Edwards Lifesciences) for a clinical fellowship (25'000€).<br>**Total impact on personal income or income to an entity controlled personally**<br>0 (none) - 10,000EUR/year
Hasdai David	**2024**	Nothing to be declared
	**2025**	Nothing to be declared
Hayrapetyan Hamlet	**2024**	**Payment from healthcare industry**<br>Servier : Hypertension<br>Egis Pharma : Risk Factors and Prevention<br>Krka Pharma : Risk Factors and Prevention<br>**Research funding from healthcare industry received by department/institution or an entity controlled by department/institution, under direct/personal responsibility**<br>Acino : Multicenter International Study, Principal Investigator<br>Servier : RCT, Principal investigator<br>**Total impact on personal income or income to an entity controlled personally**<br>0 (none) - 10,000EUR/year
	**2025**	**Payment from healthcare industry**<br>Servier : Hypertension<br>Meril : Interventional Cardiology<br>Tectonic Therapeutic : Pulmonary Circulation, Pulmonary Embolism, Right Heart Failure<br>Egis Pharma : Risk Factors and Prevention<br>Gedeon Richter : Risk Factors and Prevention<br>**Total impact on personal income or income to an entity controlled personally**<br>>10,000EUR/year

Expert		Type of Relationship with Industry
Heidecker Bettina	**2024**	**Ongoing research funding from public and/or not-for-profit organisations**<br>Pfizer<br>**Payment from healthcare industry**<br>Pfizer, Bayer, Sanofi-Aventis, Novartis, Astra Zeneca : Clinical Skills , Chronic Heart Failure , Acute Heart Failure , Myocardial Disease , Pericardial Disease<br>**Research funding from healthcare industry received by department/institution or an entity controlled by department/institution, under direct/personal responsibility**<br>Pfizer : Analysis of biomarkers in blood of patients with amyloidosis; assess risk for adverse cardiovascular events, Principal investigator<br>**Total impact on personal income or income to an entity controlled personally**<br>0 (none) - 10,000EUR/year
	**2025**	**Payment from healthcare industry**<br>Astra Zenca, Bayer, Sandbox AQ, BMS : Clinical Skills, Chronic Heart Failure, Acute Heart Failure, Myocardial Disease, Pericardial Disease<br>**Total impact on personal income or income to an entity controlled personally**<br>0 (none) - 10,000EUR/year
Hennemuth Anja	**2024**	**Ongoing research funding from public and/or not-for-profit organisations**<br>BMBF<br>Deutsche Forschungsgemeinschaft<br>**Total impact on personal income or income to an entity controlled personally**<br>0 (none) - 10,000EUR/year
	**2025**	**Ongoing research funding from public and/or not-for-profit organisations**<br>BMFTR<br>Deutsche Forschungsgemeinschaft<br>**Total impact on personal income or income to an entity controlled personally**<br>0 (none) - 10,000EUR/year
Hildick-Smith David	**2024**	**Payment from healthcare industry**<br>Abbott Laboratories : Stroke<br>Boston Scientific : Valvular Heart Disease<br>Medtronic : Valvular Heart Disease<br>**Total impact on personal income or income to an entity controlled personally**<br>0 (none) - 10,000EUR/year

Expert		Type of Relationship with Industry
Hildick-Smith David	**2025**	**Payment from healthcare industry**<br>Abbott Laboratories : Stroke<br>Boston Scientific : Valvular Heart Disease<br>Medtronic : Valvular Heart Disease<br>**Total impact on personal income or income to an entity controlled personally**<br>0 (none) - 10,000EUR/year
Hirlekar Geir	**2024**	Nothing to be declared
	**2025**	**Positions of influence in non-ESC organisations and non-ESC journals with a focus in the field of cardiovascular medicine/sciences**<br>Other editorial positions:Icelandic medical journal<br>**Total impact on personal income or income to an entity controlled personally**<br>0 (none) - 10,000EUR/year
Iglesias Juan F	**2024**	**Payment from healthcare industry to department or institution or any other body for personal services**<br>Medtronic : Interventional Cardiology<br>Biotronik : Interventional Cardiology<br>Cordis : Interventional Cardiology<br>Recor Medical : Interventional Cardiology<br>Penumbra : Interventional Cardiology<br>**Funding from healthcare industry for travel to, and attendance of events and meetings, unrelated to any specific activity**<br>Medtronic : Interventional Cardiology<br>**Research funding from healthcare industry received by department/institution or an entity controlled by department/institution, under direct/personal responsibility**<br>Biotronik : Unrestricted research grant to the institution for an investigator-initiated randomized clinical trial, Principal investigator<br>Cathworks : Unrestricted research grant to the institution for an investigator-initiated randomized clinical trial, Principal investigator<br>concept medical : Unrestricted research grant to the institution for an investigator-initiated randomized clinical trial, Principal investigator<br>SMT : Unrestricted research grant to the institution for an investigator-initiated randomized clinical trial, Principal Investigator<br>Terumo Corp. : Unrestricted research grant to the institution for an investigator-initiated randomized clinical trial, Principal investigator

Expert		Type of Relationship with Industry
Iglesias Juan F	**2024**	**Any other interest (financial or otherwise) that should be declared in view of holding an ESC position**<br>American College of Cardiology (fellow) European Cardiovascular Research Institute (ECRI) (Scientific Advisory Board member) Swiss Working Group Interventional Cardiology (Board member) Acta Cardiologica (Editorial Board Member)<br>**Total impact on personal income or income to an entity controlled personally**<br>0 (none) - 10,000EUR/year
	**2025**	**Payment from healthcare industry to department or institution or any other body for personal services**<br>Biotronik : Interventional Cardiology<br>Cordis : Interventional Cardiology<br>Penumbra : Interventional Cardiology<br>**Research funding from healthcare industry received by department/institution or an entity controlled by department/institution, under direct/personal responsibility**<br>Biotronik : Unrestricted research grant to the institution for an investigator-initiated randomized clinical trial, Principal investigator<br>Cathworks : Unrestricted research grant to the institution for an investigator-initiated randomized clinical trial, Principal investigator<br>concept medical : Unrestricted research grant to the institution for an investigator-initiated randomized clinical trial, Principal investigator<br>SMT : Unrestricted research grant to the institution for an investigator-initiated randomized clinical trial, Principal Investigator<br>Terumo Corp. : Unrestricted research grant to the institution for an investigator-initiated randomized clinical trial, Principal investigator<br>**Total impact on personal income or income to an entity controlled personally**<br>0 (none) - 10,000EUR/year
James Stefan	**2024**	**Ongoing research funding from public and/or not-for-profit organisations**<br>Swedish research council<br>**Payment from healthcare industry**<br>Edwards Lifesciences : Valvular Heart Disease<br>Medtronic : Valvular Heart Disease<br>**Research funding from healthcare industry received by department/institution or an entity controlled by department/institution, under direct/personal responsibility**<br>Amgen Inc : Clinical trial, Executive committee<br>Merck Sharp & Dohme : Clinical trial, Executive committee<br>New Amsterdam : DSMB committee. Lipid management , DSMB committee<br>Janssen-Cilag : Executive committee, Executive committee

Expert		Type of Relationship with Industry
James Stefan	**2024**	**Membership or affiliation in political or advocacy groups working in the field of cardiovascular medicine/sciences**<br>Board member of the Swedish research council<br>**Total impact on personal income or income to an entity controlled personally**<br>0 (none) - 10,000EUR/year
	**2025**	**Ongoing research funding from public and/or not-for-profit organisations**<br>Swedish research council<br>**Payment from healthcare industry**<br>Edwards Lifesciences : Valvular Heart Disease<br>Medtronic : Valvular Heart Disease<br>**Membership or affiliation in political or advocacy groups working in the field of cardiovascular medicine/sciences**<br>Board member of the Swedish research council<br>**Positions of influence in non-ESC organisations and non-ESC journals with a focus in the field of cardiovascular medicine/sciences**<br>Leadership positions:Associate editor for Circulation until June 26<br>**Total impact on personal income or income to an entity controlled personally**<br>0 (none) - 10,000EUR/year
Jegere Sanda	**2024**	**Payment from healthcare industry**<br>Bayer : Atrial Fibrillation (AF), Cardiovascular Disease in Special Populations<br>Boehringer Ingelheim : Atrial Fibrillation (AF), Chronic Heart Failure<br>Krka Pharma : Risk Factors and Prevention<br>**Total impact on personal income or income to an entity controlled personally**<br>0 (none) - 10,000EUR/year
	**2025**	**Payment from healthcare industry**<br>Bayer : Atrial Fibrillation (AF), Cardiovascular Disease in Special Populations<br>Boehringer Ingelheim : Atrial Fibrillation (AF), Chronic Heart Failure<br>UAB Baltics Medical : Interventional Cardiology<br>Krka Pharma : Risk Factors and Prevention<br>**Total impact on personal income or income to an entity controlled personally**<br>0 (none) - 10,000EUR/year
Kadyrov Bahram	**2024**	Nothing to be declared
	**2025**	Nothing to be declared

Expert	Type of Relationship with Industry	
Kereselidze Zviad	**2024**	**Membership or affiliation in political or advocacy groups working in the field of cardiovascular medicine/sciences**<br>Head of working group in Interventional Cardiology of Georgian Society of Cardiology President of Georgian Association of Interventional Cardiologists<br>**Any other interest (financial or otherwise) that should be declared in view of holding an ESC position**<br>Head of working group of Interventional Cardiology in ESC represent Georgian Society of Cardiology President of Georgian Association of Interventional Cardiologists<br>**Total impact on personal income or income to an entity controlled personally**<br>0 (none) - 10,000EUR/year
	**2025**	**Membership or affiliation in political or advocacy groups working in the field of cardiovascular medicine/sciences**<br>Head of working group in Interventional Cardiology of Georgian Society of Cardiology President of Georgian Association of Interventional Cardiologists<br>**Total impact on personal income or income to an entity controlled personally**<br>0 (none) - 10,000EUR/year
Landmesser Ulf	**2024**	**Ongoing research funding from public and/or not-for-profit organisations**<br>German Center of Cardiovascular Research (DZHK)<br>Leducq Foundation - Network of excellence in cardiovascular research<br>**Payment from healthcare industry**<br>Menarini : Coronary Artery Disease (Chronic) / Chronic Coronary Syndromes (CCS)<br>Amgen : Risk Factors and Prevention<br>Novartis : Risk Factors and Prevention<br>Sanofi Aventis : Risk Factors and Prevention<br>**Research funding from healthcare industry received by department/institution or an entity controlled by department/institution, under direct/personal responsibility**<br>Abbott : Clinical Study, Principal Investigator<br>Merck Sharp & Dohme : Clinical Study, Steering Committee<br>Novartis : Support of clinical study to institution, Principal investigator<br>**Total impact on personal income or income to an entity controlled personally**<br>0 (none) - 10,000EUR/year

Expert	Type of Relationship with Industry	
Landmesser Ulf	**2025**	**Ongoing research funding from public and/or not-for-profit organisations**<br>German Center of Cardiovascular Research (DZHK)<br>**Payment from healthcare industry**<br>Boehringer Ingelheim : Chronic Heart Failure<br>Amgen : Coronary Artery Disease (Chronic) / Chronic Coronary Syndromes (CCS)<br>Novartis : Coronary Artery Disease (Chronic) / Chronic Coronary Syndromes (CCS)<br>Sanofi Aventis : Coronary Artery Disease (Chronic) / Chronic Coronary Syndromes (CCS)<br>**Research funding from healthcare industry received by department/institution or an entity controlled by department/institution, under direct/personal responsibility**<br>Merck Sharp Dohme : Clinical study, Investigator<br>Novartis : Research Funding received by Institution, Principal Investigator<br>Abbott : Research support for studies in Cath lab, Investigator<br>**Total impact on personal income or income to an entity controlled personally**<br>0 (none) - 10,000EUR/year
Lip Gregory Yh	**2024**	**Payment from healthcare industry to department or institution or any other body for personal services**<br>Pfizer : Atrial Fibrillation (AF)<br>Bristol Myers Squibb : Atrial Fibrillation (AF)<br>**Funding from healthcare industry for travel to, and attendance of events and meetings, unrelated to any specific activity**<br>Daiichi Sankyo : Atrial Fibrillation (AF)<br>**Research funding from healthcare industry received by department/institution or an entity controlled by department/institution, under direct/personal responsibility**<br>Bristol Myers Squibb : AF ‘real world’ registry work. Academic, non promotional research work on registries , project supervision<br>Boehringer-Ingelheim : AF registries [unrestricted educational grant]. Academic, non promotional research work on registries by fellows., project supervision<br>Anthos : AF registries [unrestricted educational grant]. Academic, non promotional research work on registries by fellows., project supervision<br>**Any other interest (financial or otherwise) that should be declared in view of holding an ESC position**<br>Editorial roles in a number of academic journals. Shares in private limited company (a legal separate entity in UK), but no salary/income/personal renumeration received.<br>**Total impact on personal income or income to an entity controlled personally**<br>0 (none) - 10,000EUR/year

Expert		Type of Relationship with Industry
Lip Gregory Yh	**2025**	**Payment from healthcare industry to department or institution or any other body for personal services**<br>Pfizer : Atrial Fibrillation (AF)<br>Bristol Myers Squibb : Atrial Fibrillation (AF)<br>**Research funding from healthcare industry received by department/institution or an entity controlled by department/institution, under direct/personal responsibility**<br>Bristol Myers Squibb : AF ‘real world’ registry and outcomes research work. Academic, non promotional research work on 'big data' registries , project supervision<br>Anthos : AF registries [unrestricted educational grant]. Academic, non promotional research work on registries by fellows., project supervision<br>**Positions of influence in non-ESC organisations and non-ESC journals with a focus in the field of cardiovascular medicine/sciences**<br>Positions as Editor in Chief:Thrombosis & Haemostasis (clinical co-Editor in Chief)<br>Other editorial positions:Guest Editor for Circulation. Misc. editorial roles (section editor or editorial board member) for various academic journals, eg JACC Asia, Heart, Expert Reviews in Cardiovascular Therapy, Journal of Clinical Medicine, etc<br>**Any other interest (financial or otherwise) that should be declared in view of holding an ESC position**<br>Shares in private limited company (a legal separate entity in UK), but no salary/income/personal renumeration received.<br>**Total impact on personal income or income to an entity controlled personally**<br>0 (none) - 10,000EUR/year
Lunegova Olga	**2024**	Nothing to be declared
	**2025**	Nothing to be declared
Marandi Toomas	**2024**	**Payment from healthcare industry**<br>Novartis Baltics : Acute Coronary Syndromes<br>**Total impact on personal income or income to an entity controlled personally**<br>0 (none) - 10,000EUR/year
	**2025**	**Payment from healthcare industry**<br>Amgen : Training and Education<br>Boehringer-Ingelheim : Training and Education<br>Novartis : Training and Education<br>**Total impact on personal income or income to an entity controlled personally**<br>0 (none) - 10,000EUR/year
Mcevoy John William	**2024**	Nothing to be declared

Expert		Type of Relationship with Industry
Mcevoy John William	**2025**	Nothing to be declared
Mercieca Balbi Matthew	**2024**	Nothing to be declared
	**2025**	Nothing to be declared
Mihaylova Borislava	**2024**	**Ongoing research funding from public and/or not-for-profit organisations**<br>Barts Charity, UK<br>Bowel Research UK<br>Cancer Research UK<br>National Institute for Health and Care Research, UK<br>**Total impact on personal income or income to an entity controlled personally**<br>0 (none) - 10,000EUR/year
	**2025**	Nothing to be declared
Moelgaard Inge	**2024**	Nothing to be declared
	**2025**	Nothing to be declared
Mokarrab Mostafa Ibrahim	**2024**	Nothing to be declared
	**2025**	Nothing to be declared
Myrianthopoulos Kimon	**2024**	Nothing to be declared
	**2025**	Nothing to be declared
Neubeck Lis	**2024**	**Ongoing research funding from public and/or not-for-profit organisations**<br>Burdett Trust for Nursing<br>Chief Scientists Office<br>**Any other interest (financial or otherwise) that should be declared in view of holding an ESC position**<br>Consultant Editor British Journal of Cardiac Nursing<br>**Total impact on personal income or income to an entity controlled personally**<br>0 (none) - 10,000EUR/year
	**2025**	**Ongoing research funding from public and/or not-for-profit organisations**<br>Burdett Trust for Nursing<br>Chief Scientists Office

Expert		Type of Relationship with Industry
Neubeck Lis	**2025**	**Any other interest (financial or otherwise) that should be declared in view of holding an ESC position**<br>Consultant Editor British Journal of Cardiac Nursing<br>**Total impact on personal income or income to an entity controlled personally**<br>0 (none) - 10,000EUR/year
Okuyan Ertugrul	**2024**	Nothing to be declared
	**2025**	Nothing to be declared
O'neill Jim	**2024**	**Funding from healthcare industry for travel to, and attendance of events and meetings, unrelated to any specific activity**<br>Pfizer : Chronic Heart Failure<br>**Research funding from healthcare industry received personally**<br>Pfizer<br>**Research funding from healthcare industry received by department/institution or an entity controlled by department/institution, under direct/personal responsibility**<br>Pfizer : Heart Failure Preserved Ejection Fraction, PI, supervisor of student<br>**Total impact on personal income or income to an entity controlled personally**<br>>10,000EUR/year
	**2025**	**Payment from healthcare industry**<br>Novartis : History of Cardiology<br>**Funding from healthcare industry for travel to, and attendance of events and meetings, unrelated to any specific activity**<br>Pfizer : Chronic Heart Failure<br>**Research funding from healthcare industry received by department/institution or an entity controlled by department/institution, under direct/personal responsibility**<br>Pfizer : Heart Failure Preserved Ejection Fraction, PI, supervisor of student<br>**Total impact on personal income or income to an entity controlled personally**<br>0 (none) - 10,000EUR/year
Ostadal Petr	**2024**	**Payment from healthcare industry**<br>Amgen : Acute Coronary Syndromes<br>Novartis : Acute Coronary Syndromes<br>Sanofi Aventis : Acute Coronary Syndromes<br>Zentiva : Acute Coronary Syndromes<br>Boehringer-Ingelheim : Acute Heart Failure<br>Abiomed : Acute Heart Failure<br>Xenios/Fresenius : Acute Heart Failure

Expert		Type of Relationship with Industry
Ostadal Petr	2024	**Total impact on personal income or income to an entity controlled personally**<br>0 (none) - 10,000EUR/year
	2025	**Payment from healthcare industry**<br>Johnson & Johnson : Acute Cardiac Care<br>Fresenius Nutrition : Acute Cardiac Care<br>Herbacos : Acute Cardiac Care<br>Amgen : Acute Coronary Syndromes<br>Novartis : Acute Coronary Syndromes<br>Sanofi Aventis : Acute Coronary Syndromes<br>Boehringer-Ingelheim : Acute Heart Failure<br>Astra Zeneca : Chronic Heart Failure<br>**Positions of influence in non-ESC organisations and non-ESC journals with a focus in the field of cardiovascular medicine/sciences**<br>Leadership positions:President, Czech Society of Cardiology<br>**Total impact on personal income or income to an entity controlled personally**<br>0 (none) - 10,000EUR/year
Parkhomenko Alexander	2024	**Payment from healthcare industry**<br>Pfizer : Atrial Fibrillation (AF), Pulmonary Circulation, Pulmonary Embolism, Right Heart Failure<br>Boehringer-Ingelheim : Chronic Heart Failure<br>Astra Zeneca : Chronic Heart Failure, Acute Coronary Syndromes<br>Novartis : Chronic Heart Failure, Acute Heart Failure<br>Amgen : Coronary Artery Disease (Chronic) / Chronic Coronary Syndromes (CCS)<br>Novo-Nordisk : Risk Factors and Prevention<br>**Research funding from healthcare industry received by department/institution or an entity controlled by department/institution, under direct/personal responsibility**<br>Boehringer-Ingelheim : Testing a new medication for acute myocardial infaction, principal investigator<br>**Total impact on personal income or income to an entity controlled personally**<br>>10,000EUR/year

Expert		Type of Relationship with Industry
Parkhomenko Alexander	**2025**	**Payment from healthcare industry**<br>Pfizer : Atrial Fibrillation (AF), Pulmonary Circulation, Pulmonary Embolism, Right Heart Failure<br>Boehringer-Ingelheim : Chronic Heart Failure<br>Astra Zeneca : Chronic Heart Failure, Acute Coronary Syndromes<br>Novartis : Chronic Heart Failure, Acute Heart Failure<br>Amgen : Coronary Artery Disease (Chronic) / Chronic Coronary Syndromes (CCS)<br>Novo-Nordisk : Risk Factors and Prevention<br>**Research funding from healthcare industry received by department/institution or an entity controlled by department/institution, under direct/personal responsibility**<br>Amgen : Testing a new medication for MACE prevention in CAD patients , principal investigator<br>**Total impact on personal income or income to an entity controlled personally**<br>>10,000EUR/year
Patti Giuseppe	**2024**	**Ongoing research funding from public and/or not-for-profit organisations**<br>Novo Nordisk<br>**Total impact on personal income or income to an entity controlled personally**<br>0 (none) - 10,000EUR/year
	**2025**	**Ongoing research funding from public and/or not-for-profit organisations**<br>Novo Nordisk<br>**Total impact on personal income or income to an entity controlled personally**<br>0 (none) - 10,000EUR/year
Petkoska Danica	**2024**	Nothing to be declared
	**2025**	Nothing to be declared
Porela Pekka	**2024**	Nothing to be declared
	**2025**	Nothing to be declared
Prescott Eva	**2024**	Nothing to be declared
	**2025**	Nothing to be declared

Expert		Type of Relationship with Industry
Radu Razvan Ilie	**2024**	**Payment from healthcare industry**<br>Chiesi Pharma : Acute Coronary Syndromes<br>Astra Zeneca : Chronic Heart Failure<br>Boehringer-Ingelheim : Chronic Heart Failure<br>**Funding from healthcare industry for travel to, and attendance of events and meetings, unrelated to any specific activity**<br>Boehringer-Ingelheim : Chronic Heart Failure<br>**Total impact on personal income or income to an entity controlled personally**<br>0 (none) - 10,000EUR/year
	**2025**	**Payment from healthcare industry**<br>Boehringer-Ingelheim : History of Cardiology, Chronic Heart Failure<br>**Funding from healthcare industry for travel to, and attendance of events and meetings, unrelated to any specific activity**<br>Boehringer-Ingelheim : Chronic Heart Failure<br>**Total impact on personal income or income to an entity controlled personally**<br>0 (none) - 10,000EUR/year
Rocca Bianca	**2024**	**Ongoing research funding from public and/or not-for-profit organisations**<br>Cancer UK<br>**Payment from healthcare industry**<br>Bayer AG : Pharmacology and Pharmacotherapy<br>**Research funding from healthcare industry received by department/institution or an entity controlled by department/institution, under direct/personal responsibility**<br>Bios Therapy : Substance Based medical devices in obesity, site investigator<br>**Membership or affiliation in political or advocacy groups working in the field of cardiovascular medicine/sciences**<br>ISTH, Italian Society of Thrombosis and Haemostasis (SISET), italian society of Pharmacology (SIF), Mediterranean League against Thrombosis<br>**Total impact on personal income or income to an entity controlled personally**<br>0 (none) - 10,000EUR/year
	**2025**	**Ongoing research funding from public and/or not-for-profit organisations**<br>Cancer UK<br>**Payment from healthcare industry**<br>Chiesi Pharma : Acute Coronary Syndromes<br>**Research funding from healthcare industry received by department/institution or an entity controlled by department/institution, under direct/personal responsibility**<br>Aboca SRL : natural substance based medical devices in obesity, site investigator

Expert		Type of Relationship with Industry
Rocca Bianca	**2025**	**Positions of influence in non-ESC organisations and non-ESC journals with a focus in the field of cardiovascular medicine/sciences**<br>Other editorial positions:Editorial Board International Journal of Cardiology Editorial Board European Cardiology Review<br>**Total impact on personal income or income to an entity controlled personally**<br>0 (none) - 10,000EUR/year
Rossello Francisco Javier	**2024**	**Any other interest (financial or otherwise) that should be declared in view of holding an ESC position**<br>Associate editor of Heart - https://heart.bmj.com/pages/editorial-board Associate Statistical Editor of Circulation: Cardiovascular Quality and Outcomes - https://www.ahajournals.org/circoutcomes/editorial-board<br>**Total impact on personal income or income to an entity controlled personally**<br>0 (none) - 10,000EUR/year
	**2025**	**Payment from healthcare industry**<br>Boehringer-Ingelheim : Chronic Heart Failure<br>**Total impact on personal income or income to an entity controlled personally**<br>0 (none) - 10,000EUR/year
Sannino Anna	**2024**	Nothing to be declared
	**2025**	**Payment from healthcare industry**<br>Venus Medtech : Valvular Heart Disease<br>**Total impact on personal income or income to an entity controlled personally**<br>0 (none) - 10,000EUR/year
Serhal Ahmad	**2024**	Nothing to be declared
	**2025**	Nothing to be declared
Shuka Naltin	**2024**	Nothing to be declared
	**2025**	Nothing to be declared

Expert	Type of Relationship with Industry	
Skoric Bosko	**2024**	**Payment from healthcare industry**<br>Salveo : Acute Coronary Syndromes<br>Berlin Chemie AG : Atrial Fibrillation (AF)<br>Menarini : Chronic Heart Failure<br>Boehringer-Ingelheim : Chronic Heart Failure, Acute Heart Failure<br>Teva Pharmaceutical Industries : Chronic Heart Failure, Acute Heart Failure<br>Novo-Nordisk : Coronary Artery Disease (Chronic) / Chronic Coronary Syndromes (CCS)<br>Takeda Pharmaceuticals : Other<br>**Payment from healthcare industry to department or institution or any other body for personal services**<br>Bayer : Atrial Fibrillation (AF)<br>Astra Zeneca : Chronic Heart Failure<br>Teva Pharmaceutical Industries : Chronic Heart Failure, Acute Heart Failure, Other<br>Astellas : Other<br>Zentiva : Other<br>Medis Adria : Other<br>**Funding from healthcare industry for travel to, and attendance of events and meetings, unrelated to any specific activity**<br>Boehringer-Ingelheim : Atrial Fibrillation (AF)<br>**Research funding from healthcare industry received personally**<br>Medis Adria<br>**Total impact on personal income or income to an entity controlled personally**<br>0 (none) - 10,000EUR/year

Expert	Type of Relationship with Industry	
Skoric Bosko	**2025**	**Payment from healthcare industry**<br>Boehringer-Ingelheim : Chronic Heart Failure<br>Viatris : Chronic Heart Failure<br>Takeda Pharmaceuticals : Other<br>Zentiva : Other<br>Biotest : Other<br>Berlin Chemie AG : Syncope and Bradycardia, Coronary Artery Disease (Chronic) / Chronic Coronary Syndromes (CCS)<br>**Funding from healthcare industry for travel to, and attendance of events and meetings, unrelated to any specific activity**<br>Berlin Chemie AG : Coronary Artery Disease (Chronic) / Chronic Coronary Syndromes (CCS)<br>Zentiva : Other<br>Biotest : Other<br>**Research funding from healthcare industry received by department/institution or an entity controlled by department/institution, under direct/personal responsibility**<br>Astra Zeneca : The role of basophils in heart transplant rejection, principal investigator<br>**Total impact on personal income or income to an entity controlled personally**<br>0 (none) - 10,000EUR/year
Smer Aiman	**2024**	Nothing to be declared
	**2025**	Nothing to be declared
Studencan Martin	**2024**	**Payment from healthcare industry to department or institution or any other body for personal services**<br>Novartis : Risk Factors and Prevention<br>**Total impact on personal income or income to an entity controlled personally**<br>0 (none) - 10,000EUR/year
	**2025**	Nothing to be declared
Tanner Felix C	**2024**	**Ongoing research funding from public and/or not-for-profit organisations**<br>Swiss Heart Foundation<br>**Any other interest (financial or otherwise) that should be declared in view of holding an ESC position**<br>Secretary, UEMS Cardiac Section Board, Swiss Institute for Medical Education Board, Swiss Heart Foundation Editorial Board, International Journal of Cardiology Editorial Board, Journal of Clinical Medicine Editorial Board, Swiss Medical Weekly<br>**Total impact on personal income or income to an entity controlled personally**<br>0 (none) - 10,000EUR/year

Expert	Type of Relationship with Industry	
Tanner Felix C	**2025**	**Ongoing research funding from public and/or not-for-profit organisations**<br>Swiss Heart Foundation<br>**Positions of influence in non-ESC organisations and non-ESC journals with a focus in the field of cardiovascular medicine/sciences**<br>Leadership positions:UEMS Cardiac Section, Secretary Swiss Institute of Medical Education, Board Member Swiss Society of Cardiology, Education Committee, Member Swiss Society of Cardiology, Cardiac Imaging Working Group, Board Member Swiss Heart Foundation, Board Member<br>Other editorial positions:International Journal of Cardiology, Editorial Board, Member Reviews in Cardiovascular Medicine, Editorial Board, Member Journal of Clinical Medicine, Editorial Board, Member Swiss Medical Weekly, Academic Editor<br>**Any other interest (financial or otherwise) that should be declared in view of holding an ESC position**<br>Secretary, UEMS Cardiac Section Board, Swiss Institute for Medical Education Board, Swiss Heart Foundation Editorial Board, International Journal of Cardiology Editorial Board, Journal of Clinical Medicine Editorial Board, Swiss Medical Weekly<br>**Total impact on personal income or income to an entity controlled personally**<br>0 (none) - 10,000EUR/year
Terkelsen Christian Juhl	**2024**	**Payment from healthcare industry**<br>Edwards Lifesciences : Device Therapy<br>Medtronic : Device Therapy<br>Meril A/S : Device Therapy<br>**Funding from healthcare industry for travel to, and attendance of events and meetings, unrelated to any specific activity**<br>Edwards Lifesciences : Device Therapy<br>Medtronic : Device Therapy<br>Meril A/S : Device Therapy<br>**Research funding from healthcare industry received personally**<br>Medtronic<br>Meril A/S<br>**Total impact on personal income or income to an entity controlled personally**<br>>10,000EUR/year

Expert		Type of Relationship with Industry
Terkelsen Christian Juhl	**2025**	**Ongoing research funding from public and/or not-for-profit organisations**<br>Danish Heart Foundation<br>Novo Nordic Foundation<br>**Payment from healthcare industry**<br>Edwards Lifesciences : Interventional Cardiology<br>Meril Lifesciences : Interventional Cardiology<br>Medtronic A/S : Interventional Cardiology<br>**Research funding from healthcare industry received personally**<br>Meril Lifesciences<br>Medtronic A/S<br>**Total impact on personal income or income to an entity controlled personally**<br>>10,000EUR/year
Theotonio Rita	**2024**	**Funding from healthcare industry for travel to, and attendance of events and meetings, unrelated to any specific activity**<br>Amgen : Acute Coronary Syndromes<br>Abbott : Interventional Cardiology<br>Boston Scientific : Interventional Cardiology<br>**Total impact on personal income or income to an entity controlled personally**<br>0 (none) - 10,000EUR/year
	**2025**	**Funding from healthcare industry for travel to, and attendance of events and meetings, unrelated to any specific activity**<br>Medtronic : Acute Coronary Syndromes<br>Novartis : Acute Coronary Syndromes, Risk Factors and Prevention<br>**Total impact on personal income or income to an entity controlled personally**<br>0 (none) - 10,000EUR/year
Thunstrom Erik	**2024**	Nothing to be declared
	**2025**	**Research funding from healthcare industry received by department/institution or an entity controlled by department/institution, under direct/personal responsibility**<br>Novartis : I am doing a clinical registry study on the compliance to inclisiran in Sweden , Chief investigator

Expert		Type of Relationship with Industry
Thunstrom Erik	**2025**	**Positions of influence in non-ESC organisations and non-ESC journals with a focus in the field of cardiovascular medicine/sciences**<br>Leadership positions:I am bord member of the Swedish national society of cardiology<br>**Total impact on personal income or income to an entity controlled personally**<br>0 (none) - 10,000EUR/year
Tomaniak Mariusz	**2024**	**Ongoing research funding from public and/or not-for-profit organisations**<br>Polish Cardiac Society (Research grant following external review)<br>Polish National Science Center (NCN) - Research grant following external review<br>**Payment from healthcare industry**<br>Abbott : Other<br>Polpharma : Other<br>**Total impact on personal income or income to an entity controlled personally**<br>0 (none) - 10,000EUR/year
	**2025**	**Ongoing research funding from public and/or not-for-profit organisations**<br>Polish Cardiac Society (Research grant following external review)<br>Polish National Science Center (NCN) - Research grant following external review<br>**Payment from healthcare industry**<br>Abbott : Other<br>Polpharma : Other<br>Shockwave medical : Other<br>**Research funding from healthcare industry received by department/institution or an entity controlled by department/institution, under direct/personal responsibility**<br>Shockwave medical : research grant to institution for an investigator-initiated study , principal investigator<br>**Positions of influence in non-ESC organisations and non-ESC journals with a focus in the field of cardiovascular medicine/sciences**<br>Leadership positions:President-Elect of the Club 30 of the Polish Cardiac Society (2025-2027) Treasurer of the Association for Cardiovascular Interventions of the Polish Cardiac Society (2025-2027)<br>**Total impact on personal income or income to an entity controlled personally**<br>0 (none) - 10,000EUR/year

Expert	Type of Relationship with Industry	
Toutouzas Konstantinos	**2024**	**Ongoing research funding from public and/or not-for-profit organisations**<br>Hellenic Society of Cardiology<br>**Payment from healthcare industry**<br>Abbott : Interventional Cardiology<br>Bayer : Interventional Cardiology<br>Medtronic : Interventional Cardiology<br>Biotronik : Interventional Cardiology<br>Gore : Interventional Cardiology<br>Meril Myval : Interventional Cardiology<br>Astra Zeneca : Pharmacology and Pharmacotherapy<br>Boehringer-Ingelheim : Pharmacology and Pharmacotherapy<br>Pfizer : Pharmacology and Pharmacotherapy<br>Sanofi Aventis : Pharmacology and Pharmacotherapy<br>**Payment from healthcare industry to department or institution or any other body for personal services**<br>Meril Myval : Interventional Cardiology<br>Chiesi Pharma : Pharmacology and Pharmacotherapy<br>Pfizer : Research Methodology<br>**Total impact on personal income or income to an entity controlled personally**<br>0 (none) - 10,000EUR/year

Expert	Type of Relationship with Industry	
Toutouzas Konstantinos	**2025**	**Ongoing research funding from public and/or not-for-profit organisations**<br>Hellenic Society of Cardiology<br>**Payment from healthcare industry**<br>Pfizer : Cardiovascular Disease in Special Populations<br>Abbott : Interventional Cardiology<br>Bayer : Interventional Cardiology<br>Medtronic : Interventional Cardiology<br>Biotronik : Interventional Cardiology<br>Gore : Interventional Cardiology<br>Meril Myval : Interventional Cardiology<br>Astra Zeneca : Pharmacology and Pharmacotherapy<br>Boehringer-Ingelheim : Pharmacology and Pharmacotherapy<br>Pfizer : Pharmacology and Pharmacotherapy<br>Sanofi Aventis : Pharmacology and Pharmacotherapy<br>**Payment from healthcare industry to department or institution or any other body for personal services**<br>Meril Myval : Interventional Cardiology<br>Chiesi Pharma : Pharmacology and Pharmacotherapy<br>Pfizer : Research Methodology<br>**Total impact on personal income or income to an entity controlled personally**<br>0 (none) - 10,000EUR/year

Expert	Type of Relationship with Industry	
Trendafilova Elina	**2024**	**Payment from healthcare industry**<br>Pfizer : Atrial Fibrillation (AF)<br>Teva Pharmaceutical Industries : Atrial Fibrillation (AF) , Chronic Heart Failure , Acute Heart Failure , Coronary Artery Disease (Chronic) / Chronic Coronary Syndromes (CCS) , Acute Coronary Syndromes , Risk Factors and Prevention<br>Bayer : Chronic Heart Failure<br>Astra Zeneca : Chronic Heart Failure, Acute Heart Failure<br>Boehringer-Ingelheim : Chronic Heart Failure, Acute Heart Failure<br>Novartis : Chronic Heart Failure, Acute Heart Failure, Risk Factors and Prevention<br>**Funding from healthcare industry for travel to, and attendance of events and meetings, unrelated to any specific activity**<br>Astra Zeneca : Chronic Heart Failure, Acute Heart Failure, European Society of Cardiology<br>Novartis : European Society of Cardiology<br>**Research funding from healthcare industry received personally**<br>Novo-Nordisk<br>**Total impact on personal income or income to an entity controlled personally**<br>>10,000EUR/year
	**2025**	**Payment from healthcare industry**<br>Teva Pharmaceutical Industries : Atrial Fibrillation (AF), Chronic Heart Failure, Acute Heart Failure, Coronary Artery Disease (Chronic) / Chronic Coronary Syndromes (CCS), Acute Coronary Syndromes, Risk Factors and Prevention<br>Astra Zeneca : Chronic Heart Failure, Acute Heart Failure<br>Boehringer-Ingelheim : Chronic Heart Failure, Acute Heart Failure<br>Novartis : Chronic Heart Failure, Acute Heart Failure, Risk Factors and Prevention<br>Menarini : Hypertension<br>Swixx Biopharma : Myocardial Disease<br>**Funding from healthcare industry for travel to, and attendance of events and meetings, unrelated to any specific activity**<br>Novartis : European Society of Cardiology<br>**Research funding from healthcare industry received personally**<br>Novo-Nordisk<br>**Positions of influence in non-ESC organisations and non-ESC journals with a focus in the field of cardiovascular medicine/sciences**<br>Leadership positions:Co-chair of Bulgarian heart and vessels association<br>**Total impact on personal income or income to an entity controlled personally**<br>>10,000EUR/year

Expert		Type of Relationship with Industry
Van Belle Eric	2024	**Ongoing research funding from public and/or not-for-profit organisations**<br>Programme Hospitalier de Recherhce Clinique (PHRC) - French Ministry of Health funding<br>**Total impact on personal income or income to an entity controlled personally**<br>0 (none) - 10,000EUR/year
	2025	**Ongoing research funding from public and/or not-for-profit organisations**<br>Programme Hospitalier de Recherhce Clinique (PHRC) - French Ministry of Health funding<br>**Positions of influence in non-ESC organisations and non-ESC journals with a focus in the field of cardiovascular medicine/sciences**<br>Leadership positions:past president of GACI - a branch of the French Society of Cardiology<br>Positions as Editor in Chief:NA<br>Other editorial positions:NA<br>**Total impact on personal income or income to an entity controlled personally**<br>0 (none) - 10,000EUR/year
Zavatta Marco	2024	Nothing to be declared
	2025	Nothing to be declared
Zeppenfeld Katja	2024	Nothing to be declared
	2025	Nothing to be declared
Zied Ben El Hadj	2024	**Payment from healthcare industry**<br>Hikma : Chronic Heart Failure<br>Boehringer-Ingelheim : Chronic Heart Failure, Coronary Artery Disease (Chronic) / Chronic Coronary Syndromes (CCS), Acute Coronary Syndromes, Myocardial Disease<br>galpharma : Coronary Artery Disease (Chronic) / Chronic Coronary Syndromes (CCS), Acute Coronary Syndromes<br>Menarini : Hypertension<br>**Funding from healthcare industry for travel to, and attendance of events and meetings, unrelated to any specific activity**<br>KT BIO : Clinical Skills, Coronary Artery Disease (Chronic) / Chronic Coronary Syndromes (CCS)<br>Philadelphia : Hypertension<br>**Total impact on personal income or income to an entity controlled personally**<br>0 (none) - 10,000EUR/year
	2025	**Payment from healthcare industry**<br>Boehringer-Ingelheim : Acute Coronary Syndromes

Expert	Year	Type of Relationship with Industry
Zied Ben El Hadj	**2025**	**Total impact on personal income or income to an entity controlled personally**<br>0 (none) - 10,000EUR/year
Ztot Samir	**2024**	Nothing to be declared
	**2025**	Nothing to be declared
